# Identification of early salt stress responsive proteins in seedling roots of upland cotton (*Gossypium hirsutum* L.) employing iTRAQ-based proteomic technique

**DOI:** 10.3389/fpls.2015.00732

**Published:** 2015-09-11

**Authors:** Wu Li, Fu'an Zhao, Weiping Fang, Deyi Xie, Jianan Hou, Xiaojie Yang, Yuanming Zhao, Zhongjie Tang, Lihong Nie, Shuping Lv

**Affiliations:** ^1^College of Life Sciences, Henan UniversityKaifeng, China; ^2^Economic Crop Research Institute, Henan Academy of Agricultural SciencesZhengzhou, China

**Keywords:** *Gossypium hirsutum*, salt stress, iTRAQ, root, proteomics

## Abstract

Soil salinity is a major abiotic stress that limits plant growth and agricultural productivity. Upland cotton (*Gossypium hirsutum* L.) is highly tolerant to salinity; however, large-scale proteomic data of cotton in response to salt stress are still scant. Here, an isobaric tag for relative and absolute quantitation (iTRAQ)-based proteomic technique was employed to identify the early differentially expressed proteins (DEPs) from salt-treated cotton roots. One hundred and twenty-eight DEPs were identified, 76 of which displayed increased abundance and 52 decreased under salt stress conditions. The majority of the proteins have functions related to carbohydrate and energy metabolism, transcription, protein metabolism, cell wall and cytoskeleton metabolism, membrane and transport, signal transduction, in addition to stress and defense. It is worth emphasizing that some novel salt-responsive proteins were identified, which are involved in cell cytoskeleton metabolism (actin-related protein2, ARP2, and fasciclin-like arabinogalactan proteins, FLAs), membrane transport (tonoplast intrinsic proteins, TIPs, and plasma membrane intrinsic proteins, PIPs), signal transduction (leucine-rich repeat receptor-like kinase encoding genes, LRR-RLKs) and stress responses (thaumatin-like protein, TLP, universal stress protein, USP, dirigent-like protein, DIR, desiccation-related protein PCC13-62). High positive correlation between the abundance of some altered proteins (superoxide dismutase, SOD, peroxidase, POD, glutathione S-transferase, GST, monodehydroascorbate reductase, MDAR, and malate dehydrogenase, MDH) and their enzyme activity was evaluated. The results demonstrate that the iTRAQ-based proteomic technique is reliable for identifying and quantifying a large number of cotton root proteins. qRT-PCR was used to study the gene expression levels of the five above-mentioned proteins; four patterns are consistent with those of induced protein. These results showed that the proteome of cotton roots under NaCl stress is complex. The comparative protein profiles of roots under salinity vs control improves the understanding of the molecular mechanisms involved in the tolerance of plants to salt stress. This work provides a good basis for further functional elucidation of these DEPs using genetic and/or other approaches, and, consequently, candidate genes for genetic engineering to improve crop salt tolerance.

## Introduction

Soil salinity is one of the most important environmental factors limiting plant growth and productivity throughout the world (Munns, 2002). Excessive Na^+^ in the soil inhibits the absorption of mineral nutrients and moisture leading to the accumulation of toxic ions in plants. Plants employ several strategies to cope with salt stress. These include regulating the expression of specific proteins for the reestablishment of proper cellular ion and osmotic homeostasis with other concomitant processes of repair and detoxification (Chinnusamy et al., 2005). The salt signal is primarily perceived through roots, which rapidly respond to maintain function and transmit signals to the shoot for appropriate changes in function (Zhao et al., 2013). Salt-tolerance studies in plants provide insights into the molecular and biochemical basis of plant stress tolerance, which ultimately lead to crop improvement.

Upland Cotton (*Gossypium hirsutum* L.) is one of the most important textile fiber crops. Although cotton has a higher basal level of tolerance to NaCl compared with other major crops (Maas and Hoffman, 1977), its growth, yield and fiber quality are adversely affected, especially at germination and at the young seedling stage (Ahmad et al., 2002). Understanding the molecular mechanism of salt tolerance can provide many candidate genes for genetic engineering. Many salt-resistant genes have been identified in model plants but only a few salt stress-inducible genes, e.g. Na^+^/H^+^antiporter (Wu et al., 2004), *DREB* (Gao et al., 2009), *ERF* (Champion et al., 2009; Jin et al., 2010), *NAC* (Meng et al., 2009), *GhMT3a* (Xue et al., 2009), *MPK* (Zhang et al., 2011), *MKK* (Lu et al., 2013), and *ZFP* (Guo et al., 2009), have been documented in cotton. Recently, with advances in transcriptome mapping (high-throughput sequencing), some salt-responsive genes and molecular regulatory pathways have been identified in cotton (Yao et al., 2011; Wang et al., 2012; Xu et al., 2013). These studies provide relevant information about the stress-responsive genes, but the transcriptome data may not correlate with results from proteomic analysis due to post-transcriptional and post-translational modifications. Therefore, it is necessary to investigate the change of proteins under salt stress conditions to be able to understand the adaptive mechanism of salt tolerance in cotton.

Proteomic analysis is a tool that facilitates the study of global protein expression and provides a large amount of information about the individual proteins involved in specific biological responses. It has been used to analyze salt stress induced alterations in the root proteome of plant species, such as *Arabidopsis* (Jiang et al., 2007), rice (Chitteti and Peng, 2007; Cheng et al., 2009), barley (Witzel et al., 2009), wheat (Peng et al., 2009; Guo et al., 2012a), maize (Zörb et al., 2010), soybean (Aghaei et al., 2009), tomato (Manaa et al., 2011; Gong et al., 2014), cucumber (Du et al., 2010), and salt cress (Zhou et al., 2010). Over 850 DEPs of salt-stressed roots have been identified in the above-mentioned studies. Many previous studies relied upon 2D gel electrophoresis data; however, it is difficult to identify low abundant proteins, proteins with low (< 15 kDa) or high (>150 kDa) molecular weights, proteins that are excessively acidic or basic as well as hydrophobic proteins (Zieske, 2006). Non-gel-based quantitative proteomics techniques established in recent years have overcome some of the drawbacks of the above-mentioned method. iTRAQ is a mass spectrometry-based proteomics technique that can be used to evaluate cell metabolic differences. Zhu et al. (2009) employed iTRAQ to reveal functional differentiation of *Brassica napus* guard cells and mesophyll cells. iTRAQ can also be used to investigate plant responses to deficient or excess mineral nutrients. For example, Yang et al. (2013) successfully analyzed the protein profile of *Citrus sinensis* roots in response to long-term boron-deficiency with iTRAQ. In addition, Fukao et al. (2011) used iTRAQ analysis to reveal mechanisms of growth defects due to excess zinc in *Arabidopsis*. It can retain information on the post-translational modification (PTM), simultaneously analyze multiple samples, help to quantify proteins not amenable to the 2D gel approach (Wang et al., 2014) and relatively quantify peptides at a global level (Ghosh et al., 2013). Gong et al. (2014) used iTRAQ to identify a set of DEPs in tomato roots exposed to salt and alkali stress. However, large-scale proteomic data of cotton roots in response to salt stress has not been reported in previous studies. In this present study, an iTRAQ-based proteomic technique was used to identify the early DEPs in order to elucidate the effects of salt stress in cotton seedling roots treated with NaCl for 24 h.

## Materials and methods

### Plant culture and salt treatments

Seeds of ZMS23, a salt-tolerant variety, were obtained from the Institute for Cotton Research of the Chinese Academy of Agriculture Science. Cotton seeds were sterilized with 10% H_2_O_2_ for 30 min and rinsed with distilled water. The sterilized seeds were germinated on filter paper soaked in distilled water in Petri dishes at 26°C. After 7 days, 54 uniform germinated seedlings were transferred to six plastic containers (48 × 36 × 15 cm) and each contained nine seedlings, which were filled with Hoagland's solution (5 mM Ca(NO_3_), 3 mM KNO_3_, 2 mM MgSO_4_, 0.5 mM KH_2_PO_4_, 2.5 μM FeNa_2_(EDTA), 2.5 μM H_3_BO_3_, 5 μM MnC1_2_, 0.5 μM ZnSO_4_, 0.3 μM CuSO_4_, and 0.05 μM (NH_4_)_6_MoO_24_), and renewed every 2 days. The experiment was performed in a climate chamber under the following conditions: 28/23°C day/night temperature, relative humidity of 70–80% and a 14 h light period/day at an intensity of 600 μmol m^−2^ s^−1^. When the plants grew to the trefoil stage, three plastic containers (including 27 seedlings) were renewed with Hoagland's solution and 200 mM NaCl was added, but no NaCl was added to the other three containers used as a control for 24 h, respectively. After treatment, the 1–5 cm portions of root tips were harvested and frozen at −80°C. In the same way, another biological repeat was carried out.

### Protein extraction

Cotton roots (approximately 1 g) were immersed in liquid nitrogen and ground to a fine powder. Four milliliter of lysis buffer (5 mM Tris-HCl, pH 7.4, 1 mM PMSF, 2 mM EDTA, 10 mM DTT, and 1%TritonX-100) was added to the powder and subjected to ultrasonic vibrations for 15 min. The supernatant was transferred to a 50 mL tube after centrifugation at 25,000 g for 20 min; then, five volumes of cold acetone was added and incubated at −20°C for 2 h. Thereafter, the tubes were centrifuged at 16,000 g for 20 min and the supernatants discarded. The pellets were resuspended in the lysis buffer and centrifuged as described above. Finally, the protein pellets were washed twice with 30 ml of ice-cold acetone, lyophilized and stored at −80°C.

### Protein digestion, iTRAQ labeling and strong cation exchange

iTRAQ analysis was performed at the Beijing Genomics Institute (BGI, Shenzhen, China). Protein samples (100 μg of each protein) were reduced with 10 mM DTT at 56°C for 2 h, alkylated with 55 mM iodoacetamide at room temperature in the dark for 45 min, digested with trypsin at 20:1 mass ratio at 37°C for 12 h, then labeled using the iTRAQ Reagents 8-plex kit according to the manufacturer's instructions (AB Sciex Inc., MA, USA). The salt-treated samples' replicates were labeled with iTRAQ tags 113, 114, and the untreated labeled with tags 115, 116, respectively. After labeling, the samples were mixed and lyophilized before dissolving in 4 mL of strong cation exchange (SCX) buffer A (25 mM NaH_2_PO_4_ in 25% acetonitrile pH2.7). The peptides were fractionated on Ultremex SCX column (4.6 × 250 mm) using Shimadzu LC-20AB HPLC. The subsequent experiment was performed as described in Zhu et al. (2009).

### Tandem mass spectrometry analysis

The fractionated samples were lyophilized to remove acetonitrile and resuspended in Solvent A (5% acetonitril, 0.1% formic acid). Peptide samples, 5 μL (2.5 μg) each were loaded onto a C18LC Packings PepMap trap column and separated on a PepMapC18 75 μm inner diameter (LC Packings) column at a flow rate of 300 nl/min using Shimadzu LC-20AD HPLC. Peptides were eluted from the HPLC column by a linear gradient from 2% buffer B (95% acetonitrile, 1% formic acid) to 35% for 40 min, followed by ramping up to 80% buffer B for 5 min, and then held on 80% buffer B for 4 min. Peptides separated by liquid chromatography were sprayed into the orifice of the Q-Exactive mass spectrometer (Thermo Fisher Scientific, San Jose, CA, USA) and subsequently analyzed according to previously described methods (Qiao et al., 2012).

### Data analysis and protein identification

The MS data were performed using Proteome Discoverer 1.3 software (Thermo Fisher Scientific, San Jose, CA, USA). Relative abundance quantitation and protein identification were processed using Mascot 2.3.02 (Matrix Science, London, United Kingdom). The analysis was carried out with cotton AD genome annotation database (81147 sequences) and the National Center for Biotechnology Information (NCBI) non-redundant fasta database (6833826 sequences). The search parameters were set as follows: Type of search: MS/MS Ion search; Enzyme: Trypsin with one missed cleavage; Monoisotopic mass; Tragment Mass Tolerance: 0.02Da; Peptide Mass Tolerance: 15 ppm; oxidation of methionine and tyrosine labeled by iTRAQ 8-plex as variable modifications, while carbamidomethylation on cysteine, iTRAQ 8-plex labeled N-term and lysine as fixed modifications. False discovery rate (FDR) correction was adopted with a threshold of 0.01 to reduce the false identification of peptide, and a Mascot probability of 95% was set for the identification and quantification of protein. Protein identification was considered if at least one unique peptide was identified for each protein.

### Go and KEGG analysis

Differentially expressed proteins were classified according to Gene Ontology (http://www.geneontology.org). Kyoto Encyclopedia of Genes and Genomes (KEGG) (http://www.genome.jp/kegg/ or http://www.kegg.jp/) was used to predict molecular function, biological processes and significant pathways involved in response to salt stress.

### Measurement of enzyme activities

The activities of SOD, POD and MDAR were assayed according to Chen et al. (2008). GST and MDH were extracted and assayed according to Gronwald et al. (1987) and Chen et al. (2009), respectively.

### qRT-PCR analysis

Total RNA was extracted from salt-treated and control cotton roots by Trizol reagent (TaKaRa), and cDNA was reverse transcribed from 1 μg of to total RNA using a First Strand cDNA Synthesis Kit (Invitrogen). Gene-specific primers (GSPs) used for qRT-PCR were designed using primer3 (http://primer3.ut.ee/) according to cDNA sequences obtained from the cotton (Table S1). The cotton 18s-rRNA gene was used as an endogenous control for normalization. The PCR reaction was carried out in a 20 uL volume containing 10 μL 2 × SYBR Green Master Mix reagent (TaKaRa), 1 μL template cDNA and 0.5 μL of each GSPs with the following reaction conditions: 95°C for 30 s; followed by 40 cycles of 95°C for 10 s; 55°C for 10 s and 72°C for 15 s. Relative gene expression was calculated using the ddCt alogorithm (Zhang et al., 2003).

## Results

### Primary data analysis and protein detection

**Figure 1:**
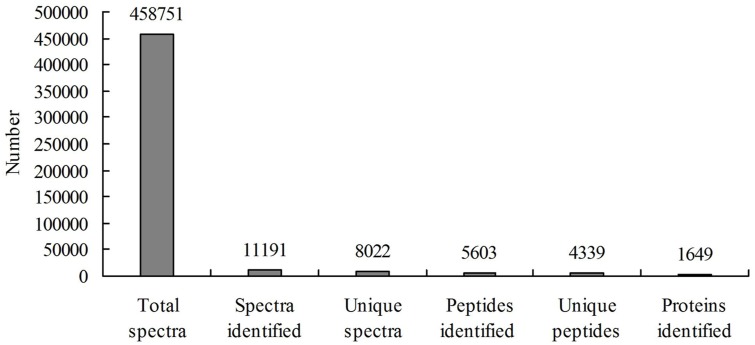
**Spectra, peptides and proteins identified from iTRAQ proteomics after searching against the sequence databases**.

**Figure 2:**
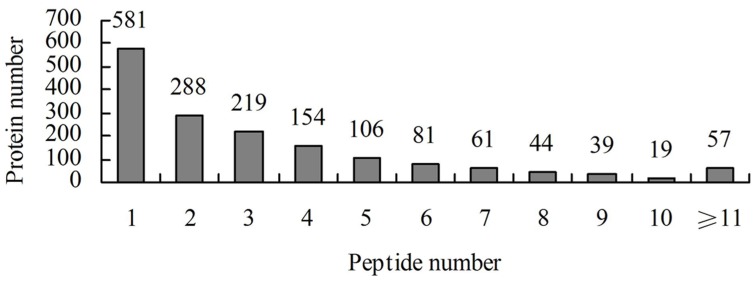
**Number of peptides that were matched to proteins using MASCOT**.

**Figure 3:**
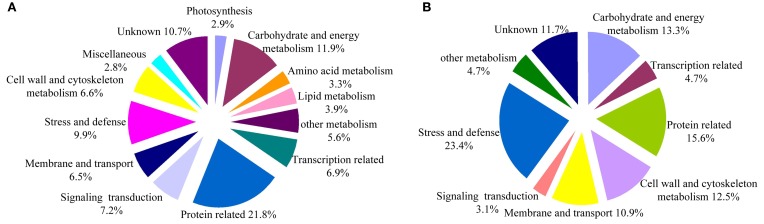
**Functional classification of the identified proteins**. **(A)** All 1649 proteins. **(B)** Differentially expressed proteins in salt stress cotton roots as compared to the control. The percentage for each class is shown and represented in the pie-chart.

A total of 458,751 spectra were generated from the iTRAQ experiment using the proteins of salt-treated and untreated roots as materials. The data were analyzed using Mascot software (version 2.3.02). Mascot detected a total of 11,191 spectra matched to known spectra, 8022 spectra matched to unique spectra, 5603 peptides, 4339 unique peptides, and 1649 proteins (Figure 1). The distribution of the number of peptides defining each protein is shown in Figure 2 and over 64.7% of the proteins included at least two peptides. These proteins were involved in multiple metabolic, regulatory and defense pathways (Figure 3A, Table S2).

### Identification and functional classification of DEPs

DEPs were selected based on the following criteria: (i) proteins in which the mean ratio {corresponding to the protein reporter ion intensity originating from salt-treated protein samples (113 and 114) with respect to fully control protein samples (115 and 116)} had a 1.5 fold change; (ii) a *p* < 0.05. Based on these criteria, 128 DEPs were identified in cotton roots, 76 (59.4%) of which displayed increased, and 52 (40.6%) decreased abundance under salt stress conditions. The main biological functions for the 128 DEPs were: carbohydrate and energy metabolism (13.3%), transcription related (4.7%), protein metabolism (15.6%), cell wall and cytoskeleton metabolism (12.5%), membrane, and transport (10.9%), signal transduction (3.1%), and stress and defense (23.4%). In addition, six proteins were involved in other metabolic processes (4.7%) and 15 in unknown biological processes (11.7%). Detailed information can be found in Figure 3B, Table 1, Figure S1 and Table S3.

**Table 1:** **Differentially expressed proteins in cotton roots subject to salt stress (200 mM NaCl)**.

	**Accession**	**Proteins**	**Species**	**Percent coverage**	**No. of unique peptide**	**Mean ratio^a^**	**Up/down^b^**
**CARBOHYDRATE AND ENERGY METABOLISM**							
1	gi|377824753	Pectin methylesterase	*Gossypium hirsutum*	11.6	4	0.666	↓
2	gi|211906490	Malate dehydrogenase	*Gossypium hirsutum*	28.9	2	0.664	↓
3	gi|122216326	Perakine reductase	*Rauwolffia serpentina*	28.0	3	0.666	↓
4	gi|339265919	Phosphogluconate dehydrogenase	*Lotus grandiflorus*	17.9	3	1.521	↑
5	gi|225455555	Enolase	*Glycine max*	38.5	4	0.679	↓
6	gi|55584187	Quinone oxidoreductase-like protein	*Arabidopsis thaliana*	20.8	4	0.623	↓
7	gi|117940179	Dihydrolipoyllysine-residue Acetyltransferase component 1 of pyruvate dehydrogenase complex, mitochondrial	*Arabidopsis thaliana*	10.5	1	1.625	↑
8	gi|225465847	NADPH: quinone oxidoreductase	*Vitis vinifera*	25.9	3	0.570	↓
9	gi|75262456	ATP-citrate synthase beta chain protein 2	*Arabidopsis thaliana*	21.9	3	0.671	↓
10	gi|242129048	ATP synthase delta subunit 2	*Gossypium hirsutum*	21.9	2	0.566	↓
11	gi|91981275	Pectin methylesterase	*Citrus bergamia*	2.1	1	0.571	↓
12	gi|21431800	NADP-dependent alkenal double bond reductase P2	*Arabidopsis thaliana*	10.7	2	0.576	↓
13	gi|75268018	Probable fructose-bisphosphate aldolase 3	*Arabidopsis thaliana*	5.6	2	1.485	↑
14	gi|470127114	Aldose 1-epimerase-like	*Fragaria vesca* subsp. *vesca*	13.1	4	1.517	↑
15	gi|37193998	Phosphoenolpyruvate carboxykinase	Mitella japonica	6.6	3	0.466	↓
16	gi|224057577	Glutathione reductase	*Populus trichocarpa*	7.5	3	1.992	↑
17	gi|356532527	Dihydrolipoyl dehydrogenase-like	*Glycine max*	8.3	2	0.503	↓
**TRANSCRIPTION RELATED**							
18	gi|75262442	Nuclear transcription factor Y subunit B-2	*Arabidopsis thaliana*	15.3	5	1.511	↑
19	gi|341958560	CASP-like protein	*Populus trichocarpa*	7.5	1	0.605	↓
20	gi|356521678	Putative DNA repair protein RAD23-1-like isoform 1	*Glycine max*	7.8	1	1.750	↑
21	gi|55976204	Transcription factor HY5	*Solanum lycopersicum*	10.1	1	2.103	↑
22	gi|224133758	Histone H1	*Populus trichocarpa*	3.8	1	1.846	↑
23	gi|75321585	Zinc finger CCCH domain-containing protein 40	*Oryza sativa* subsp. *japonica*	2.0	1	1.681	↑
**PROTEIN TRANSLATION, PROCESSING, AND DEGRADATION**							
24	gi|75266342	40S ribosomal protein S20-2	*Arabidopsis thaliana*	9.8	1	2.103	↑
25	gi|22096379	40S ribosomal protein S30	*Arabidopsis thaliana*	7.4	1	0.674	↓
26	gi|17865566	60S ribosomal protein L36-3	*Arabidopsis thaliana*	21.8	2	1.743	↑
27	gi|24473796	60s acidic ribosomal protein	*Prunus dulcis*	57.0	2	0.645	↓
28	gi|6015064	Elongation factor 1-delta	*Pimpinella brachycarpa*	26.0	2	2.089	↑
29	gi|23503072	Eukaryotic translation initiation factor 3 subunit I	*Arabidopsis thaliana*	15.3	3	0.666	↓
30	gi|18803	Polyubiquitin protein	*Helianthus annuus*	16.2	4	0.675	↓
31	gi|95116512	Ubiquitin activating enzyme	*Theobroma cacao*	3.8	3	0.564	↓
32	gi|356560787	Ubiquitin-conjugating enzyme E2 5-like	*Glycine max*	10.3	2	1.543	↑
33	gi|117949833	T-complex protein 1 subunit gamma	*Arabidopsis thaliana*	8.4	4	1.578	↑
34	gi|75115360	Protein disulfide isomerase-like 1-6	*Arabidopsis thaliana*	3.2	2	1.911	↑
35	gi|255541132	Structural constituent of nuclear pore	*Ricinus communis*	4.8	3	1.680	↑
36	gi|255575861	Glycolipid transfer protein	*Ricinus communis*	4.1	1	1.972	↑
37	gi|82581521	Proteasome subunit beta type-4	*Arabidopsis thaliana*	5.4	2	1.526	↑
38	gi|211906494	Heat shock protein 70	*Gossypium hirsutum*	32.8	1	0.654	↓
39	gi|211906504	Heat shock protein 70	*Gossypium hirsutum*	37.2	1	0.675	↓
40	gi|289064666	S-adenosylmethionine synthase-like protein	*Eperua falcata*	34.6	1	0.536	↓
41	gi|255543963	Aspartic proteinase nepenthesin-1 precursor	*Ricinus communis*	19.8	5	1.819	↑
42	gi|229830633	L-idonate 5-dehydrogenase	*Vitis vinifera*	3.8	1	0.601	↓
43	gi|308743337	Asparagine synthetase 1	*Solanum tuberosum*	6.6	3	0.537	↓
**CELL WALL AND CYTOSKELETON METABOLISM**							
44	gi|89212812	Actin depolymerizing factor 2	*Gossypium hirsutum*	44.4	4	1.524	↑
45	gi|117553550	Actin-binding protein ABP29	*Vitis vinifera*	15.9	1	1.663	↑
46	gi|182627650	Actin-related protein4	*Oryza sativa* subsp. *indica*	6.0	3	1.503	↑
47	gi|157273642	Fasciclin-like arabinogalactan protein 4	*Gossypium hirsutum*	28.3	1	0.603	↑
48	gi|150416583	Fasciclin-like arabinogalactan protein 11	*Gossypium hirsutum*	28.3	1	0.652	↑
49	gi|157273666	Fasciclin-like arabinogalactan protein 16	*Gossypium hirsutum*	13.0	3	1.563	↓
50	gi|157273646	Fasciclin-like arabinogalactan protein 6	*Gossypium hirsutum*	15.9	3	1.711	↑
51	gi|157273640	Fasciclin-like arabinogalactan protein 3	*Gossypium hirsutum*	12.0	2	1.574	↑
52	gi|157273638	Fasciclin-like arabinogalactan protein 2	*Gossypium hirsutum*	14.0	3	3.059	↑
53	gi|157273660	Fasciclin-like arabinogalactan protein 13	*Gossypium hirsutum*	5.6	2	2.124	↑
54	gi|253509569	Caffeic acid O-methyltransferase 2	*Gossypium hirsutum*	23.0	5	0.682	↓
55	gi|224552010	Hybrid proline-rich protein	*Gossypium hirsutum*	5.2	1	3.507	↑
56	gi|255547195	Glycine-rich RNA-binding protein	*Ricinus communis*	27.3	1	1.502	↑
**MEMBRANE AND TRANSPORT**							
57	gi|1336803	Vacuolar H(+)-ATPase subunit A	*Gossypium hirsutum*	48.5	2	1.635	↑
58	gi|2493146	V-type proton ATPase 16 kDa proteolipid subunit	*Gossypium hirsutum*	10.9	2	2.155	↑
59	gi|75273758	Cysteine-rich repeat secretory protein 38	*Arabidopsis thaliana*	22.7	3	0.628	↓
60	gi|7105717	Plasma membrane proton ATPase	*Kosteletzkya virginica*	17.5	4	1.732	↑
61	gi|224130846	Multidrug/pheromone exporter, MDR family, ABC transporter family	*Populus trichocarpa*	6.6	3	1.699	↑
62	gi|292653531	Aquaporin TIP1;7	*Gossypium hirsutum*	6.3	1	0.641	↓
63	gi|292653547	Aquaporin TIP2;5	*Gossypium hirsutum*	4.4	1	0.658	↓
64	gi|461929	Probable aquaporin TIP-type	*Antirrhinum majus*	7.0	1	0.503	↓
65	gi|300793602	TPA: TPA_inf: aquaporin TIP1;4	*Gossypium hirsutum*	6.8	1	0.451	↓
66	gi|292653535	Aquaporin TIP1;10	*Gossypium hirsutum*	6.2	1	0.506	↓
67	gi|300793598	Aquaporin PIP2;10	*Gossypium hirsutum*	21.5	2	0.291	↓
68	gi|256568429	PIP protein	*Gossypium hirsutum*	20.7	2	0.541	↓
69	gi|164668308	PIP2 protein	*Gossypium hirsutum*	10.2	2	0.522	↓
70	gi|118132686	PIP1 protein	*Gossypium hirsutum*	3.5	1	0.270	↓
**SIGNAL TRANSDUCTION**							
71	gi|363807628	Probable leucine-rich repeat receptor-like protein kinase	*Glycine max*	9.9	3	1.454	↑
72	gi|1702983	Auxin-repressed 12.5 kDa protein	*Fragaria ananassa*	69.4	4	1.586	↑
73	gi|349504495	Leucine rich repeat-containing protein	*Corchorus capsularis*	1.5	2	1.572	↑
74	gi|1346675	Nucleoside diphosphate kinase B	*Flaveria bidentis*	16.9	2	1.523	↑
**STRESS AND DEFENSE**							
75	gi|74229677	Cytoplasmic Cu/ZnSOD	*Gossypium hirsutum*	16.5	1	2.940	↑
76	gi|357470271	Peroxidase	*Medicago truncatula*	29.7	8	1.751	↑
77	gi|115345276	Peroxidase	*Populus alba*	6.5	1	1.667	↑
78	gi|73913500	Peroxidase	*Phaseolus lunatus*	19.9	5	1.673	↑
79	gi|255551599	Peroxidase 26 precursor	*Ricinus communis*	12.2	2	1.981	↑
80	gi|32351452	Class III peroxidase	*Gossypium hirsutum*	10.6	2	1.805	↑
81	gi|255581003	Peroxidase 2 precursor	*Ricinus communis*	19.8	4	1.643	↑
82	gi|25453205	Peroxidase 12	*Arabidopsis thaliana*	12.5	3	1.700	↑
83	gi|225447324	Peroxidase 27	*Vitis vinifera*	20.2	1	0.674	↓
84	gi|220967704	Monodehydroascorbate reductase	*Solanum lycopersicum*	14.2	3	0.667	↓
85	gi|195973264	Glutathione S-transferase	*Gossypium hirsutum*	18.7	3	1.648	↑
86	gi|354620267	pCPR10-16	*Gossypium barbadense*	49.7	1	1.684	↑
87	gi|15811629	Ribonuclease-like PR-10	*Gossypium arboreum*	30.8	2	1.797	↑
88	gi|33338347	Osmotin-like pathogenesis-related protein	*Gossypium hirsutum*	15.3	3	1.546	↑
89	gi|255537367	Osmotin precursor	*Ricinus communis*	8.5	1	1.780	↑
90	gi|383932370	Nodulin-like protein	*Gossypium hirsutum*	27.4	2	0.612	↓
91	gi|38258655	Monocopper oxidase-like protein SKU5	*Arabidopsis thaliana*	10.5	4	1.444	↑
92	gi|354620271	MLP	*Gossypium barbadense*	19.1	1	0.625	↓
93	gi|194321204	Laccase	*Gossypium hirsutum*	1.3	1	1.680	↑
94	gi|65998365	Dirigent-like protein	*Gossypium barbadense*	12.4	1	1.788	↑
95	gi|66276977	Dirigent-like protein	*Gossypium barbadense*	12.6	1	1.848	↑
96	gi|118926	Desiccation-related protein PCC13-62	*Craterostigma plantagineum*	20.5	4	2.237	↑
97	gi|359480830	L-ascorbate oxidase-like	*Vitis vinifera*	11.6	4	1.565	↑
98	gi|166203457	Universal stress protein 1	*Gossypium arboreum*	11.0	1	1.947	↑
99	gi|94717590	GDP-mannose 3,5-epimerase 2	*Oryza sativa* subsp. *Japonica*	12.5	3	1.529	↑
100	gi|225455388	Germin-like protein 11-1	*Vitis vinifera*	14.7	1	0.603	↓
101	gi|470122858	Plant cadmium resistance 2-like isoform 1	*Fragaria vesca* subsp. *vesca*	8.3	3	1.919	↑
102	gi|75099392	Subtilisin-like protease	*Arabidopsis thaliana*	4.6	1	1.832	↑
103	gi|68064400	Thaumatin-like protein	*Phaseolus vulgaris*	8.9	2	1.714	↑
104	gi|319433441	Copper binding protein 3	*Gossypium hirsutum*	13.0	1	0.551	↓
105	gi|259016223	Glucan endo-1,3-beta-glucosidase 7	*Arabidopsis thaliana*	5.7	2	3.108	↑
106	gi|255546283	Glucan endo-1,3-beta-glucosidase precursor	*Ricinus communis*	7.5	3	2.234	↑
107	gi|255573702	Glucan endo-1,3-beta-glucosidase precursor	*Ricinus communis*	2.3	1	1.934	↑
**OTHER METABOLISM**							
108	gi|89258498	Short chain alcohol dehydrogenase	*Gossypium hirsutum*	39.1	1	0.664	↓
109	gi|7546402	Chain A, Structures of adenylosuccinate Synthetase from *Triticum aestivum* and *Arabidopsis thaliana*	*Arabidopsis Thaliana*	19.4	6	1.553	↑
110	gi|74273629	Gibberellin 20-oxidase 1	*Gossypium hirsutum*	13.3	3	1.631	↑
111	gi|395406786	Putative inactive methylesterase 20	*Arabidopsis thaliana*	6.4	2	0.656	↓
112	gi|255554698	Homogentisate 1,2-dioxygenase	*Ricinus communis*	9.5	3	0.629	↓
113	gi|3183454	Uncharacterized oxidoreductase ykwC	*Bacillus subtilis*	2.9	1	2.067	↑
**UNKNOWN**							
114	gi|224137260	Predicted protein	*Populus trichocarpa*	23.8	2	2.309	↑
115	gi|297736988	Unnamed protein product	*Vitis vinifera*	19.5	6	1.889	↑
116	gi|224106732	Predicted protein	*Populus trichocarpa*	3.4	2	1.775	↑
117	gi|297736988	Unnamed protein product	*Vitis vinifera*	25.6	8	1.717	↑
118	gi|225458697	Uncharacterized protein	*Vitis vinifera*	12.7	5	1.660	↑
119	gi|147767808	Hypothetical protein VITISV_032830	*Vitis vinifera*	8.0	2	1.596	↑
120	gi|224092318	Predicted protein	*Populus trichocarpa*	12.6	5	1.521	↑
121	gi|147820236	Hypothetical protein VITISV_010210	*Vitis vinifera*	12.6	8	1.494	↑
122	gi|217073300	Unknown	*Medicago truncatula*	14.7	3	0.667	↓
123	gi|359496362	Uncharacterized protein LOC100854560	*Vitis vinifera*	5.6	1	0.664	↓
124	gi|388499178	Unknown	*Lotus japonicus*	13.2	2	0.645	↓
125	gi|223943077	Unknown	*Zea mays*	17.8	5	0.644	↓
126	gi|388500070	Unknown known	*Lotus japonicus*	29.4	2	0.582	↓
127	gi|84453208	Putative cytosolic factor	*Trifolium pratense*	13.5	3	0.547	↓
128	gi|147782603	Hypothetical protein VITISV_010455	*Vitis vinifera*	19.4	3	0.447	↓

a Mean ratio corresponds to the protein reporter ion intensity originating from salt-treated protein samples (113 and 114) relative to fully control protein samples (115 and 116) with a 1.5 fold-changes and a p < 0.05.

b Proteins increased in abundance (↑) or decreased in abundance (↓).

### Analysis of differentially expressed enzymes

**Figure 4:**
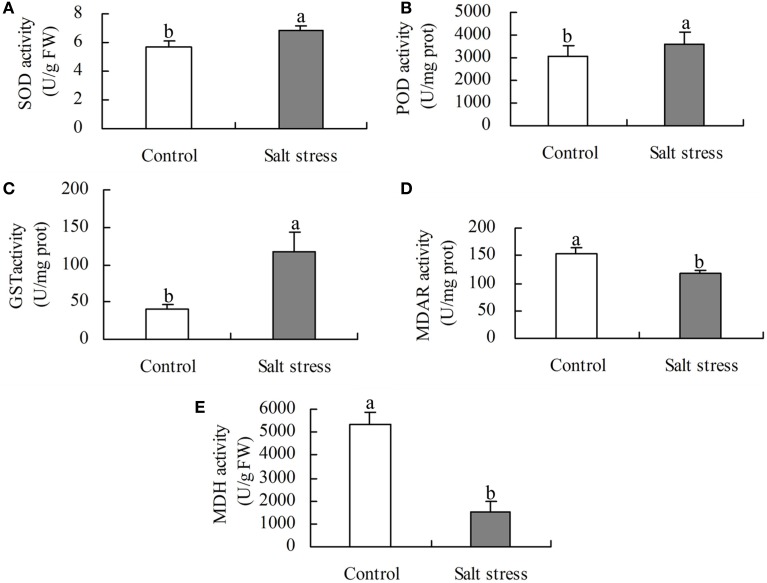
**Activity of (A) superoxide dismutase (SOD), (B) peroxides (POD), (C) glutathione S-transferase (GST), (D) monodehydroascorbate reductase (MDAR), and (E) malate dehydrogenate (MDH) in salt stress and control roots**. Bars represented means ± SE (*n* = 3), Different letters above the bar indicate a significant difference at *P* < 0.05.

Under the same conditions, the level of activity is positively correlated with the enzyme protein abundance (Yang et al., 2013). To validate the DEPs, five enzymes involved in ROS scavenging and organic acid metabolism were selected for activity analysis. The activities of SOD, POD, and GST were higher in NaCl-treated roots than in the control, whereas the activities of MDH and MDAR were lower (Figure 4). These results agree with the protein profiles of the iTRAQ analysis.

### Transcriptional analysis of genes for some DEPs

**Figure 5:**
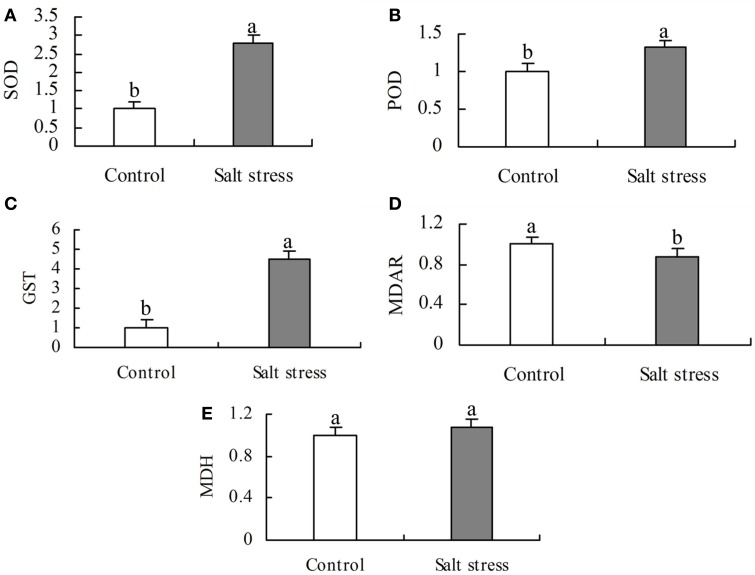
**Relative abundances of (A) superoxide dismutase (SOD), (B) peroxides (POD), (C) glutathione S-transferase (GST), (D) monodehydroascorbate reductase (MDAR), and (E) malate dehydrogenate (MDH) in salt stress and control roots revealed by qRT-PCR**. Bars represent mean ± SE (*n* = 3). Different letters above the bars indicate a significant difference at *P* < 0.05.

In order to assess the correlation of expression levels between mRNA and protein, qRT-PCR was applied to five DEP genes (POD, SOD, GST, MDAR and MDH) as shown in Figure 5. The expression of the former four genes (POD, SOD, GST and MDAR) is consistent with the corresponding DEPs, indicating that the expression of these proteins is regulated at the transcriptional level, but this was not the case for MDH (Table 1).

## Discussion

### Carbohydrate and energy metabolism

Salt stress alters the abundance of many proteins involved in carbon and energy metabolism, including glycolysis, the tricarboxylic acid cycle (TCA), and the pentose phosphate pathway (PPP) in cotton roots. It was found that an FBP3 protein (gi|75268018) was increased. FBP aldolase, a key enzyme in the glycolytic pathways, plays an important role in the production of water-soluble carbohydrates, triose phosphates metabolism and signal transduction (Schaeffer et al., 1997). It was reported that overexpression of FBP could enhance salt tolerance in tobacco by increasing proline content (Konishi et al., 2005). In the present study, up-regulation of FBP3 aldolase increased levels of sugars and starch, and may improve the growth of cotton roots under stress. In our experiment, an enolase protein, which catalyzes the formation of a high-energy phosphoenol pyruvate from 2-phosphoglycerate in the glycolytic pathway, displayed a decrease in abundance after 24 h of salt stress treatment. This result disagreed with the expression profile of this same protein in wheat (Guo et al., 2012a) and cucumber (Du et al., 2010) under salt stress.

Three proteins related to TCA were identified. Pyruvate dehydrogenase E2 (gi|117940179) is an enzyme component of the multienzyme pyruvate dehydrogenase complex and is involved in the formation of cellular energy during the TCA cycle. In this study, the abundance of this enzyme increased under salt stress. However, MDH and ATP-citrate synthase beta chain protein 2 decreased. This suggests that the TCA cycle was inhibited in cotton roots after 24 h of salt stress treatment.

Some ROS-scavenging systems need the PPP pathway that produces NADPH under stress conditions. Phosphogluconate dehydrogenase (PGD, gi|339265919)—the key regulatory enzyme of the PPP pathway—was enhanced under salt stress conditions. Manaa et al. (2011) reported that the PGD activity increased under salt conditions in tomato roots.

The abundance of ATP synthase delta subunit 2 (gi|242129048) decreased under stress. This result supports the previous data on the expression profile of this protein in the roots of *Arabidopsis* (Jiang et al., 2007), rice (Chitteti and Peng, 2007), and cucumber (Du et al., 2010) under salt stress.

Thus, the flexibility of carbohydrate and energy metabolism may help cotton survive under salt stress conditions.

### Proteins involved in transcription

Transcriptional regulation of salt-responsive genes is a crucial part of the plant response to various abiotic and biotic stresses (Jiang et al., 2007). Previous studies showed that chromatin-mediated regulation of gene expression plays an important role in the response to abiotic stress and that histone H1 is involved in stress-induced reactions (Kim et al., 2010). In our data, the expression of histone H1 in salt-treated samples was nearly twice as high as that in the control samples, indicating its role in the salt stress response. Moreover, a zinc-finger transcription factor (gi|75321585) showed higher abundance in roots under salt stress conditions. Zinc finger proteins are well characterized in the regulation of stress responses (Chinnusamy et al., 2006), and the overexpression of CCCH-type zinc finger proteins AtSZF1 and AtSZF2 enhanced salt tolerance in *Arabidopsis* (Sun et al., 2007).

### Protein metabolism

Protein turnover, the balance between synthesis and degradation, is one of the many forms of regulation that is employed to achieve a unified cellular response (Reinbothe et al., 2010). Several proteins, involved in protein translation, processing and degradation, were identified in these iTRAQ data. The abundance of two ribosomal proteins (gi|75266342 and gi|17865566) decreased, whereas two (gi|7526634 and gi|17865566) increased in the present study. Rodriguez-Uribe et al. (2011) has also reported that levels of some of the ribosomal proteins decreased while some specific ribosomal components increased under salt stress. Moreover, our data showed lower expression of a eukaryotic translation initiation factor 3 subunit I (eIF3I, gi|23503072) under salinity, which is consistent with a previous report on *Arabidopsis* (Jiang et al., 2007). In addition, elongation factor gi|6015064 displayed higher abundance under salt stress conditions. The differential regulation of different components of the translation machinery indicates that complicated regulation mechanisms may govern protein synthesis in order to help plants cope with salt stress.

Proper protein folding and processing is important for normal cellular function under salt stress. Here, it was found that the expression of T-complex protein 1 (TCP1, gi|117949833) and protein disulfide isomerase-like 1-6 (PDIL1-6, gi|75115360) were enhanced. TCP1 is involved in proper folding and assembly of proteins to cope with salinity in wheat roots (Wang et al., 2008). PDIs are molecular chaperones that aid the formation of proper disulfide bonds during protein folding (Houston et al., 2005). Two isoforms of PDIs increased in rice roots under salt stress (Nohzadeh Malakshah et al., 2007). Hsp70 s assists in proper folding of newly synthesized polypeptides and import/translocation of precursor proteins. Two hsp70 members (gi|211906494, gi|211906504) showed lower abundance in NaCl-treated roots. This result is consistent with the expression profile of this protein in *Arabidopsis* (Jiang et al., 2007). The ubiquitin/26S proteasome pathway selectively degrade key regulatory proteins and enzymes under salt stress conditions (Vierstra, 2003). The abundance of some components of ubiquitin/26S proteasome system including ubiquitin-activating enzyme (gi|95116512) and polyubiquitin protein (gi|18803) decreased under salt stress conditions. These findings suggest that decreased protein degradation compensates for decreased protein biosynthesis in roots under salt stress.

### Cell wall and cytoskeleton metabolism

The cytoskeleton is rapidly remodeled to allow cell size adjustment in order to maintain normal cell turgor pressure under salt stress conditions (Zhang et al., 2012). In salt-treated roots, it was found that three actin-binding proteins (ABPs), including actin depolymerizing factor (ADF), actin-related protein 2 (ARP2), and actin-binding protein 29 (ABP29), can bind to actin cytoskeletons and effect remodeling. In a previous study, ABP29 from *Lilium* pollen played an important role in the remodeling of the actin cytoskeleton during pollen germination and pollen tube growth (Xiang et al., 2007). Thus, depolymerization and subsequent reorganization of the actin cytoskeleton enhanced salt tolerance in cotton roots.

Some proteins, including glycine-rich proteins (GRPs), proline-rich protein (PRPs), and arabinogalactan proteins (AGPs), are essential structural protein components of the cell walls of many higher plants. We found that a GRP (gi|255547195) and a hybrid PRP (HyPRP gi|224552010) displayed higher abundance in roots under salt stress. Biosynthesis of GRPs and their accumulation in vascular tissues are part of the plant's defense mechanism (Mousavi and Hotta, 2005). Overexpression of *HyPRP* (encoding a HyPRP) in *Arabidopsis* enhanced germination under cold and high salinity stress conditions (Qin et al., 2013). A sub-group of AGPs that include one or two AGP domains and one or two copies of the fasciclin domain are termed fasciclin-like arabinogalactan protein (FLAs). FLAs, which are located in the cell wall/plasma membrane and cell surface, have many developmental roles. Some FLAs are involved in microspore and lateral root/shoot development, maintaining proper cell expansion and/or keeping the integrity and elasticity of cell wall matrix in *Arabidopsis* (Johnson et al., 2003). In this present study, five FLAs (gi|157273666, gi|157273646, gi|157273640, gi|157273638, gi|157273660) displayed increased in abundance, but two (gi|157273642, gi|150416583) decreased. The diverse expression of FLAs suggests that these proteins may be involved in a wide range of biological process under salt stress conditions.

### Membrane and transport

Under salinity conditions, Na^+^/K^+^ ratios and Na^+^ concentration increase in plant roots causing hyperosmotic stress, ion imbalance and toxicity (Zhao et al., 2013). H^+^-ATPase plays an essential role in the maintenance of ion homeostasis in plant cells. The plasma membrane H+-ATPase in tomato (Kerkeb et al., 2001) and the vacuolar H^+^-ATPase in the roots of *Arabidopsis* (Jiang et al., 2007), rice (Cheng et al., 2009), wheat (Guo et al., 2012a), tomato (Manaa et al., 2011), and cucumber (Du et al., 2010) are induced under salt stress conditions. Here, increased abundance of one plasma membrane H^+^-ATPase (gi|7105717) and two vacuolar H^+^-ATPases (gi|1336803, gi|2493146) indicates that the increased activities of these enzymes are considered to be a cost-effective strategy for osmotic adjustment, which reduces the Na^+^ concentration in the cytosol in plants under salt stress conditions.

ABC transporters transport stress-related secondary metabolites such as alkaloids, terpenoids, polyphenols and quinines (Theodoulou, 2000). In *Arabidopsis*, ABC transporter affected Na^+^/K^+^ homeostasis and elicited a salt stress response (Lee et al., 2004). The up-regulation of an ABC transporter (gi|224130846) in cotton roots suggests that it may play an important role in salt-stressed responses.

Aquaporins (AQPs)—channel proteins that facilitate the transport of water and/or small neutral solutes or gasses in the plasma and intracellular cell membranes—are associated with plant stress tolerance (Wang et al., 2011). PIPs and TIPs, two subfamilies of AQP, are most abundant in the plasma membrane and vacuolar membrane, respectively (Danielson and Johanson, 2008). HvPIP2:1 was down-regulated in barley seedlings, and its overexpression enhanced salt sensitivity in transgenic rice under salt stress conditions (Katsuhara et al., 2003). Overexpression of the *Panax ginseng* TIP2:1 gene in *Arabidopsis* enhances tolerance to salt stress, but overexpression of *GsTIP*2:1 depresses salt tolerance and dehydration stress (Wang et al., 2011). Thus, the regulation mechanism of AQPs under salt stress conditions is complicated and requires further study (Peng et al., 2007). Here, four PIPs (gi|300793598, gi|256568429, gi|118132686, gi|118132686) and five TIPs (gi|29265353, gi|292653547, gi|461929, gi|300793602, gi|292653535) showed lower abundance in response to salt stress. This may be attributed to the reduced hydraulic conductivity of membranes to prevent water loss under salt stress conditions (Sutka et al., 2011).

### Signal transduction

LRR-RLKs function in a wide variety of signal transduction pathways related to hormone and abiotic stress responses (Hove et al., 2011). Potato LRPK1 functions under diverse stress conditions, such as wounding, and high-, low-temperature, and salinity stress (Wu et al., 2009). In our present data, the up-regulation of LRR-RLKs (gi|363807628) imply it has a role in Na^+^ and plant interactions, specific recognition, and signal transduction leading to an induced salt-stressed tolerance. Nucleoside diphosphate kinase B (NDPKB, gi|1346675) is an enzyme that converts GTP to ATP, and is involved in the H_2_O_2_ mediated mitogen-activated protein kinase signaling pathway. NDPK increased tolerance in response to NaCl in *Arabidopsis*, creeping bentgrass and rice (Jiang et al., 2007; Seong et al., 2007; Xu et al., 2010).

### Stress and defense

Salt stress causes the production of excessive reactive oxygen species (ROS), which oxidize cellular components and irreversibly damage plant cells (Askim et al., 2014). ROS can be scavenged in plants by SOD, POD and GSTs. Ten of these proteins were identified in this study (Table 1). In most cases, higher expression of these proteins was found in salt-treated samples than in the control. Increased accumulation of SOD was noted in the roots of *Arabidopsis* (Jiang et al., 2007), wheat (Guo et al., 2012a), cucumber (Du et al., 2010), and salt cress (Zhou et al., 2010) under salt stress conditions. The up-regulation in abundance of Cu/ZnSOD (gi|74229677) also indicates that it helps cope with salt stress in cotton. PODs catalyze the reduction of H_2_O_2_ using electron donors such as lignin precursors, phenolic compounds, auxins and secondary metabolites (Zhao et al., 2013). In the present study, the levels of seven POD isozymes (gi|357470271, gi|115345276, gi|73913500, gi|255551599, gi|32351452, gi|255581003, gi|25453205) increased in response to salt stress but POD 27 (gi|225447324) did not. The levels of POD increased in the salt-stressed roots of wheat (Peng et al., 2009), barley (Witzel et al., 2009), cucumber (Du et al., 2010), and rice (Cheng et al., 2009) but decreased in creeping bentgrass (Xu et al., 2010). GST increased in the salt-stressed roots of *Arabidopsis* (Jiang et al., 2007), rice (Chitteti and Peng, 2007), barley (Witzel et al., 2009), and wheat (Peng et al., 2009). Here, higher levels of a GST (gi|195973264) were also observed in salt-stressed cotton roots. GSTs may play a pivotal role in preventing the degradation of organic hydroperoxides to cytotoxic aldehyde derivatives under salt stress conditions in cotton. Thus, it is demonstrated that antioxidant enzymes protect salt-stressed cotton roots from oxidative damage.

MDAR catalyzes the reduction of monodehydroascorbate to ascorbate (ASA) and is essential in order to maintain a reduced pool of ascorbate. Germin-like proteins (GLP) possess both oxalate activity and SOD activity. Here, the decreased expression of MDAR (gi|220967704) and GLP (gi|225455388) was identified in salt- stressed cotton roots. It is suggested that although plants require MDAR and GLP in order to eliminate ROS, the fine tuning of the levels of various antioxidants is also an important consideration in stress responses (Lisenbee et al., 2005).

In addition to the redox related proteins, plants have developed cross-tolerance mechanisms to be able to cope with different stresses (Zhang et al., 2012). Some biotic and abiotic stress-responsive proteins play important roles in salt tolerance (Table 1). Some biotic stress-related proteins were induced under salt stress conditions, such as pCPR10-16 (gi|354620267), ribonuclease-like PR-10 (gi|15811629), osmotin-like pathogenesis-related proteins (gi|3333834), thaumatin-like protein (TLP, gi|68064400), USP (gi|166203457), and glucan endo-1,3-beta-glucosidases (gi|255546283, gi|259016223, gi|255573702). PR10 mediates tolerance to heavy metals (Wang et al., 2014) and pathogen attack (Coumans et al., 2009). TLP, a subgroup of pathogenesis-related proteins, is induced by phytohormones (SA, JA, and ABA) and stress stimuli (wounding, cold temperature and high salinity) (Wang et al., 2010). Overexpression of the *GbTLP*1 in tobacco enhances resistance to *Verticillium dahliae*, salinity and drought (Munis et al., 2009). USP helps cotton plants adapt to water stress (Maqbool et al., 2009). Glucan endo-1, 3-beta-glucosidase accumulates in rice in response to ABA and salt stress (Li et al., 2010). Moreover, some abiotic stress-related proteins, e.g. DIR (gi|65998365, gi|66276977), desiccation-related protein PCC13-62 (gi|118926) also respond to salt stress (Bartels et al., 1990; Guo et al., 2012b). DIR is involved in the response to drought, salts and oxidation (Guo et al., 2012b). Desiccation-related protein PCC13-62 promotes the plant's tolerance to extreme desiccation (Bartels et al., 1990). These proteins provide novel insights into the understanding of the cross-tolerance mechanisms in roots in response to biotic and abiotic stress.

### The correlation of protein abundance and gene expression

There might be a weak correlation between the transcript levels of genes and their protein abundance (Yang et al., 2013). The discrepancy between protein and mRNA expression may be caused by the various levels of regulation, e.g., post-transcriptional, translational or post-translational regulation (Tian et al., 2004). A discrepancy between transcript levels of MDH and the abundance of the corresponding proteins (Figure 5E, Table 1) highlights the effect of post-transcriptional modifications.

## Conclusion

An iTRAQ-based proteomic technique was employed to compare the abundance of proteins in untreated and salt-treated roots for 24 h. One hundred and twenty-eight DEPs were identified, 76 of which displayed increased abundance and 52 decreased under salt stress conditions. These DEPs are mainly involved in the biological processes of carbohydrate and energy metabolism, transcription, protein metabolism, cell wall and cytoskeleton metabolism, membrane and transport, signal transduction and stress and defense. The diverse array of proteins affected by salt stress conditions indicates that there is a remarkable flexibility in cotton root metabolism, which may contribute to its survival in salinity conditions. High positive correlation between the abundance of some altered proteins (SOD, POD, GST, MDAR, and MDH) and their enzyme activity demonstrates that the iTRAQ-based proteomic technique is sufficiently reliable for the identification and quantification of a large number of cotton root proteins. qRT-PCR results suggest that the expression of some proteins (e.g., MDH) can be regulated by post-transcriptional modifications. With this technology, many new salt-responsive proteins, such as ARP2, FLAs, TIPs, PIPs, LRR-RLKs, TLP, USP, DIR and the desiccation-related protein PCC13-62 were identified from cotton roots. These novel proteins provide a good starting point for further research into their functions using genetic or other approaches. These findings significantly improve the understanding of the molecular mechanisms involved in the tolerance of plants to salt stress.

### Conflict of interest statement

The authors declare that the research was conducted in the absence of any commercial or financial relationships that could be construed as a potential conflict of interest.

## Abbreviations

ABPs: actin-binding proteins
ADF: actin depolymerizing factor
ARP2: actin-related protein 2
AGPs: arabinogalactan proteins
AQPs: Aquaporins
DEPs: differentially expressed proteins
DIR: dirigent-like protein
FBP3: fructose-bisphosphate aldolase 3
FLAs: fasciclin-like arabinogalactan proteins
GST: glutathione S-transferase
GRPs: glycine-rich proteins
iTRAQ: isobaric tag for relative and absolute quantitation
LRR-RLKs: leucine-rich repeat receptor-like kinase-encoding genes
MDAR: monodehydroascorbate reductase
MDH: malate dehydrogenase
NDPK: nucleoside diphosphate kinase
PPP: pentose phosphate pathway
PGD: phosphogluconate dehydrogenase
POD: peroxidase
PDIL1-6: protein disulfide isomerase-like 1-6
PRPs: proline-rich proteins
PIPs: plasma membrane intrinsic proteins
SOD: superoxide dismutase
TCA: tricarboxylic acid cycle
TCP1: T-complex protein 1
TIPs: tonoplast intrinsic proteins
TLP: thaumatin-like protein
USP: universal stress protein.
