# Supplementary material for: Identification of early salt stress responsive proteins in seedling roots of upland cotton (Gossypium hirsutum L.) employing iTRAQ-based proteomic technique
Source: Front Plant Sci. 2015 Sep 11;6:732. doi: 10.3389/fpls.2015.00732 (PMC4566050; doi:10.3389/fpls.2015.00732)
Supplement: Supplementary file 4 [file Image1.PDF]

1 Garb_14205 gi|377824753 pectin methylesterase

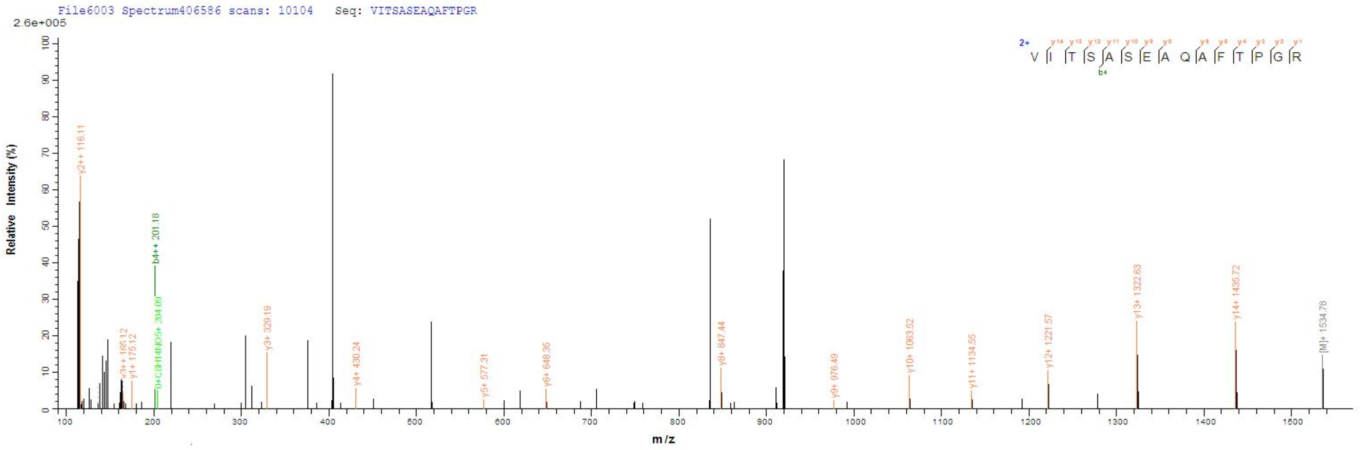

MFIGDGRTKTIITGSRNVVDGSTTFHSATVAAVGEKFLARDITFQNTAGPSKHQAVALRVGSDLSAFYNCDMLAYQDTLYVHSNRQFYVNCLVAGTVDFIFGNAAAVFQNCDIHARKPNSGQKNMVTAQGRTDPNQNTGIVIQKCRIGATSDLQPVRKNFPTYLGRPWKEYSRTVVMQSTISDVIQPAGWHEWSGSFALKTLFYAEYQNTGAGASTSARVKWGGYKVITSASEAQAFTPGRFIAGGSWLSSTGFPFALGFFLAYQFSVLFQWDFVRLIKTSMDSIKSFKGYGKVDEAEERAFKRKTRRRLIILVISIIVLLAIAAKILGLLTNFRIPIHRRLLGFQKAAPSEFPAWVSPTERRLLQESKPTPNVIVAKDGSGHFRTINEAVQLVGKKNQSRFVIYVKEGKYVENVNLDKHRWNVMIYGDGKTKTIISGSRNFVDGTATFDTATFTVAGRGFIAKDIKFENTAGAAKHQAVAMRSGSDRSVFYSCAFDAYQDTLYAHSNRQFYRECDILGTIDFIFGNAAVVFQNCNIQPRQPLPNQFNTITAQGKKDPNQNTGISIQKCTITPFGNLTANTYLGRPWKEFSTTVIMQSNIGAFLNPVGWREWVTNVDPPSTILYAEYQNTGPG2

2 Garb_13664 gi|211906490 malate dehydrogenase

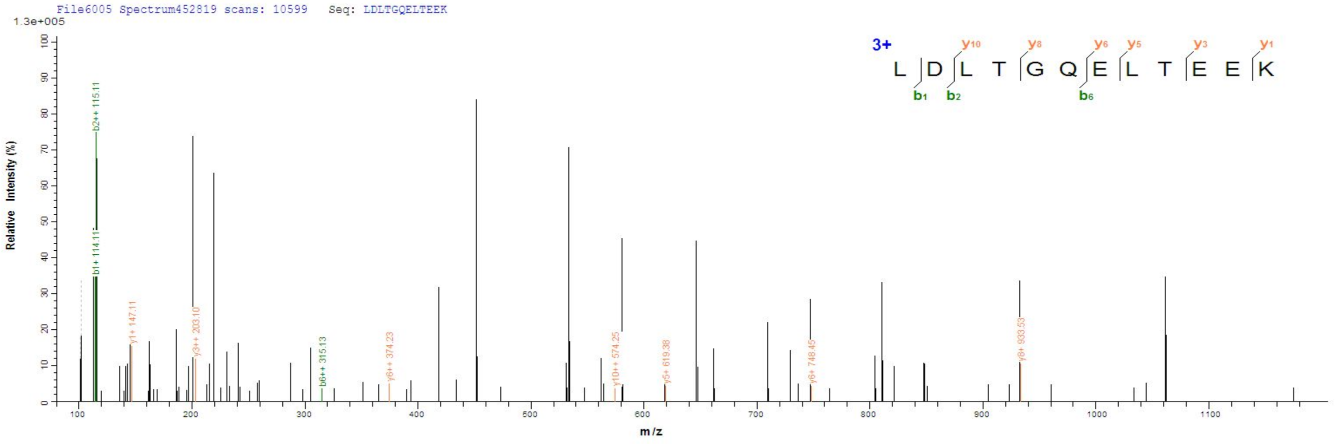

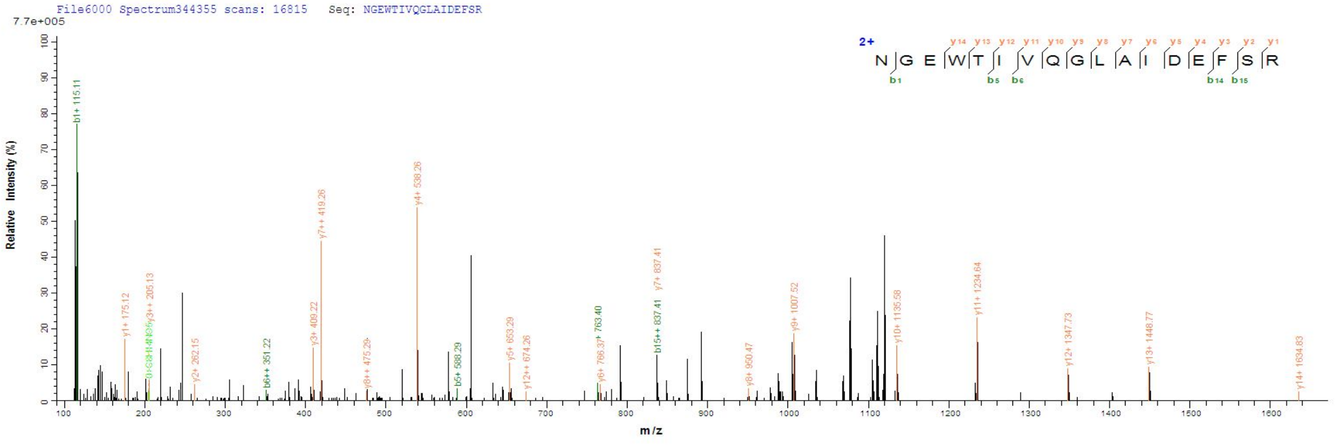

MAKEPVRVLVTGAAGQIGYALVPMIARGVMLGADQPVILHMLDIPPAAETLNGVKMELVDAAFPLLKGVVATTDVVE ACTGVNVAVMVGGFPRKEGMERKDVMSKNVSIYKSQASALEQHAAPNCKVLVVANPANTNALILKEFAPSIPAKNITC LTRLDHNRALGQISEKLNVQVSDVKNVIIWGNHSSSQYPDVNHATVMTPSGEKPVRELVKDDAWLNGEFITTVQQRG AAIIKARKLSSALSAASAACDHIRDWVLGTPEGTWVSMGVYSDGSYNAPAGVIYSFPVTCKNGEWTIVQGLAIDEFSR KKLDLTGQELTEEKELAYSCLS

3 cotton_GLEAN_10015770 gi|122216326 Perakine reductase

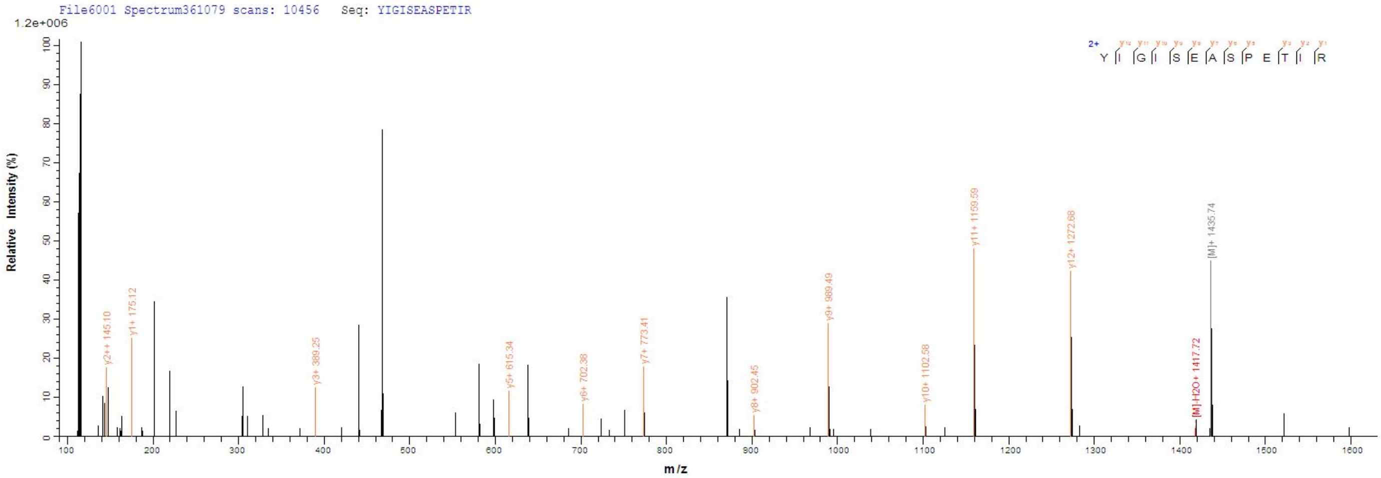

MAIQIPRVKLGTQGFEVSKLGFGCMGLSGHNNSVSDEVGIAIIKHAFERGITHFDTADMYGPKTNEILVGKALKHLPRE KVQLATKFGVESMGPGGPVINGTPEFVRSSLEASLQRLDVDYIDLYYIIRVDHKTPIEDTMEELKKLVEEGKIKYIGISEA SPETIRRAHAVHPLTAVQLEWSLWTRDVEEEIIPLCRELGIGIVPYSPLGRGFFAGRANKDTSNTPLRMFPRFSGENLEKN RILYSKVEKLAEKHGCTAAQLALAWVLHQGDDVAPIPGTTKIENLDSNIESVKVKLTKEDLKEIIDTIPIHEVAGSNYPDS LKQFTWKYGNTPPKKST

4 Garb_33770 gi|339265919 phosphogluconate dehydrogenase

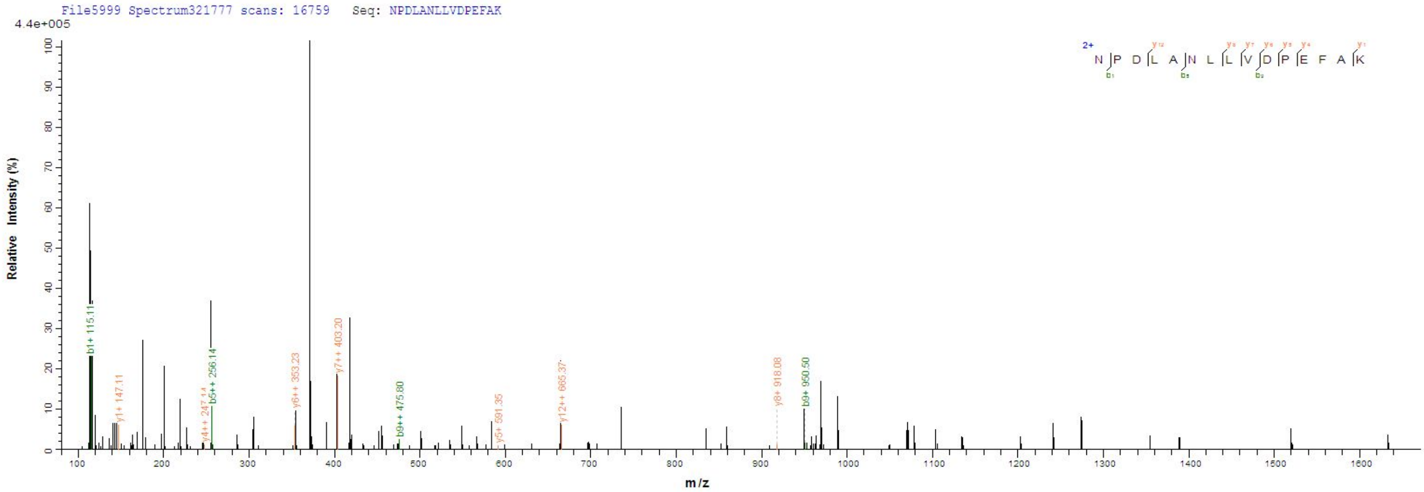

MAPPTRIGLAGLAVMGQNLALNIAEKGFPISVYNRTTSKVDETVERAKQEGDLPLFGFHDPESFVQSIQKPRVIIMLVKA GAPVDQTIKTLSAYMEKGDCIIDGGNEWYENTERREKEMSGLGLLYLGMGVSGGEEGARNGPSLMPGGSFEAYKYIE DILHKVAAQVSDSGPCVTYIGKGGSGNFVKMVHNGIEYGDMQLIAEAYDVLKSVGKLSNEELRSVFLEWNRGELLSF LIEITADIFGIKDDKGEGYLVDKVLDKTGMKGTGKWTVQQAADLSIAAPTIAASLDSRFLSGLKEERVEAAKVFKSGGF GDVLTDQTVDKAKLIDDVRQALYASKICSYAQGMNLIRAKSIEKGWDLKLGELARIWKGGCIIRAIFLDRIKKAYDRNP DLANLLVDPEFAKEIIDRQSAWRRVVCLAINSGISTPGMSASLAYFDTYRRERVPANLVQAQRDYFGAHTYERVDMEGS FHTEWFKTAKQLKN

5 Garb_01661 gi|225455555 enolase

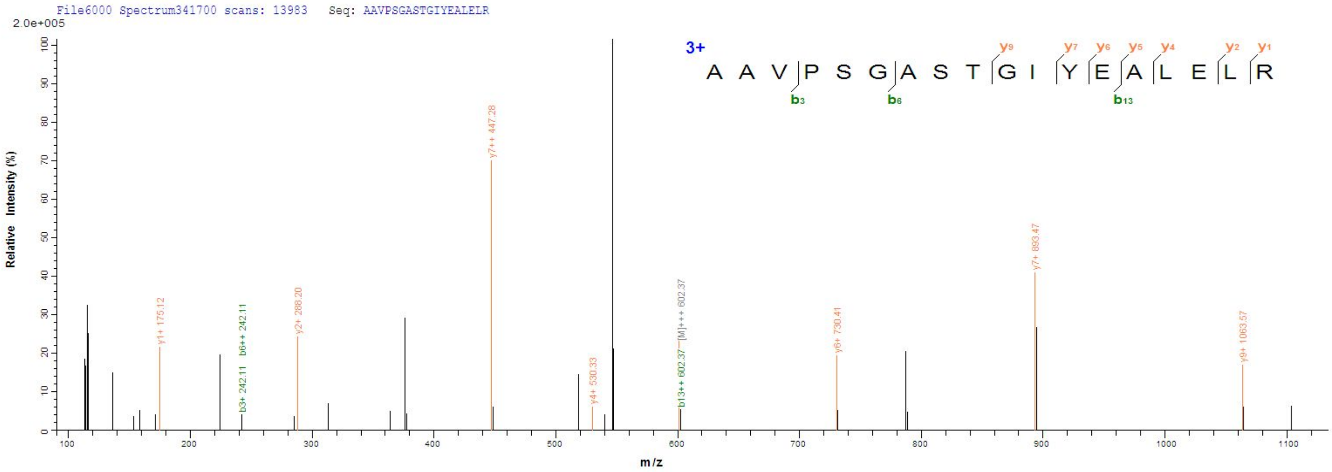

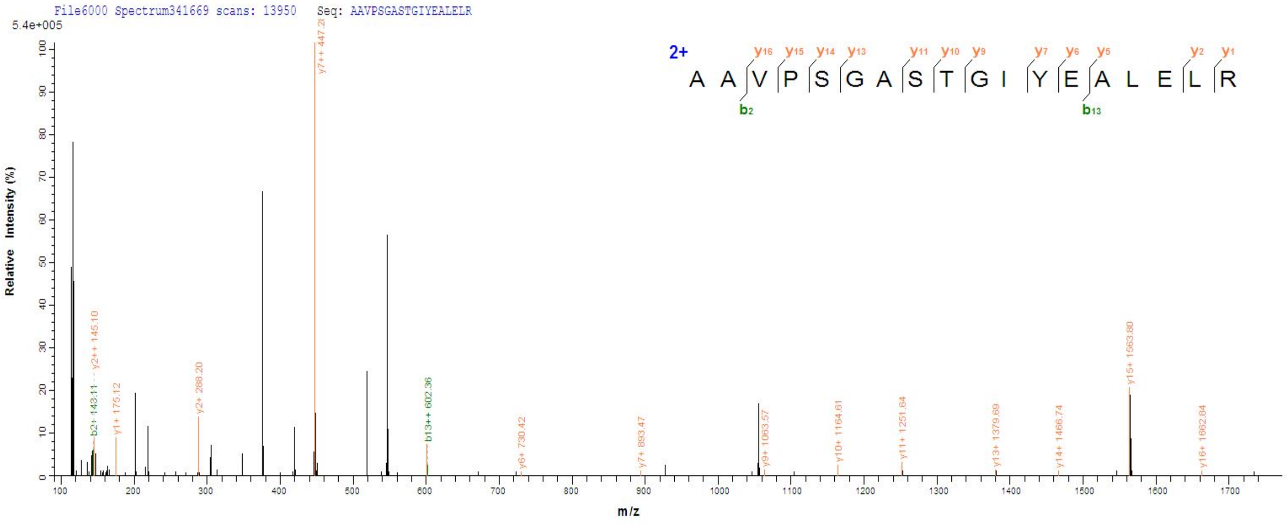

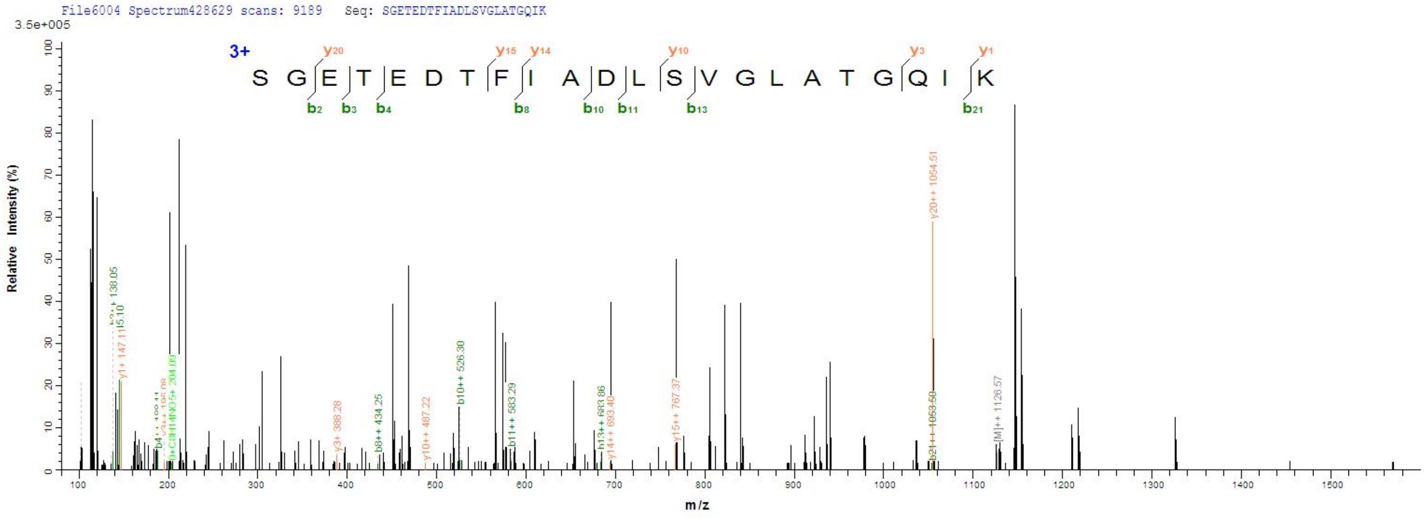

MATIKCVKARQIFDSRGNPTVEVDVSLSDGIVARAAVPSGASTGIYEALELRDGGSDYIGKGVLKAVENVNTIIGPALVG
KDPTEQAKIDNFMVQQLDGTVNEWGWCKQKLGANAILAVSLAVCKAGAMLKKIPLYHIANLAGNKTLVLPVPAFNVI
NGGSHAGNKLAMQEFMILPVGASSFKEAMKMGVEVYHHLKAVIKKKYGQDATNVGDEGGFAPNIQENKEGLELLKT
AISKAGYTGKVVIGMDVAASEFYDNKDKTYDLNFKEENNDGSQKISGDSLKNVYKSFVTDYPIVSIEDPFDQDDWEHY
AKMTSEIGEQVQIVGDDLLVTNPKRVEKAIKEKTCNALLLKVNQIGSVTESIEAVKMSKSAGWGVMASHRSGETEDTF

IADLSVGLATGQIKTGAPCRSERLAKYNQLLRIEEELGAAAVYAGAKIRAPVEPY

6 cotton_GLEAN_10028104 gi|55584187 Quinone oxidoreductase-like protein At1g23740

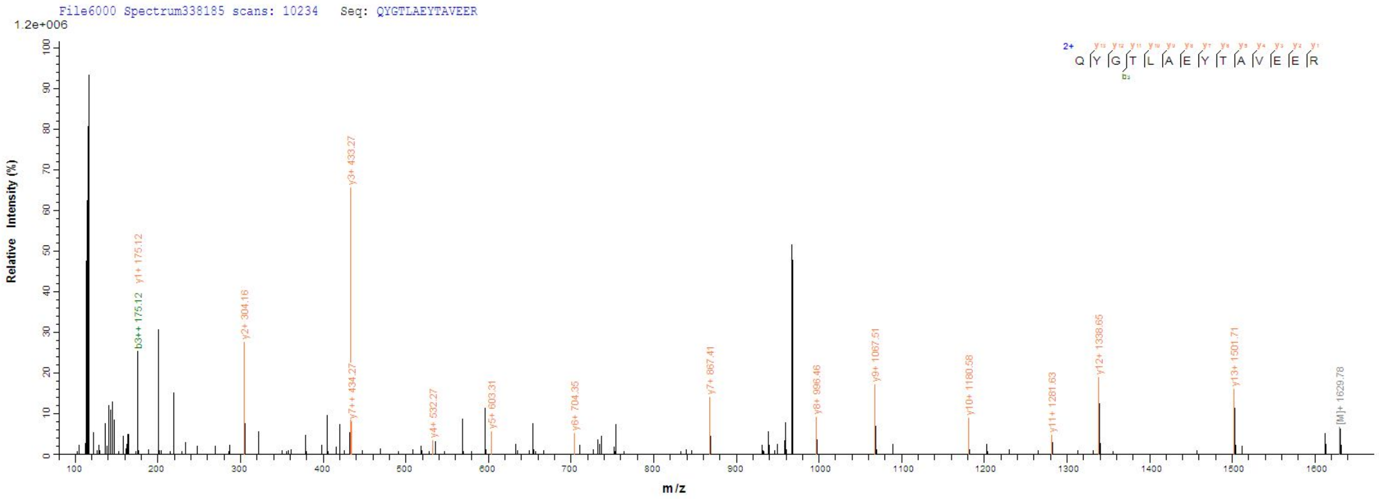

MENVPYTMKAWIYGQHGKPEDVLKLKSDVVVPELKEDQVLVKVMASGLNPVDNKRMLGIFVQAECPFPTVPGYDVAGVVVKVGSQVKNLKVGDEVYGNIHEKALDHPKQYGTLAEYTAVEERLLAPKPKNLSFTEAASLPLAKHVFGASRVVATASTGKLELLRNLGADLAVDYTKENFEDLPEKFDVVYDSVEQCERAVKAMKEGGEVVIVIGAVTVPAFVFIVTSNGADLEKLNPYLESGKVKAVIDPNGIYPFSQTLEGLAYVDTGRVAGKVVIYPIQQDN

7 Garb_02390 gi|117940179 Dihydrolipoyllysine-residue acetyltransferase component 1 of pyruvate dehydrogenase complex, mitochondrial

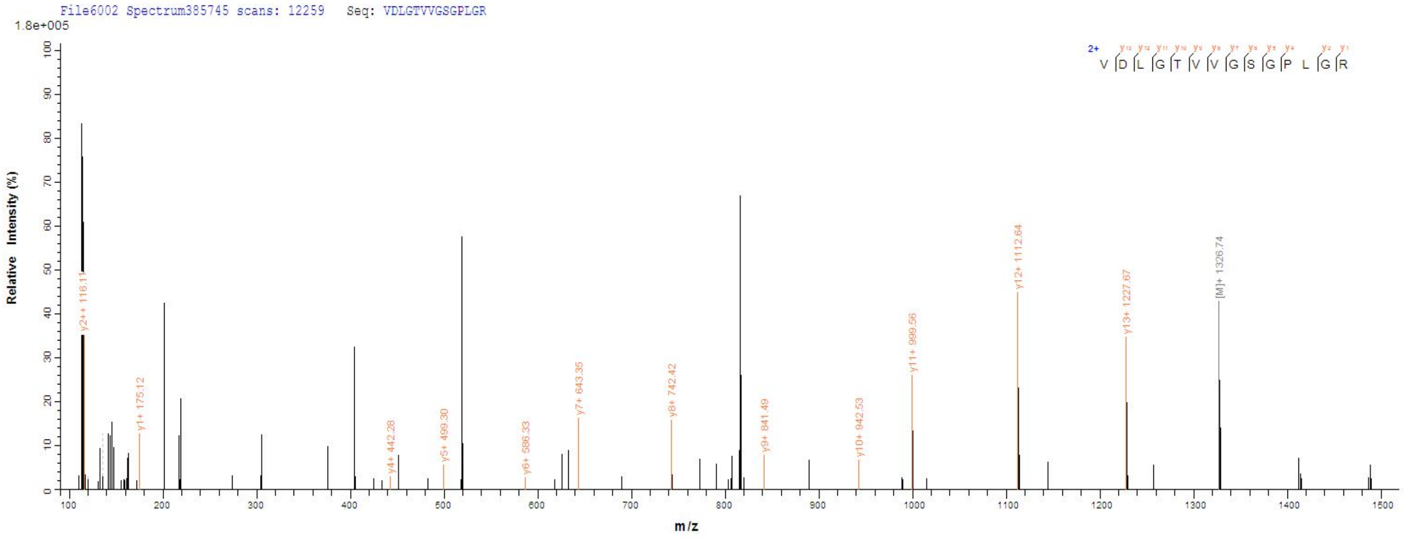

MANVLQTSFLPSTSSLRRPSISGSKGKSQSLQVKAKIREIFMPALSSTMTEGKIVSWMKSEGDKLSKGESVVVVESDKADMDVESFHEGFLAAIMVEEGGVAPVGSAIALLAETEDEIAEAKTKSQSSSNSTPQRIVASPYAKKLAKELKVDLGTVVGSGPLGRIVAKDVEAAAVAVPVAASPAKPDPVAPGIELGTVVPFTTMQGAVSRNMVESLSVPTFRVGYTITTNALDALYKKIKSKGVTMTALLAKATALALVQHPVVNSCCRDGNSFTYNSSINIAVAVAIDGGLITPVLQDADKVDIYTLSRKWKELVDKARAKQLQPHEYNTATNWPRKLLQCKEAKSSLLAKESCKAAKAAIYSTFGSSQSLLPLAQIGVTESSPNIVAATAVLSASPCTFTLSNLGMFGVDRFDAILPPGTGAIMAVGASQPSVVASMDGRIGMKNQMQVNVTADHRVIYGADLAAFLQTLAKIVEDPKDLTF

8 Garb_19216 gi|225465847 NADPH:quinone oxidoreductase

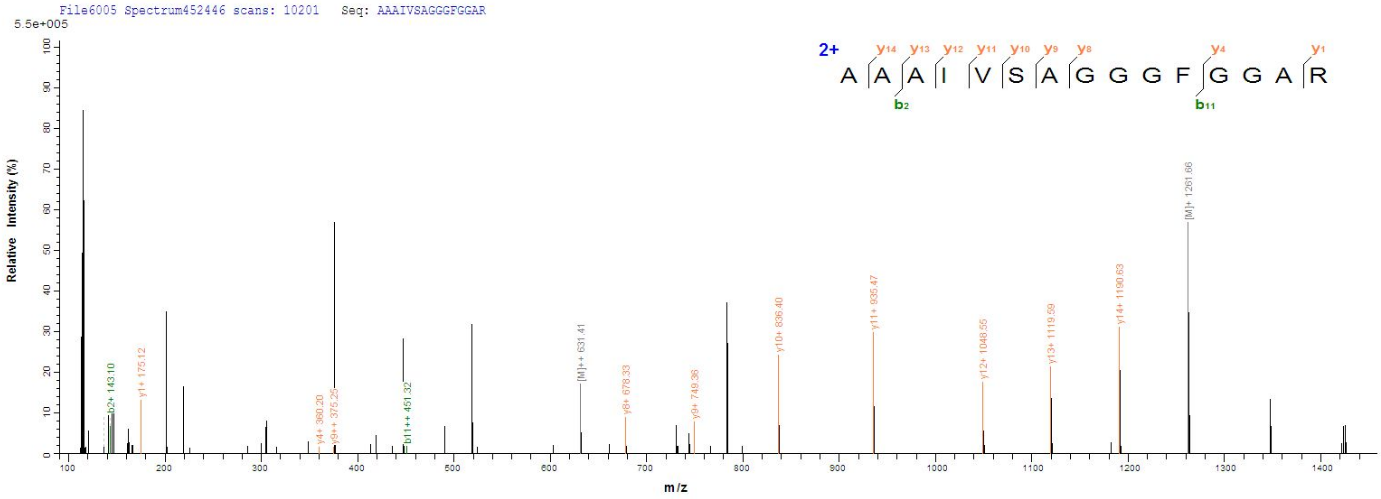

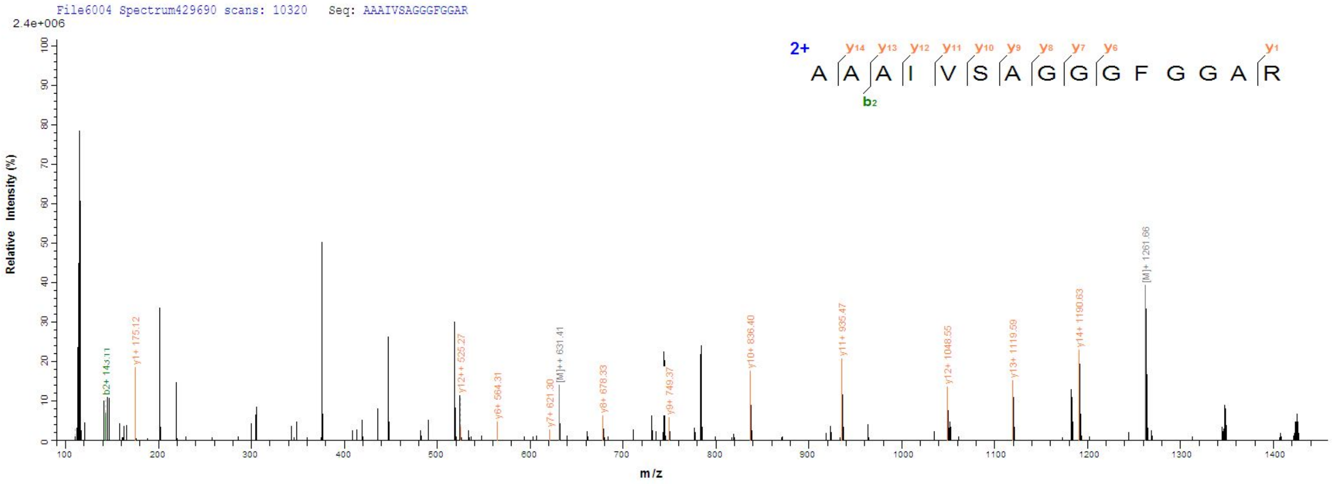

MEAVVASKPVIKVAAICGSLRKASYNRGLIRTALELTKESIAGLQMEYIDISPLPMLDTDLEVDGKFPPAVEAFRQKILEADSILIASPEYNYSLTAPLKNALDWASRPPNVWADKAAAIVSAGGGFGGARSHYHLRQVGVFLDLHFINKPEFYLNAFQPPAKFDSDGNLIDEDSKERMKQVLLSLQAFTLRLQPKN

9、cotton_GLEAN_10006221 gi|75262456 ATP-citrate synthase beta chain protein 2

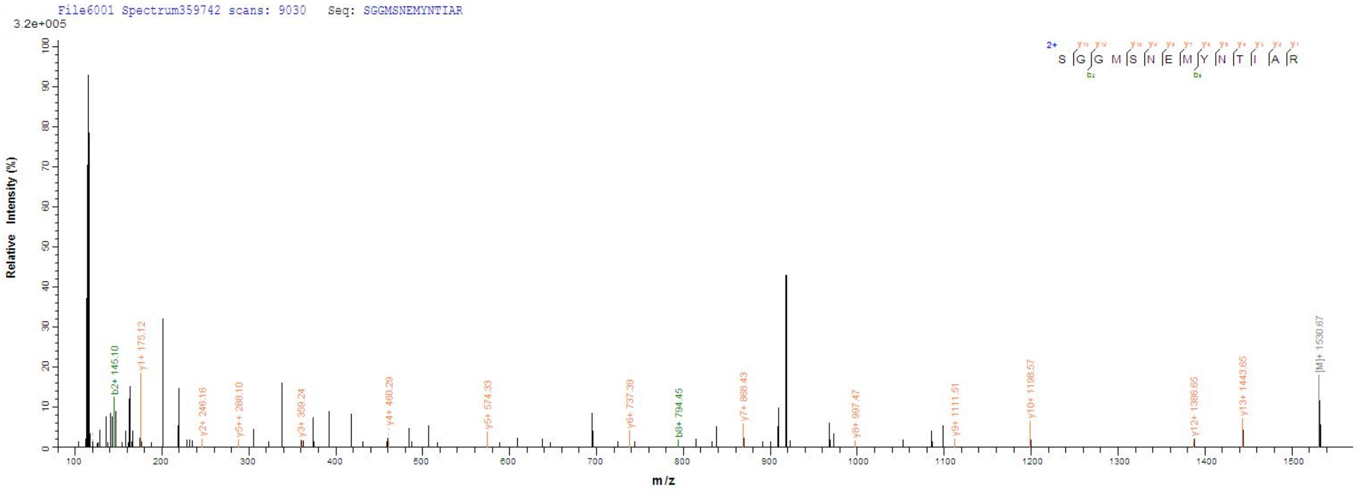

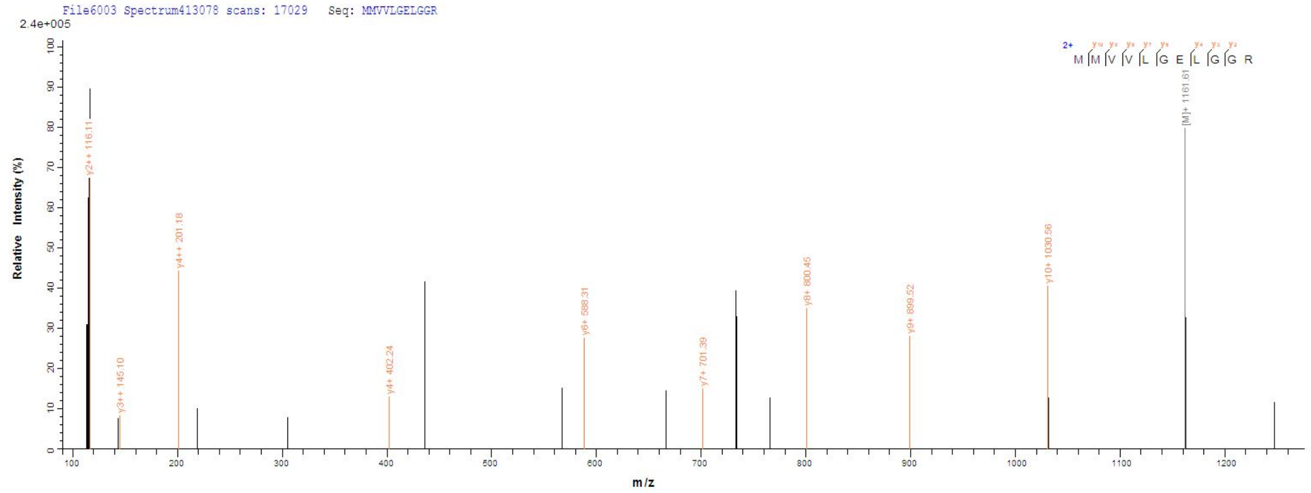

MATGQLFSRTTQALFYNYKQLPIQRMLDFDFLCGRETPSVAGIINPGAEGFQKLFFGQEEIAIPVHSTIEAACAAHPTAD
VFINFASYRSAAASSMSALKQPTIRVVAIIAEGVPESDTKQLIAYARSNNKVVIGPATVGGIQAGAFKIGDTAGTIDNIVH
CKLYRPGSVGFVSKSGGMSNEMYNTIARVTDGIYEGIAIGGDVFPGSTLSDHILRFNNIPQVKMMVVLGELGGRDEYSL
VEALKQGKVNKPVVAWVSGTCARLFKSEVQFGHAGAKSGGEMESAQAKNQALREAGAVVPTSYEALEATIKETFEQL
VEEGKITPVKEVKPPQIPEDLNSAIKSGKVRAPTHIISTISDDRGEEPCYAGVPMSSIVEQGLGVGDVISLLWFKRSLPRY
CTKFIEICIMLCADHGPCVSGAHNTIVTARAGKDLVSSLVSGLLTIGPRFGGAIDDAARYFKDAYDRGLTPYEFVESMKK
KGIRVPGIGHRIKRGDNRDKRVELLQLFARTNFPSVKYMEYAVQVETYTLSKANNLVLNVDGAIGSLFLDLLAGSG

10. Garb_26322 gi|242129048 ATP synthase delta subunit 2

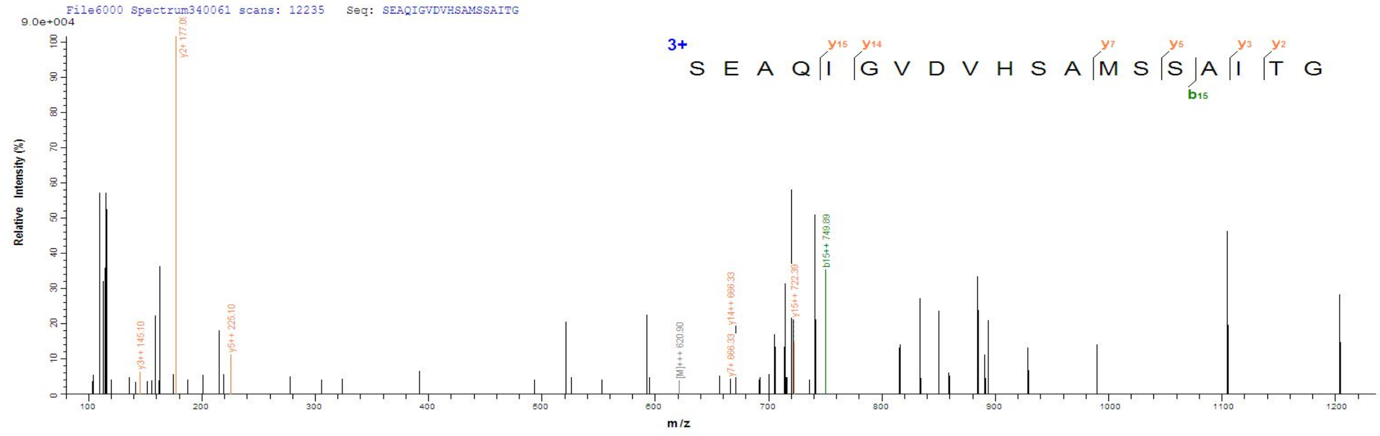

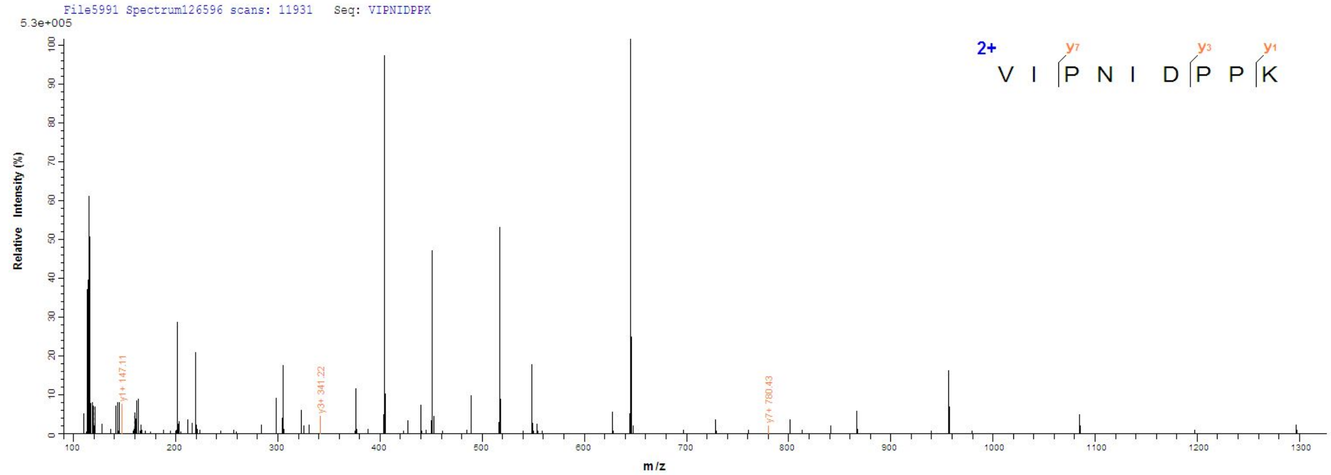

MFRQASRLLARTTTPWRRSRAFSSDLPATPAQDSNFIESWSKVIPNIDPPKTPSSFMTPRPATPSAIPSKLTVNFVLPYASELSAKEVDMVIVPATTGQMGVLPGHVPTIAELKPGILSVHEGNDVTKYFLSSGFALIHANSVADIIAVEAVPVDHLDPALVQKGLADFTQKLGSATTDLEKSEAQIGVDVHSAMSSAITG

11. Garb_06228 gi|91981275 pectin methylesterase, partia

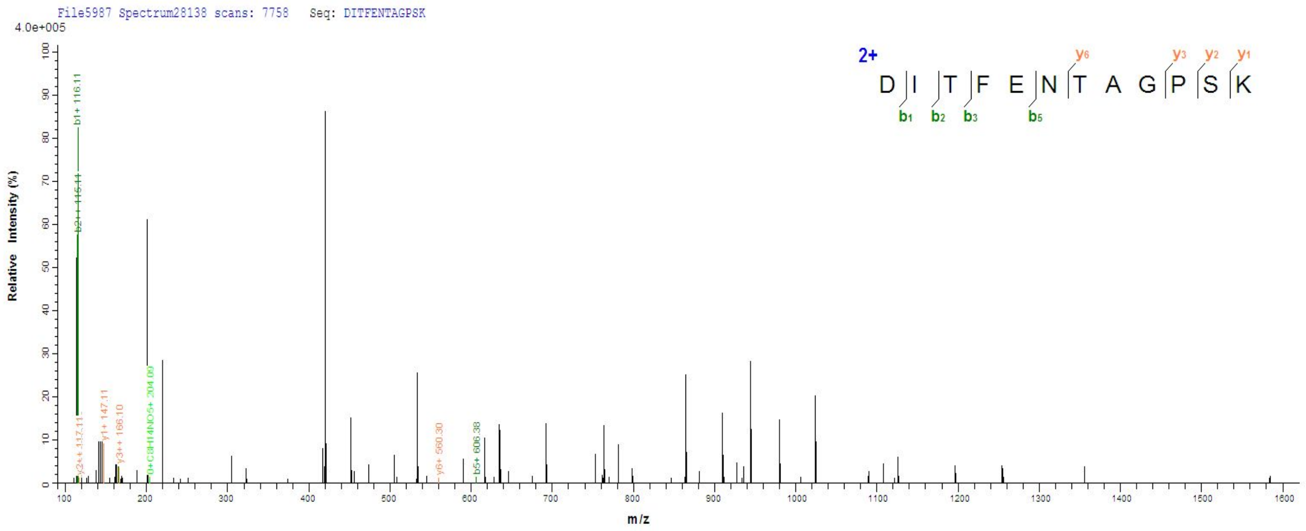

MNESKEILTKIFDSAKHISSTKKHKKIFLAVLACLVIVAALIGIVAKENSRNKFDETNTSHHAIVKSACSSTFYPDLCFSAIAAAAAKNFTGKKDVIRLSLNITASAVEHNYFKINKLLARTGLTEREVTALQDCLETINVTLDELHQAVEDLHGYPYKKPLTQHADDLKTLISAAMTNQETCLDGFSHKNADKEVRKALIDGEKYVEKMCSNALAMIKNMTDVDIANEMLIKRSNRKLEEEGNGIGWPRWLSTGDRRRLRSSSVIPNVVVVADGSGDYKTVSEAVAKAPVNSRKRYVIRIKAGVYRENVEIPSNKTNIMFIGDGRSRTIITGKRNVVDGSTTFRSASVAVAGEGFLARDITFENTAGPSKHQAVALRVSADLSAFYQCNMIAYQDTLYVHSQRQFFVNCLITGTVDFIFGNAAAVFQNCDIRARRPHRGQKNMVTAQGRTDPNQNTGIVFQKCRIGATSDLQSVKNNFPTYLGRPWKEYSRTVILQSDISDVIQPAGWHDWNGSFALKTLFYGEYQNTGASAGTHARVKWEGHKVITNESEAQSYTADKFISGGSWLYSTGFPFSLDL

12 Garb_00279 gi|21431800 NADP-dependent alkenal double bond reductase P2

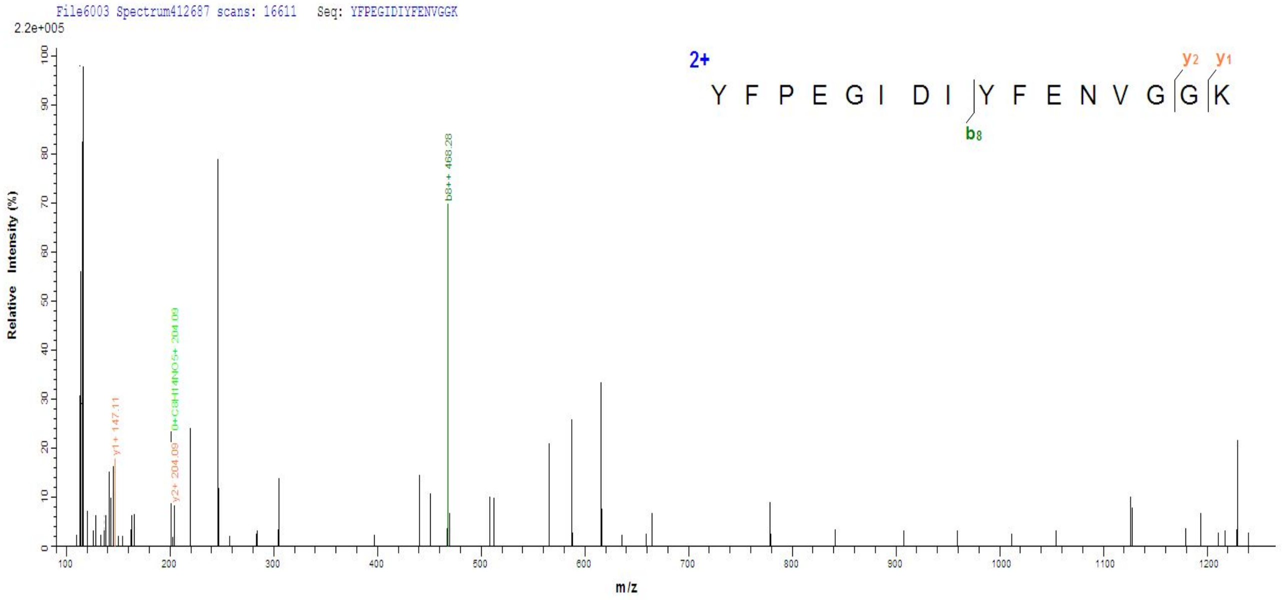

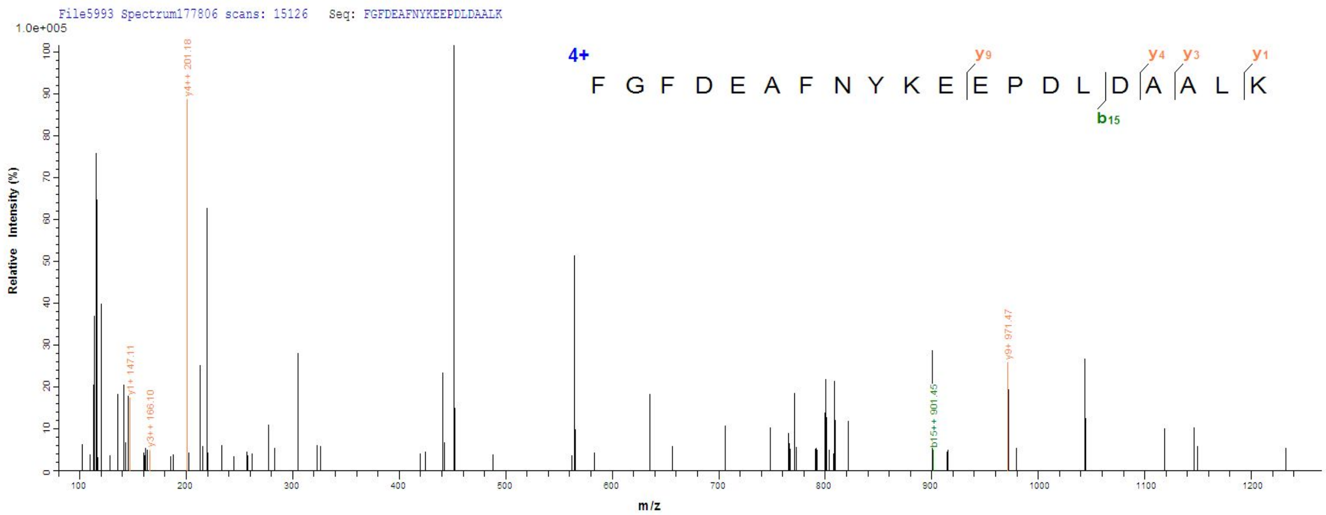

MADEALVKNKQVVLKNYVNAGLPKESDMEIKEKSIQLKVPEGTKDAILVKNLYLSCDPYMRNRMKKLEDSYVPSFEP
RLPISGYGVAKVVDSTHPEYKNGDLVWGTTGWEEYSLLRAPGLLFKIQHTDLPLTYYTGLLGMAGMTAYTGFYEICTP
KKGEYVYVSAASGAVGQLVGQFAKLLGCYVIDLLKNKFGFDEAFNYKEEPDLDAALKRYFPEGIDIYFENVGGKMLD
AVLLNMRVHGRIAVCGMISQYNNDKPEATHNLMYIIPKRVRMQGFIVSDFYHLYPKYLEMVIPSIKEGKITYIEDVAEGI
ESAPTALIGLFIGRNVGKQLVVVSRD

13 Garb_21160 gi|75268018 Probable fructose-bisphosphate aldolase 3

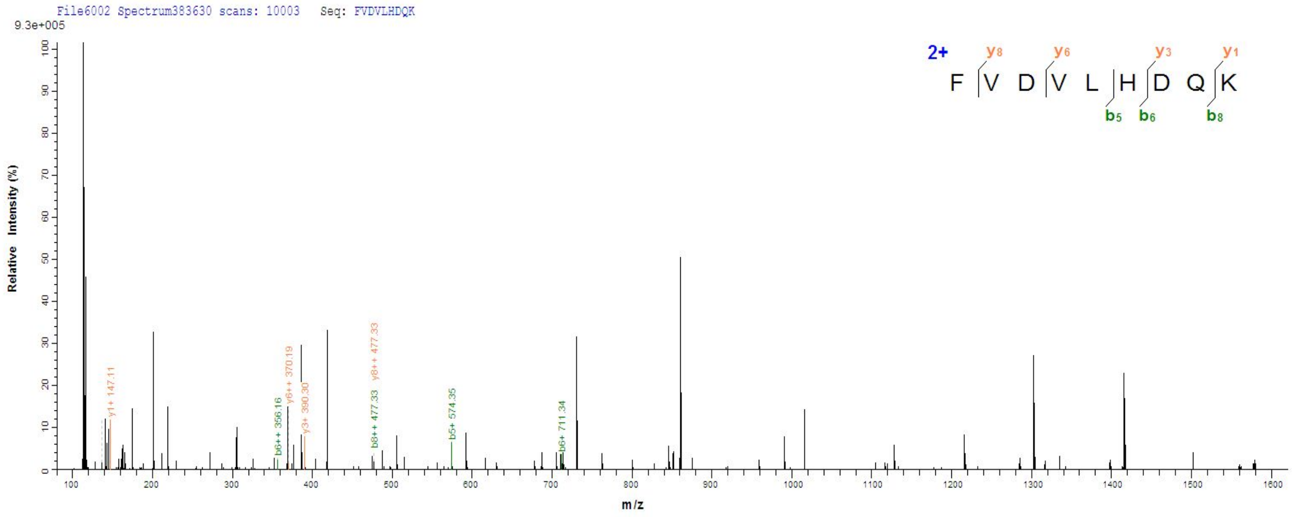

\

MACVSFAKLNAPSSSWIGGQQSLPQRSGSSARLATRRVSLPIRAGAYTDELIKTAKTIASPGRGILAIDESNATCGKRLSSI
GLDNTEPNRQAYRQLLLTTPGLGEYISGAILFEETLYQSTTDGKKFVDVLHDQKIVPGIKVDKGLVPLPGSNNESWCQG
LDGLSSRSAEYYKQGARFAKWRTVVSIPCGPSSLAVKEAAWGLARYAAISQDNGLVPVVEPEILLDGDHPIERTLEVAE
KVWAEVFYYLAENNVIFEGILLKPSMVTPGAEHKERANPETIAKYTLTMLKRRVPPAVPGIMFLSGGQSEMEATLNLHA
MNQSPNPWHVSFSYARALQNTVLKTWQGRPENVEAAQKALLVRAKANSLAQLGKYSAEGESEEAKKGMFVKGYTY

14 Garb_37955 gi|470127114 PREDICTED: aldose 1-epimerase-like

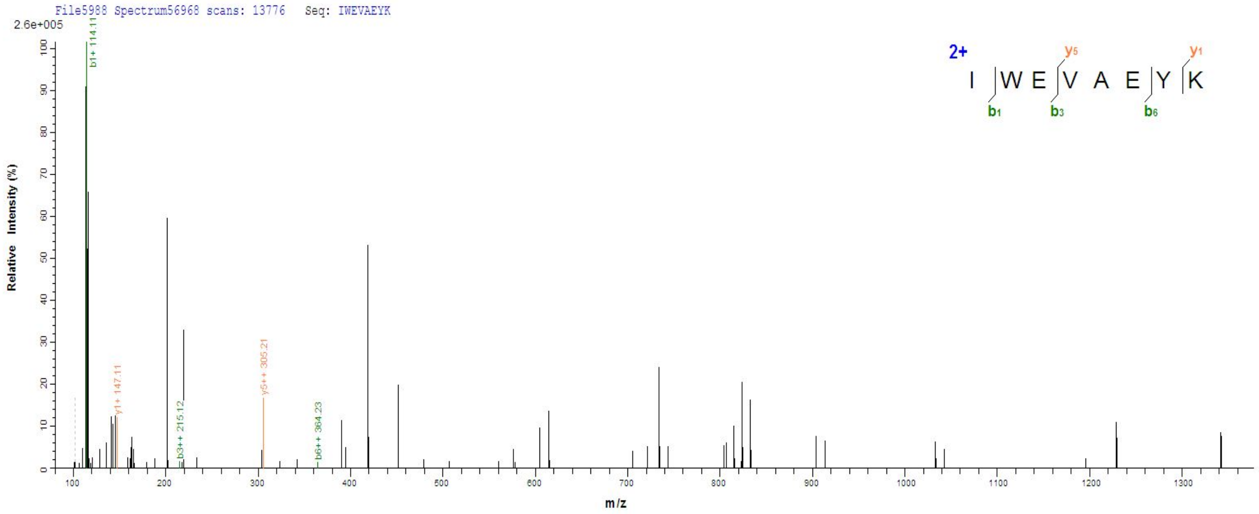

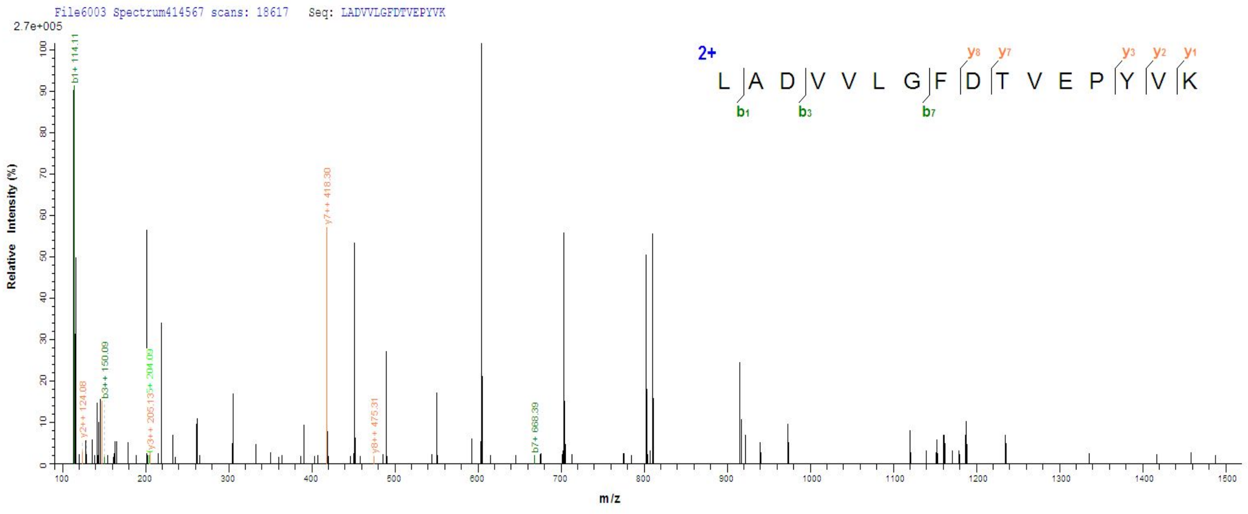

MADQPQIYELNNGTVVVKITNYGCTITSLSVPDKNGKLADVVLGFDTVEPYVKGASPYFGCIVGRVANRIKNGKFTLNGTEYTLPINKPPNSLHGGFKGFDKRIWEVAEYKKGDKPSITFKYRSSDGEEGYPSDVSVTATYTLTSTMTMKLDMEAVPENKATPINLAQHTYWNLAGHNSGNILDHTIQIWASEMTPVDENTVPTGEIKPIKGTPFDFTAEKKVGLQVHEVGLGYDRNYVLGSGEEKLGLKRAAKLKDPSSSRVLNLWTNAPGMQFYTGNHVNGVVGKGGAVYNKHAGLCLETQGFPNAINQPNFPSVVVQPGSKYQHTMLFEFSVE

15 Garb_27590 gi|37193998 phosphoenolpyruvate carboxykinase

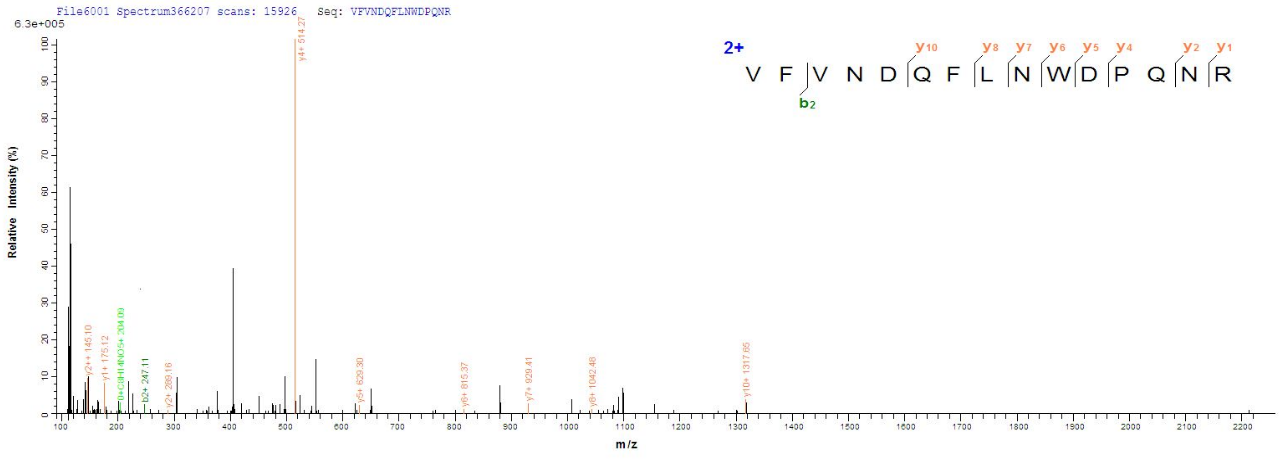

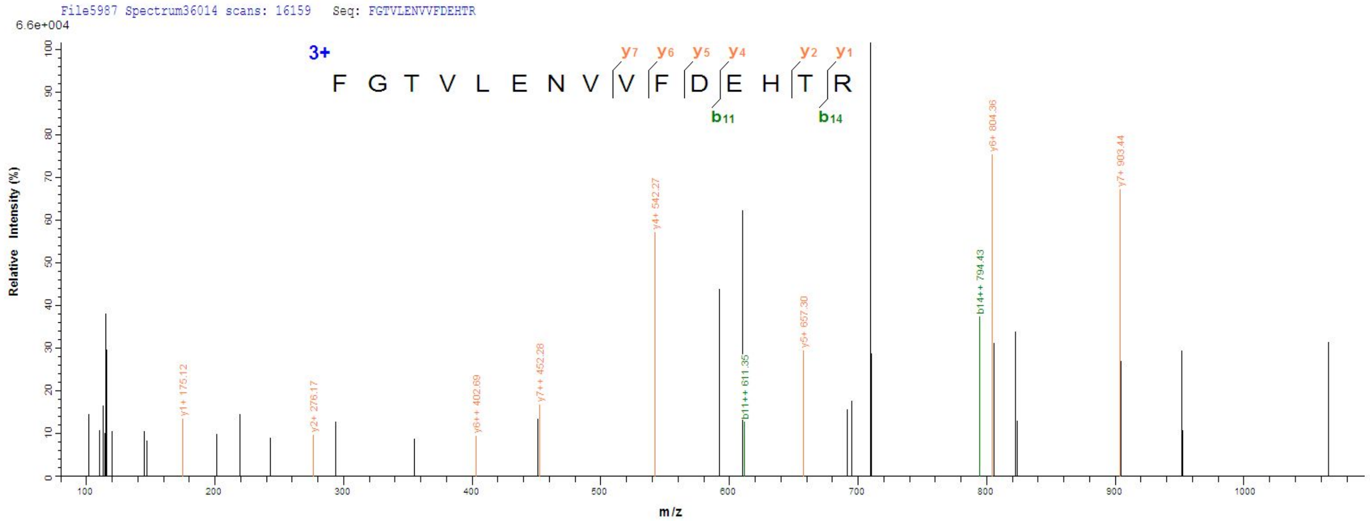

MAANGNGVSTTPKSPLARIQTQKNGGICHDDSGKPVKAQTIDELHSLQRKRSAPTTPLDGVQGAFATISEDERQRQQLQSISASLASLTRGTGPKVVRGDPAGKVQSVSHVAHHHHIEAPTISVSDSSLKFTHVLYNLSPAELYEQAIKYEKGSFITSTGALATLSGAKTGRSPRDKRVVIDDTTQDELWWGKGSPNIEMDEHTFMVNRERAVDYLNSLDKVFVNDQFLNWDPQNRIKVRIVSARAYHSLFMHNMCIRPTPEELENFGTPDFTIYNAGQFPCNRYTHYMTSSTSIDLNLARREMVILGTQYAGEMKKGLFSVMHYLMPMRQILSLHSGCNMGKDGDVALFFGLSGTGKTTLSTDHNRYLIGDDEHCWSDNGVSNIEGGCYAKCIDLSREKEPDIWNAIKFGTVLENVVFDEHTREVDYGDKSVTENLAMWIMLYEIHIIFNMILFFFLLLAENTRAAYPIEYIPNAKIPCVGPHPKNVILLACDAFGVLPPVSKLSLAQTMYHFISGYTALVAGTEDGIKEP

16 Garb_38448 gi|224057577 glutathione reductase

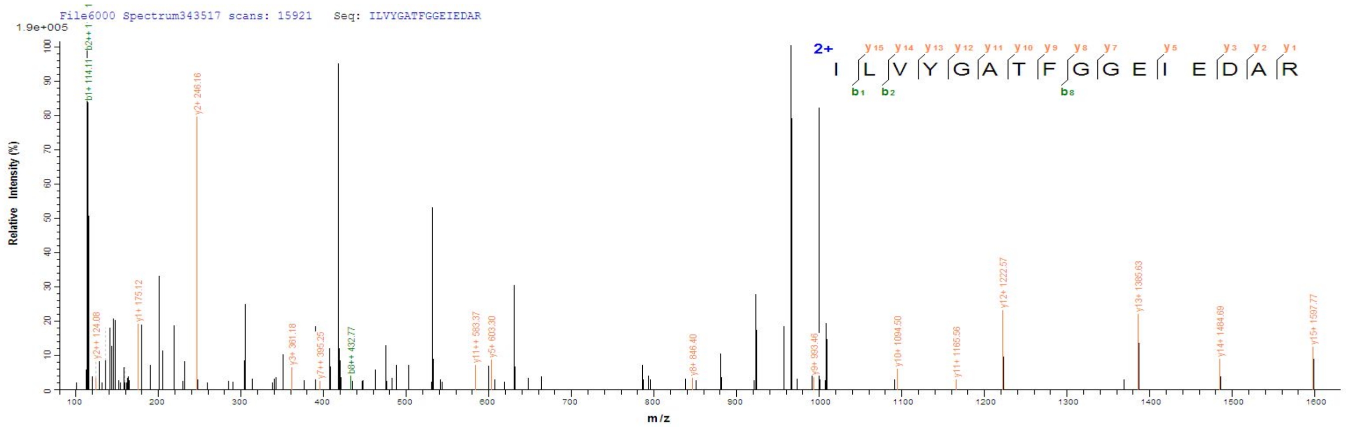

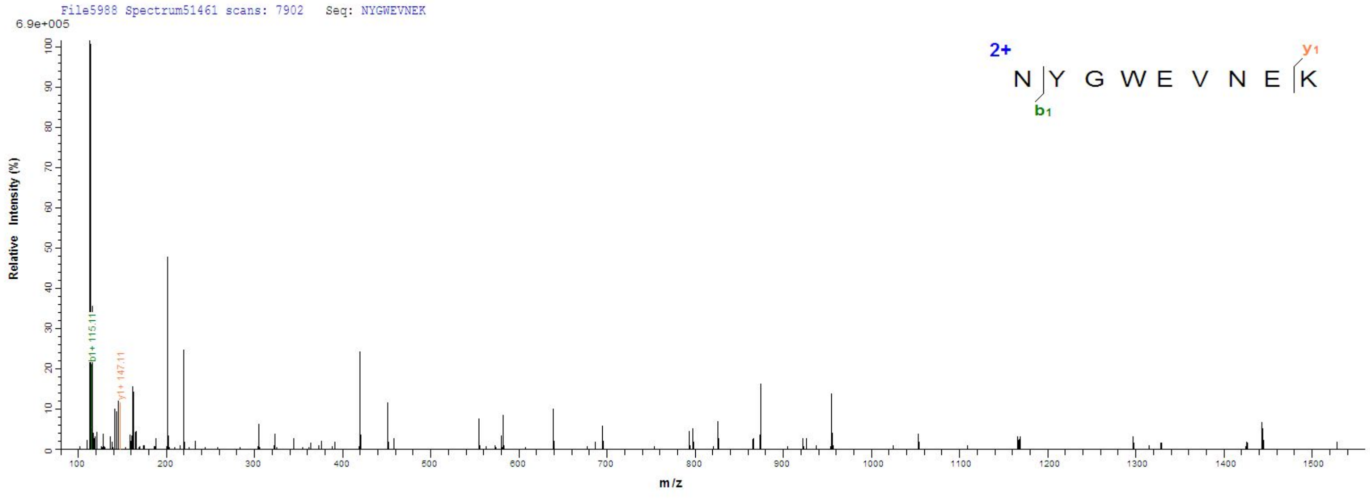

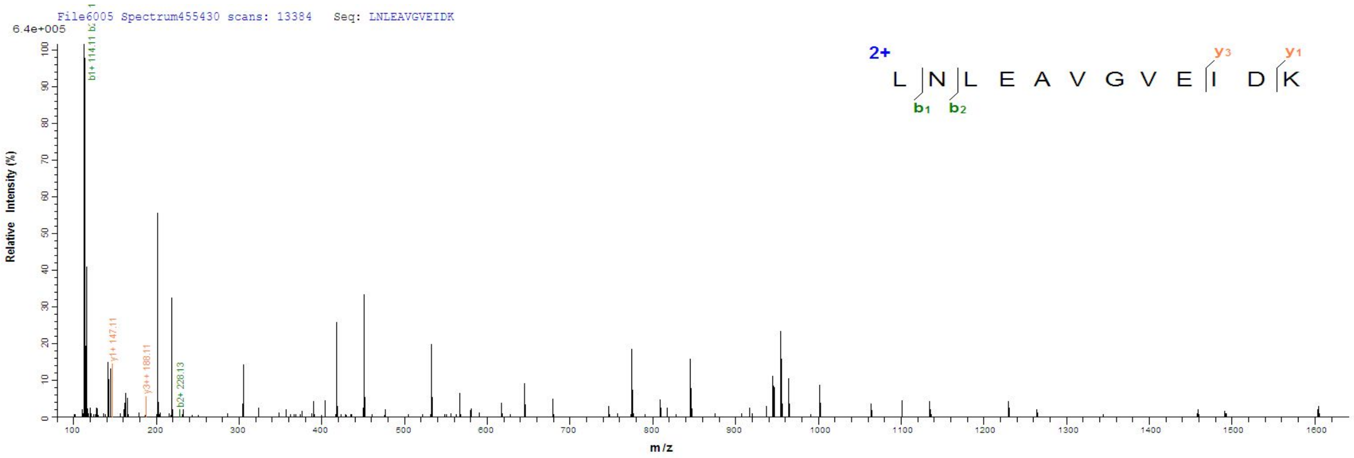

MARKMLVDGELNQTNKEGAEYDFDLFVIGAGSGGVRAARFSANYGAKVGICELPFHPISSEVIGGVGGTCVIRGCVPK
KILVYGATFGGEIEDARNYGWEVNEKLDFNWKKLLQKKTDEINRLNGIYKRLLSNASVKLLEGEGKIVGPNKVEVTQP
DGTKLSYSAKHILIATGSRAHRPPIPGQELAITSDEALSLDDLPKHAVVFGGGYIAVEFASIWRGLGATVDLFYRKELPLR
GFDDEMRAVVARNLEGRGIKLHPQTNLTELVKTDNGIKVITDHGGELIADVVLFATGRLPNTKRLNLEAVGVEIDKTGA
VKVDEYSRTNIPSIWAVGDVTNRMNLTPVALMEGTCFAKTVFGGESSKPDYRNIPCAVFSIPPLSIVGLSEEEAIEQVNGD
VLVFTSTFNPMKNTVSGRQEKTIMKLVVDAETDKVLGASMCGPDAPEIMQGIAVALKCGATKAQFDSTVGIHPSAAEE
FVTMRSVSRRVTA

17 Garb_00955 gi|356532527 dihydrolipoyl dehydrogenase-like

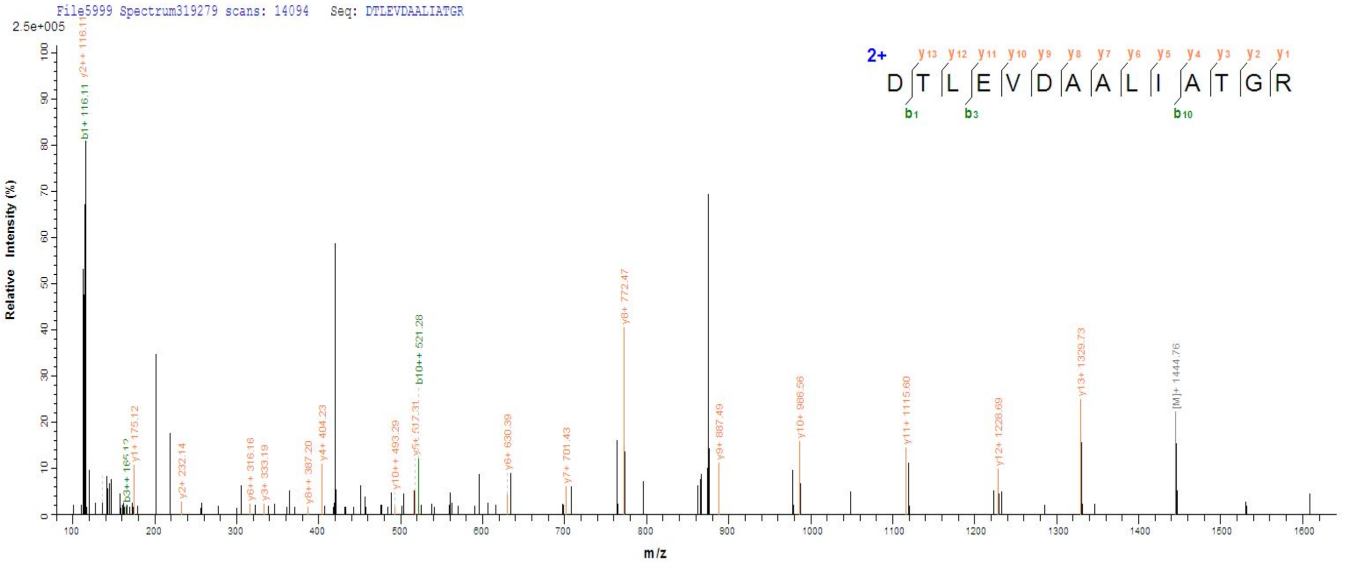

TAKNIIIATGSVPFVPKGIEVDGKTVITSDHALKLESVPDWIAIVGSGYIGLEFSDVYTALGSEVTFIEALDQLMPGFDP
EIGKLAQRVLINPRKIDYHTGVFATKITPAKDGKPVIIELIDAKTKEPKDTLEVDAALIATGRAPFTNGLGLENVVTQR
GFVPVDERLRVIDTNGNLVPHLYCIGDANGKMMLAHAASAQGISVVEQVTGQGHVLNHLSIPAACFTHPEISMVGLT
EPQAREKAQKEGFEVGVAKTSFKANTKALAENEGEGLAKLIYRPDNGEILGVHIFGLHAADLIHEASNAIALGTRIQ
DIKFAVHAHPTLSEVLDELFKSAKVSF

18 Garb_28259 gi|75262442 Nuclear transcription factor Y subunit B-2

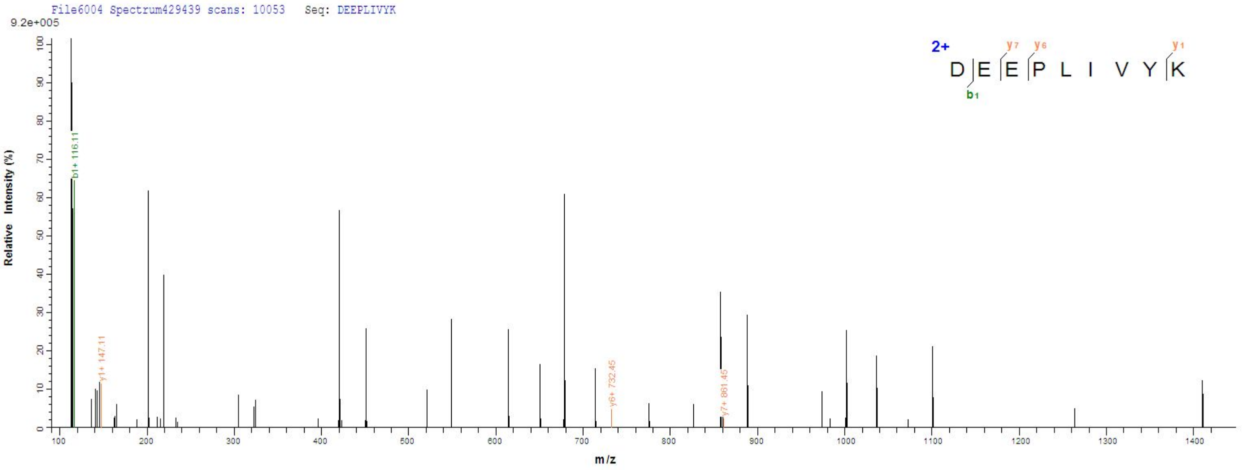

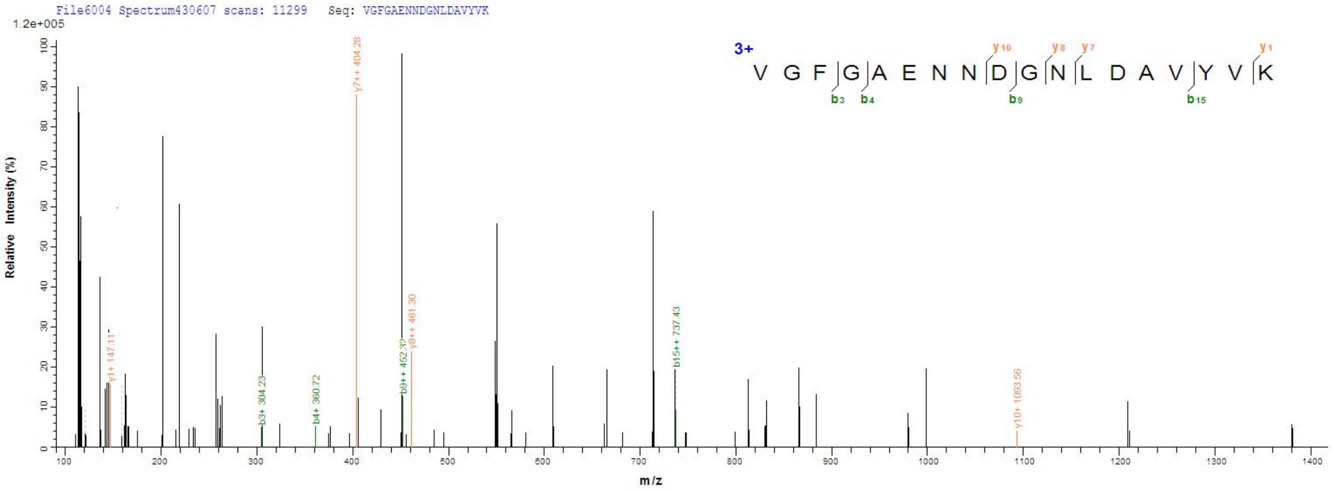

MADSDDESGEQNNNGGNAHSEASGREQDRFLPIANVSRIMKKALPPNAKISKDAKETVQECVSEFIIGSGGGEFNGG
GGMYGGMMMGHHQGHMYSSGGHEATPPHSLLIHKYHHIFPRTHSYLLHSQPTMSPATTALFFLLLLFSTSNAHNITR
ILAKHPEFSTFNHYLTLTHLASEINRRETITVLALNNAAMSSLLSKQLSLYTLKNVLSLHVLVDYFGSKKLHQITNGTA
LTSTMFQASGAAPGSSGYINITDLKGGKVGFGAENNDGNLDAVYVKSVAEIPYNISVLQIKIMSKQGCKAFADLLIAS
GADATFNENIDGGLTVFCPTDPVIKDFMPKYKNLTASKKVSLLLYHGVPVYQSMQMLKSNNGIMNTLATDGANKY
DFTIQNDGEVVTLETKVMTAKITGTLKDEEPLIVYKINKVLLPRELYKPVEAPAESPKPSKSKS

19 Garb _00970 gi|341958560 CASP-like protein POPTRDRAFT_820933

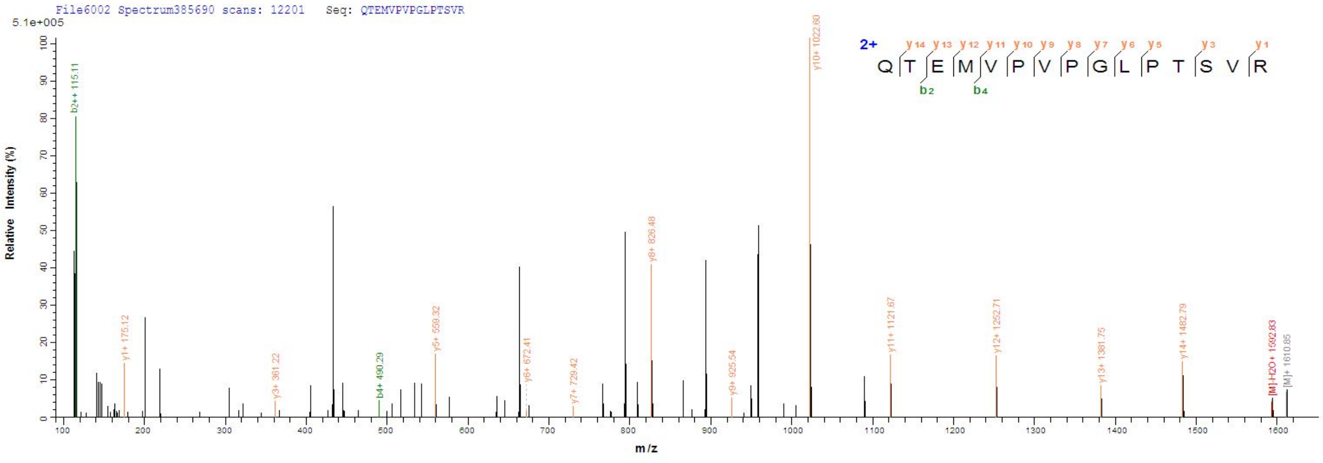

MESTDKPAAVAATTTTTPSTTVAASKCPFNYSAVDVGLRVVLFAAAVIAVVVMATSKQTEMVPVPGLPTSVRVPFSAEF TDSPAFVYFMAALSTTGLYSIITALASILLGRKPSYTNTILLAFALMDVVFVGIVASATGSAGSVAYIGLRGNSHVRWDKI CNVYDEFCRHIGTAIAFSLFAAILLVFLTMFSTFALYKKIR

20 Garb_06245 gi|356521678   putative DNA repair protein RAD23-1-like isoform 1

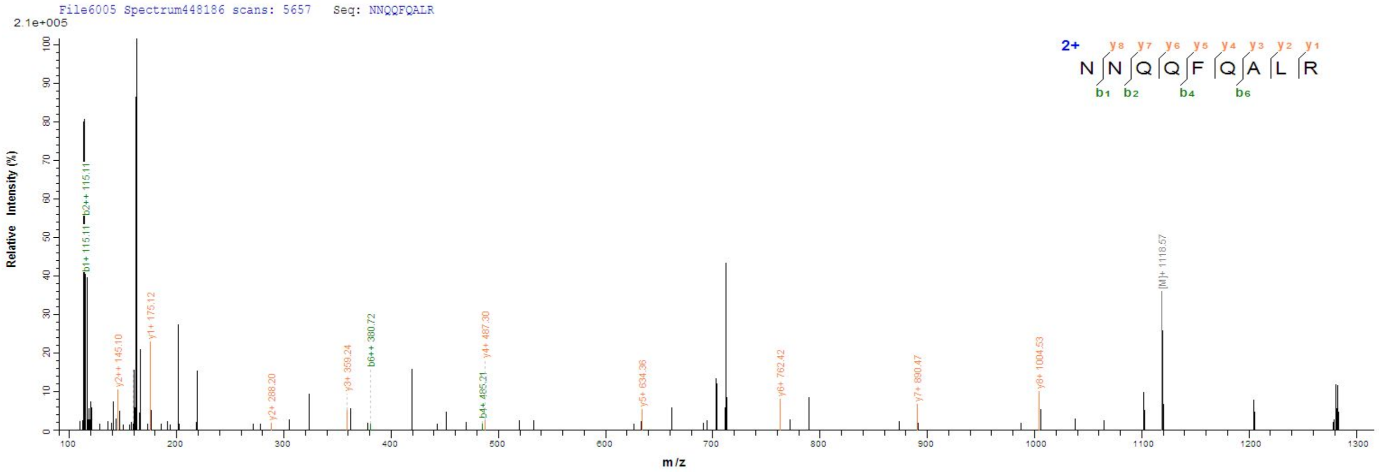

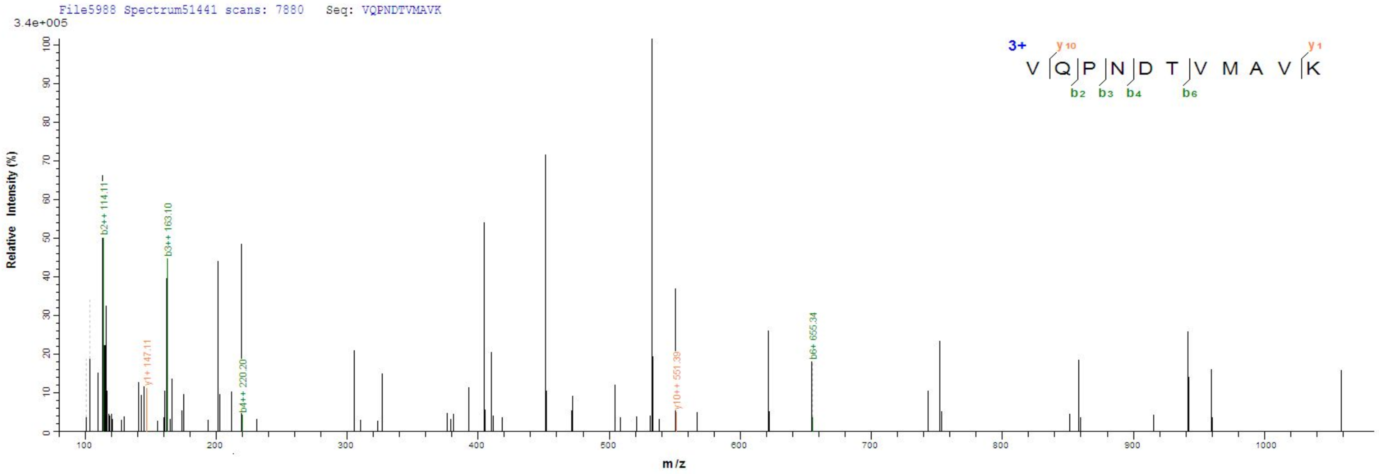

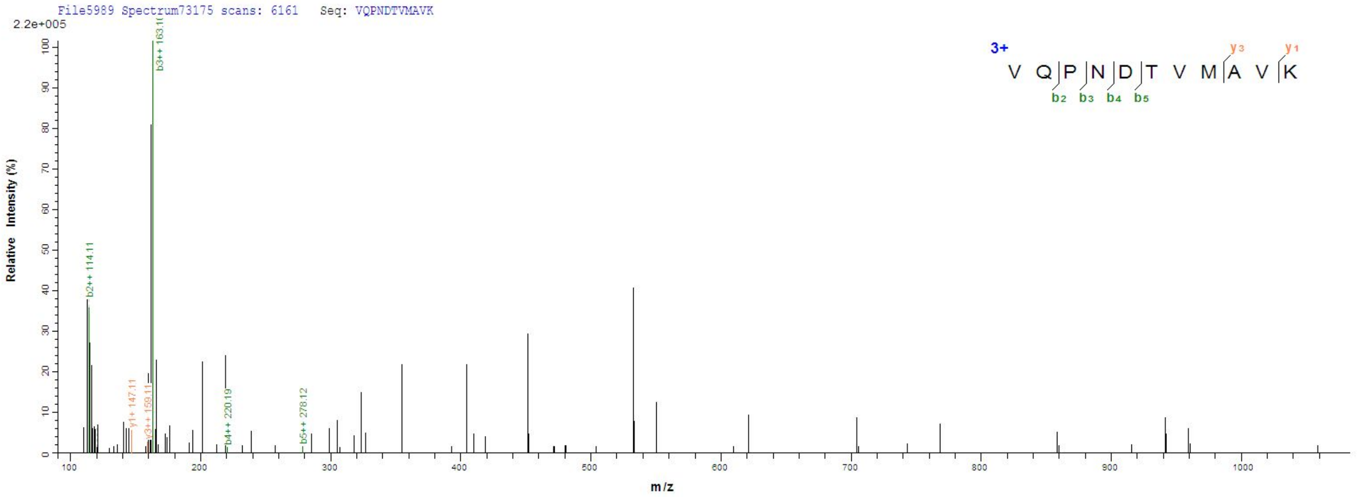

MKLIIKTLKGSHFEIRVQPNDTVMAVKKNIEDIQGKDNYPCGQQLLIHNGKVLKDETTLAENKVSEDGFLVVMLSKVY IKKKKTFLLVYIYIYLLLFSFSYFYYFLIYFQFIGVFWQSKSLGSTGASSAQVEHYVPVYVYNQPFDCLPQQPASSTPSTT VPASAPSSNSTPATGAPAQASQQTNTYSQAASNLVAGNNLEQTIQQLMDMGGGNWDKETVTRALRAAYNNPERAVDY

LYSGIPESAEVAVPVAHFSTSQTTETGAAPAAPVSGAPNSSPLNMFPQETLSGAAAGGDGSLDFLRNNQQFQALRSMVQSNPQILQPMLQELGKQNPQLLRLIQEHHAEFLQLINEPLEGSEGDIFDQAEQEMPHAVNVTPAEQEAIERLEAMGFDRALVIEAFLACDRNEELAANYLLENGGDFED

21 Garb_02220 gi|55976204 Transcription factor HY5

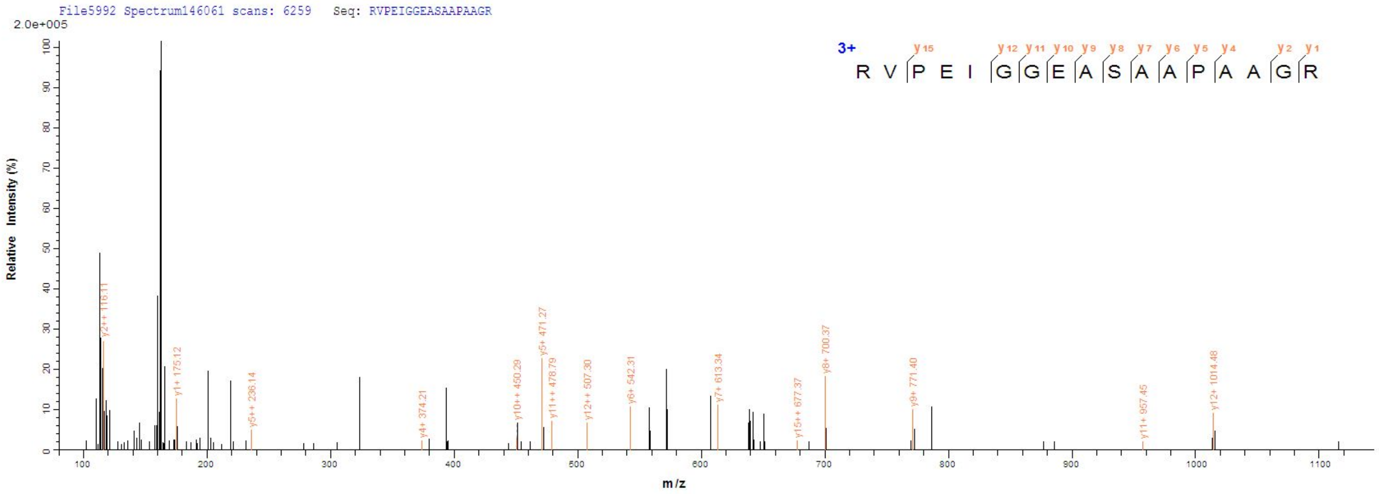

MQEQGTSSIAASSLPSSSERSSSSALQVEVKEGMESDEEIRRVPEIGGEASAAPAAGREPGSLTRLDRPQPSGEGGQRKRGRSPADKENKRLKRLLRNRVSAQQARERKKAYLNELETRVRDLEKKNSELEERLSTLHNENQMLRQIVKNTTASRRGGNGSSNAADGTL

22 Garb_16614 gi|224133758 histone H1

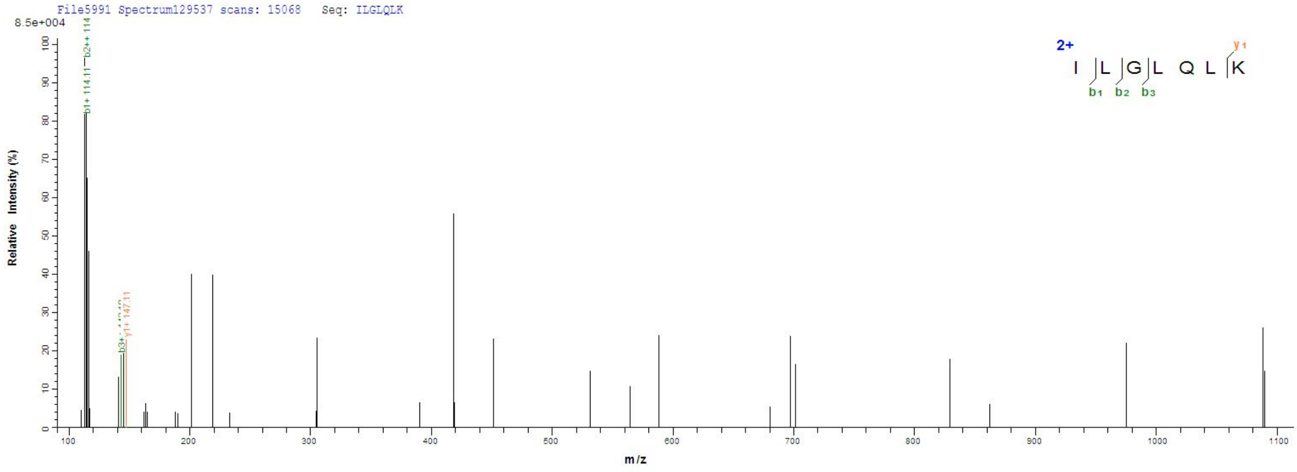

MATAEPEVPVTEQQQPAAAEEPKPAEKLVKEKRPRAPKEKKPKQPKSAAHPPYFQMIKEALLALKEKSGSSPYAIAKYMEEKHKAVLPANFRKILGLQLKNSAARGKLIKIKASYKLSEAGKKERAPVTKAKTEKKAKPASKPKKAEATKKPTKRVGAKKKSTPAKPKQPKSIKSPAAKKAKKAAA

23 Garb_04359 gi|75321585 Zinc finger CCCH domain-containing protein 40

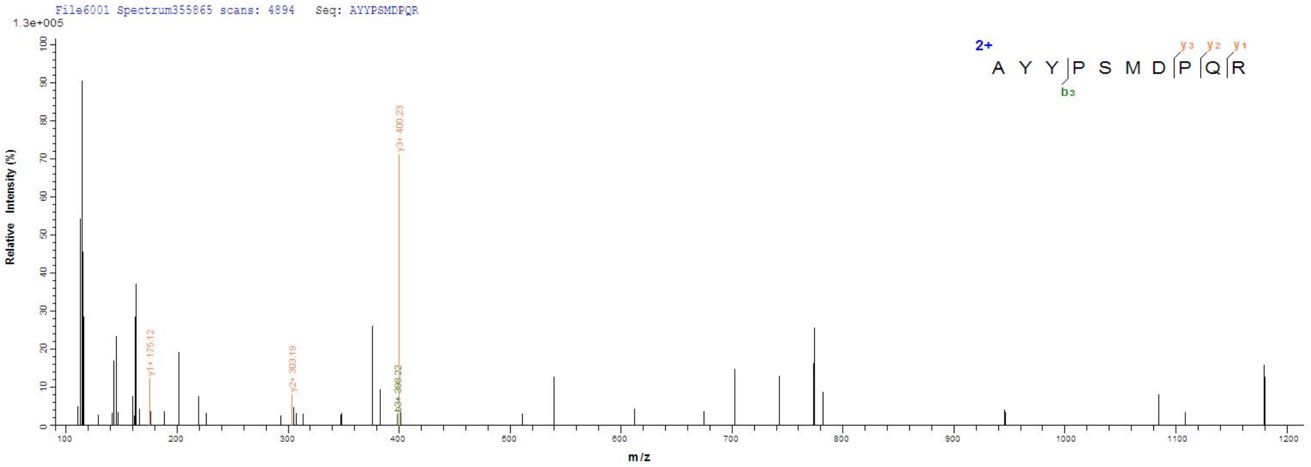

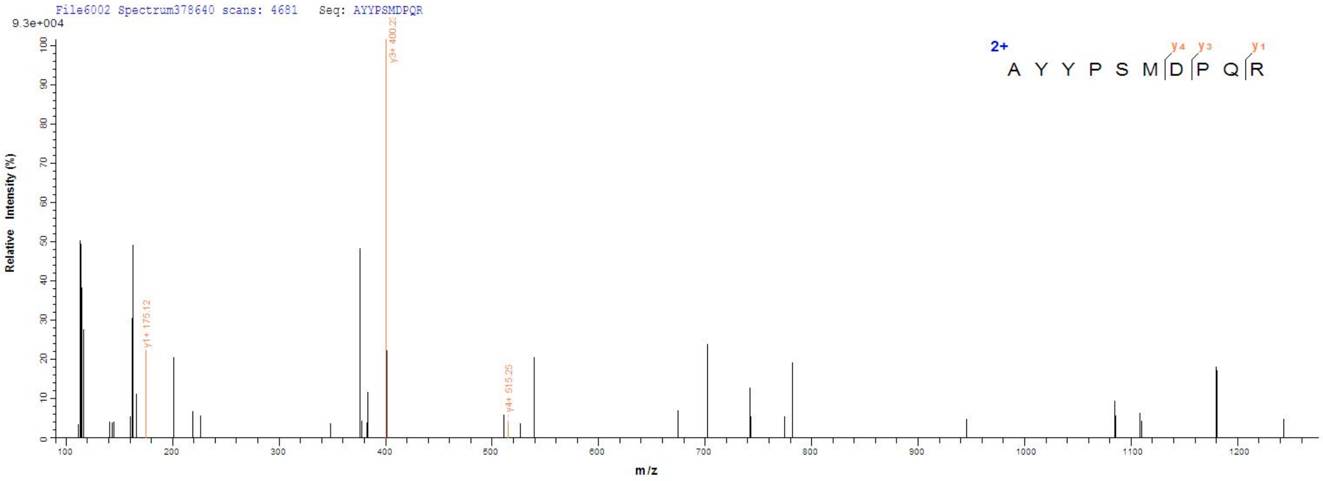

MAHRLLRDHEADGWERSDFPIICESCLGDNPYVRMTKADYDKECKICTRPFTVFRWRPGRDARYKKTEVCQTCSKLK
NVCQVCLLDLEYGLPVQVRDTALSINSNDAIPKSDVNREYFAEEHDRRARAGLDYESSYGKVRPNDTILKLQRTTPYY
KRNRAHICSFYVRGECTRGAECPYRHEMPEAGELSQQNIKDRYYGVNDPVALKLLNKAGEMPSLEAPEDESIKTLYVG
GLDKRITEQDLRDNFYAHGEIESIKMVLDKACAFVTYTTREGAEKAAEELSSKLVIKGLRLKLMWGKPQAPRPESETS
DGSRQQAAVAHSGMLPRAVISQQQNQFQPSGPGMHDQPPPMQYFNIPPPPQMDRAYYPSMDPQRMGALVPSQDGENK
LGSDKQQAQHYSYQGMPPPPPPGQYPHQHYPPYGYMQPMPPYQQYPPYHSAMPPPRGPPQHYQHSGPSRPPPPVSGP
ASTSVQPPPASTSSGSAPPPPPPPPVPVPSAAASGSSQQ

24 Garb_29307 gi|17865566 60S ribosomal protein L36-3

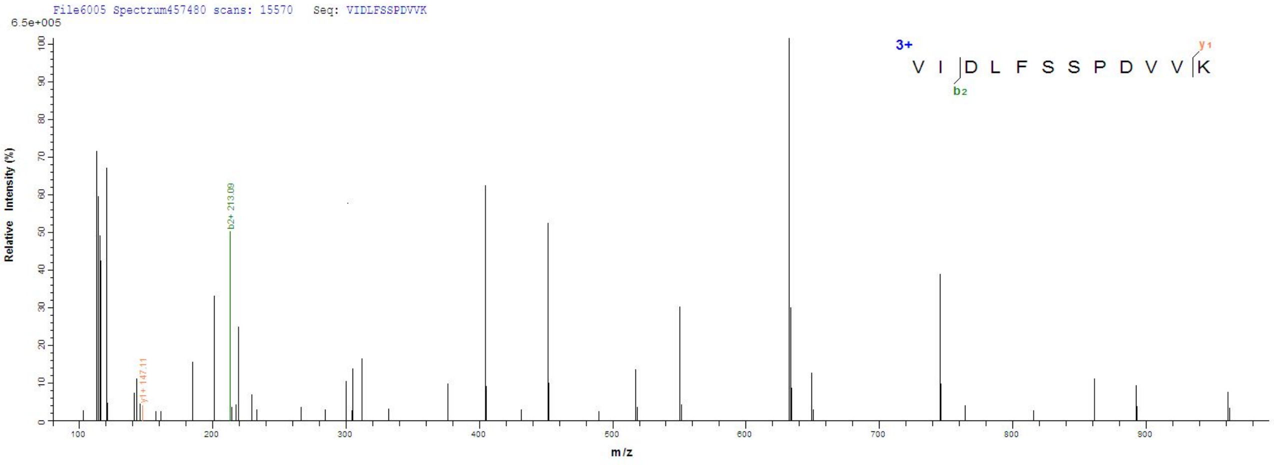

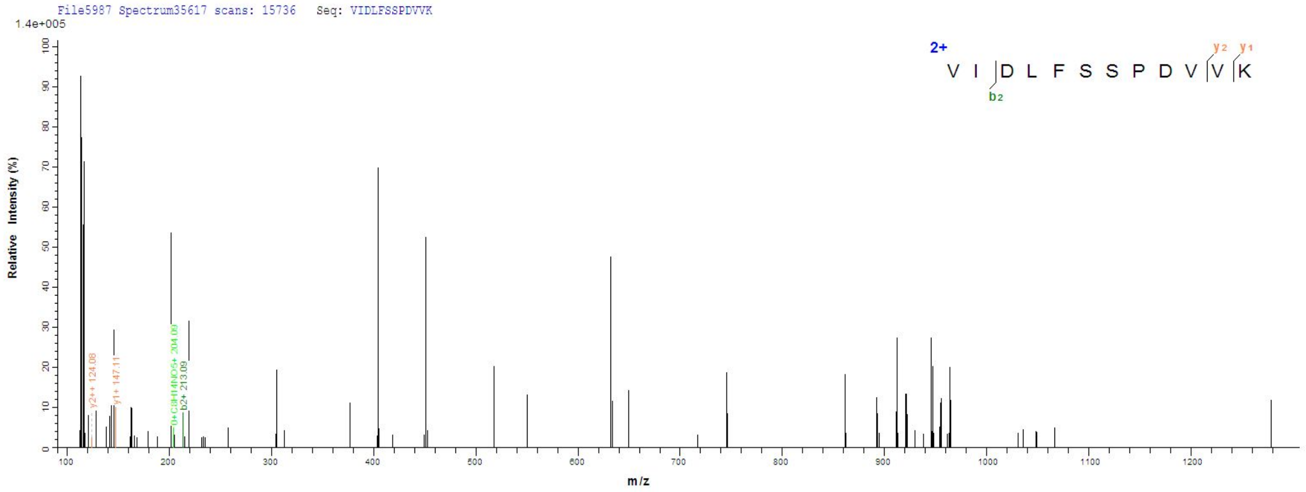

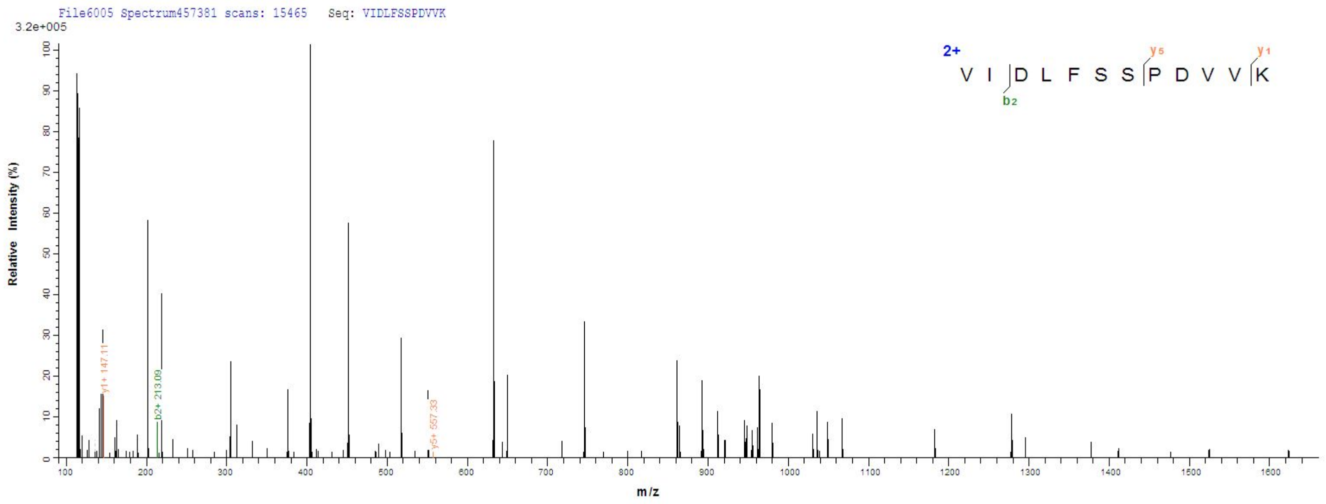

MAYATMKPTKPGLEESQEQIHKIRITLSSKNVKNLEKVCADLVRGAKDKRLRVKGPVRMPTKVLHITTRKSPCGEGTN TWDRFELRVHKRVIDLFSSPDVVKQITSITIEPGVEVEVTIADS

25 Garb_09241 gi|22096379 40S ribosomal protein S30

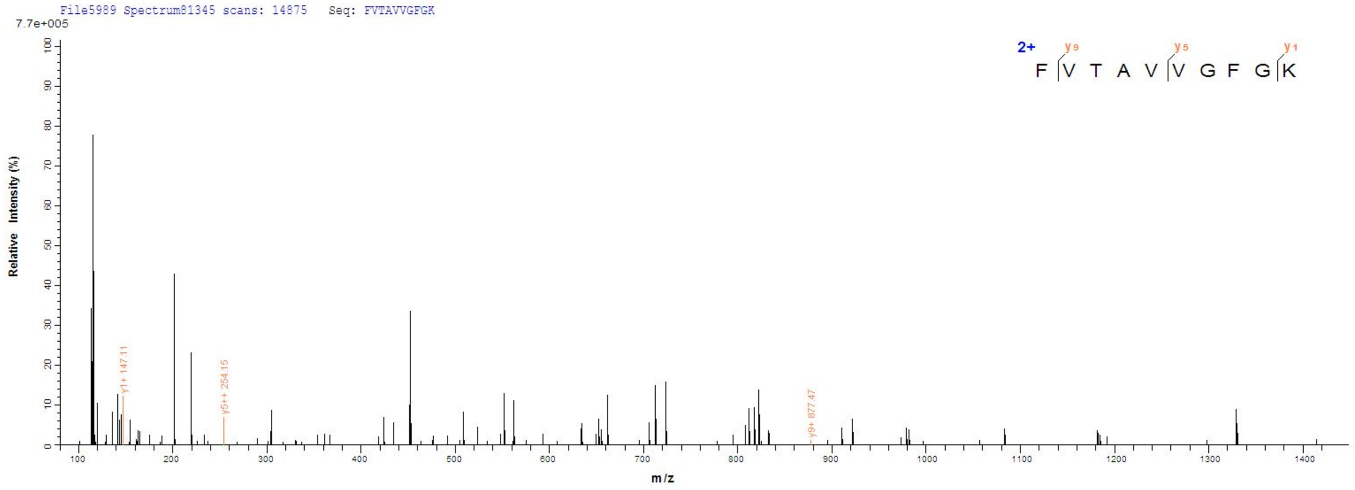

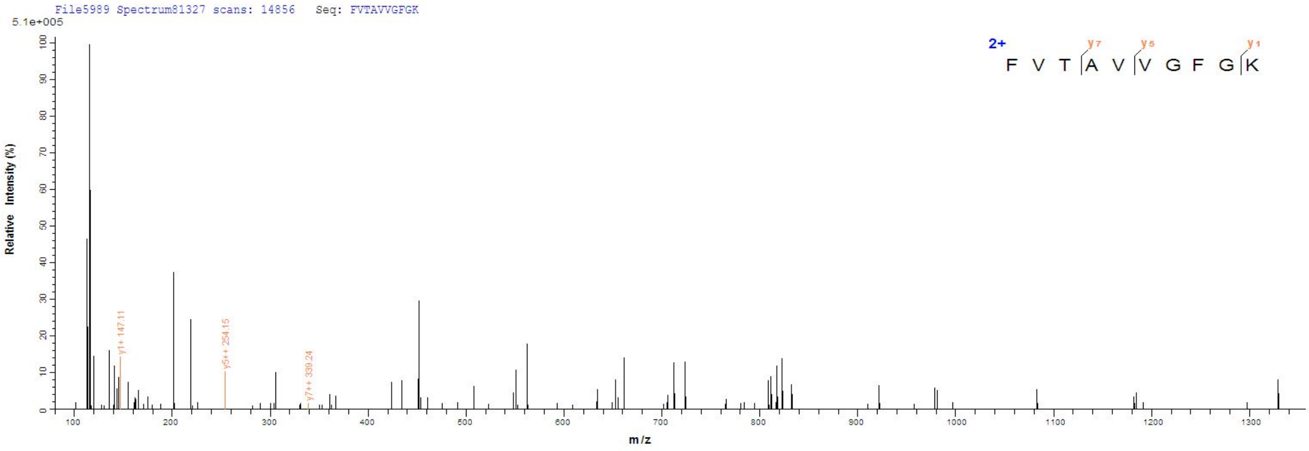

MVEKTRGRKEEVVTREYTINLHKRLHGCTFKKKAPKAIKEIRRFAEKAMGTKDVRVDEELYSLVTVAEIPAEGLKGKV
HGSLARAGKVRGQTPKVAKQDKKKKPRGRAYKRMQYNRRFVTAVVGFGKKRGPNSSEK

26 Garb_29307 gi|17865566 60S ribosomal protein L36-3

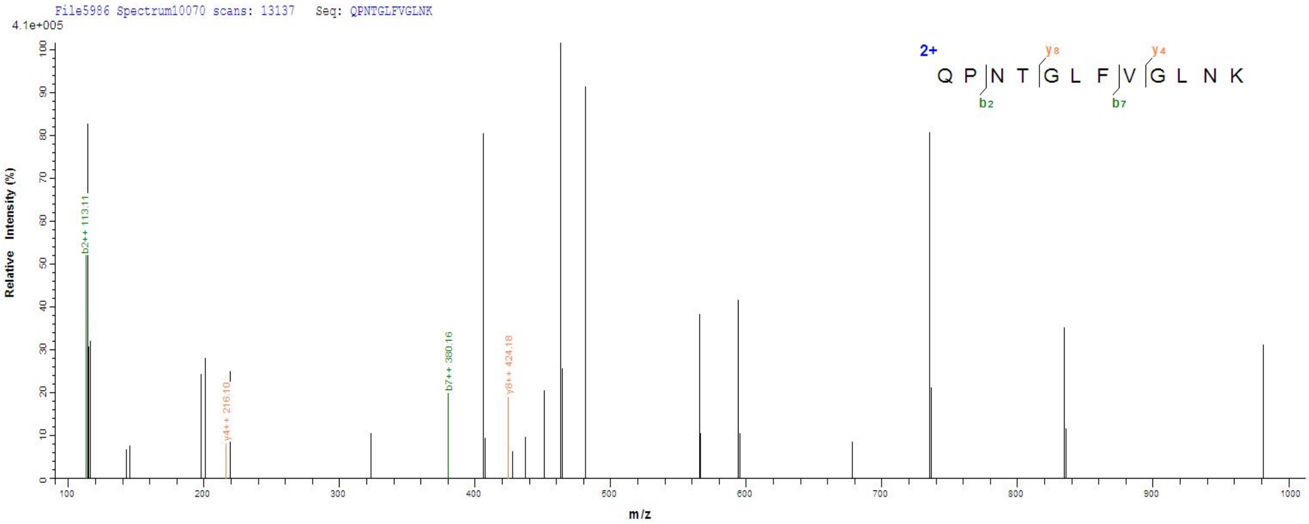

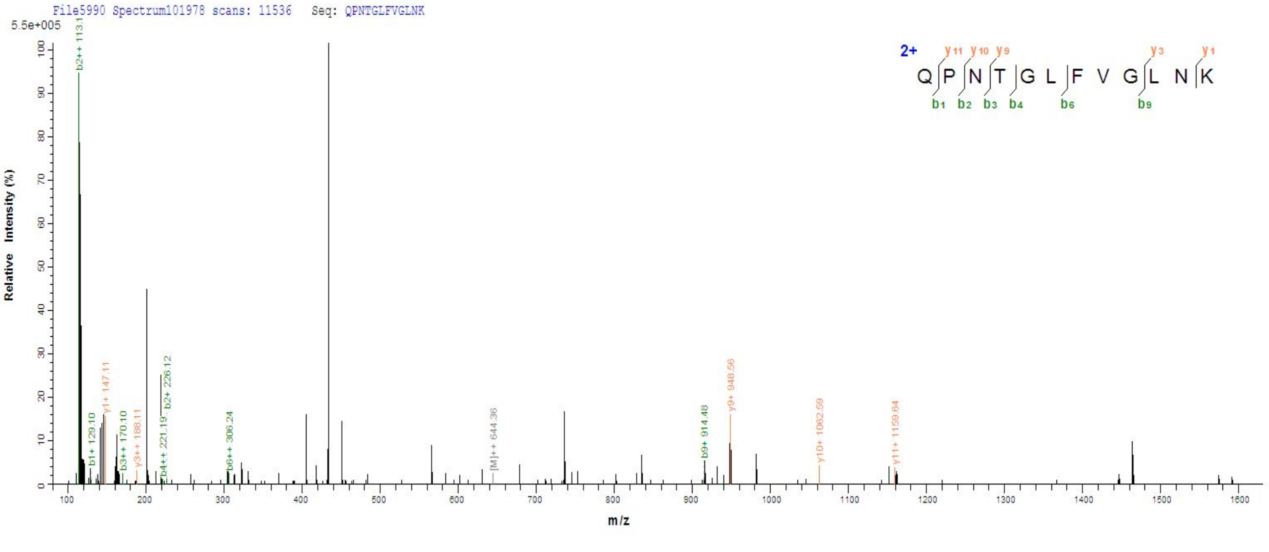

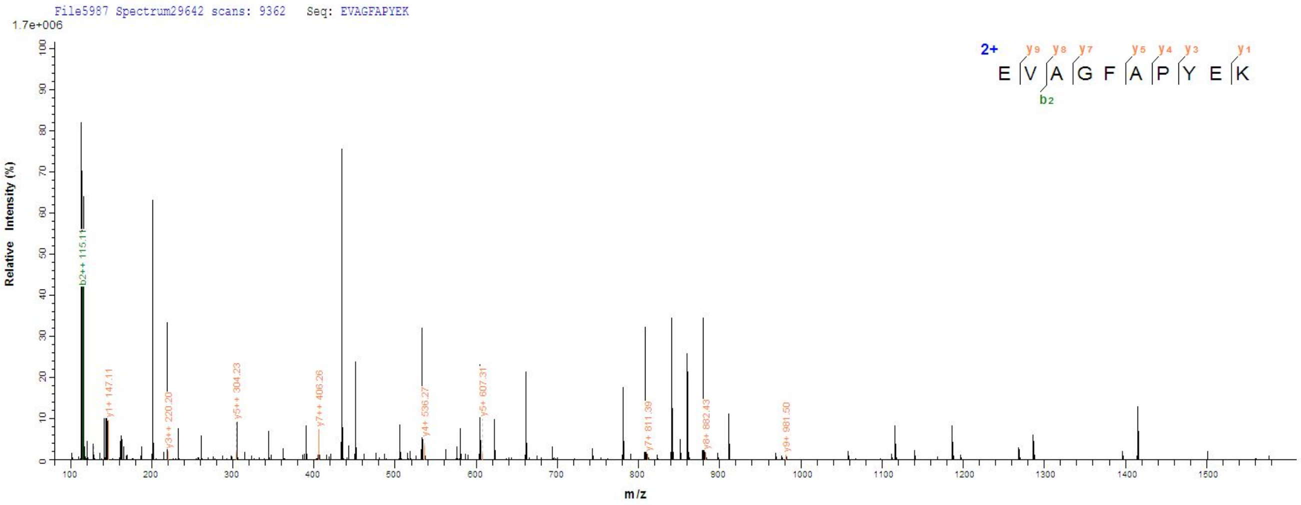

MATKQPNTGLFVGLNKGHAVTKKELAPRPSNRKGKTSKRVHFVRNLIREVAGFAPYEKRITELLKVGKDKRALKVAK
RKLGTHKRAKKKREEMSSVLRKMR

27 Garb_32910 gi|24473796 60s acidic ribosomal protein

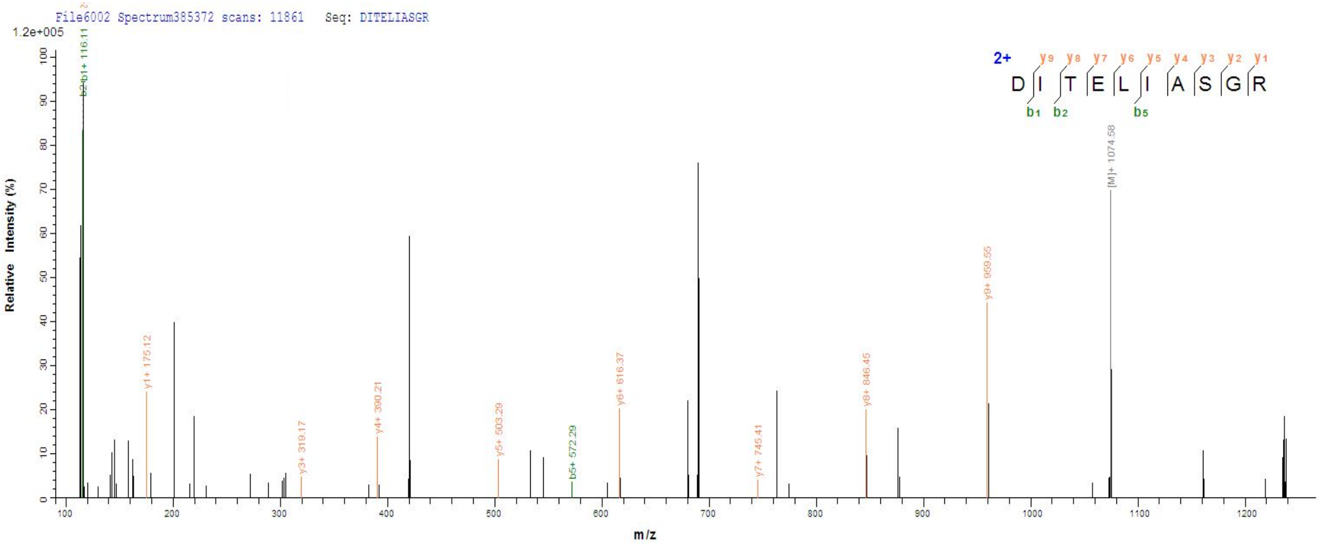

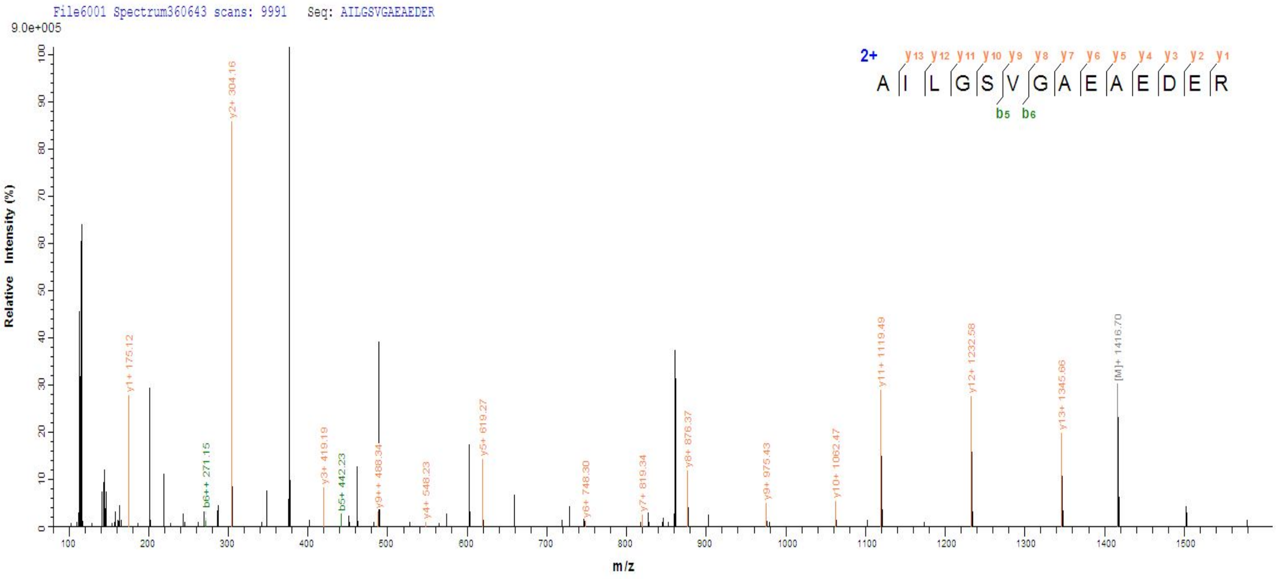

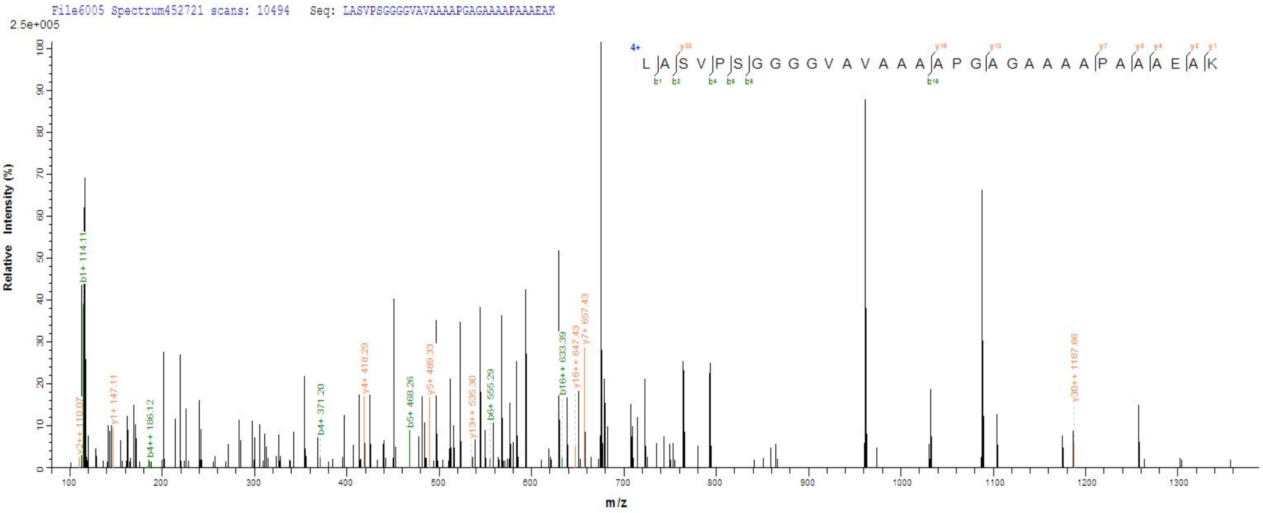

MKVVAAYLLAVLGGNTSPSADDLKAILGSVGAEAEDERIQLLLSEVKGKDITELIASGREKLASVPSGGGGVAVAAAAPGAGAAAAPAAAEAKKEEKAEEKEESDDDMGFSLFD

28 Garb_02116 gi|6015064 Elongation factor 1-delta

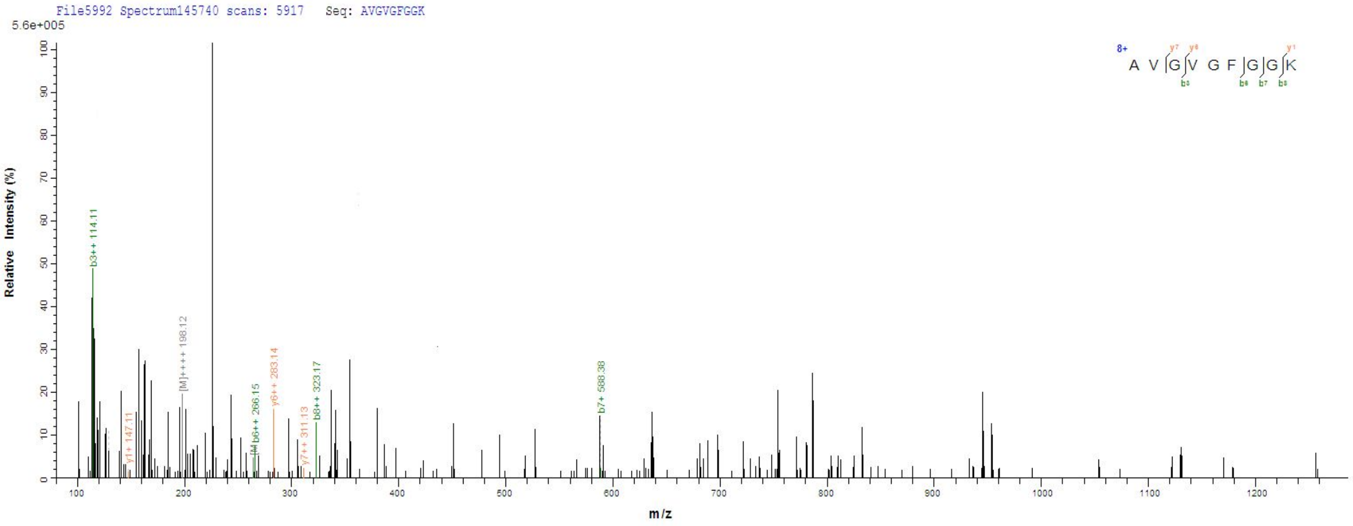

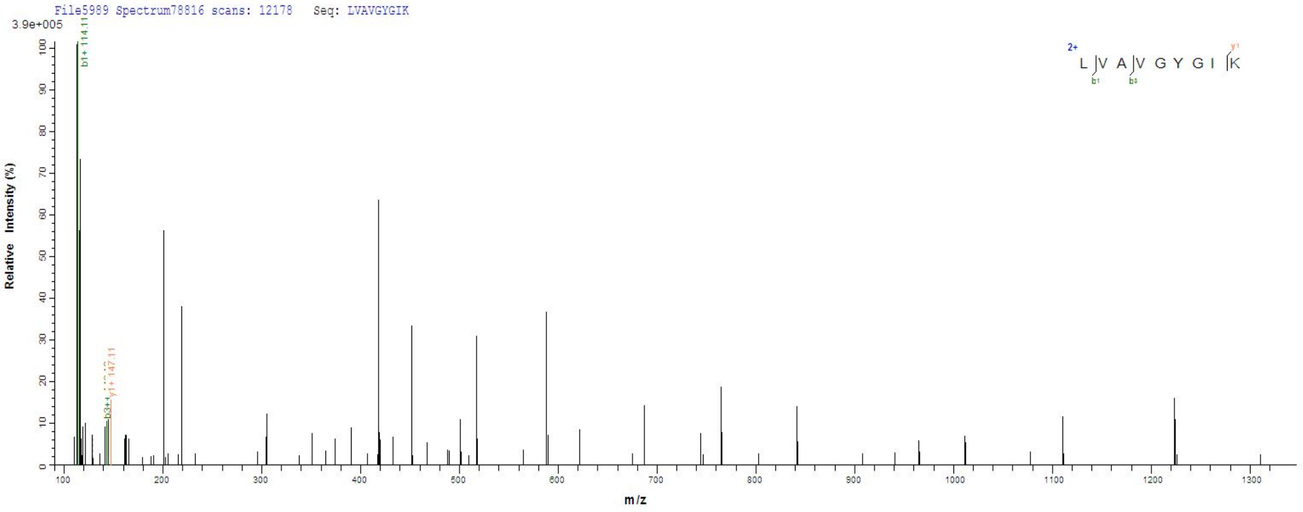

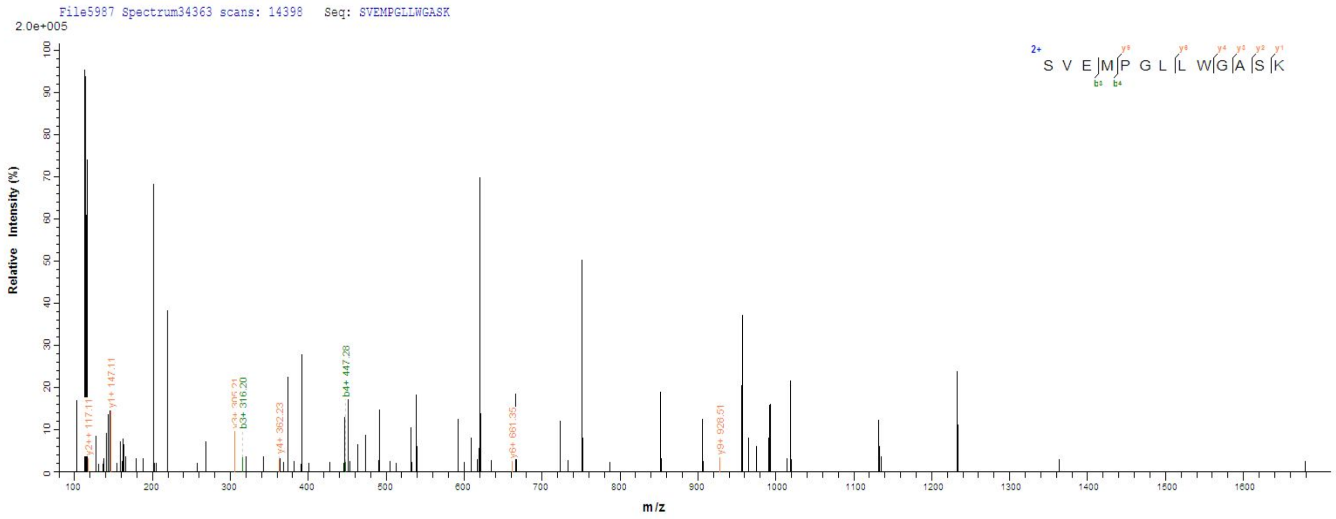

MAVTFSNLHTESGLKALNDFLSGKSYISGNKLTKDDIKVYAAVLKNPGDSFPNVSQWYNSVSSQLAASFPGKAVGVGFGGKAAPAESAKTEAADDDDDLDLFGDETEEDKKAAEEREAAKKSAKKKESGKSSVLMDVKPWDDETDMKKLEEAVRSVEMPGLLWGASKLVAVGYGIKKLQIMLTIVDDLVSVDTLIEEYLTTEPRNEYIQSCDIVAFNKI

29 Garb_36269 gi|23503072 Eukaryotic translation initiation factor 3 subunit I

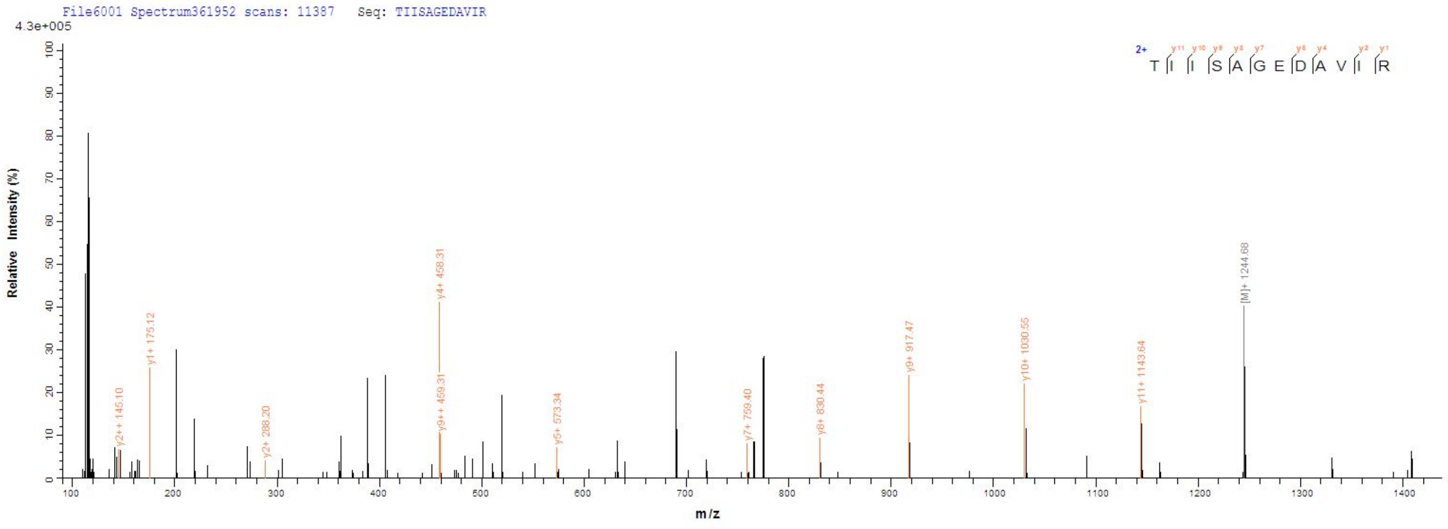

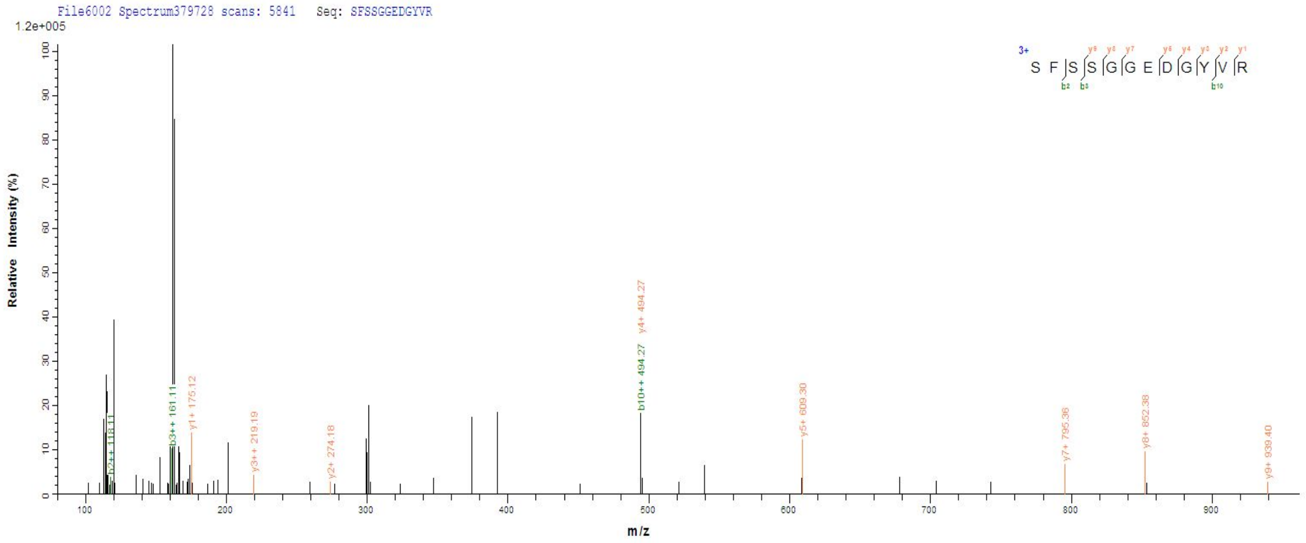

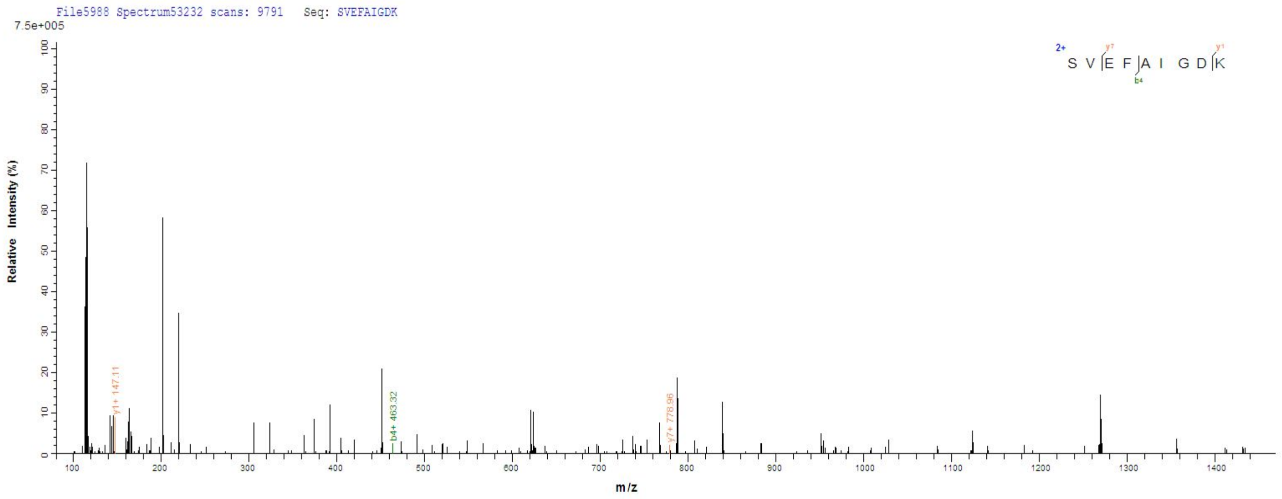

MRPILMKGHERPLTFLKYNRDGDLLFSCAKDHTPTVWFADNGERLGTYRGHNGAVWTCDVSRDSTRLITGSADQTVKLWNVQKGTQLYTFNFGSPARSVEFAIGDKLAVITTDPFMELTSAIHVKRIAGDPTEQTEESVLVIKGPQGRINRAVWGPLNRTIISAGEDAVIRIWDSETGKLLKESDKESGHKKTITSLTKAADGSHFLSGSLDKSAKLWDTRTLTLIKTYSTERPVNAVAMSPLLDHVVLGGGQDASAVTTTDHRAGKFEAKFYDKILQEEIGGVKGHFGPINALAFNPDGKSFSSGGEDGYVRLHHFDPDYFNIKI

30 Garb_25276 gi|18803 polyubiquitin protein

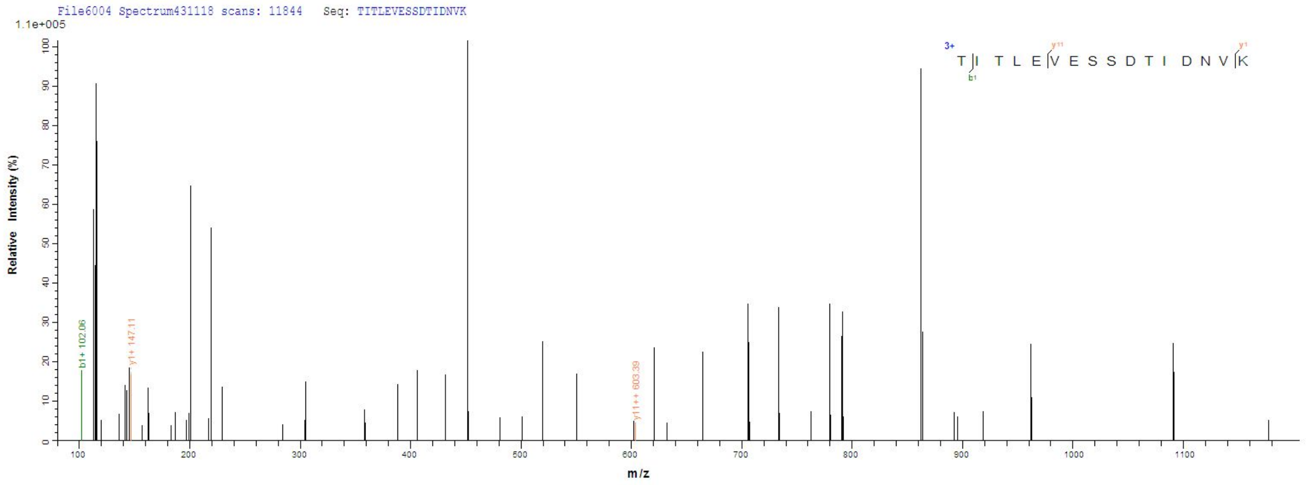

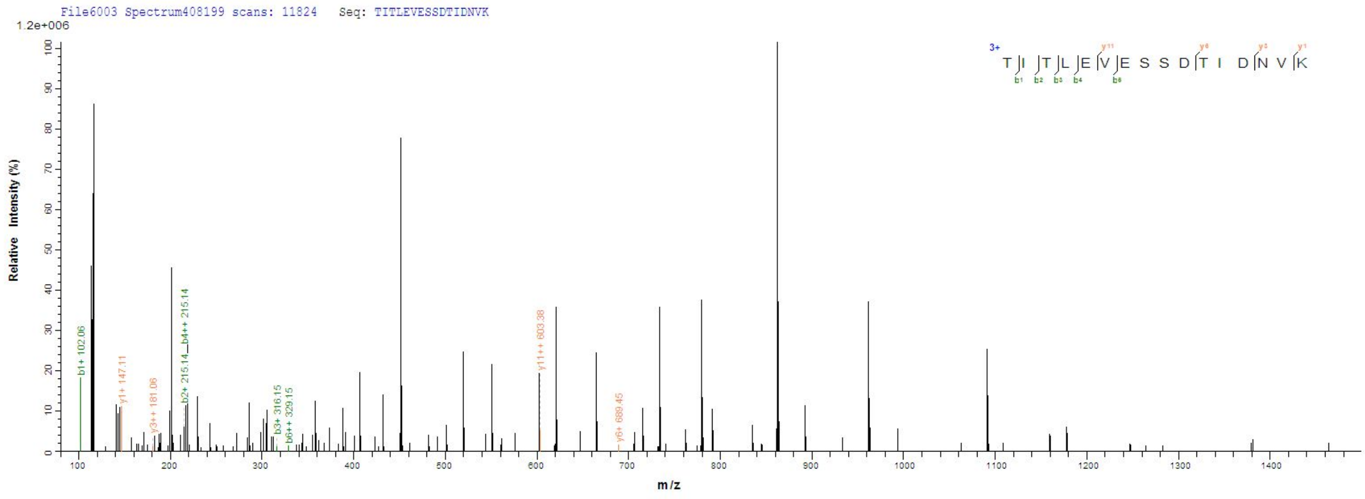

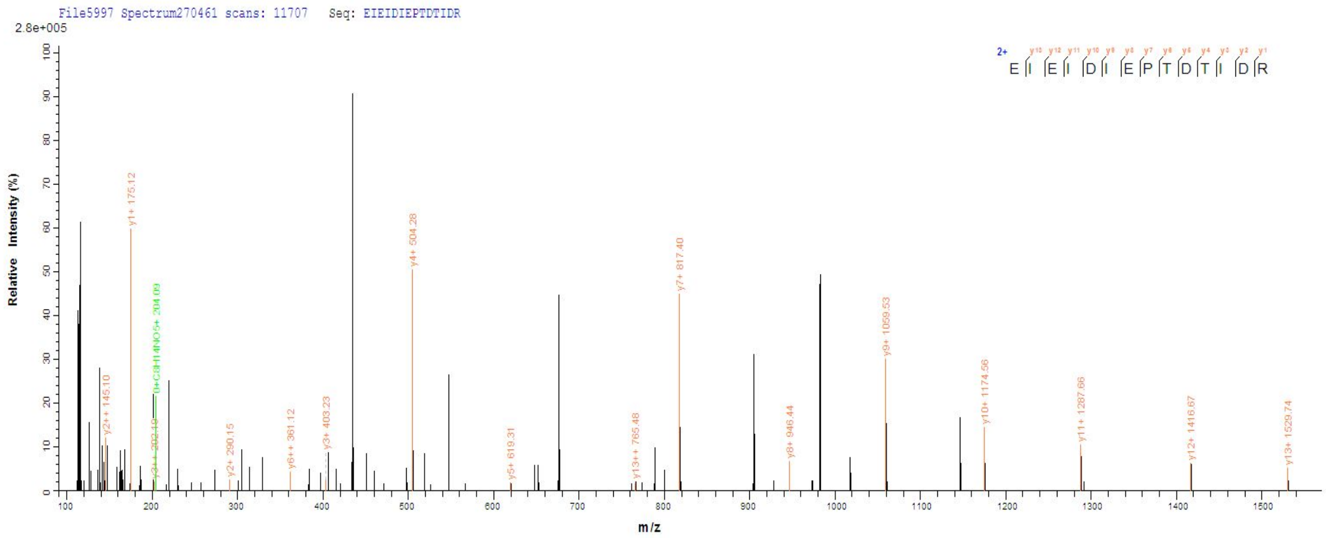

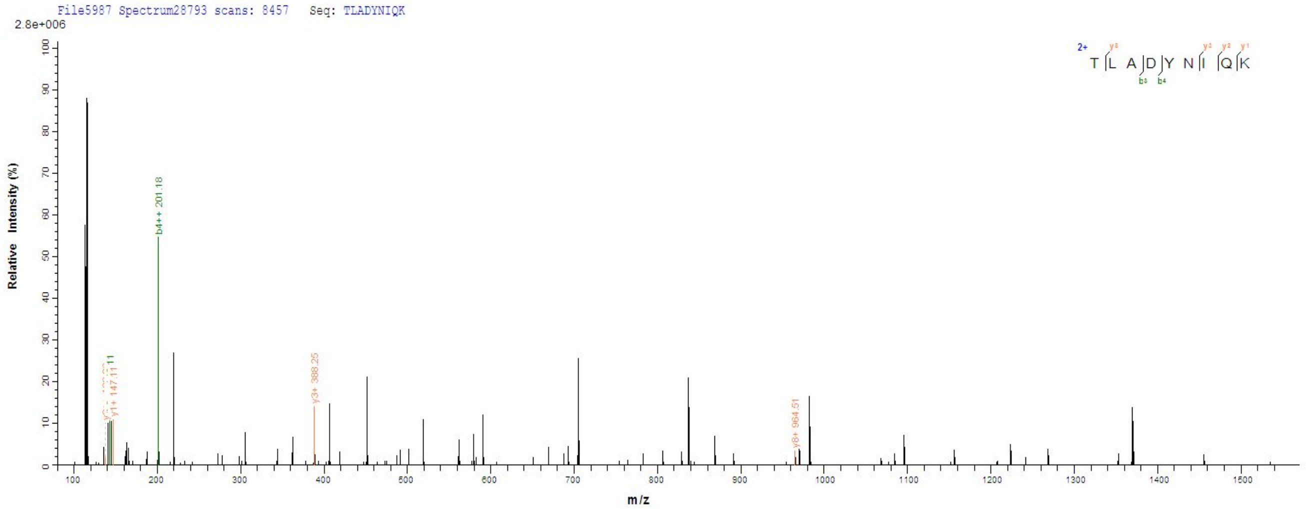

KKMQIFVKTLTGKTITLEVESSDTIDNVKAKIQDKEGIPPDQQRLIFAGKQLEDGRTLADYNIQKESTLHLVLRLRGGTM
IKVKTLTGKEIEIDIEPTDTIDRIKERVEEKEGIPPVQQRLIYAGKQLADDKTARDYNIEGGSVLHLVLALRGGLDKEGIPP
DQQRLIFAGKQLEDGRTLADYNIQKESTLHLVLRLRGGTMIKVKTLTGKEIEIDIEPTDTIDRIKERVEEKEGIPPVQQRLI
YAGKQLADDKTARDYNIEGGSVLHLVLALRGGCL

31 Garb_22303 gi||95116512 ubiquitin activating enzyme

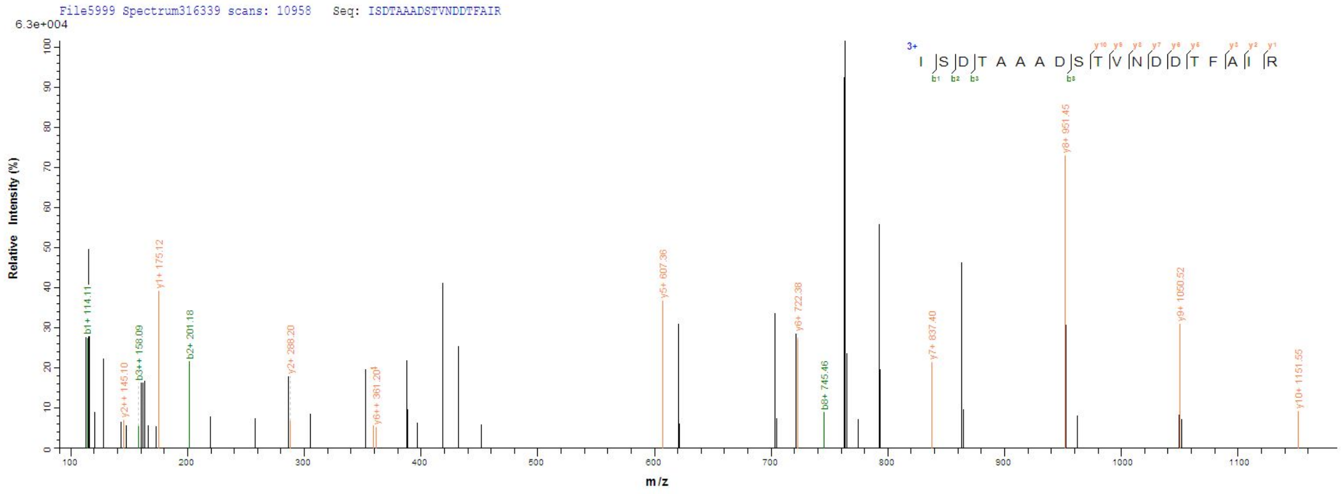

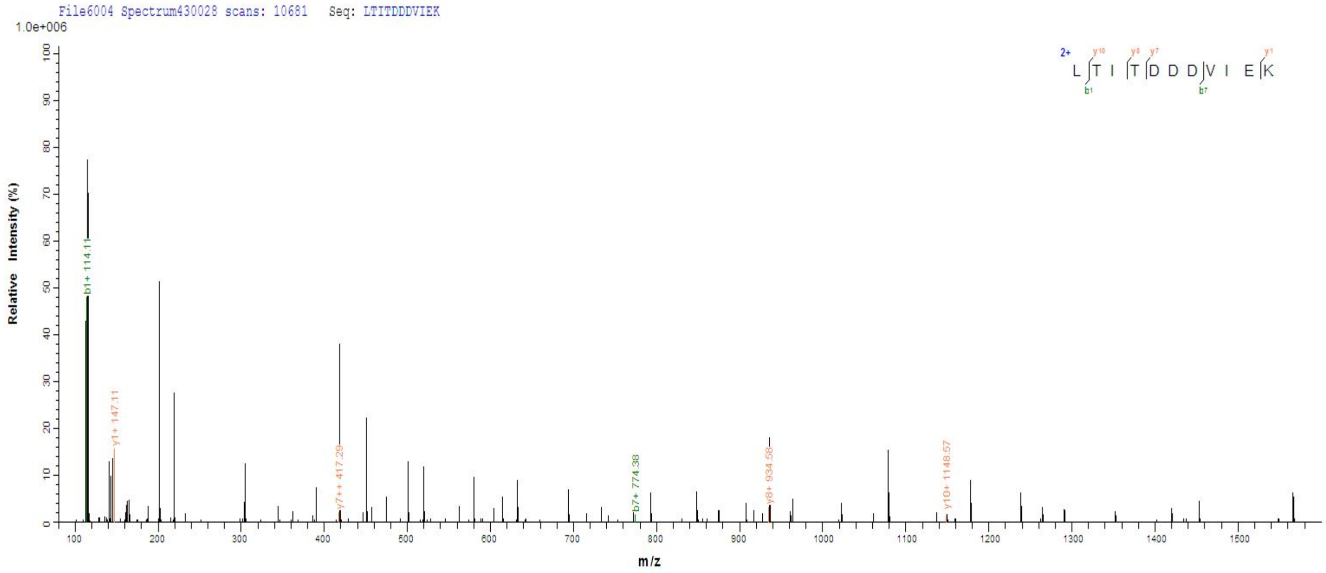

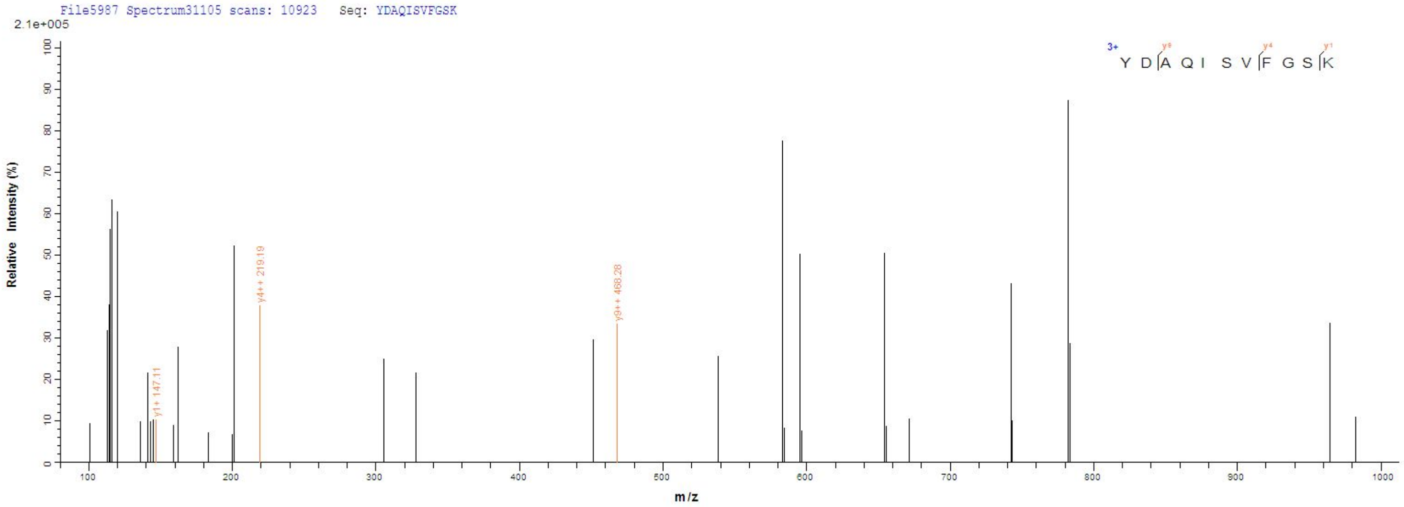

MLPRKREGQGDVVEGETENNNNSNTIKYVSVTSPVKKHRISDTAAADSTVNDDTFAIRNSSGSSSVVEPAMAPGDSNH
NDIDEDLHSRQLAVYGRETMRRLFASNVLVSGMQGLGAEIAKNLILAGVKSVTLHDEGVVELWDMSSNFVFSEDDVG
KNRALASVQKLQELNNAVVISTLTTKLTKEQLSNFQAVVFTDISLEKAIEFNDYCHNHQPPISFIKSEVRGLFGTVFCDFG
PEFTVFDVDGEEPHTGIIASISNDNPALVSCVDDERLEFQDGDLVVFSEVHGITELNDGKPRKIKSARPYSFTLEEDTIAF
GTYVKGGIVTQVKQPKMLNFKPLREALKEPGDFLLSDFAKFDRPLLLHIAFQALDKFISDFGRFPVAGSEEDAQKLASI
AANINECLGEGKVEDINPKLLRQFAFGAKAVLNPMAAMFGGIVGQEVVKACSGKFHPLFQFFYFDSVESLPAEPLDPS
DFKPLNSRYDAQISVFGSKLQKKLEDAKVFMVGSGALGCEFLKNLALMGVSCGSQGKLTITDDDVIEKSNLSRQFLFR
DWNIGQAKSTVAASAAASINPRLKIEALQNRVGPETEGVFNDTFWEKLTVVINALDNVNARLYVDQRCLYFQKPLLES
GTLGAKCNTQMVIPHLTENYGASRDPPEKQAPMCTVHSFPHNIDHCLTWARSEFEGLLEKTPAEVNAYLSNPVEYATS
MKNAGDAQAKDNLERVLECLDREKCETFQDCITWARLRFEDYFVNRVK

32 Garb_00461 gi|356560787 PREDICTED: ubiquitin-conjugating enzyme E2 5-like

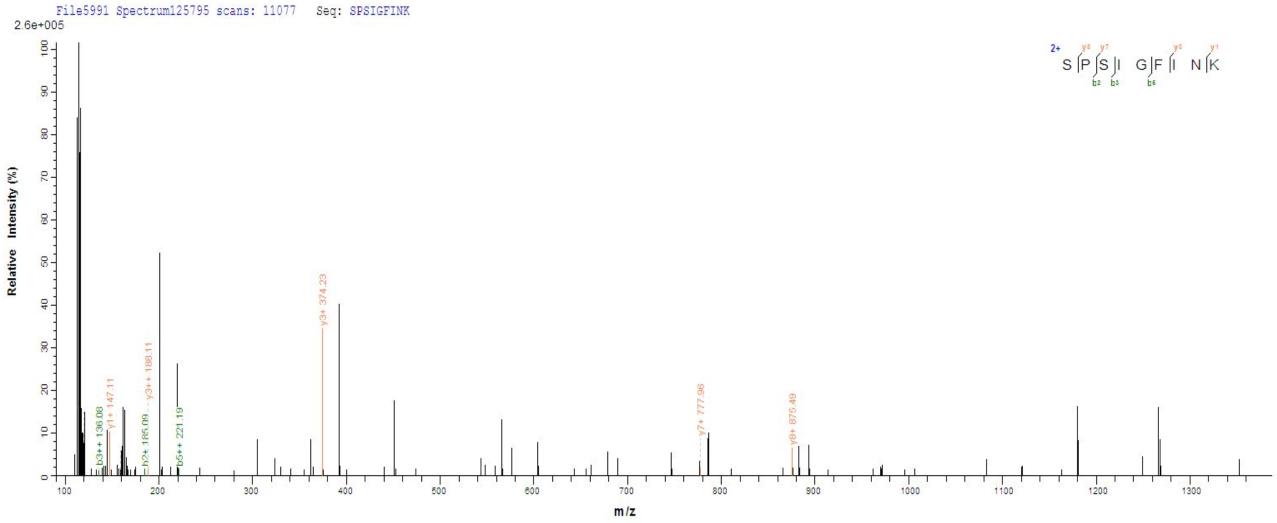

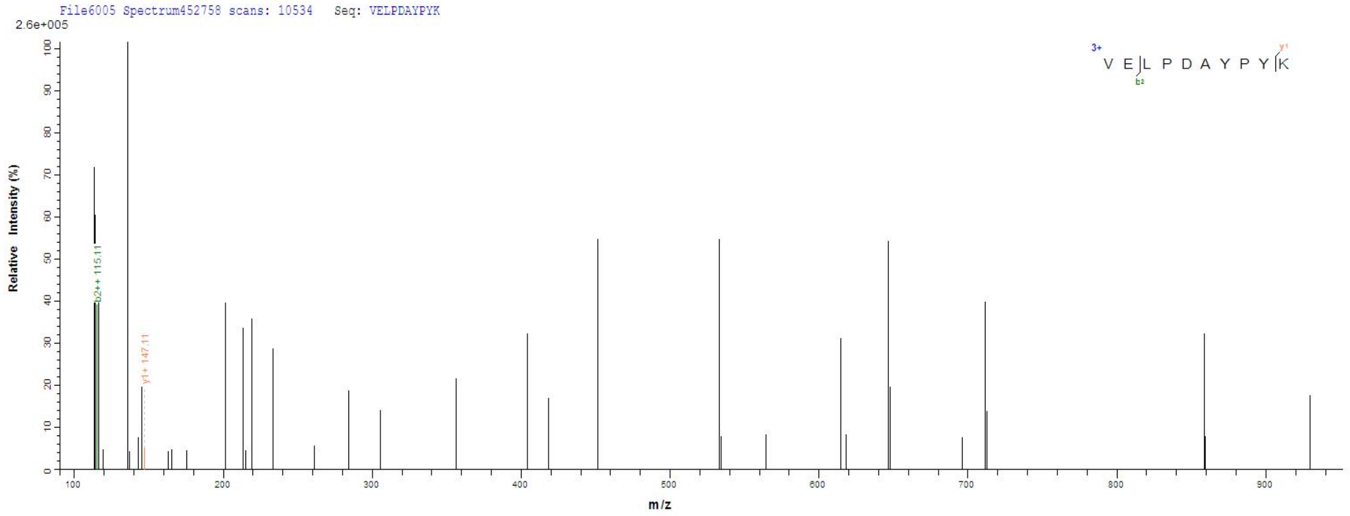

MSSPSKRREMDLMKLMMSDYKVEMINDGMQEFYVEFNGPKDSPYHGGVWKIRVELPDAYPYKSPSIGFINKIYHPNVDEMSGSVCLDVINQTWSPMFDLVNVFEVFLPQLLLYPNPSDPLNGEAAALMMRDRAAYEQRVKEYCEKYAKPEDIGAKAEEKSSDEELSEDEYAASDDEEIAGKPDP

33 cotton_GLEAN_10028977 gi|117949833 T-complex protein 1 subunit gamma

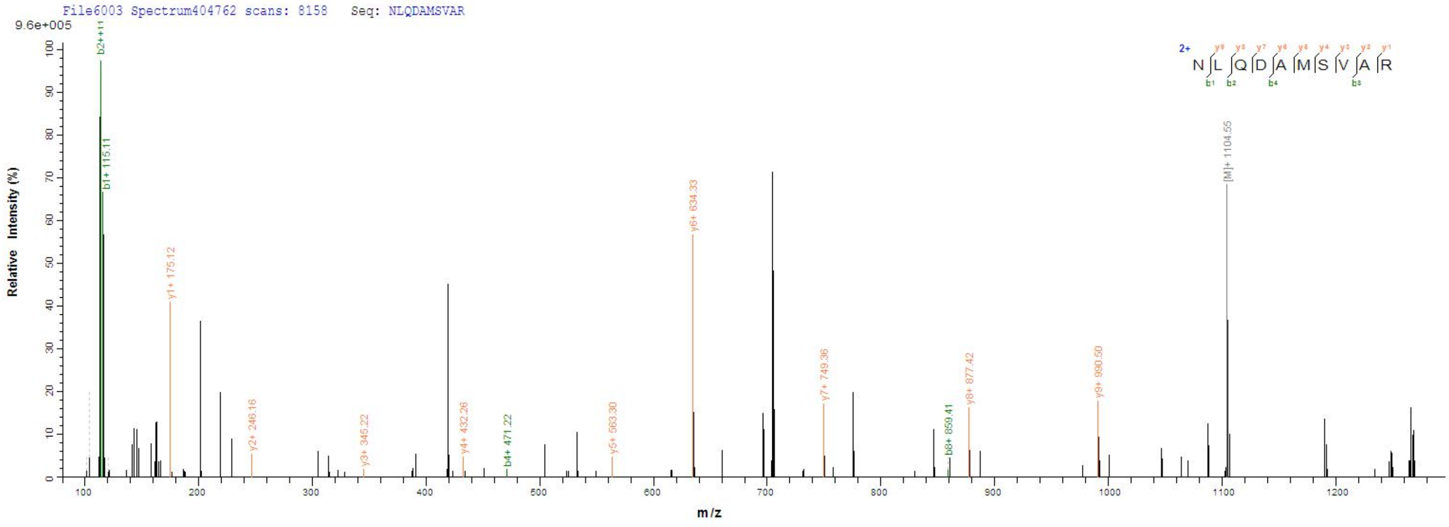

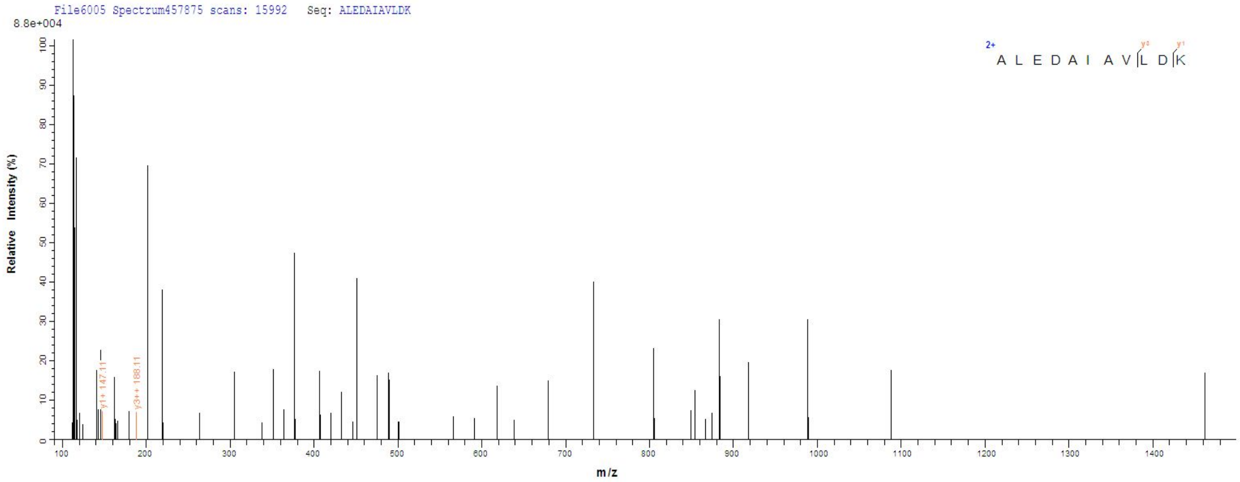

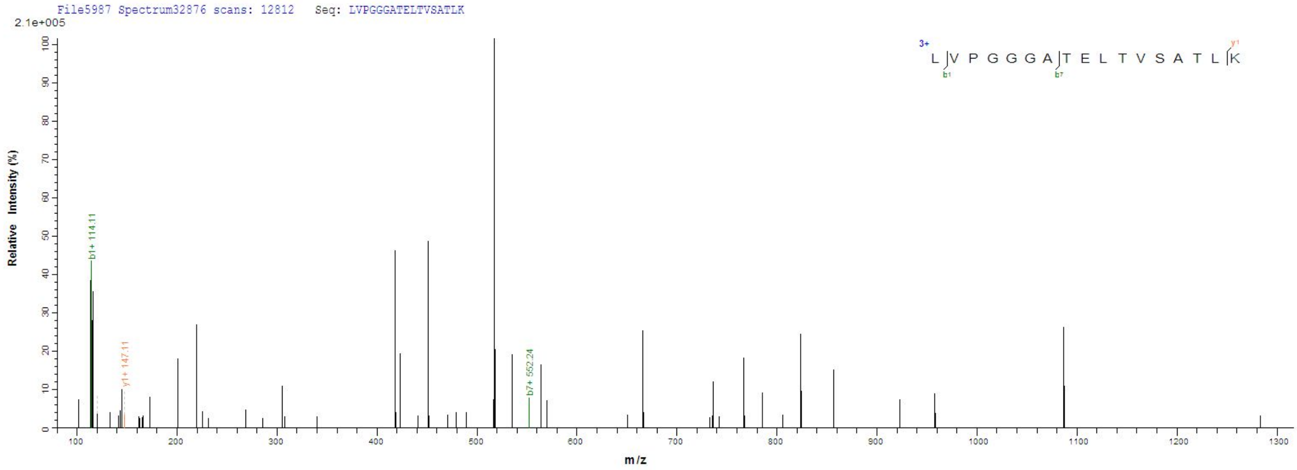

MHAPVLVLKDSLKRESGTKVHHANIQASKAVADIIRTTLGPRSMLKMLLDASGAMKFLYGFGFYQSMIELSRTQDEEV
GDGTTSVIVLAGEMLHVAEAFIEKNYHPTVICRAYNKALEDAIAVLDKIAMSIDVKDRATMLGLVKSCIGTKFTSQFGD
LIADLAIDATQTVGVDLGQGLREVDIKKYIKVEKVPGGQLEDSKVLKGVMINKDVVAPGKMKRKIVNPRIILLDCPLE
YKKGENQTNAELVKEEDWEVLLKMEEEYIESLCLQILKFKPDLVVTEKGLSDLACHYLSKAGVSAIRRVRKTDNNRIA
KASGAVIVNRPDELQESDVGTGAGLFEVKKIGDEFFAFIVDCKDPKACTVLLRGASKDLLNEVERNLQDAMSVARNIIK
NPKLVPGGGATELTVSATLKQKSSSVEGIEKWPYEAAALAFEAIPRTLAQNCGVNVIRTMTALQGKHANGENAWIGID
GNTGAIADMKERKIWDAYNVKAQTFKTAIEAACMLLRIDDIVSGIKKKQAPGAGQG

34 Garb_02841 gi|75115360 Protein disulfide isomerase-like 1-6

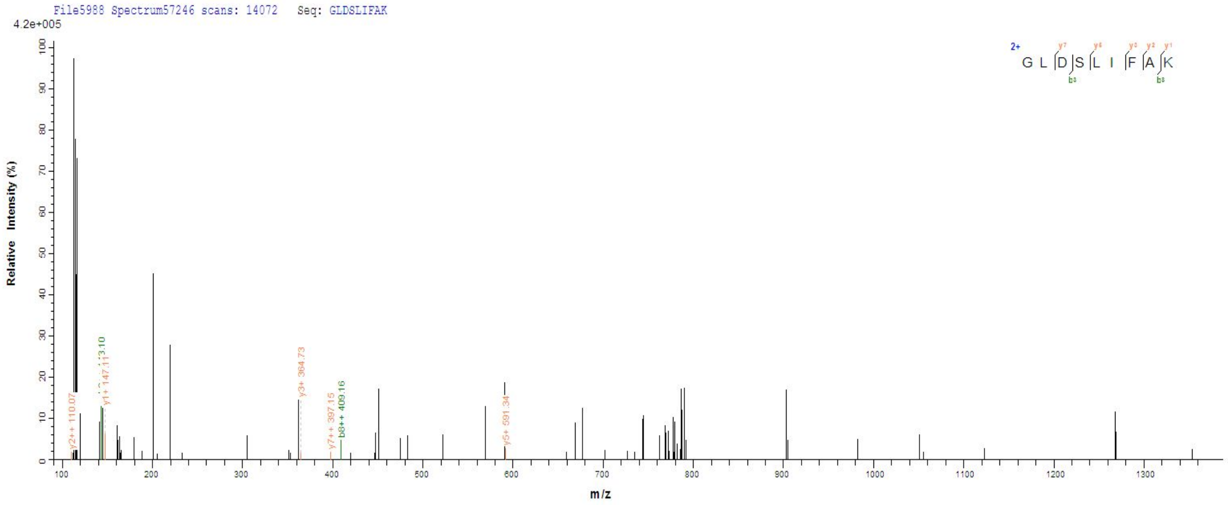

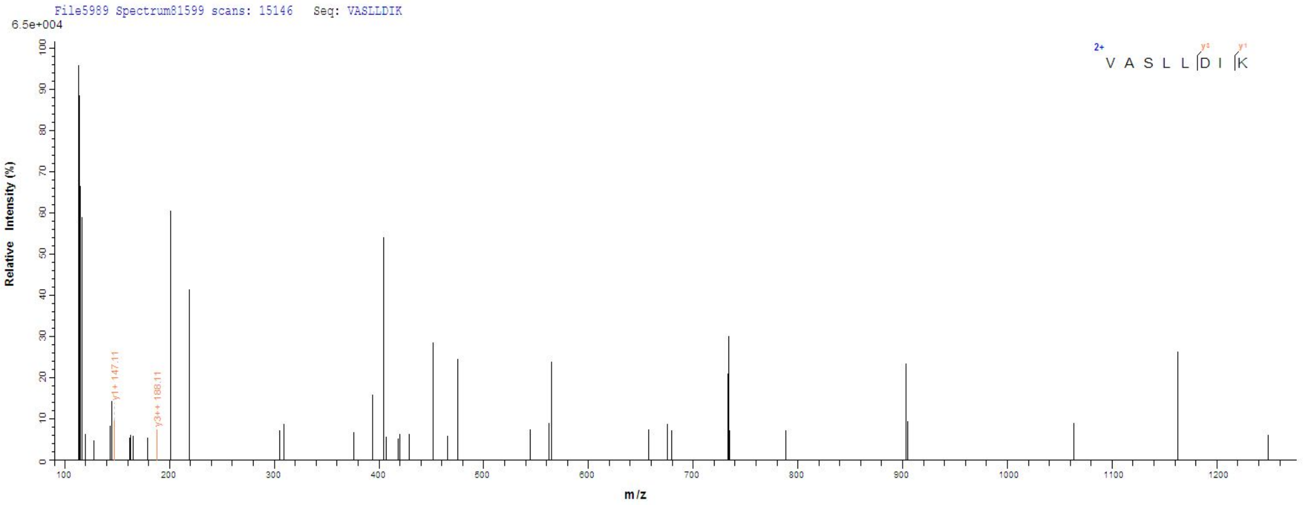

MLFSKNPTSRFLLLTLIFVLLFFFSININASDPTNSEDDSDDLEQLLALDEQEDQLPEDQDQESSSRFSEAEVLSKAQRIV LELNSDNSKRVIDENEFVLLLGYAPWCVRSAELMPQFAEAATSLKELGSPVLMAKLDAERYPKVASLLDIKGFPTLLLF VNRTSQAYTGGFSAEEIVIWARKKTGVPVIRINTVTEAEDFLKKHHMFVIGLFEKFEGSDYKAFIKAAMSPTPSNIEEFC SGLLHGSSSTYFKSQPIPDNNNASVLAVVGKTFDDLVLNSPKNVLLEVYTPWCINCETTSKQIEKLAKHFKGLDSLIFAK IDASANEHPKLQVDDYPSLFLYKAGDKDNPIKLSTKSGSKELAAFINKQVRPKDQAAKDEL

35 cotton_GLEAN_10016664 gi|255541132 structural constituent of nuclear pore, putative

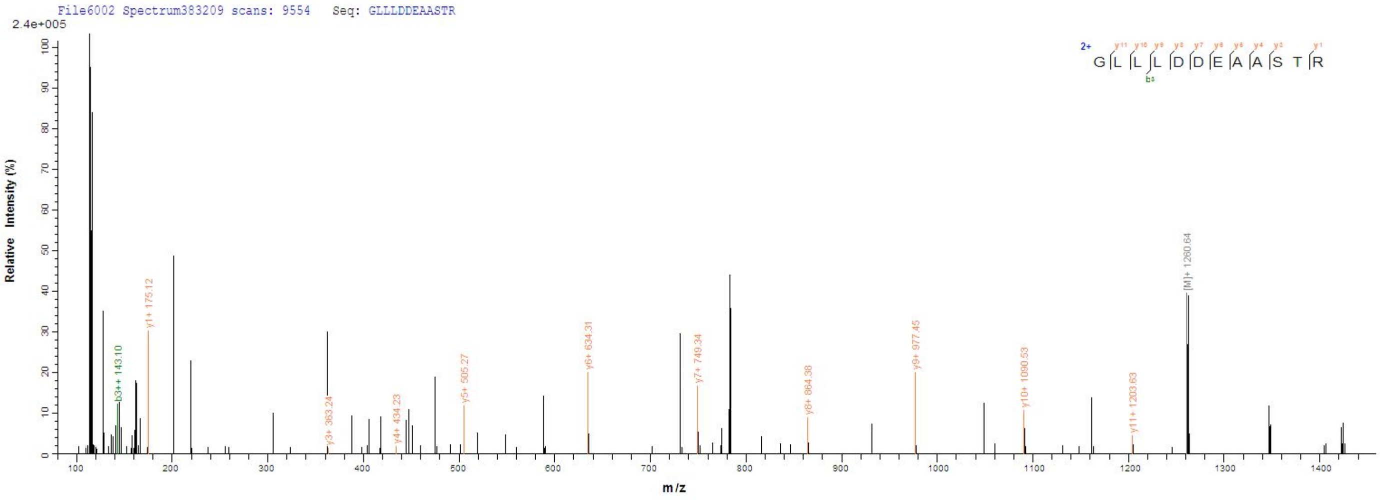

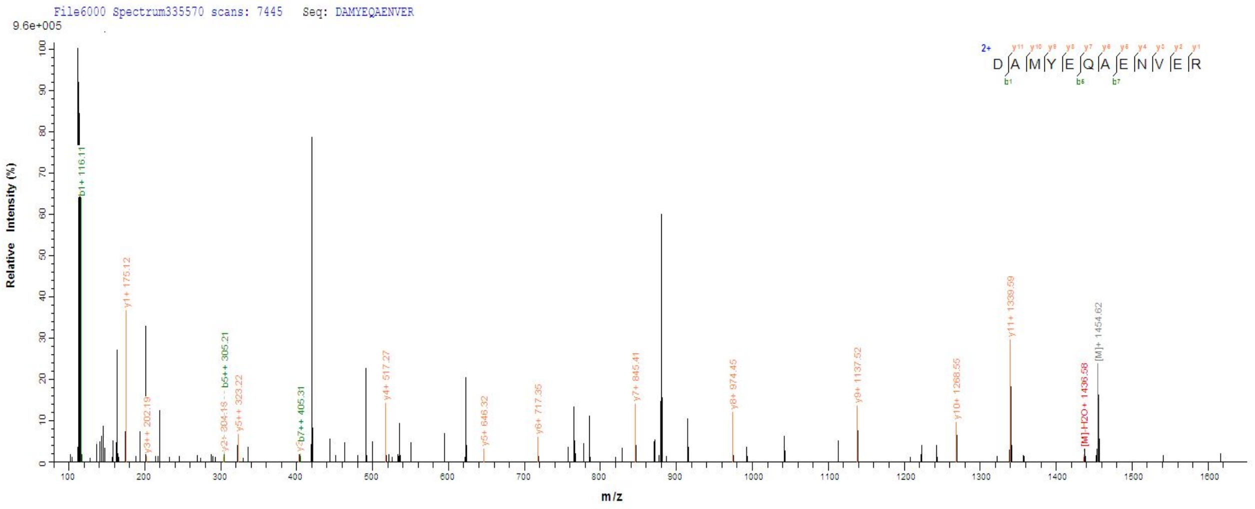

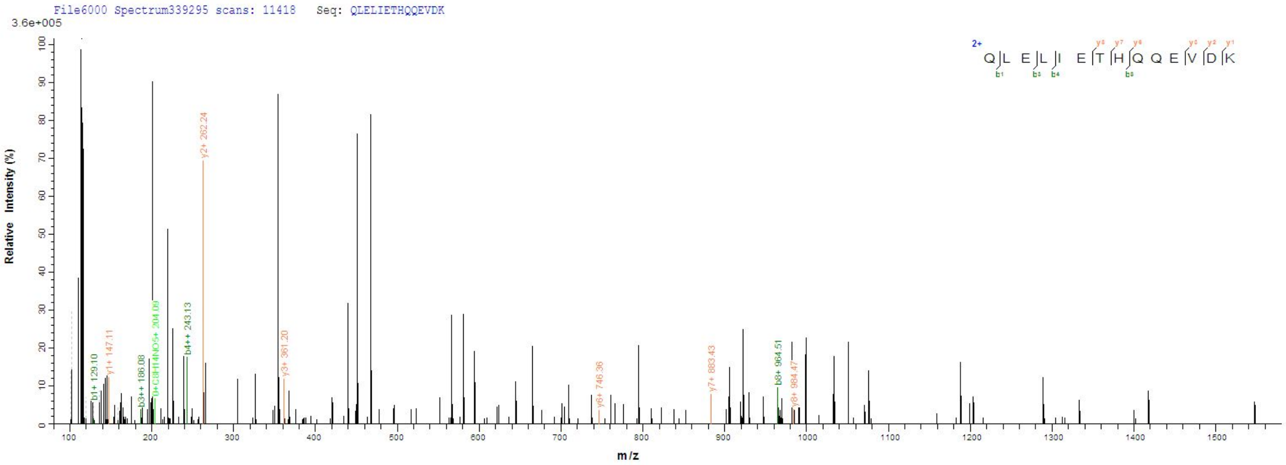

ADNPFNPSGGFGTSSSLFGASSGSSPFSSSSATGPFSFASSSSSASTATTVTTPSFSGLFSSSSASNSATTSPFGAAGFSFSVGSPFSSSSASSNTTAASTAAASTSSSTSGFSFAPSSSFGFGNPASSSSPASVFSEKVGASSSPFGASSGSTLFSSSPAPATSSSFGSSSSSSAAAPAPAPSFSSFISSSSASNSTTTSPFSSSTGFPFLGGSSFGKSTESSTTTATFTTVPSLTAASSSSSSSGFSFSLPSSASQPTFGYGNAASSSSSSSAPTVPGTFGTSSSPFGASSGSPLFSSSQAPATTTTTTAAMSSFGSLCSPSTATATTPSFPSLSSSSTASSTGFSFGGSSPFLKSTASSTPTSTYTTAPSLAAASSSSSSSFSFASPSASQPAYGFGNAASSSTPAPTLSGSAVSKPTSLSFGTSSAPLFSTVTTTTASTPAASTTTASTPAFPAFNLSSSSSSATASSAAPASSAATSAAVSSLTGFGVTNVAATSGSSGSFSGFSLSTKPSTPASSSQAQAIATAPVFSLTASGSSASTTSTSISTSAQTSSALVVASSSGTSSSATAAVSTTPKLPSEITGKTVEEIIKEWNAELQERTGKFRKQASAIAEWDRRILQNRDVLLRLEIEVAKVVETQASLERQLELIETHQQEVDKALQSMEEEAERIYKDERGLLLDDEAASTRDAMYEQAENVERELEHMTEQIKSIIETVNSSQGGELEALDGMTPLDVVVKILNNQLTSLMWIDEKAEEFSGRIRKLAAQGNAADGELIAAPKYWMS

36 Garb_13672 gi|82581521 Proteasome subunit beta type-4

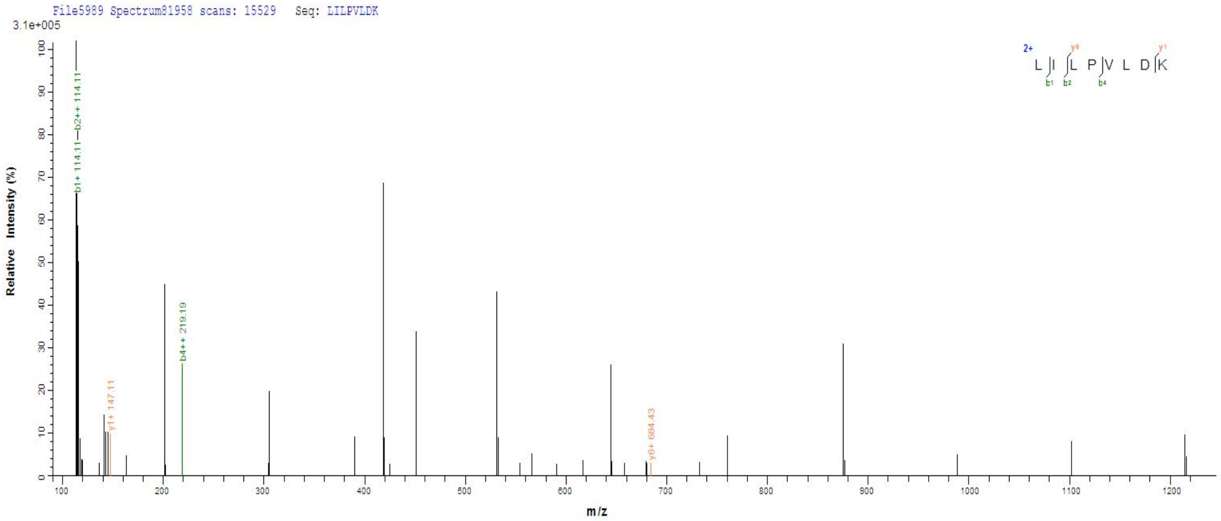

MEGSVFTPSLEGMKHIKSPEGEMLNKPFLDVCKLILPVLDKFGSAMSLVKSDVGGNISRLEKKYETDPAKYNHLYSMVKEEVDCKTATDSSSCTNGLLWLTRAMDFLVELFRNLLAHPDWTMTESCTDSYGKTLKKFHGWIASSAFTVALKLAPDRKKFMEVISGTGDVNADMEKFCSTFPPFLEENHKYLVRL

37 Garb_21548gi|82581521 Proteasome subunit beta type-4

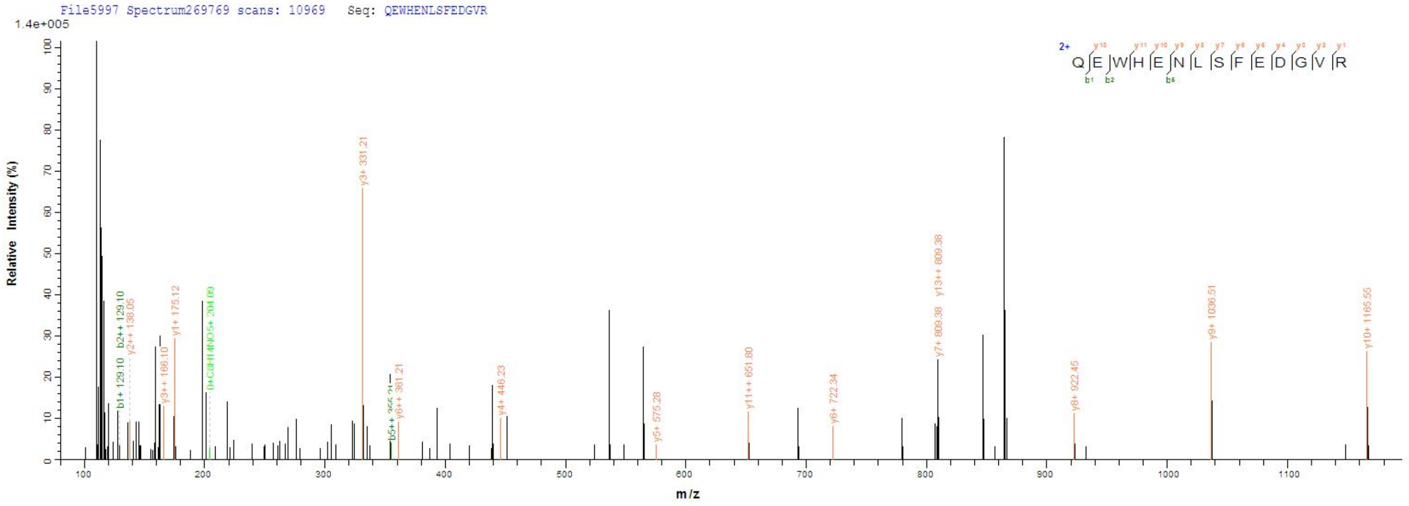

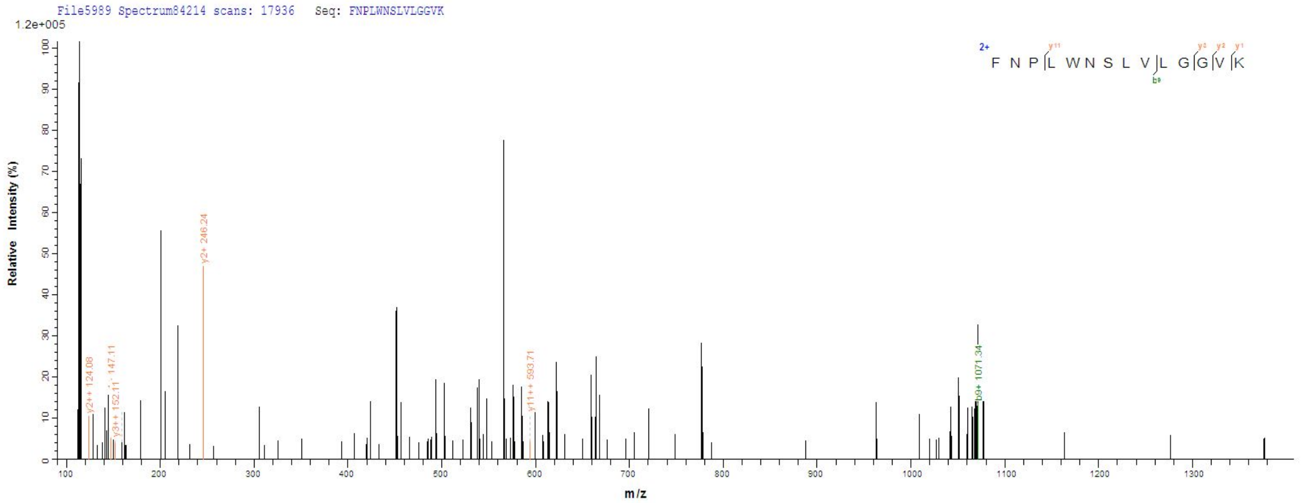

ASSSNSSSGLRSSFKEFFHSDVLCERFGGNFSSKQPISYAALHHVSYHYDVNFGEWVKSSHPTTNDDDNVEGTFECILV
PEHVPPVASFSQAAQPSSEINVTILNALHYLRNDVRGLRDKVRSHKDKSAHCPKCDLGSSRGSHVVVSSFALFLYPYVT
GTSVVALKYKDGILMAADMGGSYGSTLRYKSVERMKPIGKHSLLGASGEISDFQEILRYLDELILYDNMWDDGNSLGP
KEVHNYLTRVMYNRRNKFNPLWNSLVLGGVKNGQKYLGTVSMIGVNFEDNHVATGFGNHLARPILRQEWHENLSFE
DGVRLLEKCMRVLLYRDRSAVNKLQIAKITEDGVTISQPYSLKTYWEFSAFENPAQGAIGSW

38 Garb_12714 gi|211906494 heat shock protein 70

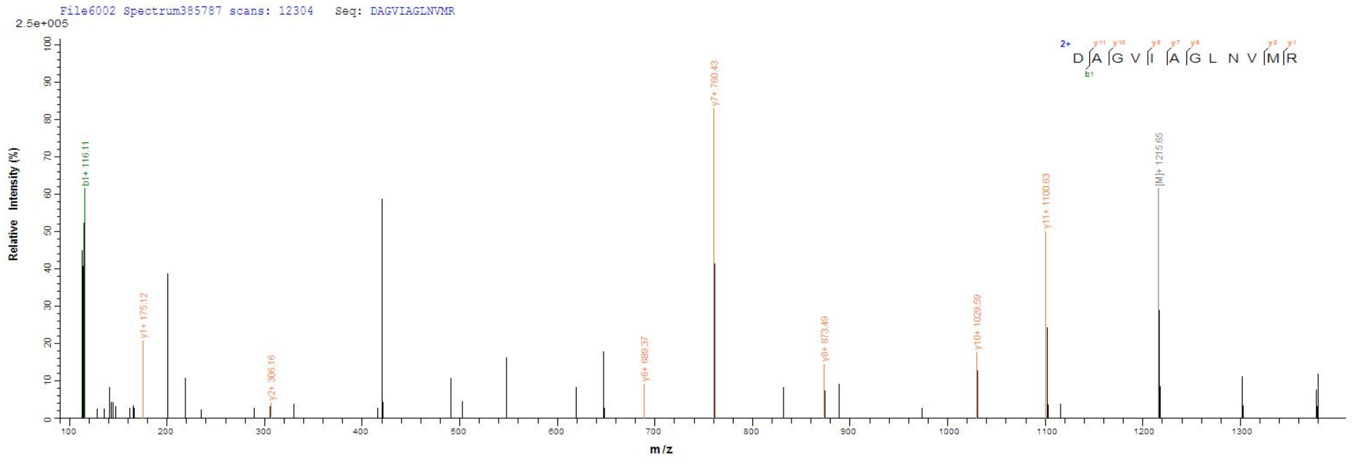

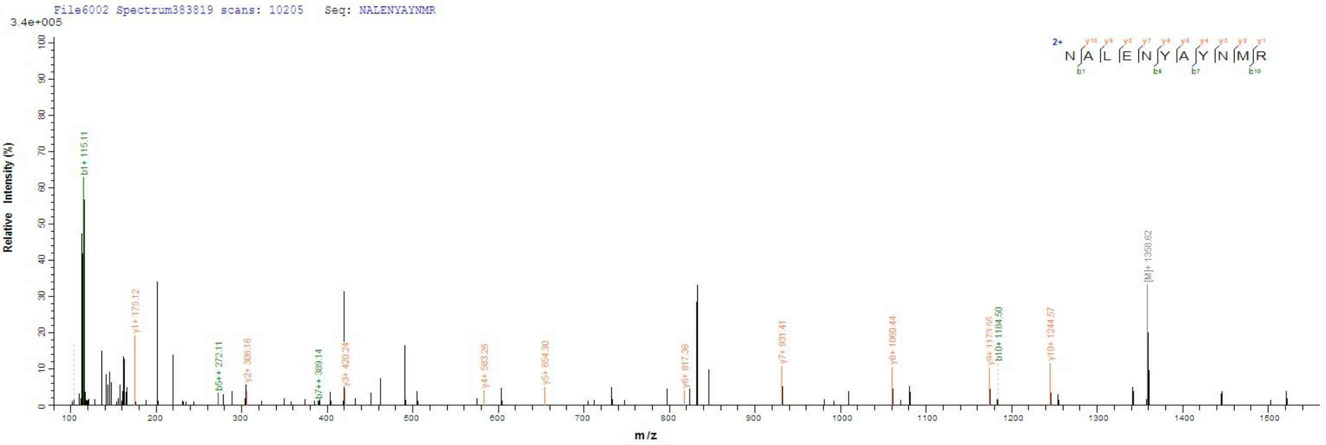

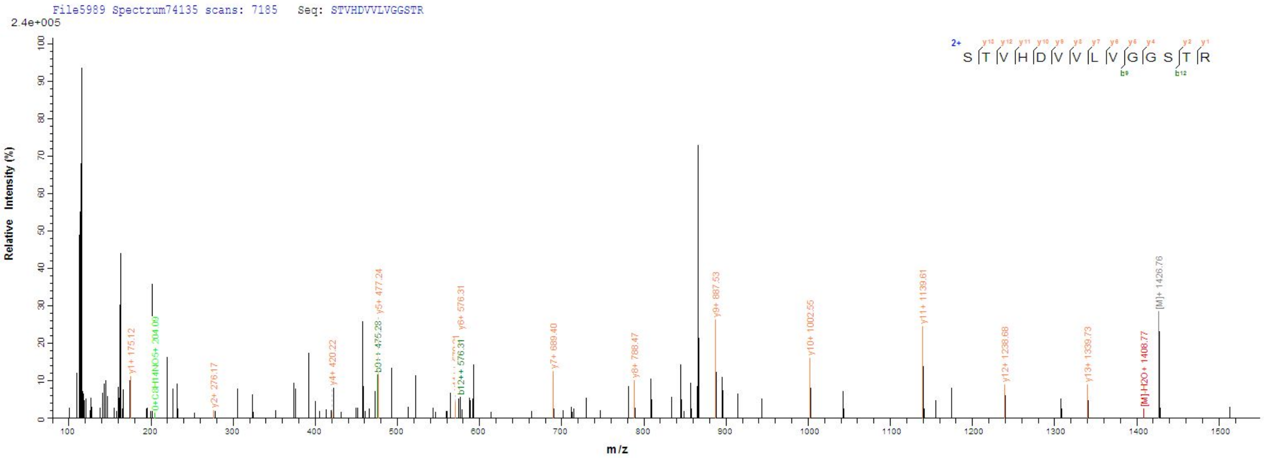

LIGRRFSDASVQSDTKLWPFKVIAGPGDKPMICVAYKGEEKQFAAEEISSMVLIKMREIAEAYLGSTIKNAVVTVPAYFN DSQRQATKDAGVIAGLNVMRIINEPTAAAIAYGLDKKATSVGEKNVLIFDLGGGTFDVSLLTIEEGIFEVKATAGDTHLG GEDFDNRMVNHFVQEFKRKNKKDISGNPRALRRLRTACERAKRTLSSTAQTTIEIDSLYEGIDFYSTITRARFEELNMDL FRKCMEPVEKCLRDAKMDKSTVHDVVLVGGSTRIPKVQQLLQDFFNGKELCKSINPDEAVAYGAAVQAAILSGEGNE KVQDLLLLDVTPLSLGLETAGGVMTVLIPRNTTIPTKKEQVFSTYSDNQPGVLIQVYEGERTRTRDNNLLGKFELSGIPP APRGVPQITVCFDIDANGILNVSAEDKTTGQKNKITITNDKGRLSKEEIEKMVQEAEKYKSEDEEHKKKVEAKNALEN YAYNMRNTVKDEKIGAKLPAADKKKIESDAIEQAIQWLDSNQLAEADEFEDKMKELESICNPIIAKMYQGAGGDMGG GMDEDVPAGGSGAGPKIEEVD

39 Garb_21426 gi|211906504 heat shock protein 70

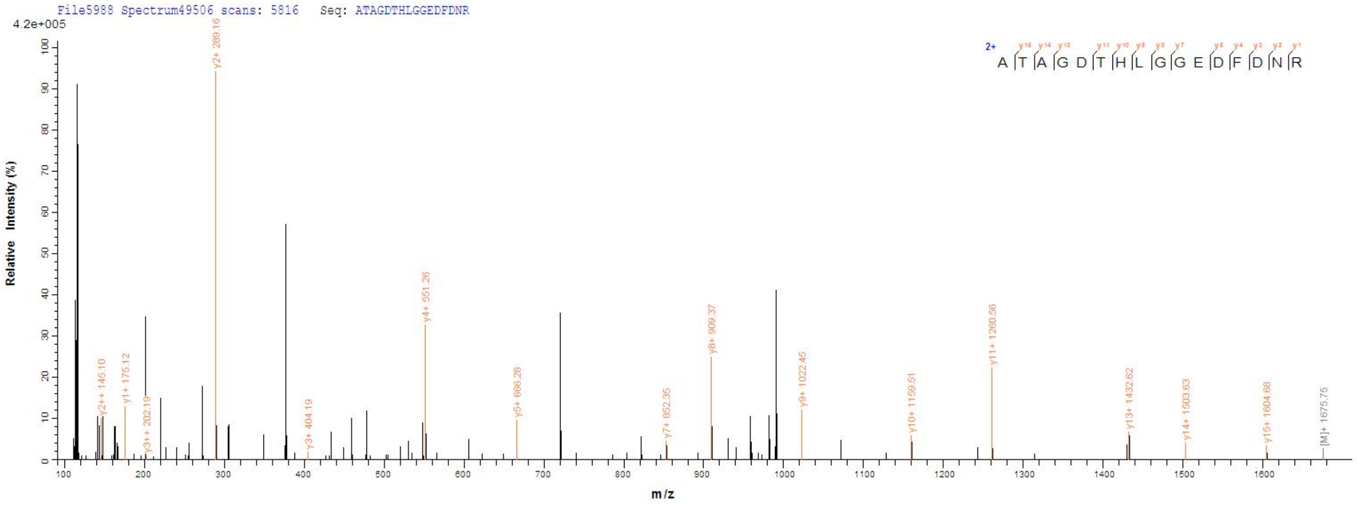

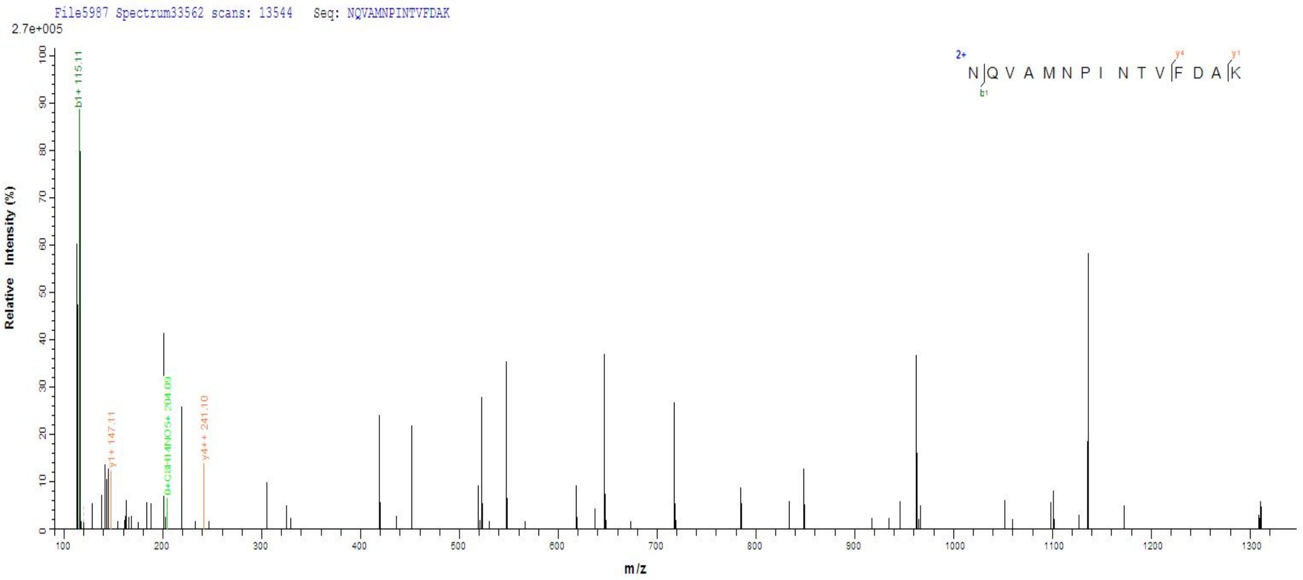

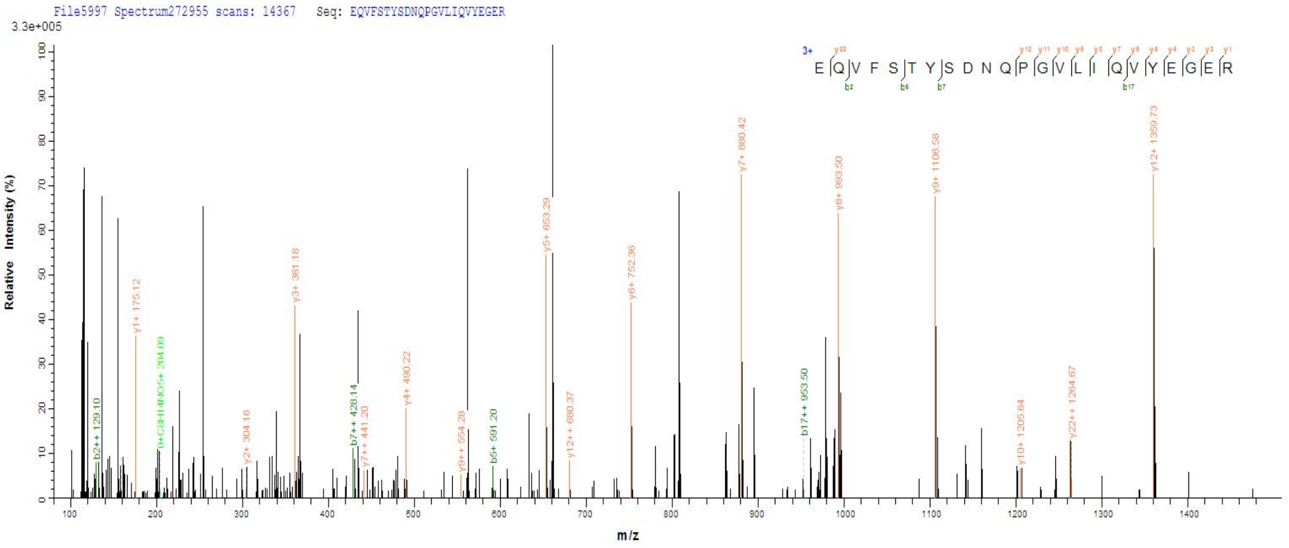

MAGKGEGPAIGIDLGTTYSCVGVWQHDRVEIIANDQGNRTTPSYVAFTDSERLIGDAAKNQVAMNPINTVFDAKRLIG
RRYSDSSVQSDMKLWPFKVIAGPGDKPMIVVTYKGEEKQFAAEEISSMVLIKMREIAEAYLGATIKNAVVTVPAYFNDS
QRQATKDAGVIAGLNVMRIINEPTAAAIAYGLDKKASSVGEKNVLIFDLGGGTFDVSLLTIEEGIFEVKATAGDTHLGGE
DFDNRMVNHFVQEFKRKNKKDISGNPRALRRLRTACERAKRTLSSTAQTTIEIDSLYEGIDFYTTITRARFEELNMDLFR
KCMEPVEKCLRDAKMDKSSVHDVVLVGGSTRIPKVQQLLQDFFNGKELCKSINPDEAVAYGAAVQAAILSGEGNEKV
QDLLLLDVTPLSLGLETAGGVMTVLIPRNTTIPTKKEQVFSTYSDNQPGVLIQVYEGERARTRDNNLLGKFELSGIPPAP
RGVPQITVCFDIDANGILNVSAEDKTTGQKNKITITNDKGRLSKEEIEKMVQEAEKYKSEDEEHKKKVEAKNALENYA
YNMRNTVKDEKIGSKLDPADKKKIEDAIDGAIQWLDGNQLAEADEFEDKMKELESICNPIIAKMYQGAGADMGGGM
DEDAPPTGGSSAGPKIEEVD

40 cotton_GLEAN_10025441 gi|289064666 S-adenosylmethionine synthase-like protein

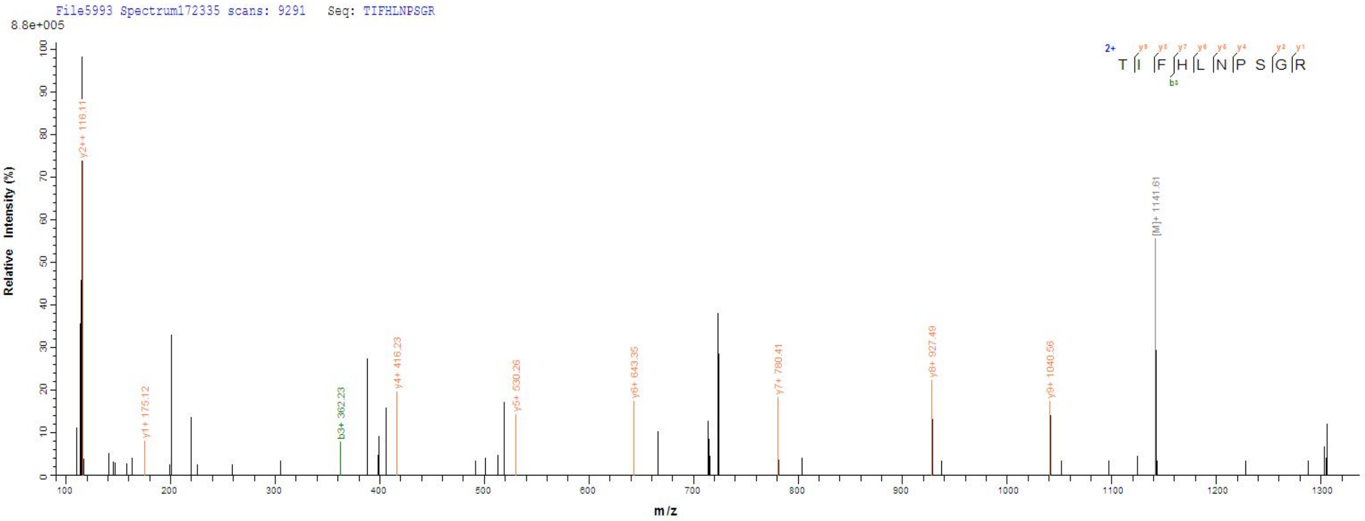

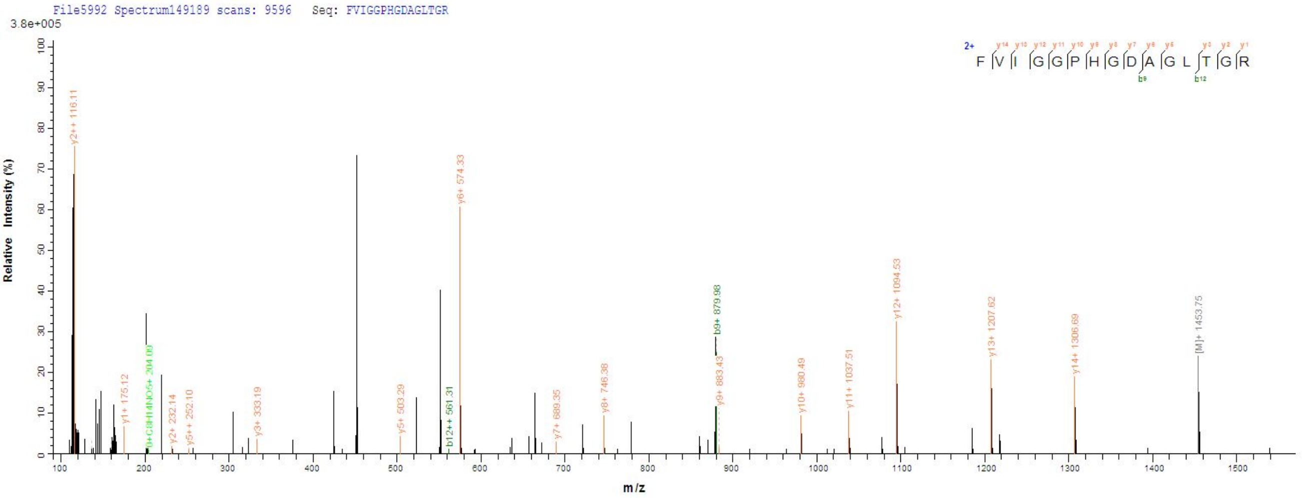

METFLFTSESVNEGHPDKLCDQISDAVLDACLAQDPDSKVACETCTKTNMVMVFGEITTKANVDYEKIVRDTCRSIGFVSDDVGLDADNCKVLVNIEQQSPDIAQGVHGHFTKRPEEIGAGDQGHMFGYATDETPELMPLSHVLATKLGARLTEVRKNGTCPWLRPDGKTQVTVEYYNDKGAMVPVRVHTVLISTQHDETVTNDEIAADLKEHVIKPVIPEKYLDEKTIFHLNPSGRFVIGGPHGDAGLTGRKIIIDTYGGWGAHGGGAFSGKDPTKVDRSGAYIVRQAAKSIVANGLARRCIVQVSYAIGVPEPLSVFVDSYGTGKIPDKEILQIVKENFDFRPGMITINLDLKRGGNGRFLKTAAYGHFGRDDPDFTWEVVKPLKWEKPQS

41 cotton_GLEAN_10013973 gi|255543963 Aspartic proteinase nepenthesin-1 precursor, putative

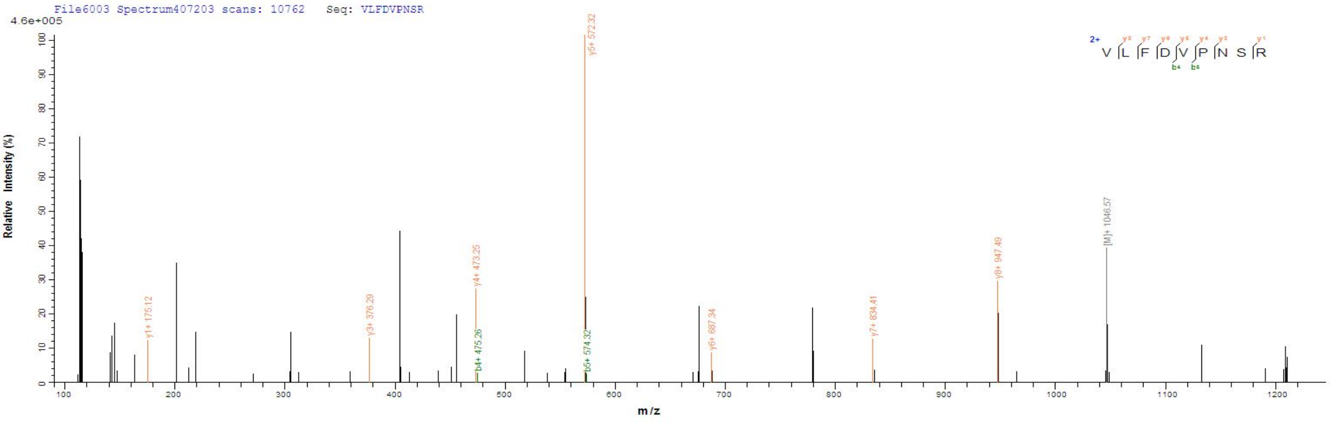

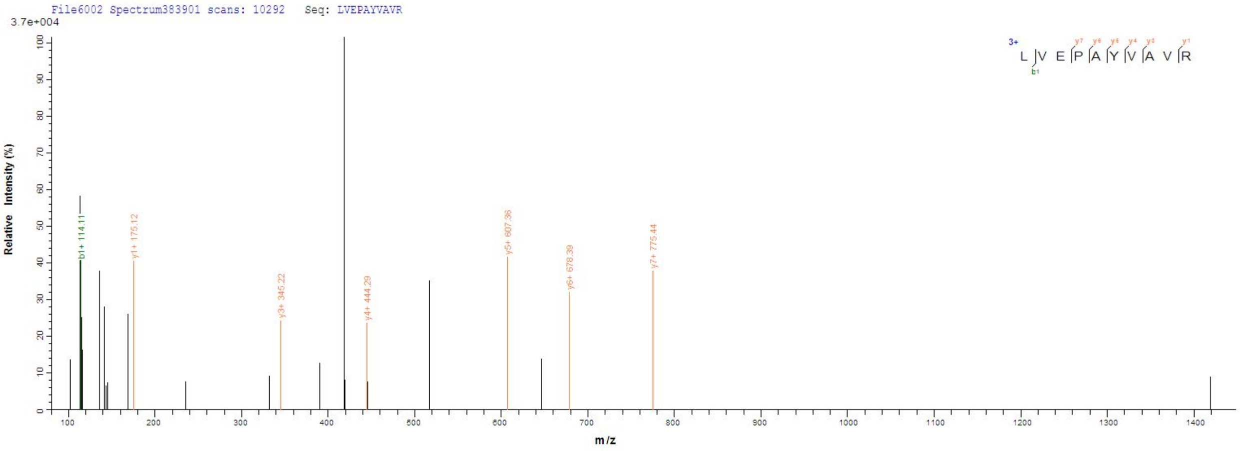

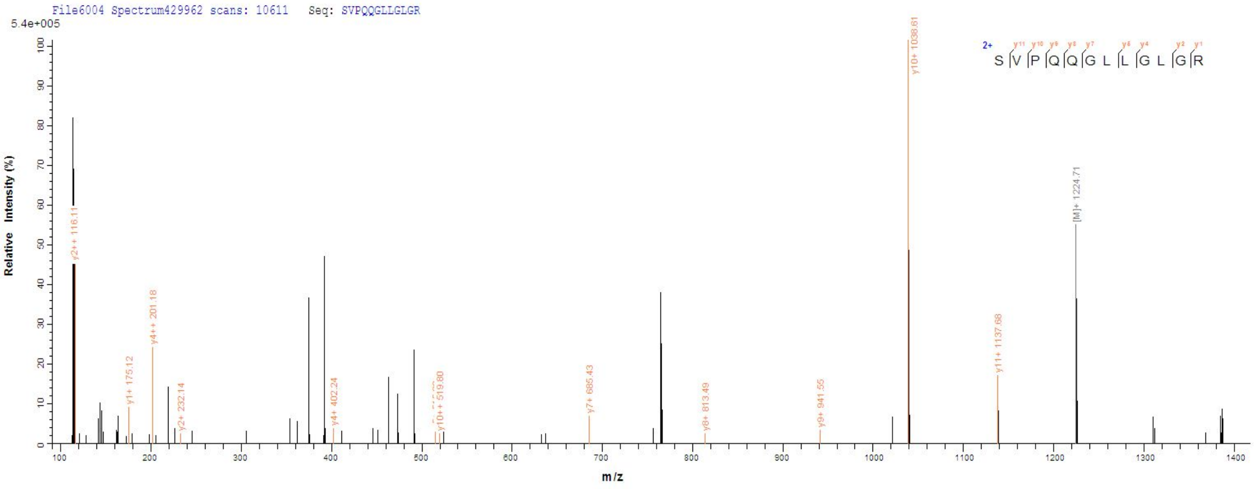

MKTQTFCLVFLFISVAQGLHSKCDTQDHGSTLQVFHIYSQCSPFKPSKPLSWEEDMLSMLAKDQARLQYLSSLVAGKS
VVPVASGRQIVQSPTYIVRAKIGTPPQTMLMAMDTSNDAAWIPCNGCIGCSSKVFNTAKSTTFNTLGCHAAQCKQVPN
PTCGGSACVFNMTYGSSSIAGNLSRDTVVLATDPIPSYTFGCLQKTTGKSVPQQGLLGLGRGPLSLLSQTQHLYKSTFS
YCLPSFRSPNFSGSLRLGPKGQPIRIKYTQLLKNPRRPSLYFVNLIGIRVGRRVVDIPPKALAFNPSTGAGTIFDSGTVFTR
LVEPAYVAVRNAFRRRVRVANVTSLGGFDTCYTVPIVAPTITFMFTGMNVTLPQENLLIHSTAGSITCLAMASAPDNVNS
VLNVIANMQQLNHRVLFDVPNSRLGVARERCT

42 Garb_35439 gi|229830633 L-idonate 5-dehydrogenase

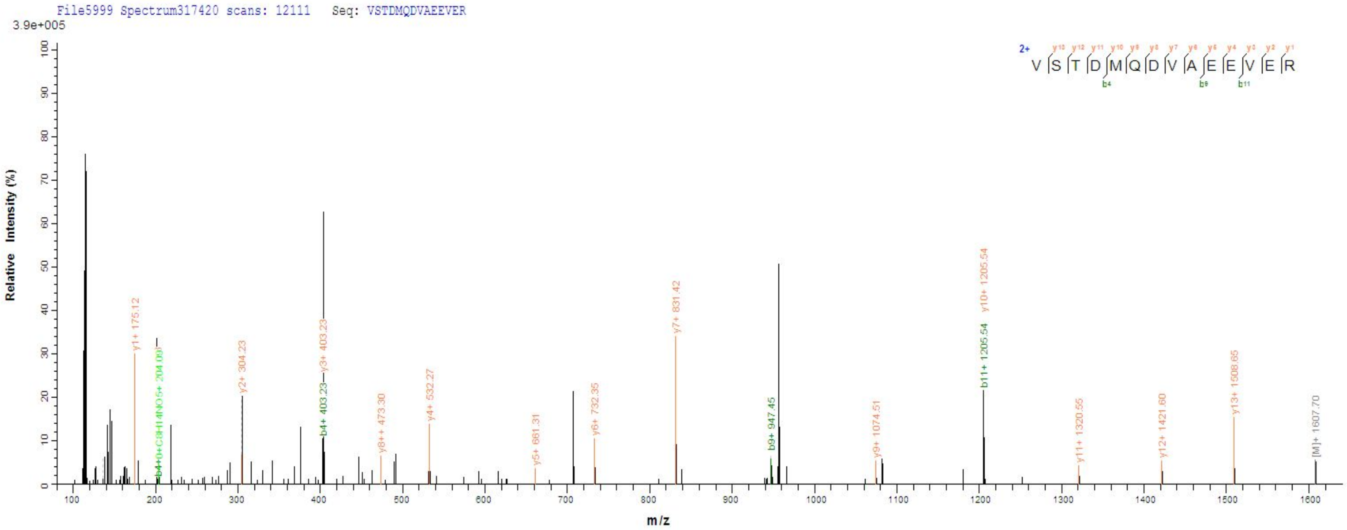

MGKGGKSHEETKSGEDENMAAWLVGLNTLKIQPFKLPPLGPHDARVRMKAVGICGSDVHYLKTMRCADFVVKEPMV
IGHECAGIIEEVGSEVKNLVPGDRVALEPGISCWRCDLCKDGRYNLCPEMKFFATPPVHGSLAHQVVHPADLCFKLPDN
VSLEEGAMCEPLSVGVHACRRANIGPETNVLVMGAGPIGLVTMMAARAFGAPRIVIVDVDDNRLSVAKNLGADGIVK
VSTDMQDVAEEVERICKAMGGGVDVSFDCAGFNKTMSTALSATRAGGKVCLVGMGHHEMTVPLTPAATREVDVIGIF
RYRNTWPLCIEFLRSGKIDVKPLITHRFGFSQKEVEEAFETSAGGGSAIKVMFNL

43 cotton_GLEAN_10014464 gi|308743337 asparagine synthetase 1

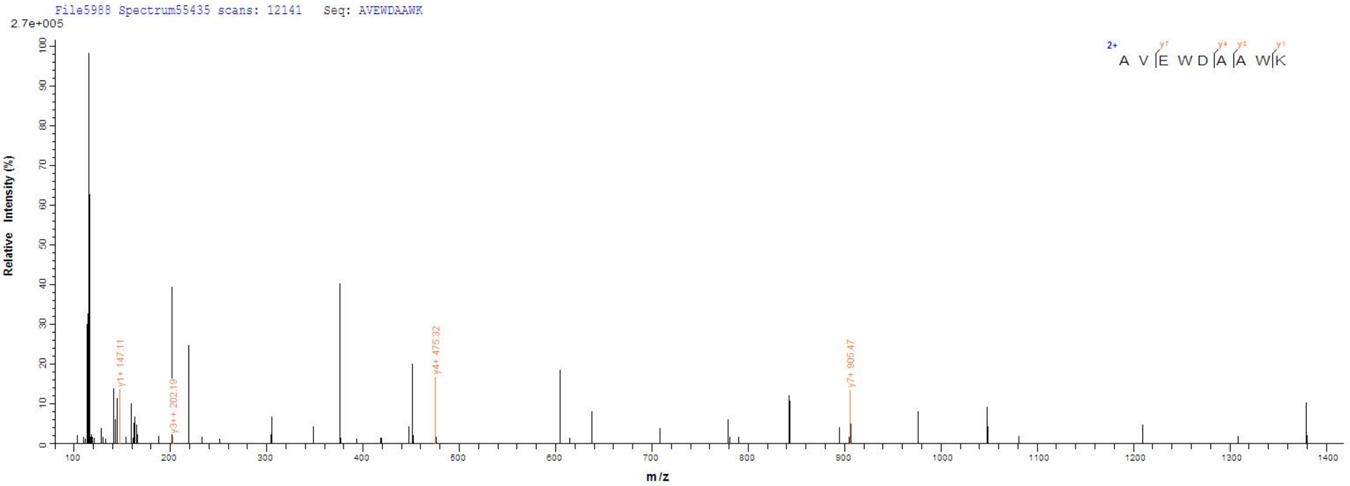

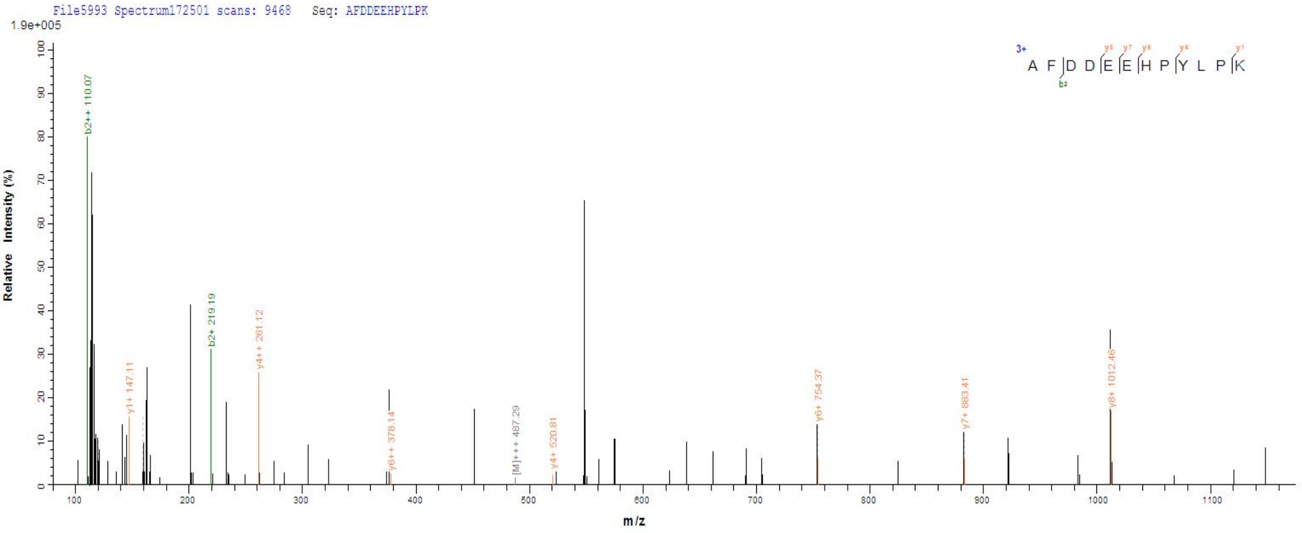

MILRRKGLKHRGPDWSGLHQHGDCYLAHQRLAIVDPASGDQPLFNEDKSVAVTVNGEIYNHEELRKKLVNHKFRTGS
DCDVIAHLYEEYGEDFVDMLDGIFSFVLLDTRDNSYIVARDAIGVASLYIGWGLDGSVWISSEMKGLNDDCEHFECFPP
GHLYSSKTGGFRRWYNPPWFSEAIPSVPYDPLVLRRAFENAVIKRLMTDVPFGVLLSGGLDSSLVASITARYLAGTKTA
KHWGSQLHSFCVGLENSPDLKAAREVADYLGTVHHEFHFTVQDGIDAIEDVIYHIETYDVTTIRASTPMFLMSRKIKSL
GVKMVISGEGSDEIFGGYLYFHKAPNKDEFHHETCRKIKALHQYDCLRANKATSAWGLEARVPFLDKQFINVAMSIDP
ESKMIKRDEGRIEKWILRRAFDDEEHPYLPKHILYRQKEQFSDGVGYSWIDGLKAHADQHVTNKMMRNASNIFPHNT
PTTKEAYYYRMIFERFFPQNSARLTVPGGATVACSTAKAVEWDAAWKNNLDPSGRAALGVHLSAYNAETPLSNMPSQ
VIDNIPRMIEFPGVAIQS

44 Garb_02475 gi|89212812 actin depolymerizing factor 2

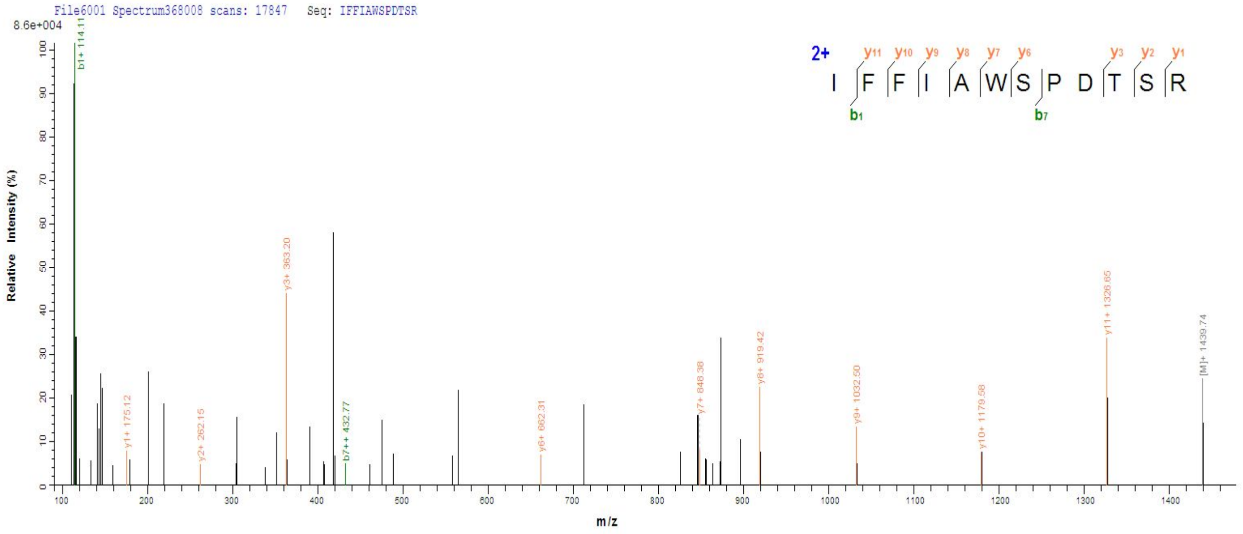

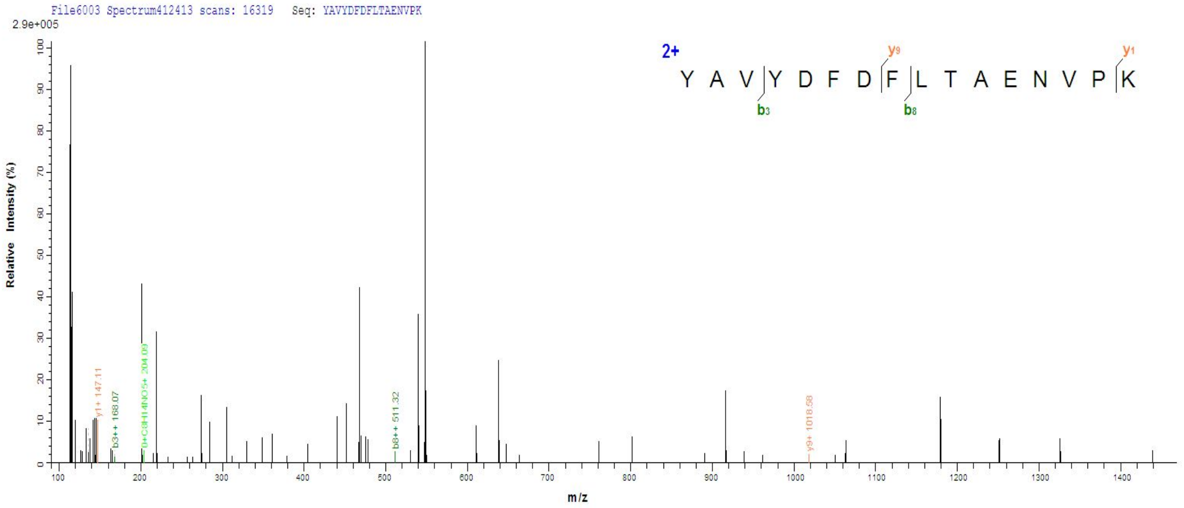

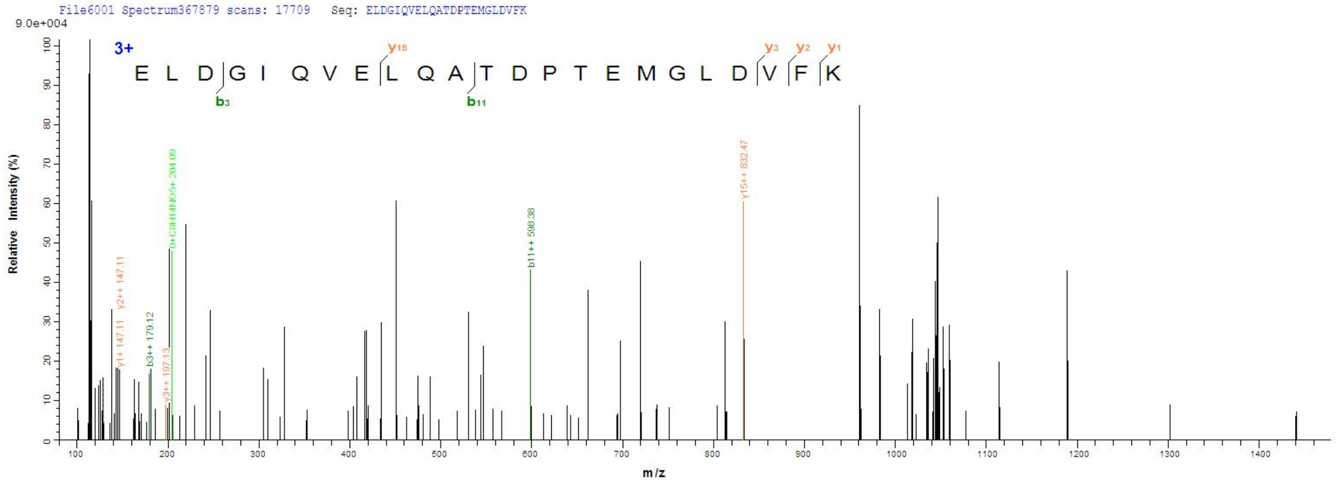

MILYSQANAASGMAVHDDCKLKFLELKAKRTHRFIVFKIEEKQKQVIVEKLGEPTESYEDFTKCLPADECRYAVYDFDFLTAENVPKSRIFFIAWSPDTSRIRSKMIYASSKDRFKRELDGIQVELQATDPTEMGLDVFKSRAN

45 cotton_GLEAN_10014637 gi|117553550 actin-binding protein ABP29

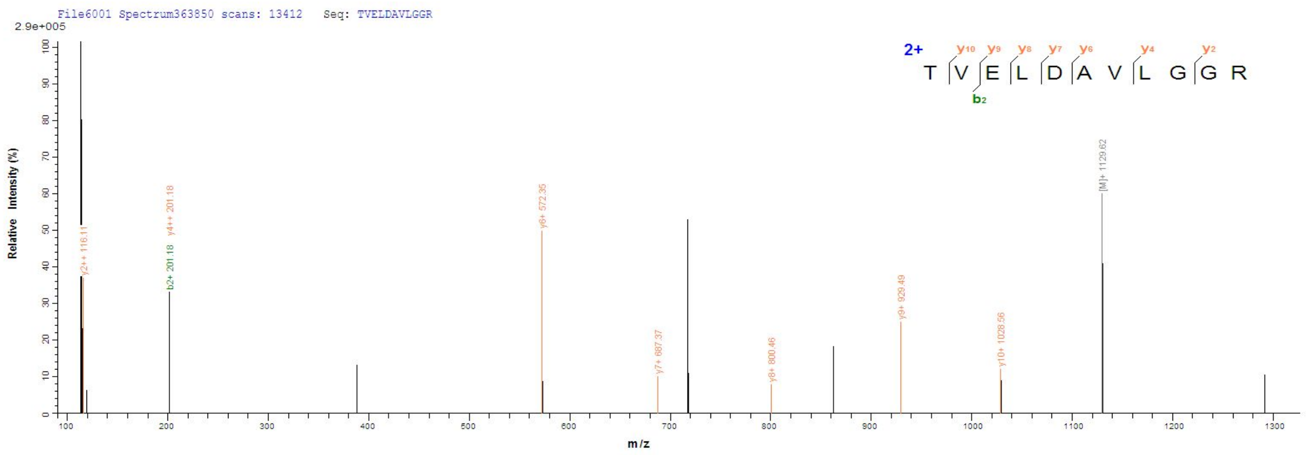

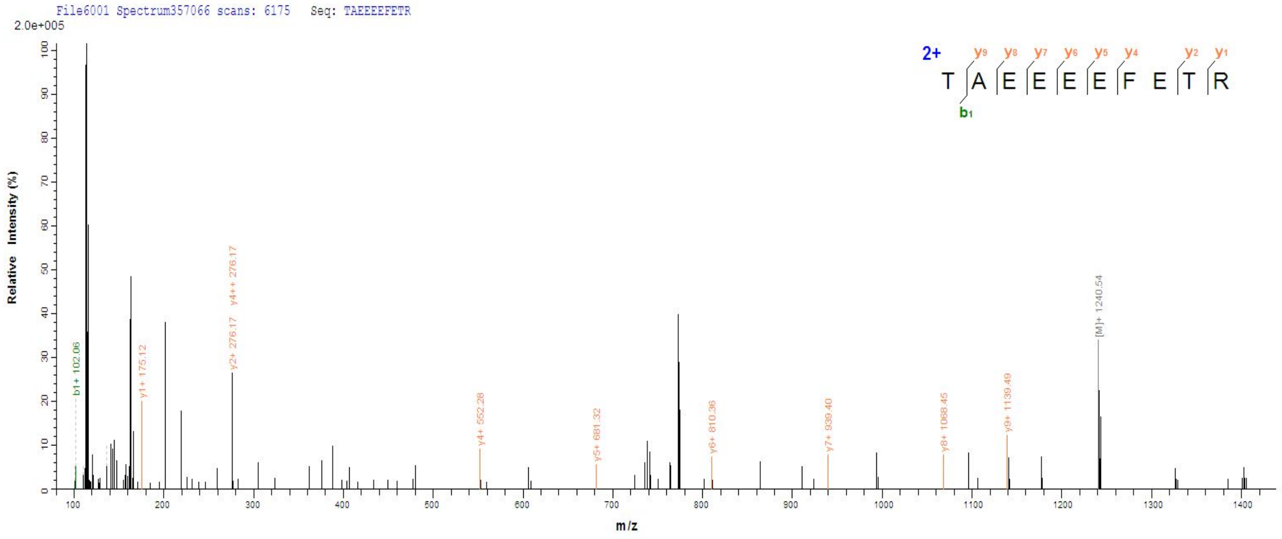

MS

SSKVLDSAFQGLLHLTSYSGTEIWRIENFQPVPLPKSDYGKFYMGDSYIVLQTTPSKGGSYLYDIHFWIGKDTSQDEAG
TAAIKTVELDAVLGGRAVQHRELQGHESDKFLSYFKPCIIPLEGGVASGFKTAEEEEFETRLYVCRGKRVVRLKQVPFAR
SSLNHDDVFILDTQNKIYQFNGANSNIQERAKALEVIQFLKEKYHEGTCNVAIVDDGKLDTESDSGEFWVLFGGFAPIG
KKVTSEDDVIPETTPAKLYSITDGEVKIVEGELSKGLLENNKCYLLDCGAEIFVWVGRVTQVEDRKAASQVAEDFIAGQ
NRPKTTRITRVIQGYETNSFKSNFDSWPAGSAAPGAEEGRGKVAALLKQQGVGVKGMSKSAPMNEEVPPLLDGGGKM
EVWCINSSAKTPLPKEDIGKFYSGDCYIVLYTYHSGDRKEDYFLCCWIGKDSVEEDQKMATRLANTMCNSLKGRPVQ
GRVFDGKEPPQFIALFQPMVVLKGGLSTGYKKSIADKGLTDETYTADCVSLFRISGTSVHNNKTLQVDAVATSLNSIDCF
LLQSGSSMFTWHGNQSTYEQQQLVARVAEFLKPGVALKHAKEGKESSAFWFALGGKLSYTSKTASTEIVRDPHLFTFSL
NKVIDETYNHSNCFFPDFVWFHMDAIGKFEVEEVYNFSQDDLLTEDILILDTHAEVFVWVGQCVDPKEKQNAFEIGQK
YIDMAASLEGLSPHVPLYKVTEGNEPCFFTTFFSWDSTQATVQGNSFQKKVALLFGASHAVEAQDRSNGNQGGPTQRA
SALAALSSAFNPSSASKASTPKPSSTSQGSQRAAAVAALSSVLTAEKKKQSPDASPIKSTSSTPAVTSPPPEAKSEVDPSEL
ADSQEVPEAKETGVVSETSGEDSEPKQEREQDENGNGSTQSTFSYEQLKAKSENPVTGIDFKRREAYLSDEEFQAVFG
MEKEAFYKLPKWKQDMLKKKVDLF

46 Garb_04328 gi|182627650 Actin-related protein4

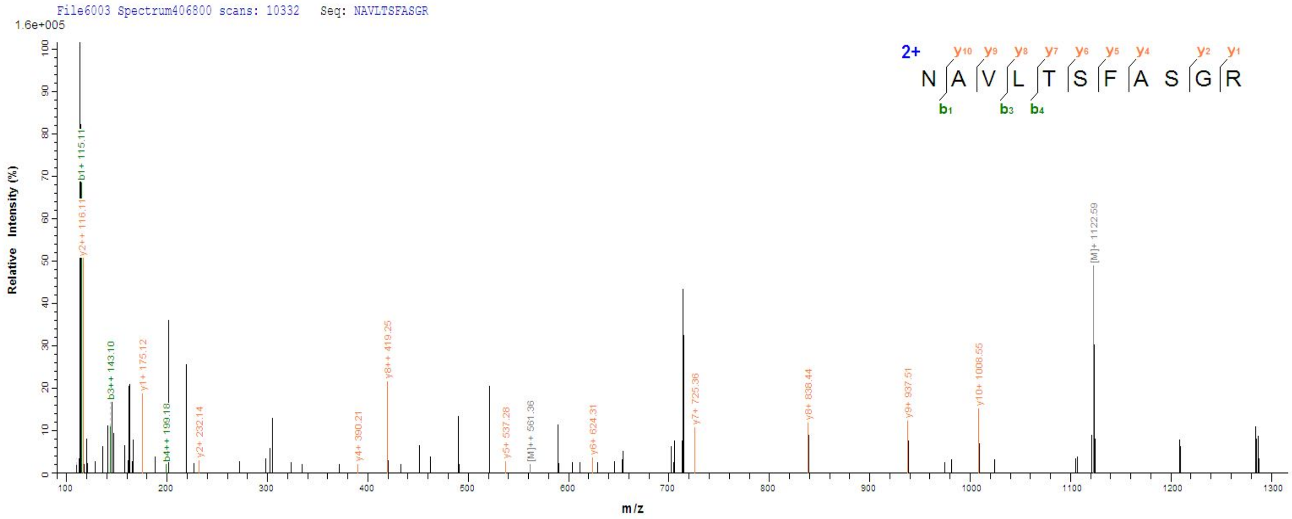

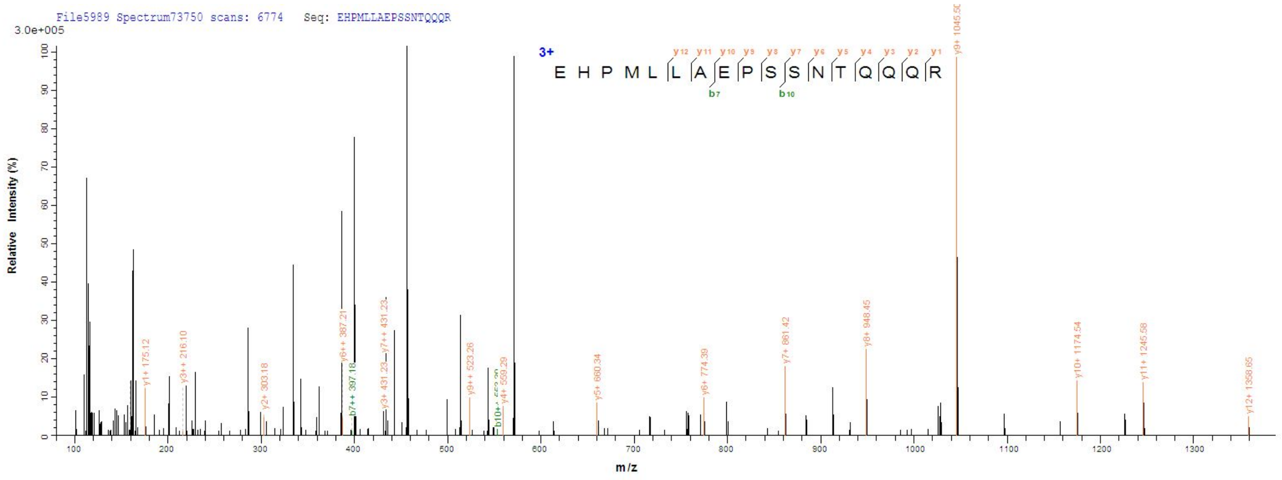

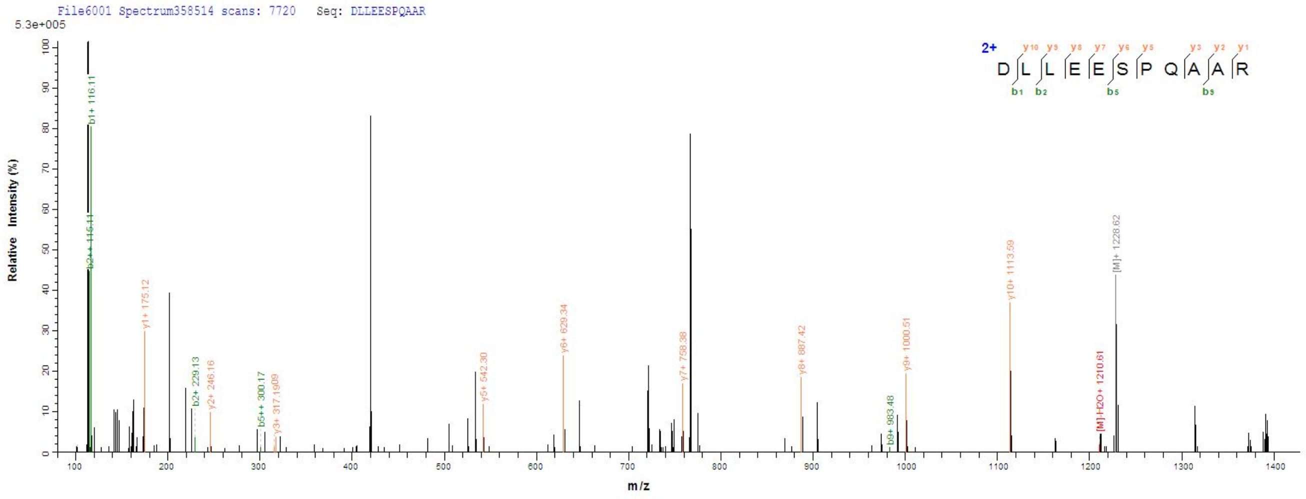

MRIRNSRPPFPLPSVSDPTSRFTHRLPSGNGLYFDDRFLVQETNRQIGWSFYHLKHAARQVSEEGPCGELNKVLTMETR
PINCQDKEKRKSYCMQEKSSQAMGGPHEARANRAHEGSEPYLNKDGNTSSGAVNFDIAQPFTSYELGGEVSDKKKKQ
ICLLETEMARGRKEDAILEKKMELGNVSEKSIEDEYGSKRTRTLFCPTNCEYFFLPENEGFEGYGMQGGMSFEDMVVE
TKEKPRRVVVGCIDQMDADDDKENNDSETNNNNVDSNKPKGKRRLYVGSQALGFRRDHMEVVSPLKDGVVVDWDI
VDSIWDHALKDCLLVDPKEHPMLLAEPSSNTQQQRERTAELMFEKYKVPALFLAKNAVLTSFASGRATSVVVDSGGGS
TTVAPVHDGYVLQKAVSSSPIGGEFLTDCLMKSLESKGIVIKPRYSFKRKEIQPGVFQTVDVDFPHTTESYKLYSQRVIAS

DIKECVCRAPDTPYDESAYSNIPMTPYELPDGQTIEIGADRFKIPDILFNPPLAQTIPGMDNLAEISSSVRGLPQMVIESIN RCDVDIRKELFGSILLAGGTASMQQLKERLEKDLLEESPQAARVKVLASGNATERRFSVWIGGSILASLGSFQQMWFSK SEYEEHGASYIQRKCP

47 Garb_19713 gi|157273642 fasciclin-like arabinogalactan protein 4

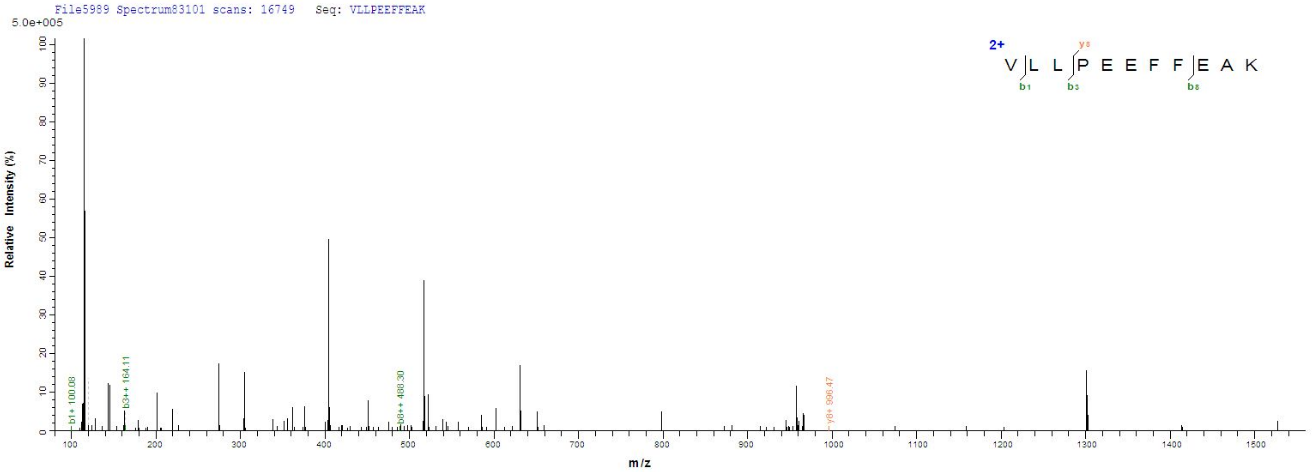

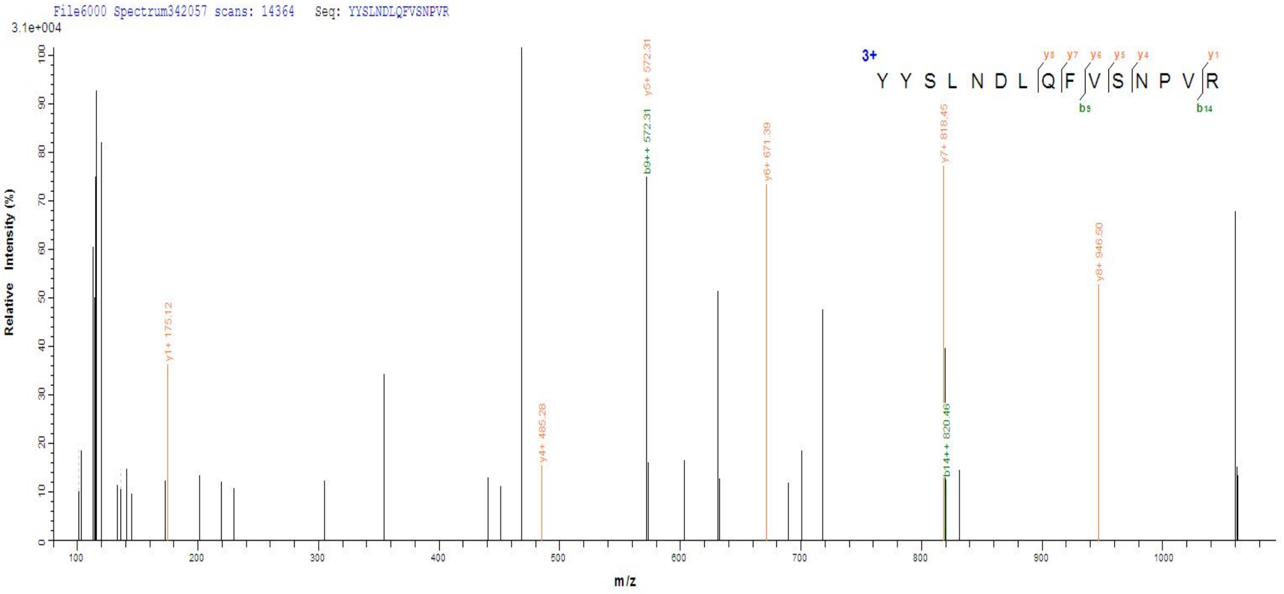

MASSSPLSPILLSLFLVFICGVSAQTAPAPAPSGPLNFTGILDKNGQYTYFLQLLAQTQVGSQVQTQLKTTTEGFTVFAPT DNAFNNLKPGTVNNLDPEQKVQLVLYHVIPKYYSLNDLQFVSNPVRTQAGEDFGLNVTGLNNQVNVSSGVVETQINN ALYQKKPLAIYQADKVLLPEEFFEAKSPAAAPSPATKKSSTGSKSNSRASATADEPASADNSGSTVRNMGLGFVVGLAL ACMGFLS

48 cotton_GLEAN_10015912 gi|150416583 fasciclin-like arabinogalactan protein 11

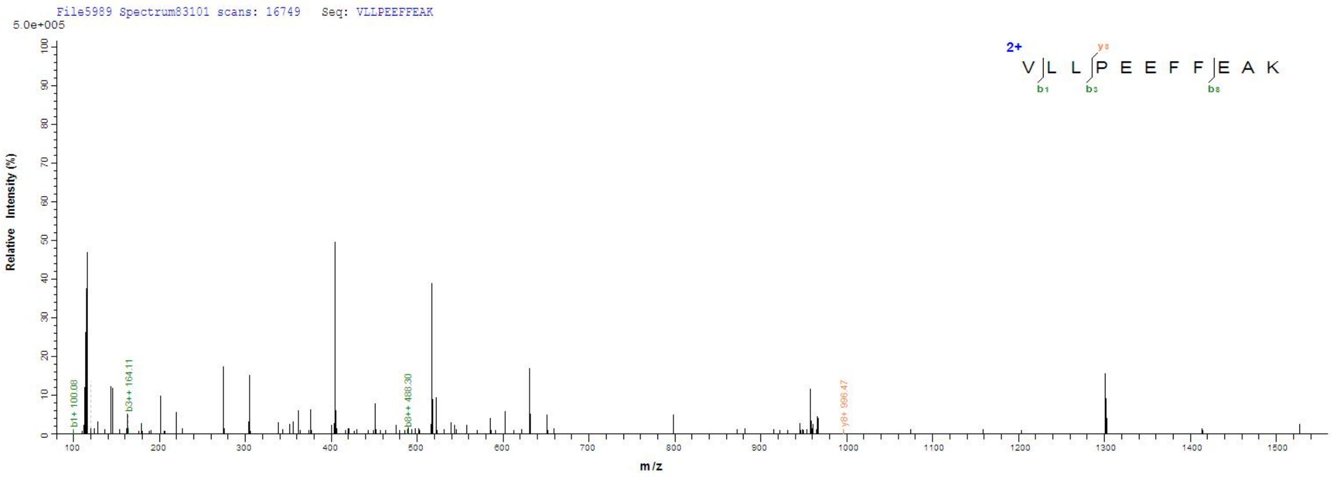

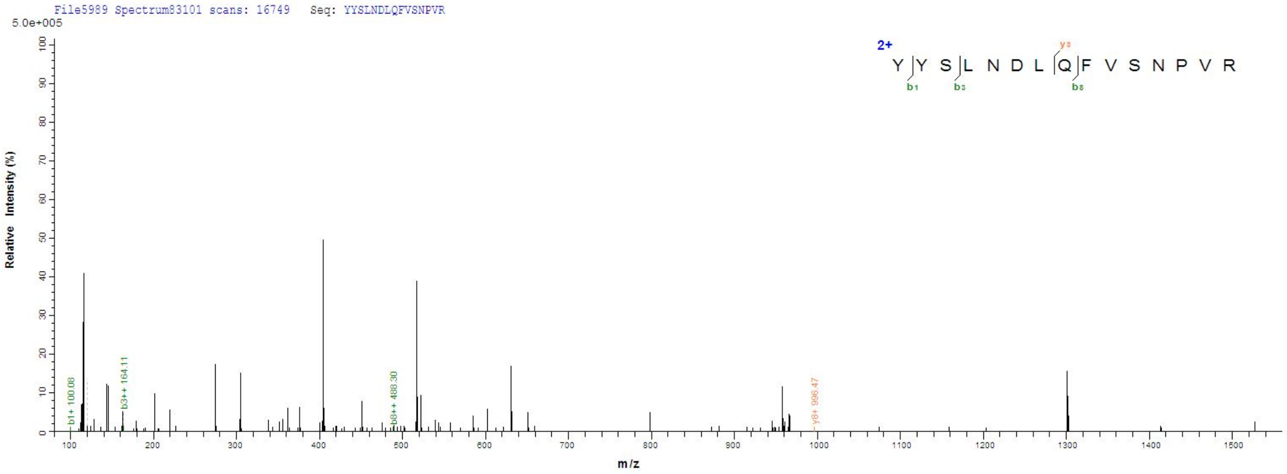

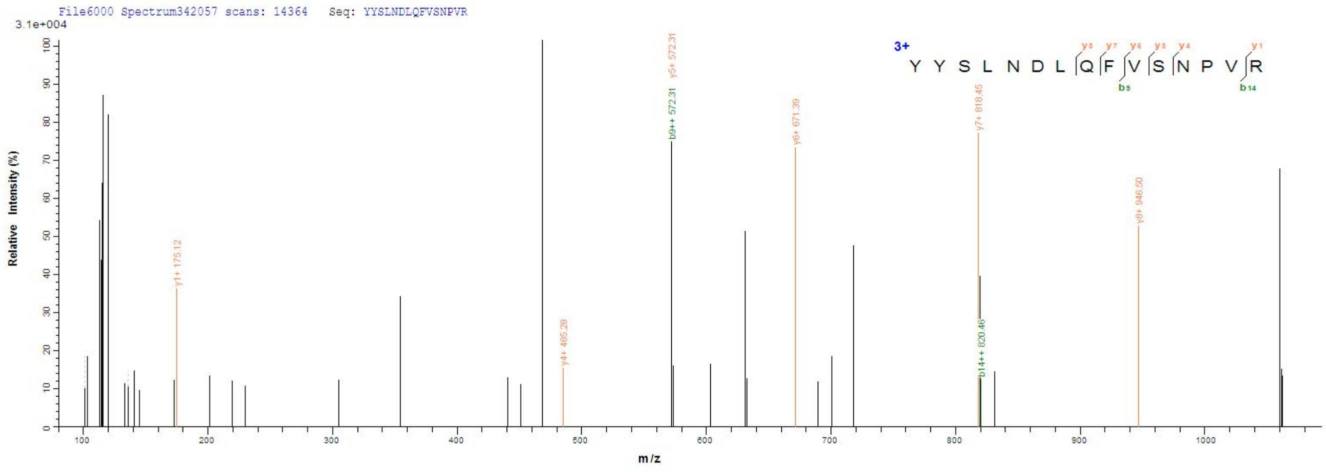

MASSSPLSPILLSLFLVFICGVSAQTAPAPAPSGPLNFTGILDKNGQYTYFLQLLAQTQVGSQVQTQLKTTTEGFTVFAPTDNAFNNLKPGTVNNLDPQQKVQLVLYHVIPKYYSLNDLQFVSNPVRTQAGQDFGLNVTGLNNQVNVSSGVVETQINNALYQKKPLAIYQADKVLLPEEFFEAKSPAAAPSPATKKSSTGSKSNSRASATADEPASADNSGSTGRNMGLGFVVGLALACMGFLS

49 Garb_17034 gi|157273666 fasciclin-like arabinogalactan protein 16

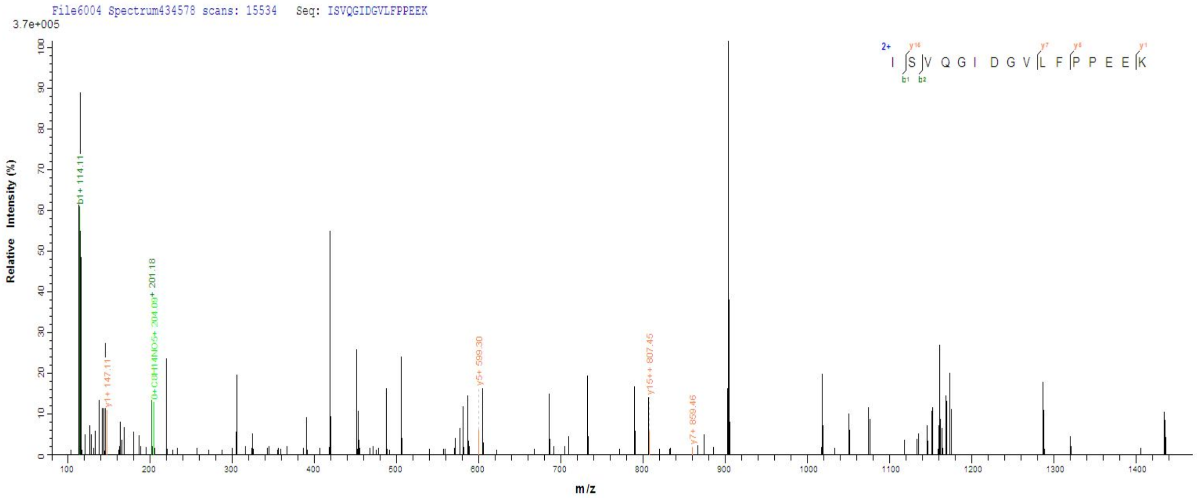

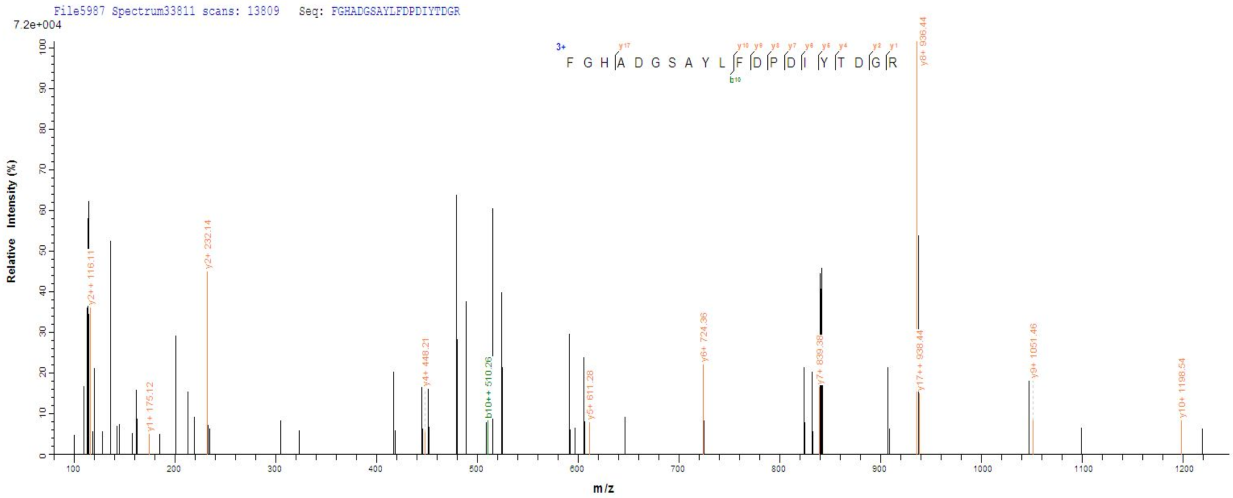

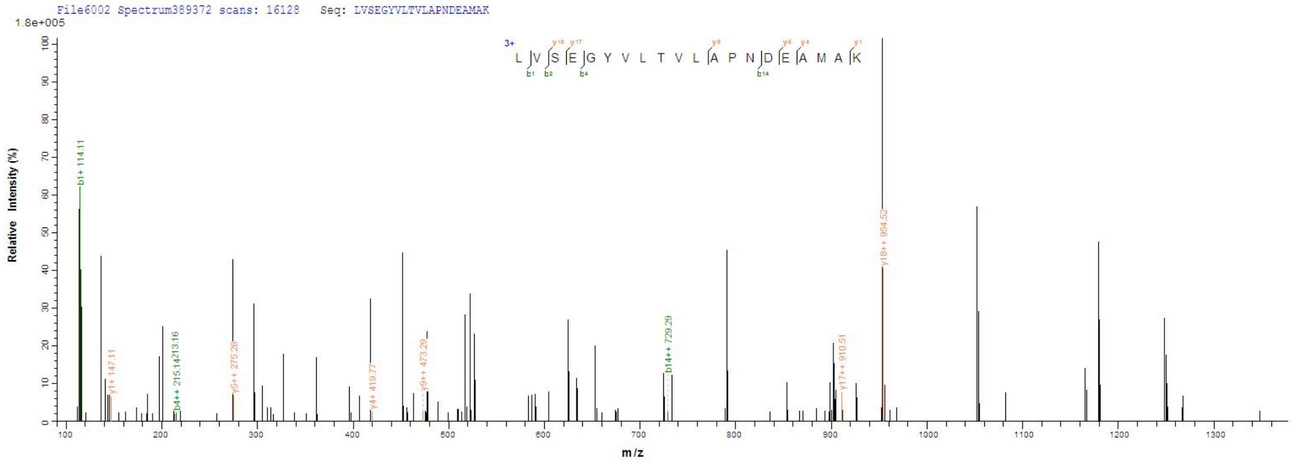

GFSLLFKFLLLLSGFGVSMALQETPLVAKNLGQISSNSVLVALLDSHYTELAELVEKALLLQSLEESVGKHNITIFAPKNE
ALERNLDPEFKRFLLEPGNLKSLQTLLLYHIVPTRIEPHSWPNSTTGSIIHRTLSNHNVELSSEDSMGVKFIGSAKVINPN
AVNRPDGVIHGIEQLLIPQSVQQDFNSRRNLRSISAVKPEGAPEVDPRTHRLKKPAPPVKPGSPPVLPIYDAMAPGPSLAP
APAPGPGGPHHHFNGMRQVKDFIQTLIQYGGYNEMADILVNLTSLATEMGRLVSEGYVLTVLAPNDEAMAKLTTDQLS
EPGAPEQIIYYHIIPEYQTEESMYNTVRRFGKVSYDTLRLPHKVSAQEADGSVKFGHADGSAYLFDPDIYTDGRISVQGI
DGVLFPPEEKTKEEKKTIKVATAKPRRGN

50 cotton_GLEAN_10018582 gi|157273646 fasciclin-like arabinogalactan protein 6

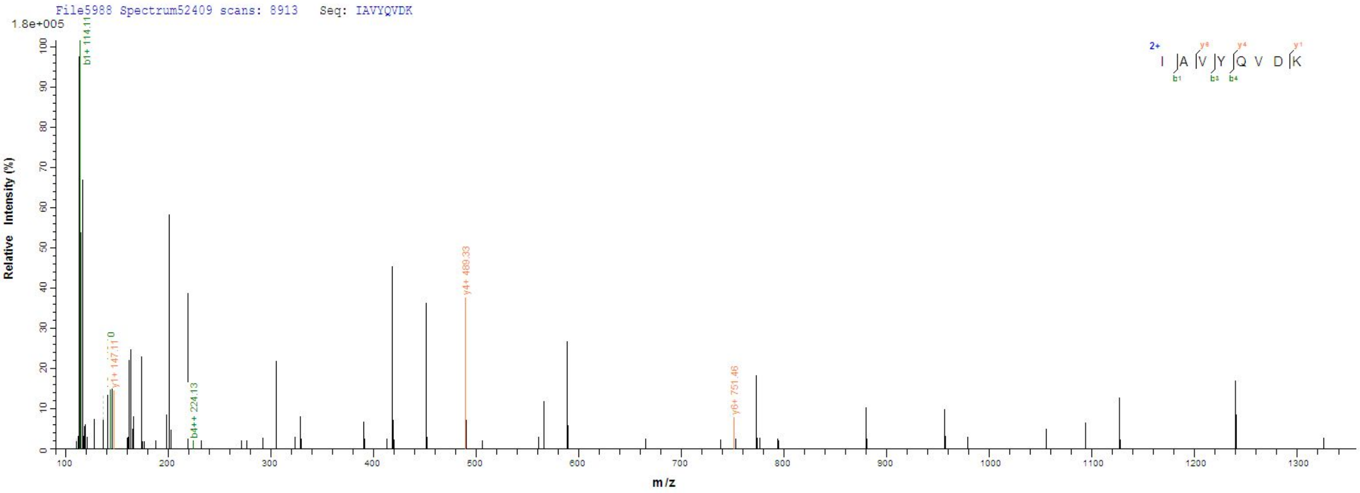

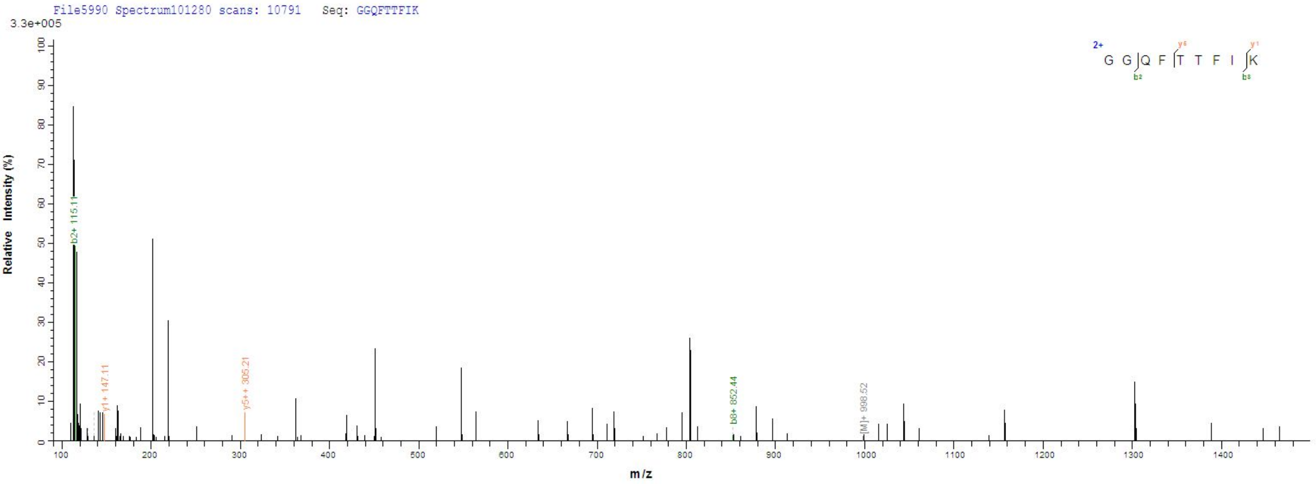

MRKLLAFAFLFFFFWDKTLAQIAPAPPLKVDNITSILEKGGQFTTFIKLLKATQVADQLNNQLSTPDPNDGITVFAPSDN
AFSGLKSGTLNSLSDQEKLQLVQFHILPTLMSTSQFQTASNPLRTQAGDVKGGKFPLNVTAEGNQVNVTTGVVNATVE
NSVFSDRRIAVYQVDKVLLPLEIFGTAPEPAPAPVVPENGGLVSSPKTAVKGADAAGAEPAPAPVVPENGGLVSSPKTAV
KGADAAGAVSLKFHALAMGFSSFAVFWAILGGF

51 cotton_GLEAN_10040764 gi|157273640 fasciclin-like arabinogalactan protein 3

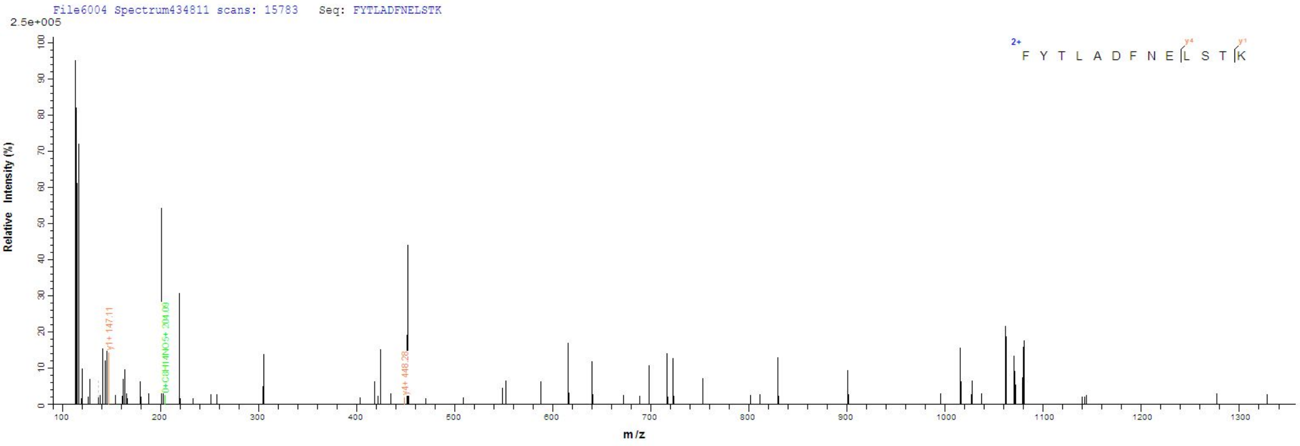

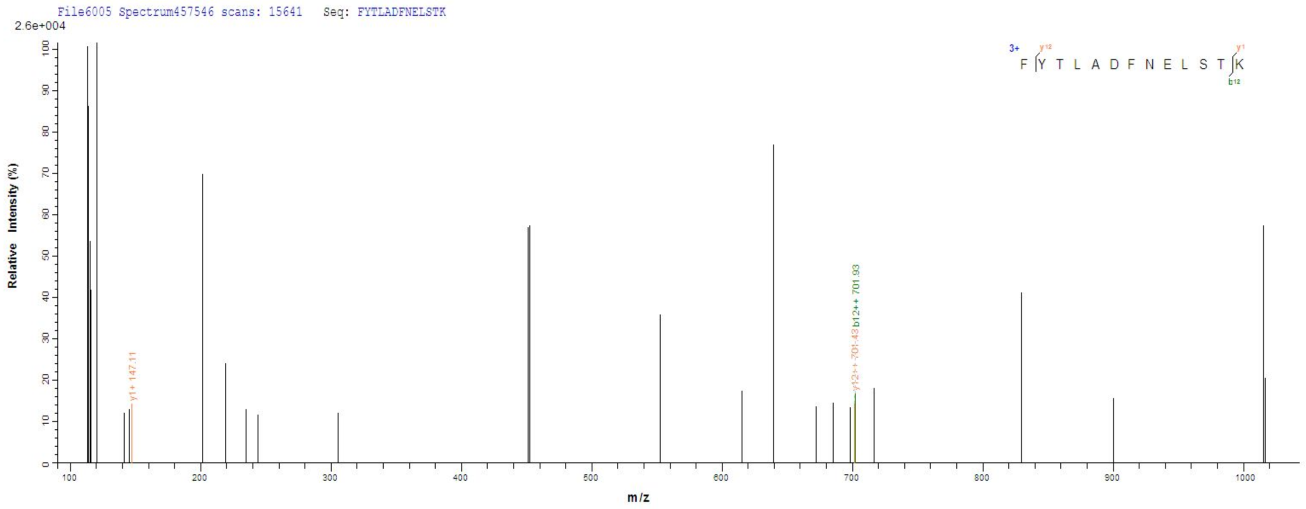

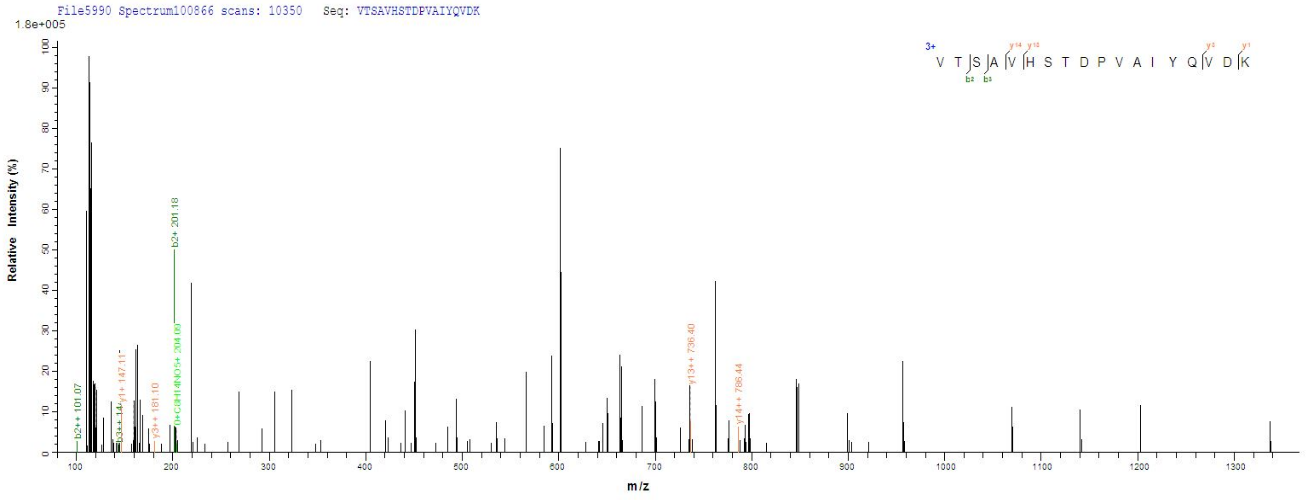

MEFSKIFTICFSVLLLCSSLAYGQARSPPAPVAMSPTPTPAYVNLTYLLSVAGPFHTFLDYLESTKVIDTFQNQANNTDQG
ITVFVPKDSAFKDLRKPSFSNLSDDQLKSLILYHALPRFYTLADFNELSTKGSISTLAGGEYTLNFTYDSGTVHLDSGWS
KTKVTSAVHSTDPVAIYQVDKVLLPEAIFGTDIPPTPAPSPAPVPDVSPAADSPSAESKESGSSPKSAPSTSSSNSIMNLCI
WRQLVLAVSAGVVLFF

52 Garb_33627 gi|157273638 fasciclin-like arabinogalactan protein 2

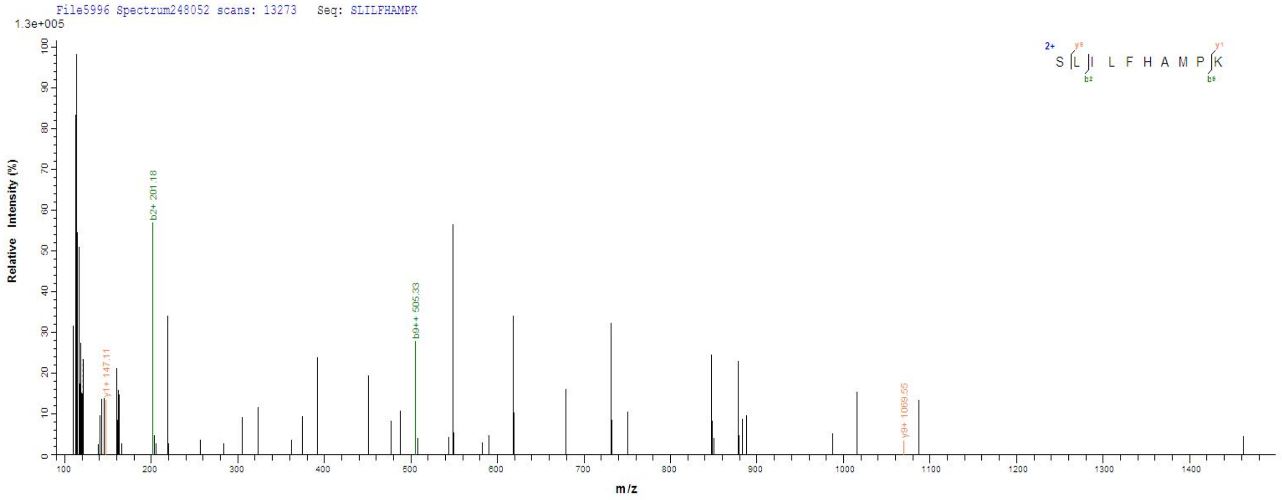

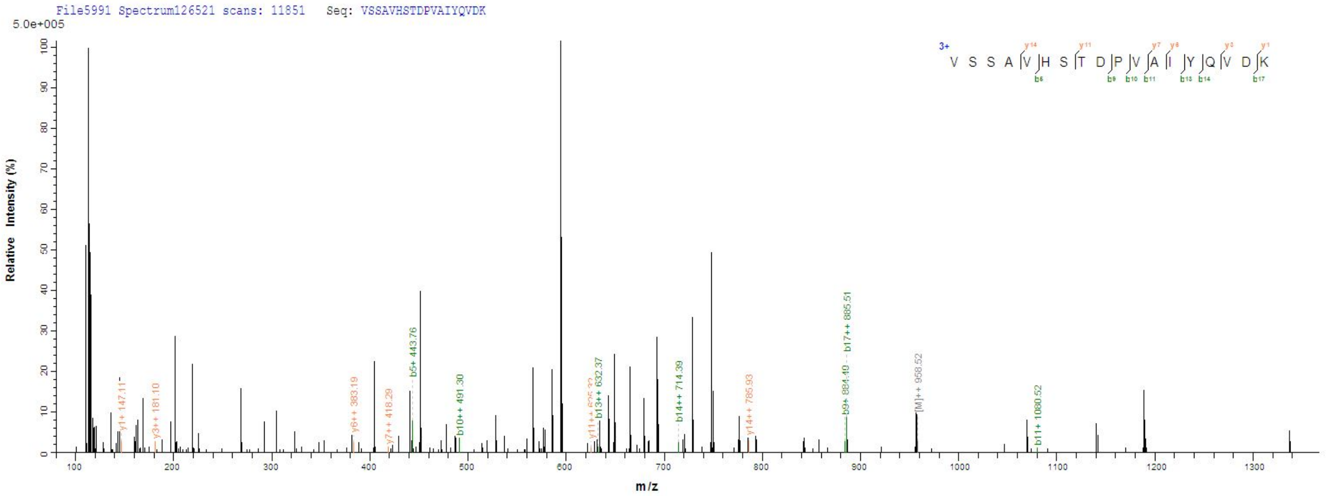

ME
FSRVSMISCFAFLLCSSLAYGASSPPAPMAMSPSPTPTPAPAPAPEYVNLTYLLSVAGPFHTFLNYLESTKVLDTFQNQAN
NTDQGITIFVPKDSAFKALKKPSLSNLTNDQLKSLILFHAMPKFYSLADFNKLSTKGPVSTLAGSQYSLNFTDNSGTVH
LDSGWSKTKVSSAVHSTDPVAIYQVDKVLLPEAIFGTDIPPMPPAPAPAPDISPAADAPSAETKGKGSSSKAEPSTSSSHRI
MNFGTWNQLVLALFGGWVMFF

53 cotton_GLEAN_10023074 gi|157273660 fasciclin-like arabinogalactan protein 13

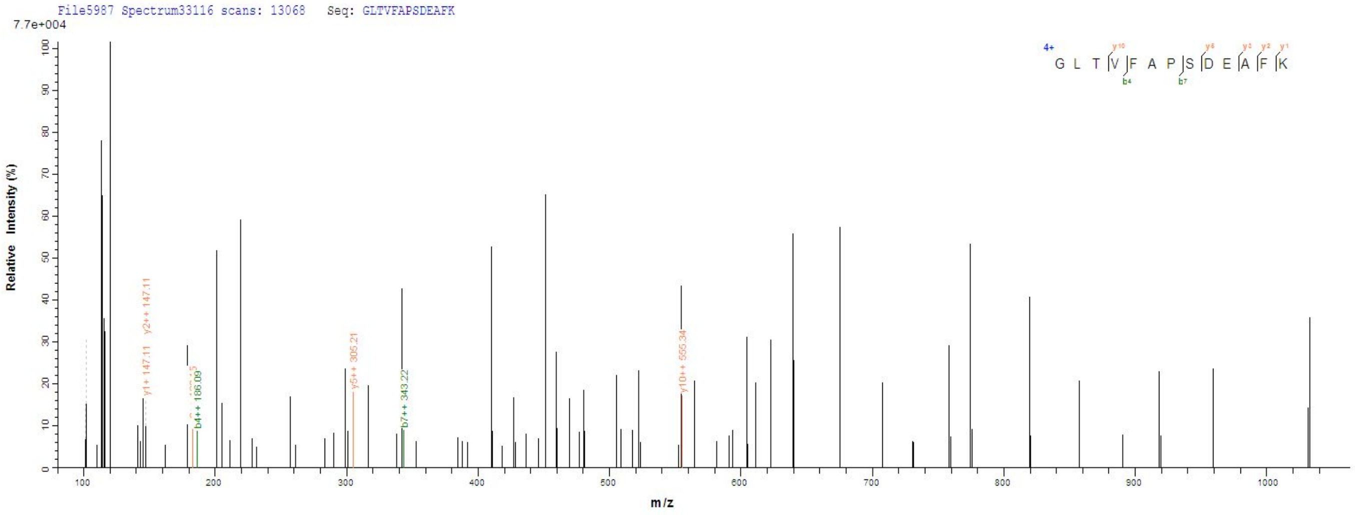

MGAFHRLNLLHFTLSLVVIAVNGHNITAILEGFPDYSVYNSFLTQTKLADEINTRETITCLVLNNGAMSALTSKHPLSVV
KNILSLHVLLDYYDPQKLHKISDGTTLTTTLYQTTGNAPGNLGFVNITDLQGGKVGFGSAIPGSKLDSSYTKSVKQIPY
NISILEISAPIIAPGILTAPAPSSSGVNITGLLEKAGCKTFASLLTSSGVLKTYESALDKGLTVFAPSDEAFKAEGVPDLGKL
TNAEQVSLLEYHASPDYKPKGTLKTTKDPISTLATNGAGKYDLTVTTAGDSVTLHTGVGPSRVAEAVFDSPPVAIFTVD
NVLLPSELFGKSPSPAPAPEPVSSPSPTPSLSPSPSPMSEAPSPLAASPPAPPTGTPVGSPADSPAGSSENSTSDNAAGHVSA
PLLGTIIFTVFATVVSSVILS

54 Garb_32655 gi|253509569 caffeic acid O-methyltransferase 2

File5994 Spectrum195904 scans: 9346 Seq: AGPGAFLSPK

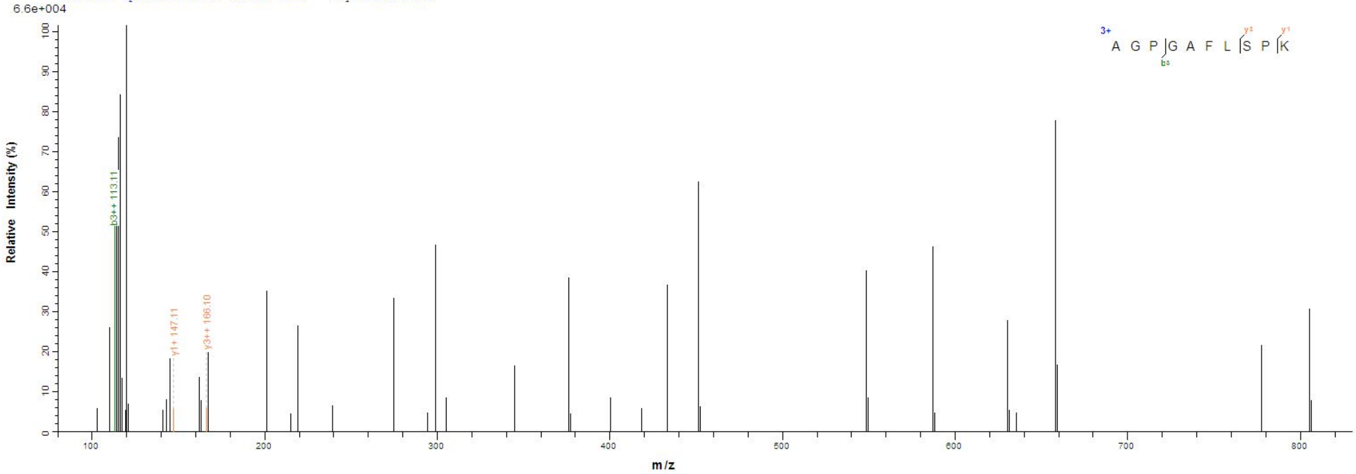

File5990 Spectrum100236 scans: 9678 Seq: SAGFQGFQVK

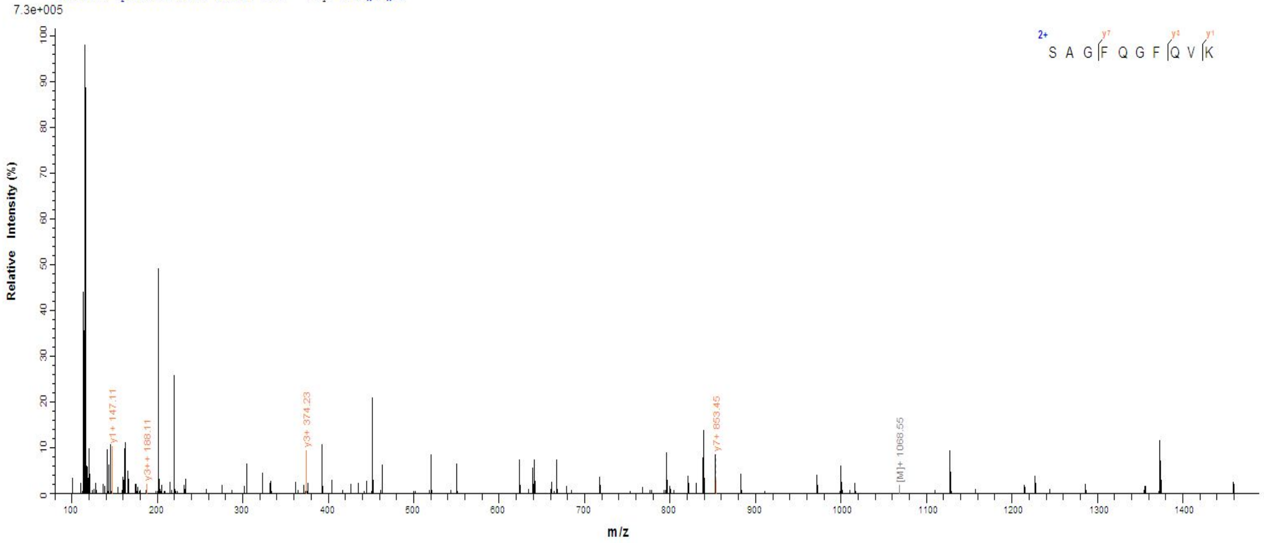

File5987 Spectrum32396 scans: 12300 Seq: DAVLEGGIPFNK

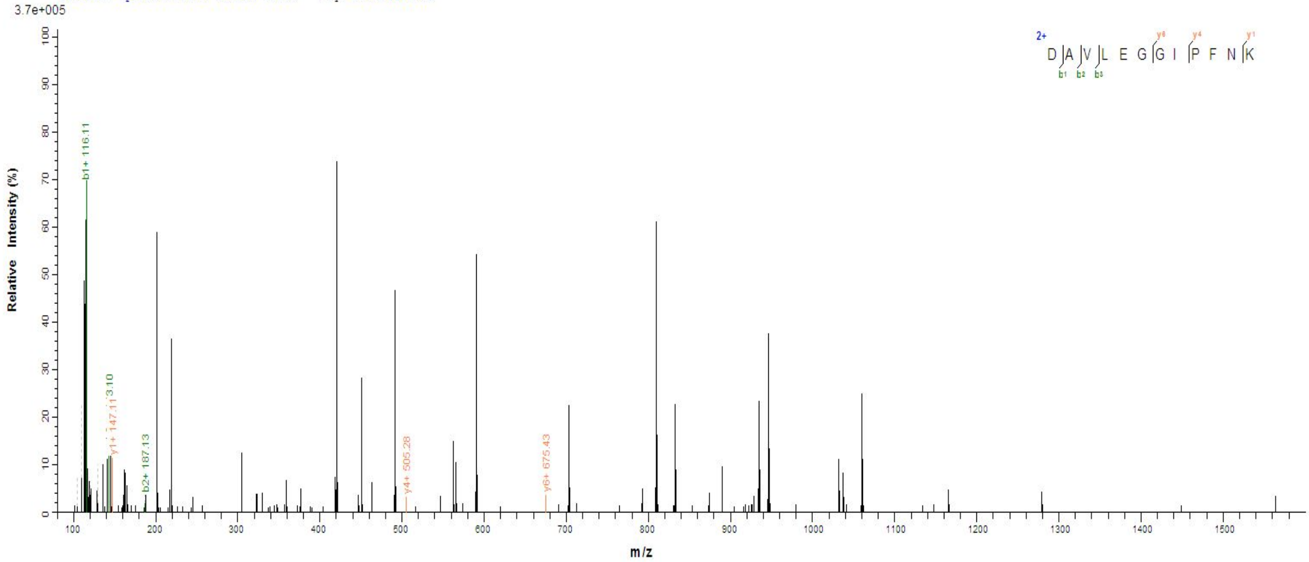

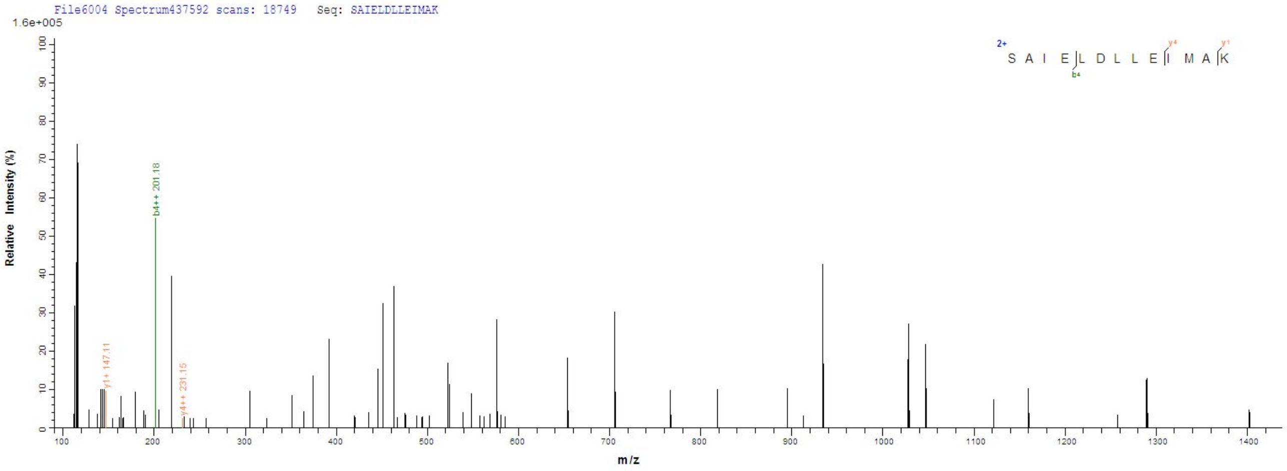

MGSTGETQMTPTQVSDEEANLFAMQLTSASVLPMVLKSAIELDLLEIMAKAGPGAFLSPKELASQLPTNNPDAPVMLDRILRLLATYSILTCSLRTLPDGKVERLYGLGPVCKFLTKNEDGVTLSALSLMNQDKVLMESWYYLKDAVLEGGIPFNKAYGMTAFEYHGTDPRFNKVFNRGMSDHSTITMKKILETYDGFEGLKTLVDVGGGTGATLNMLVTKHPSIKGINFDLPHVIEDAPAYPGVEHVGGDMFESVPKGDAIFMKWICHDWSDEHCSKFLKKCYEALPDNGKVIVAECILPDYPDPSLATKLVVHIDCIMLAHNPGGKERTEKEFEALARSAGFQGFQVKCCAFGTYIMEFLKRV

55 Garb_18854 gi|224552010 hybrid proline-rich protein

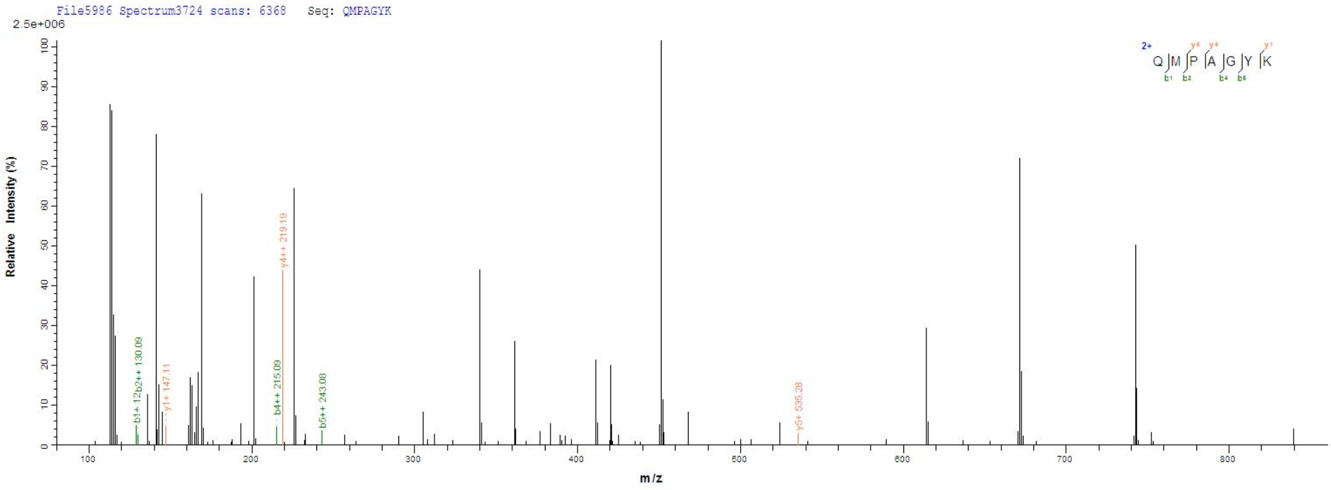

MASNTVASAALLLTLNLLFFTLVSSTYVPCPPPPKSHKPTHKSPPSAPEQPGTCPRDALKLGVCADLLGSVRVVVGPSRTKCCSLISGLADLDAAVCLCTAIKANVLGVNLNVPVSLSLLLNSCEKQMPAGYKCA

56 cotton_GLEAN_10039929 gi|255547195 glycine-rich RNA-binding protein, putative

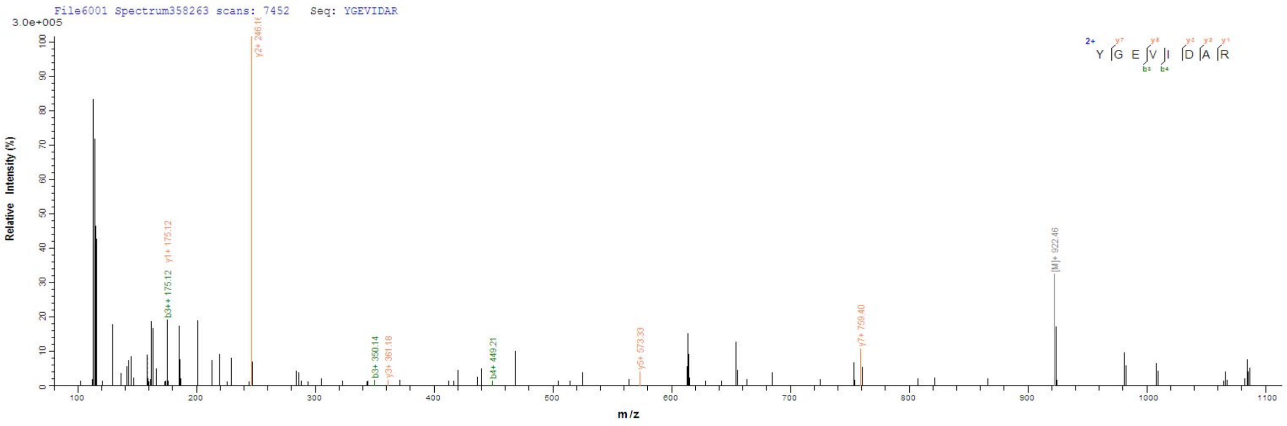

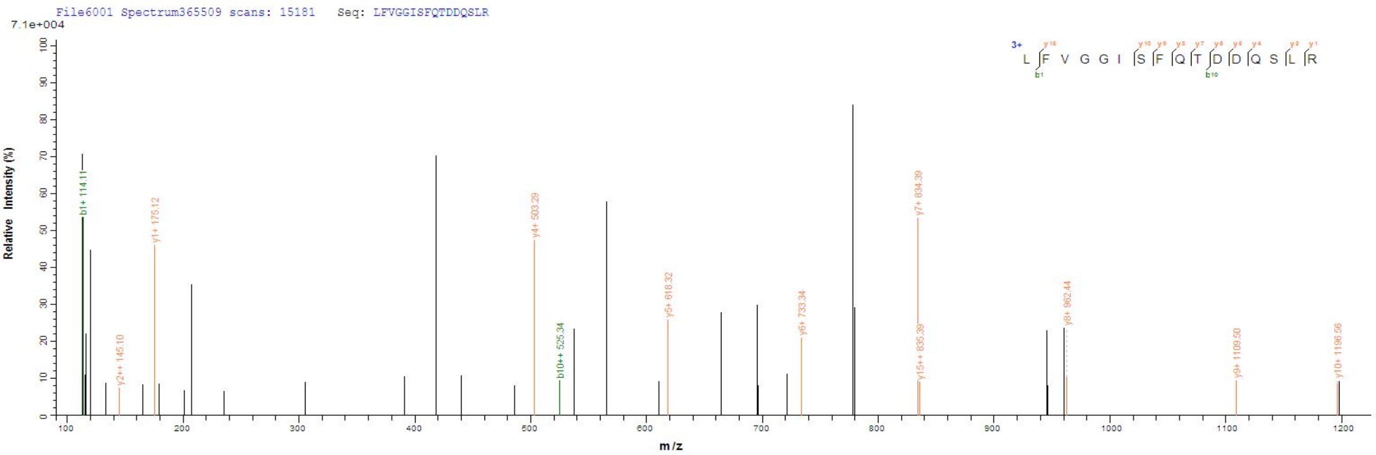

MVIAMAFLSKIGSILRQTTSMHVNAELSASRPGLFQVLRSMSTSPSSKLFVGGISFQTDDQSLREAFSKYGEVIDARIIM
DRETGRSRGFGFVTYTSSEDASSALQALDGQVLHGRQVRVNYANERPPRNFGGGGFNSGYGGGGYGGGGGGYGGGS
GGYGGGGGGYDRNDGFSSGNYGGNVGYANTGGSSYGGQGSYGGGNNYGTGGVSYGSDFGQSGVDGGNFNVGGGD
SFSTGGSTGYGGDSMGFGGGEDGYKGGAYDGNDALNDNSRDEDESGDFVKRA

57 Garb_02630 gi|259016223 Glucan endo-1,3-beta-glucosidase 7

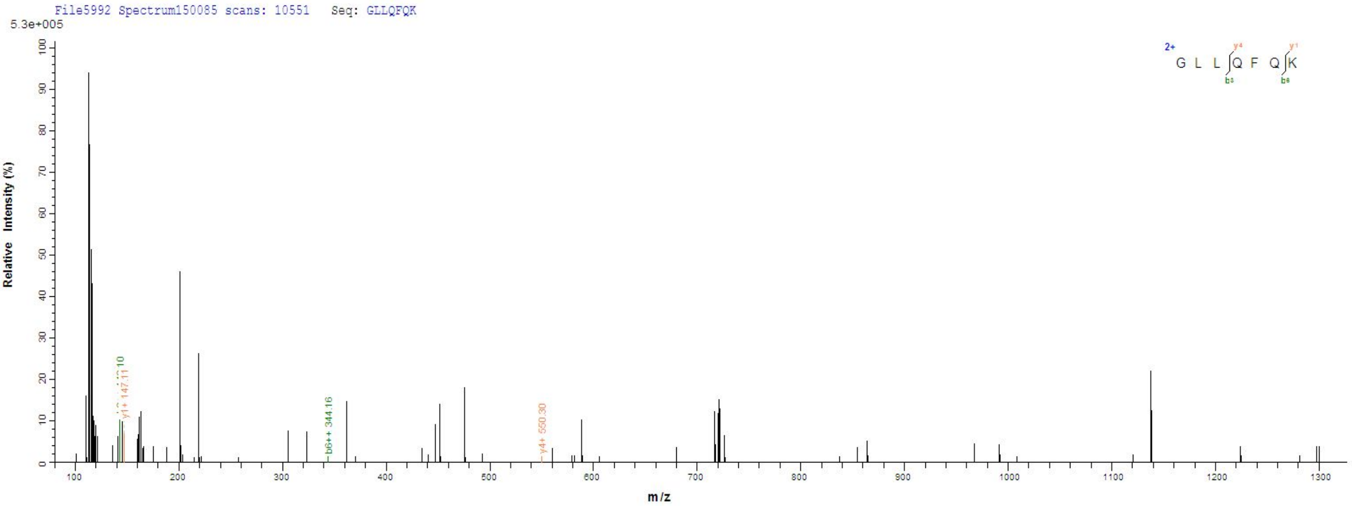

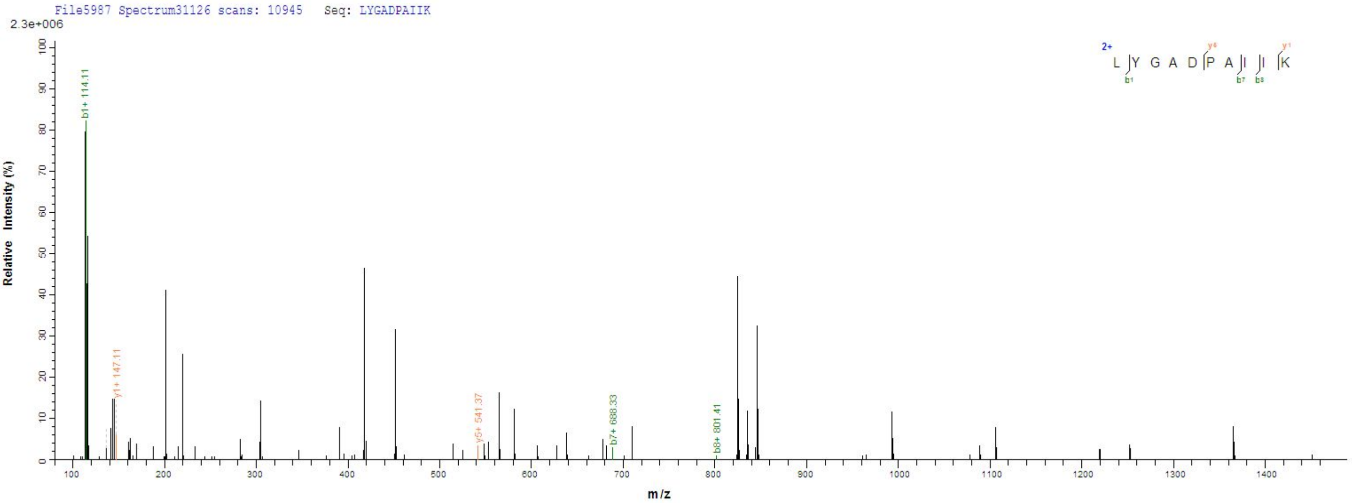

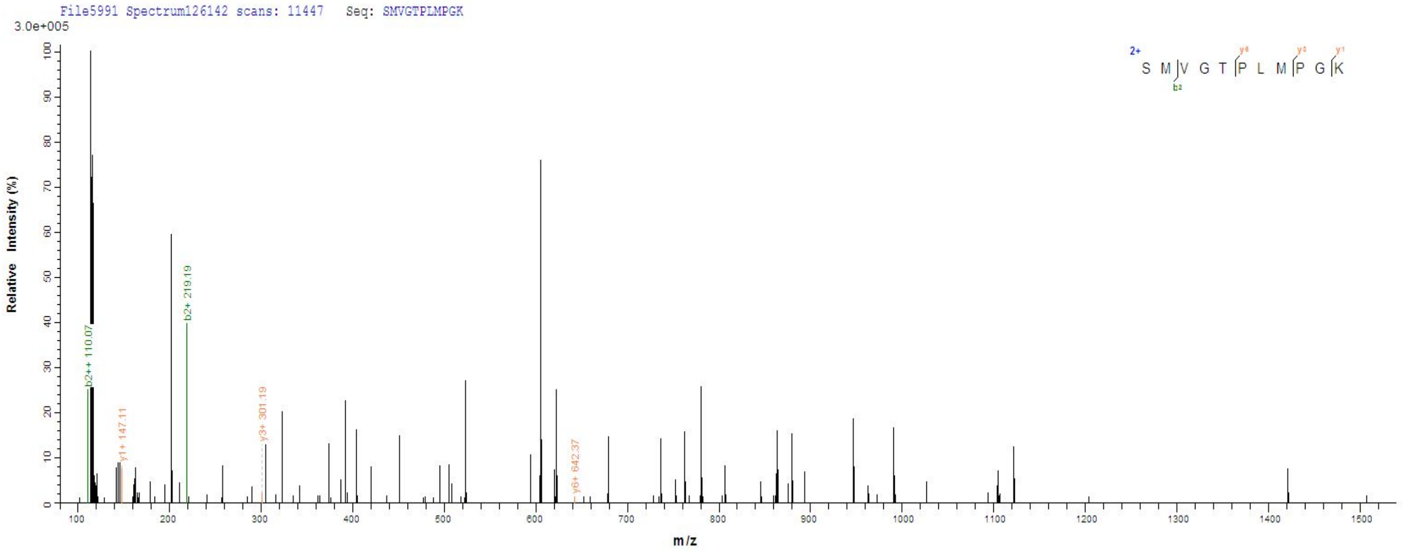

MATFFFFLLSFLICFHLSSAEPFIGVNYGQVADNLPPPSATAKLLKSTSIEKVRLYGADPAIIKALANTEIGIVIGAANGDIPSLASDPNSAAQWINSNVLPFYPASKIILITVGNEVLTTNDPNLINQLLPAMQNMQNAINGASLGGKIKVSTVHSMAVLGQSDPPSSGLFSPSYQPALKGLLQFQKDNGSPFAINPYPFFAYQSDSRPETLAFCLFQPNAGRVDSGNGIKYMNMFDAQGKRKEEDYILTIMQHFATDYGIEMLIVGVDSVHSALSAMGFKDVEIMVAETGWPYSGDSNEVGPSIENAKAYNGNLIAHLKSMVGTPLMPGKSVDTYLFALYDEDLKPGPGSERAFGLYKPDLSMVYDVGISKSSQTPSTPSTPVTPQPKPTATGWCVPKAGISDAQLQSSLDYACGQGIDCSPIQPGGACFEPNTIASHAAYAMNLYYQSSAKNPWNCDFSQTATLTSQNPSKYTSLKLHLSSIAFFLG

58 cotton_GLEAN_10037095 gi|255546283 Glucan endo-1,3-beta-glucosidase precursor, putative

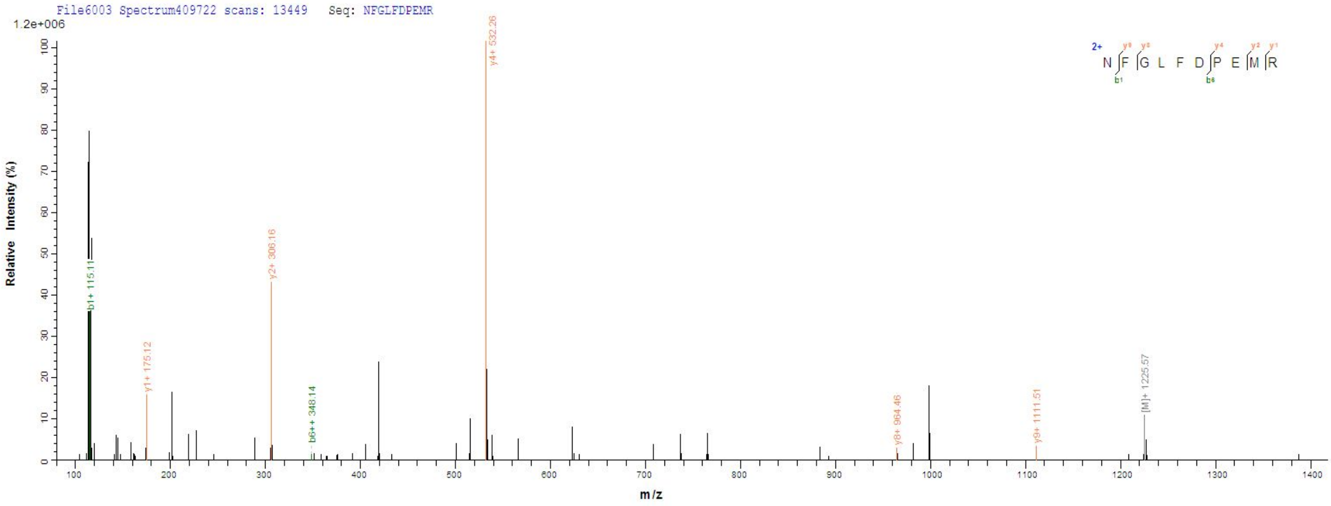

MASVYNLGFPDEGGRSWCIASSQALDSDLQYLLIWVCILGKATCSAIQPGQQCFEPDTLFSHTSFAFNNYYQKNGATDDACNFGGTGIKAMVNASITFYSHILQTQAIILIQYENKYFCRGSNVGVDYGMDADNLPSPDQVAQLVRNHNIQYLRIYNYRPEVLKAFSNTGIELMVGVPNADLSQFQSQPYVDSWLRTSILPYYPATKITHITVGVEVTNYPDNTANLVVPAMRNVVSALKSANLQGKIKVSTPLSFGVLSKSFPPSEGAFNSGYENVLRLLLDFLEENQSPFMVNLYPIYAIGASSLDAVLFKSPSTIFVDQHTGLSYKNIFDAQLDAVFYAIANRNFRTTRNTFDAQPDTVHFTLANTNSRINDIIVTETGWPTHGSRPPHASTHNAKTYNYGASLDSVDDYANIDNAQTYNTNLISHVMGGSGTPAMPGANLDVYIFSLFNENLKQGPEIERNFGLFDPEMRSVYNLNFPGKGTGKSWCIASSQASNSALQNALDWACGQGKADCSAIQPGQRCFQPDTLVSHASFAFNNYYQKNGATDDACSFGGTGIKVSTDPSYGNCIYN

59 Garb_22900 gi|255573702 Glucan endo-1,3-beta-glucosidase precursor, putativ

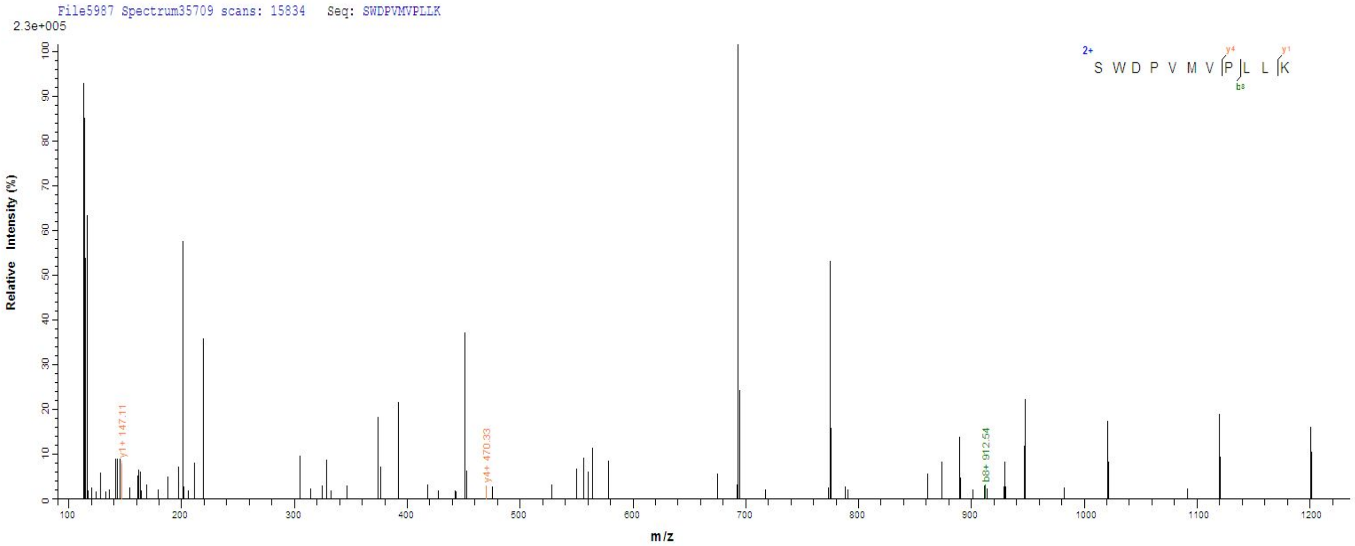

IGVNIGTDLSDMPTPTEVVALLKAQRIRHVRLYDADQAMLLALAKTGIQVTVSVPNDQLLGIGQSNATAANWVARNIV
AHVPATNITAIAVGSEVLTALPNAAPILVSALKFIHSALVASNLDDQIKVSTPHSSSIILDSFPPSQAFFNRSWDPVMVPLLK
FLQSTGSYLMLNVYPYYDYMQSNGKIPLDYALFRPLPPNKEAVDANTLLHYTNVFDAVVDAAYFAMSYLNFTNIPIVV
TESGWPSKGDSSEPDAIVDNANTYNSNLIKHVLNNTGTPKHPGIAVSTYIYELYNEDLRPGSDSEKNWGLFDANGIPVY
TLHLTGADTVFANDTTNKTFCIAKRGADPKMLQAALDWACGPGKVDCSPLLMGHPCYEPNNVASHSTYAFNAYYQR
MAKSPGTCDFKGVATITTSDPSHGLCIFPGSSRKNGTLINGTSLAPSSNDTSSARPPQSYGTGSFTTSVMIGVLLTGAVFL

60 cotton_GLEAN_10010706 gi|1336803 vacuolar H(+)-ATPase subunit A

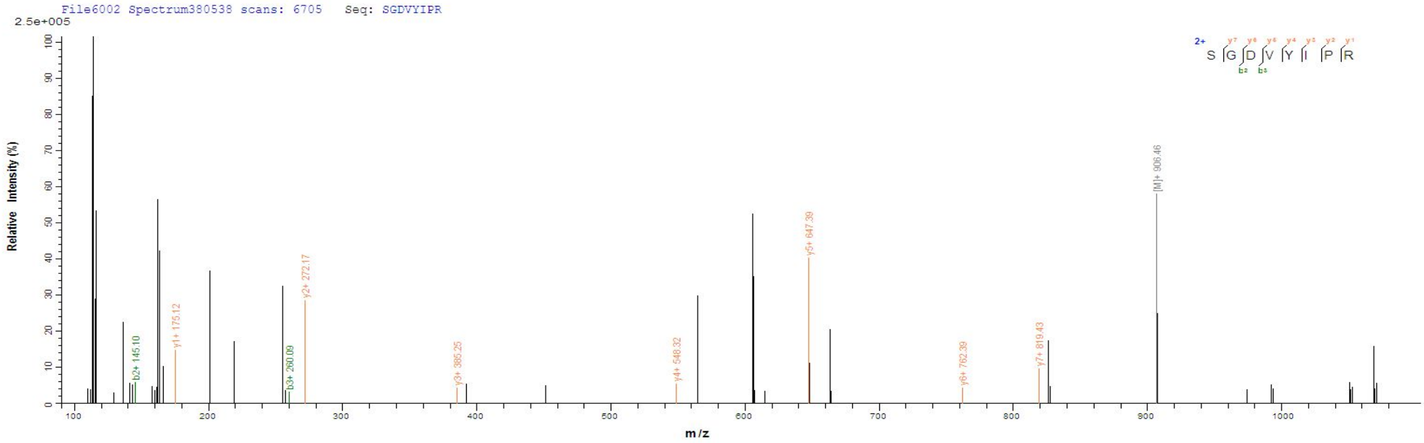

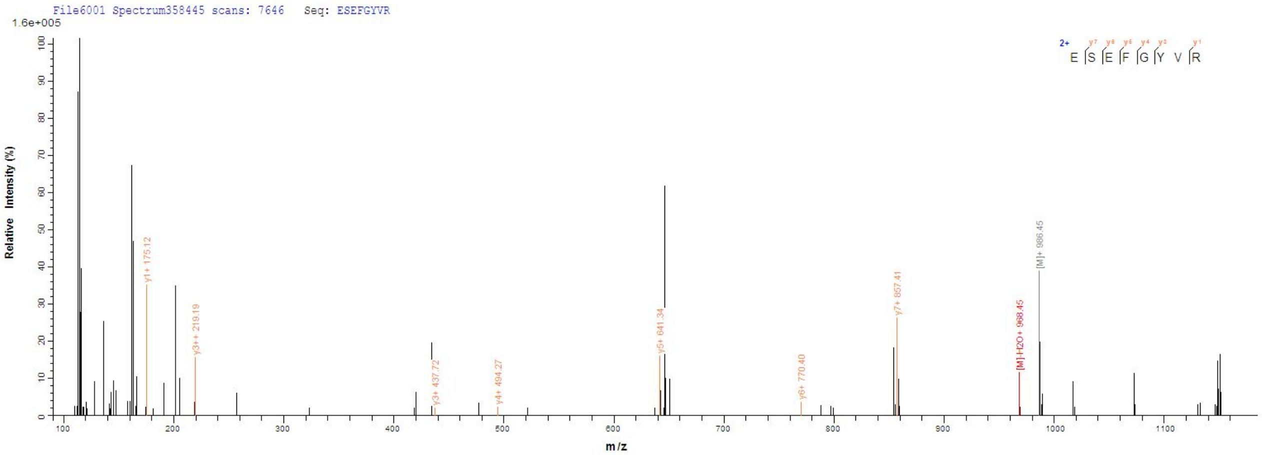

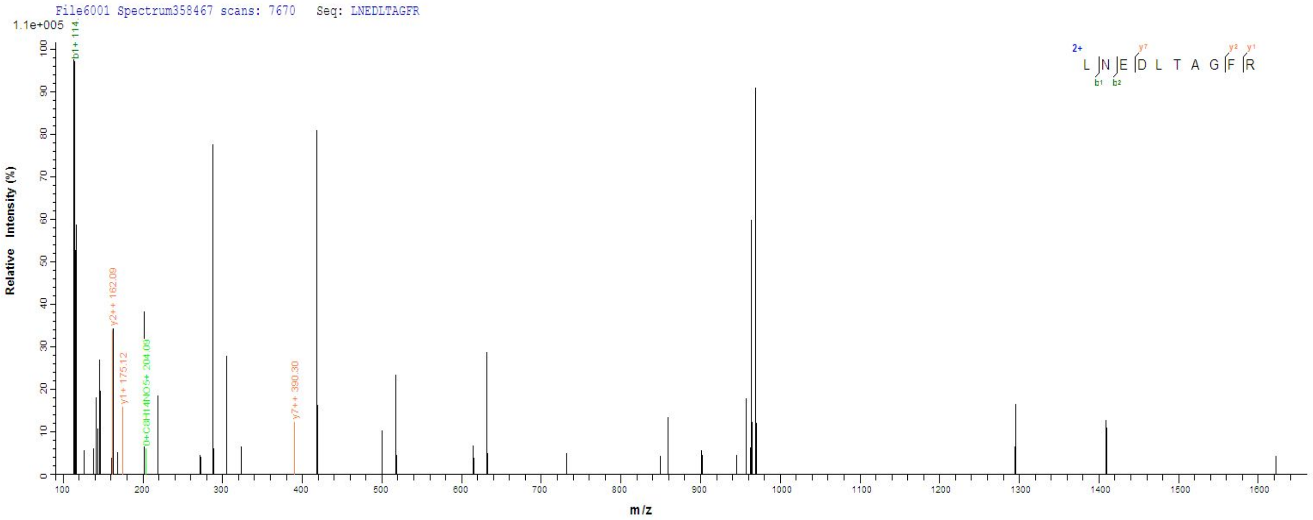

MPAVYGARLTTFEDSEKESEFGYVRKVSGPVVVADGMAGAAMYELVRVGHDNLIGEIIRLEGDSATIQVYEETAGLMVNDPVLRTHKPLSVELGPGILGNIFDGIQRPLKTIAKRSGDVYIPRGVSVPALDKDTLWEFQPKRIGEGDLLTGGDLYATVFENSLMQHHVALPPDAMGKITYIAPPGQYSLKDTVLELEFQGVKKQFTMLQTWPVRTPRPVATKLAADTPLLTGQRVLDALFPSVLGGTCAIPGAFGCGKTVISQALSKYSNSDAVVYVGCGERGNEMAEVLMDFPQLTMTLPDGREESVMKRTTLVANTSNMPVAAREASIYTGITIAEYFRDMGYNVSMMADSTSRWAEALREISGRLAEMPADSGYPAYLAARLASFYERAGKVKCLGGPERTGSVTIVGAVSPPGGDFSDPVTSATLSIVQVFWGLDKKLAQRKHFPSVNWLISYSKYSGALETFYEKFDPDFISIRTKAREVLQREDDLNEIVQLVGKDALAETDKITLETAKLLREDYLAQNAFTPYDKFCPFYKSVWMMRNIVHFNALANQAVEKAAGMDGQKITYSLIKHRLGDLFYRLVSQKFEDPAEGEEALVAKFKKLNEDLTAGFRALEDETR

61 Garb_00098 gi|2493146 V-type proton ATPase 16 kDa proteolipid subunit

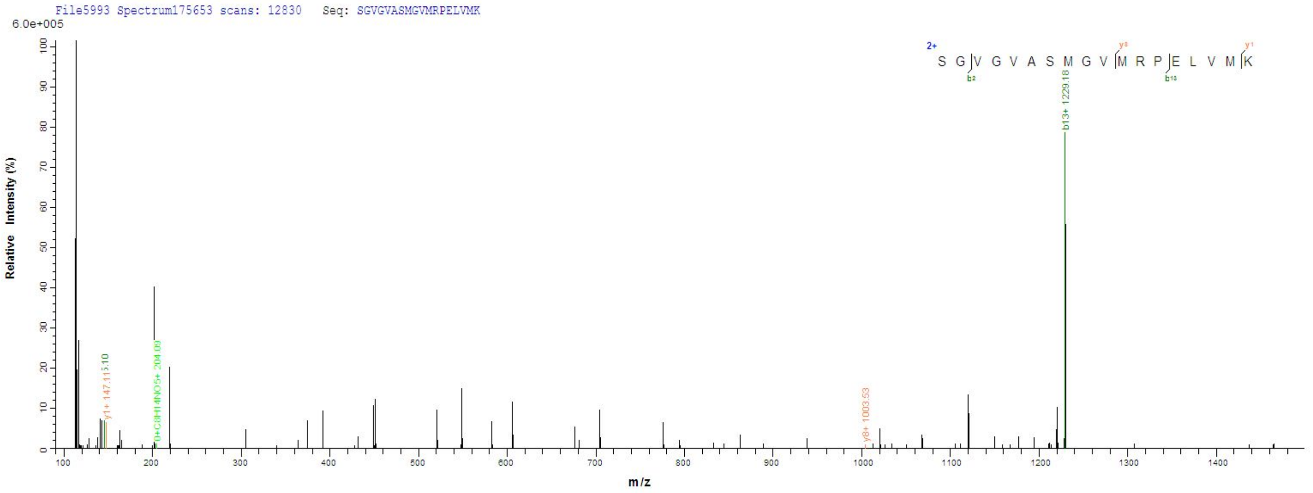

MSSTFSGDETAPFFGFLGAAAALVFSCMGAAYGTAKSGVGVASMGVMRPELVMKSIVPVVMAGVLGIYGLIIAVIISTGINPKAKSYYLFDGYAHLSSGLACGLAGLSAGMAIGIVGDAGVRANAQQPKLFVGMILILIFAEALALYGLIVGIILSSRAGQSRAE

62 cotton_GLEAN_10008387 gi|75273758 Cysteine-rich repeat secretory protein 38

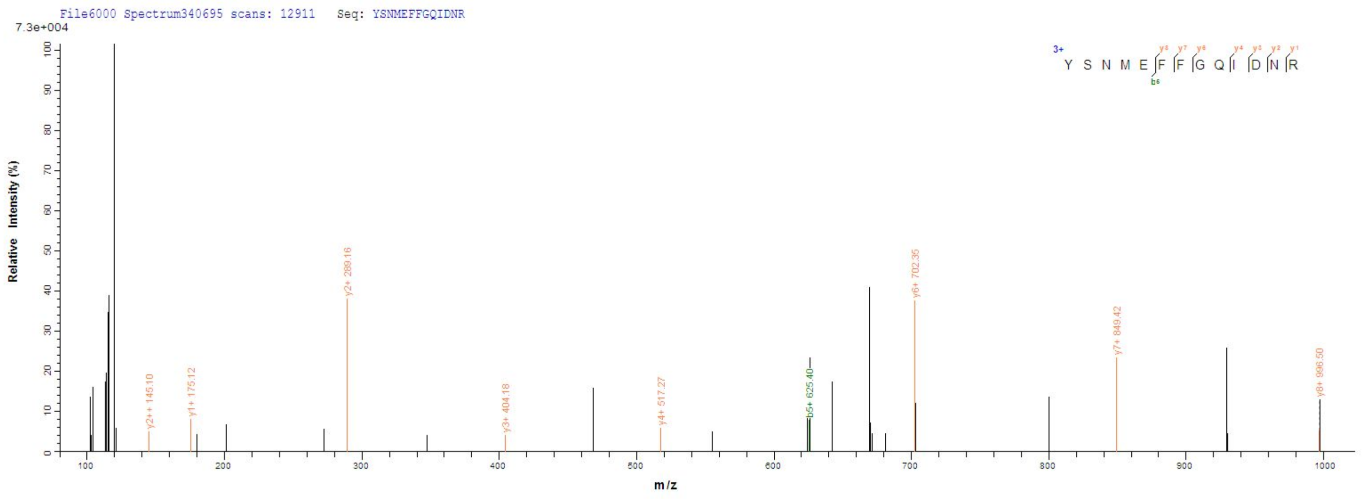

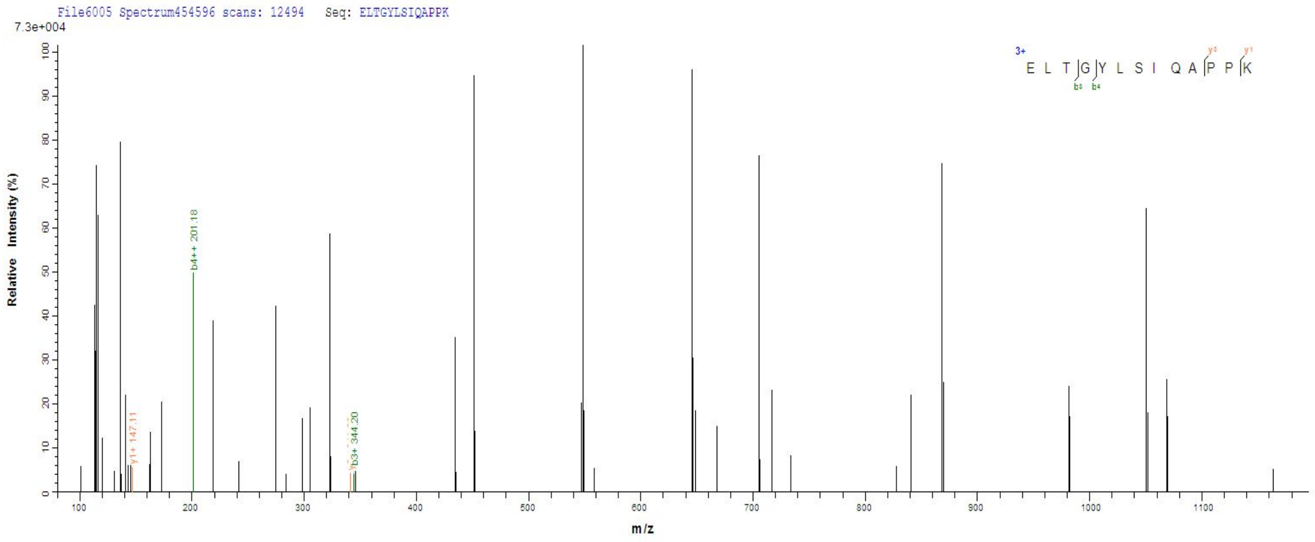

MSSSRVASFVYLLALTFIVQTAFGADPLFHFCSNSGNFSAYDPYEANLKELTGYLSIQAPPKGFGLGSIGQKPNQAYGLA
LCRGDVSTPDCKTCVVEAGSEIRKRCPYNKGAIIWYDNCLFKYSNMEFFGQIDNRNRFYMWNLNNVSEPQSFNAKTK
ELLSELANQAYSNPKMYAVGETELYGSNKLYGLTQCTRDLSSTECKKCLDGIIEELPTCCDGKEGGRVVGGSCNFRYEI
YPFVNA

63 cotton_GLEAN_10028780 gi|7105717 plasma membrane proton ATPase

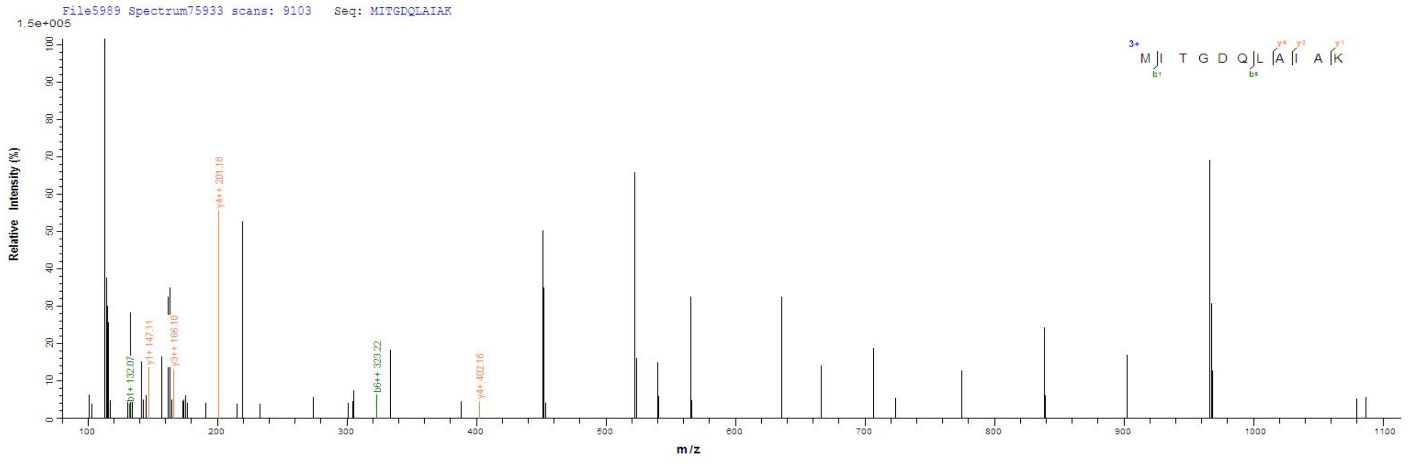

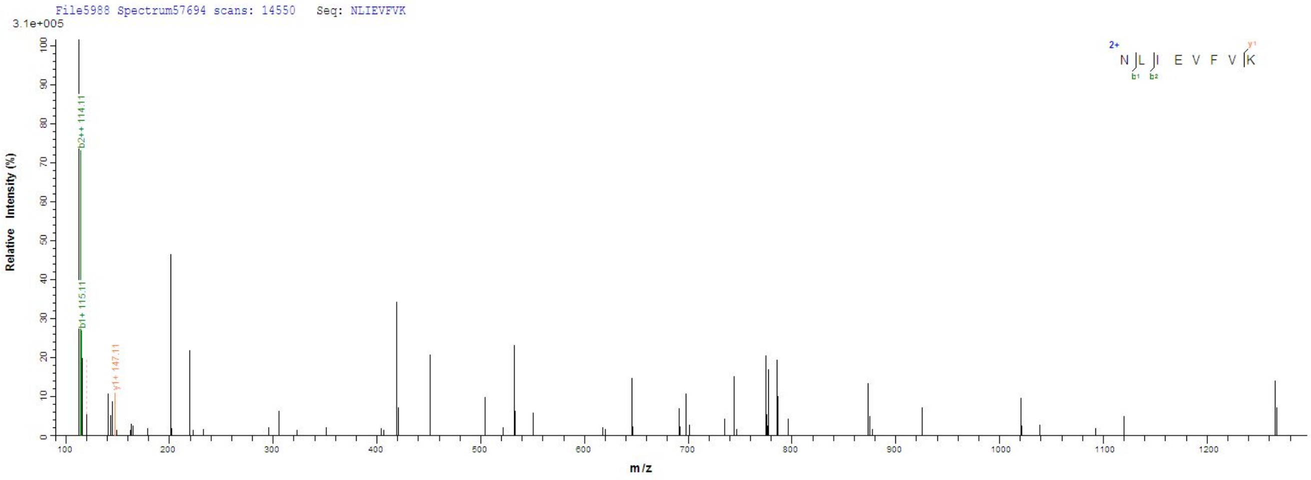

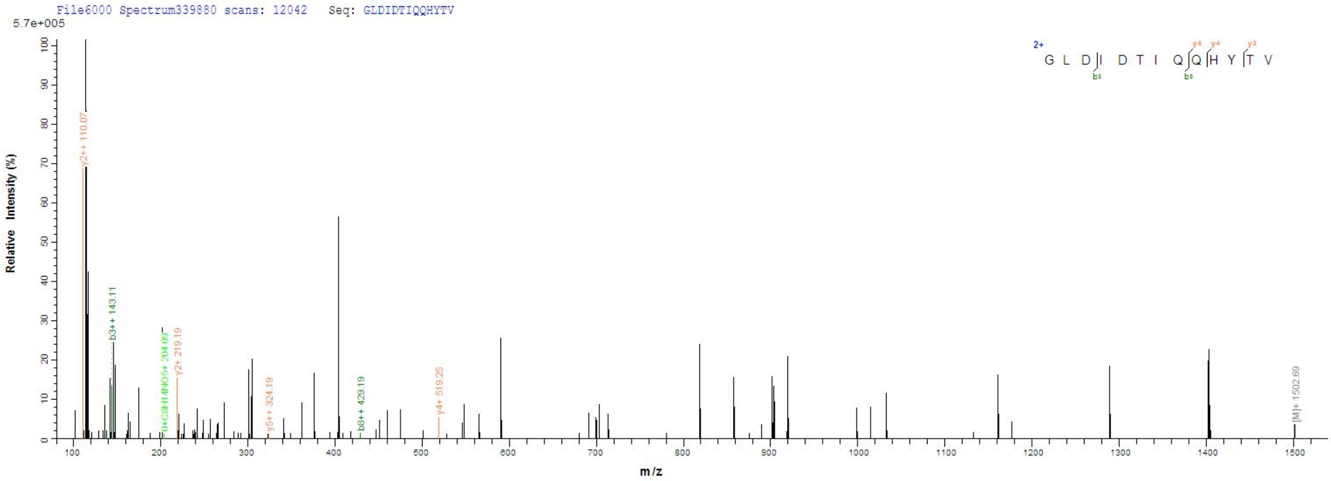

MAGISLEEIKNETVDLEKIPIEEVFEQLKCTREGLSSDEGVNRLQIFGPNKLEEKKESKILKFLGFMWNPLSWVMEAAAI
MAIALANGDGKPPDWQDFVGIVCLLVINSTISFIEENNAGNAAAALMAGLAPKTKVLRDGKWTEQEAAILVPGDIISIK
LGDIIPADARLLEGDPLKVDQSALTGESLPVTKNPGDEVFSGSTCKQGEIEAIVIATGVHTFFGKAAHLVDSTNQVGHFQ
KVLTAIGNFCICSIAIGMLVEIVVMYPIQHRKYRDGIDNLLVLLIGGIPIAMPTVLSVTMAIGSHRLSQQGAITKRMTAIEE
MAGMDVLCSDKTGTLTLNKLSVDKNLIEVFVKDADKEHVVLLAARASRTENQDAIDAAIVGMLADPKEARAGIREV
HFLPFNPVDKRTALTYIDSNGNWHRASKGAPEQILALCNAKEDLKKRVHSIIDKFAERGLRSLAVSRQQVPEKTKESAG
TPWQFVGLLPLFDPPRHDSAETIRQALHLGVNVKMITGDQLAIAKETGRRLGMGTNMYPSASLLGQDKDASIAALPVE
ELIEKADGFAGVFPEHKYEIVRKLQERKHICGMTGDGVNDAPALKKADIGIAVADATDAARSASDIVLTEPGLSVIISAV
LTSRAIFQRMKNYTIYAVSITIRIVFGFLFIALIWKFDFSPFMVLIIAILNDGTIMTISKDRVKPSPLPDSWKLKEIFATGIVL
GGYLALMTVIFFWVMHDTDFFSDKFGVRSLRERDHEMMGALYLQVSIVSQALIFVTRSRSWSYAERPGLLLVTAFIIAQ
LVATLIAVYANWGFARIKGIGWGWAGVIWLYSIVFYVPLDIMKFAIRYILSGKAWLNLLENKTAFTTKKDYGKEEREAQ
WALAQRTLHGLQPPETSNLFNDKNSYRELSEIAEQAKRRAEVARLRELHTLKGHVESVVKLKGLDIDTIQQHYTV

64 cotton_GLEAN_10025437 gi|224130846 multidrug/pheromone exporter, MDR family, ABC transporter family

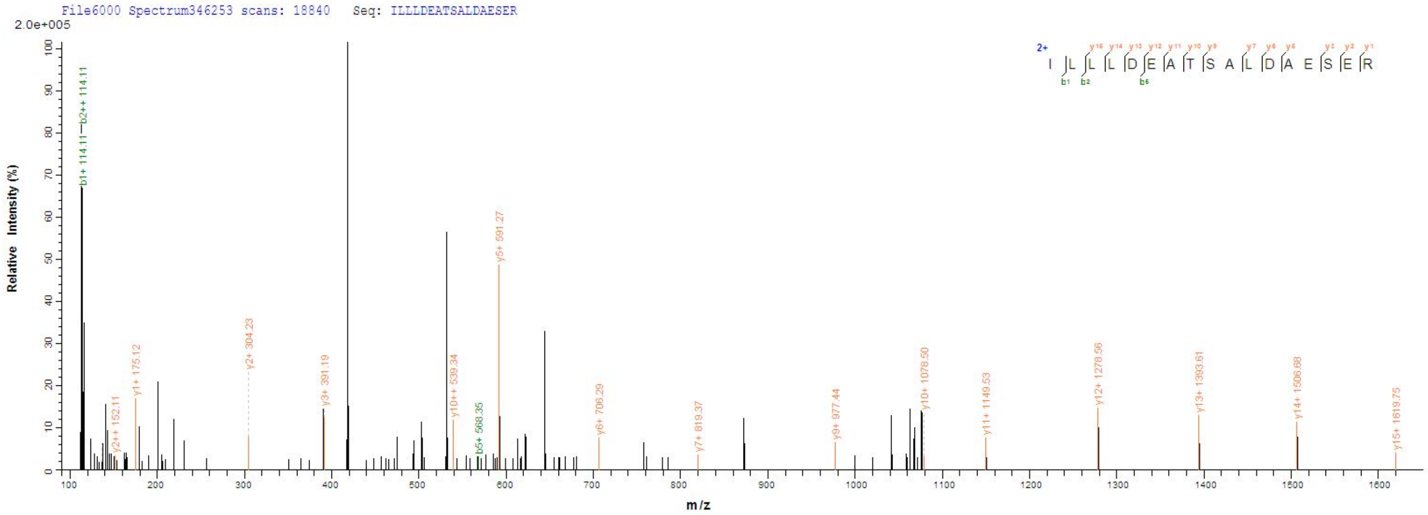

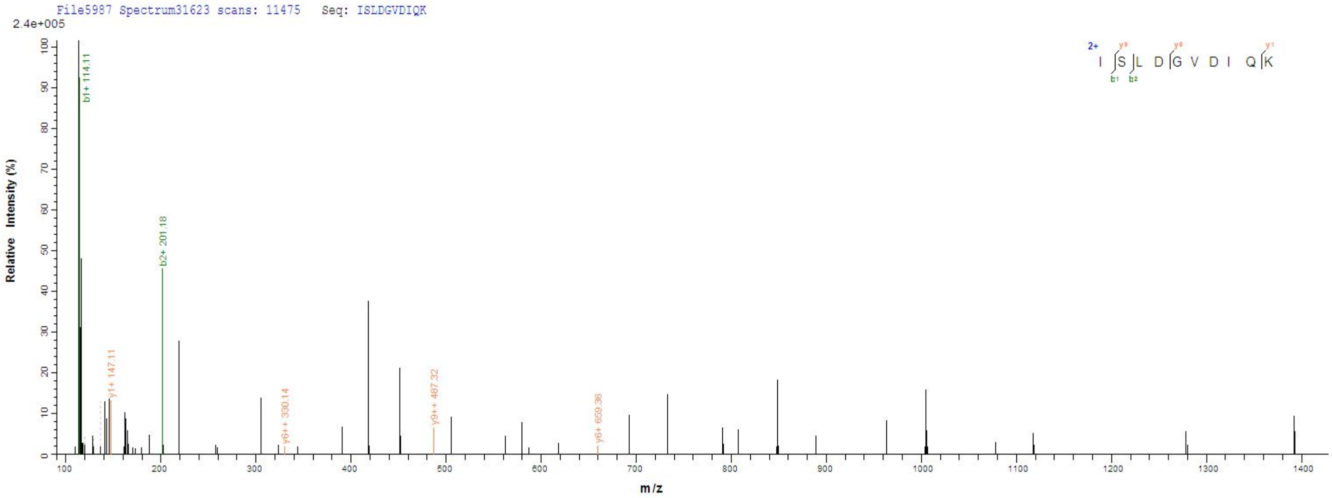

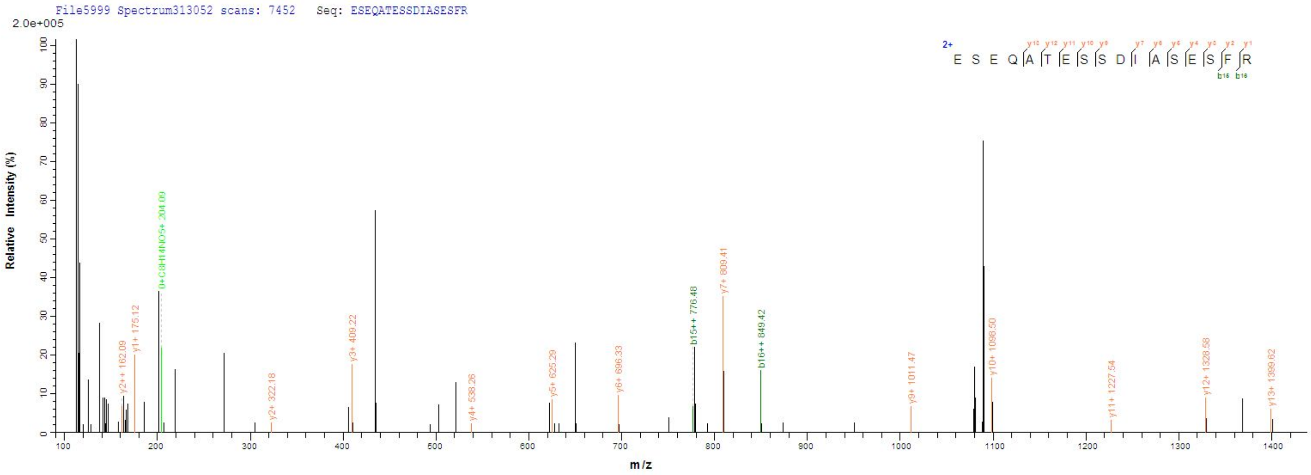

MDSENGFSDPTDLHEASTSKIQEEPDKVSGSNGDNLESKKVDDEKTNTVPFYKLFAFADSRDTLLMIVGTIGAVGNGIC
MPLMTILFGDLIDAFGENQNDDRVVDVVSRVALRFVYLAVGAGVAAFLQVTCWMVTGERQAARIRGLYLKTILRQDV
AFFDVETNTGEVVGRMSGDTVLIQDAMGEKVGKFIQLVSTFIGGFVIAFVQGWLLTLVMLSSIPPIVISGGVMALIVSK
MASRGQSAYAKAASVVEQTIGSIRTVASFTGEKQAISNYNKFLGAAYTSGVHEGFAAGLGLGVLFLVIFCSYSLAIWFG
ARMVLDRGYSGGDVINVIFAVLTGSMSLGQASPCVTAFAAGQAAAFKMFETIKRKPEIDSYDTRGKVLEDIRGDIELRD

VYFTYPARPDEQIFSGFSLSIQNGTTVALVGQSGSGKSTVISLIERFYDPHAGEVLIDGINLKEFQLRWIRGKIGLVSQEPV
LFTSSIRDNIAYGKEGATTEEIRAAAELANASKFIDKLPQGLDTMVGEHGTQLSGGQKQRVAIARAILKDPRILLLDEAT
SALDAESERVVQEALDRIMGNRTTVIVAHRLSTVRNADMIAVIHRGKMVEKGSHSELLQDHEGAYSQLIRLQEVNKES
EQATESSDIASESFRRSSLKKSLKRSIKKAPKVSVRRLAYLNKPEIPVILLGTIAAAANGVIFPIFGILISSVIDTFFKPPHEL
REDSRFWALIFLALGAAAFVVCPAQNYFFSIAGSKLIQRIRSMCFEKVVRMEVGWFDEPENSSGAIGARLSADAASIRA
LVGDALAQLVQNTSSAISGLVIAFVACWQLAFIVLVLLPLIAINGYIQVKFMKGFSADAKLMYEEASQVANDAVGSIRTV
ASFCAEEKVMQLYKKKCEGPMKTGIKQGLISGTGFGVSFFFLFSVYATSFYAGAQLVEHGYTTFRDVFQVFFALTMAAI
GISQSSSFAPDSGKAKSAAASIFAIIDRESKIDPSDESGMKLENVKGDIELHHVSFKYPSRPDIQILRDLSLSIRSGKTVALV
GESGSGKSTVISLLQRFYDPDSGRISLDGVDIQKLQLKWLRQQMGLVSQEPVLFNDTIRANIAYGKGGNATEAEILAAS
ELANAHKFISSLQQGYDTVVGERGVQMSGGQKQRIAIARAIVKSPQILLLDEATSALDAESERVVQAALDRVVVNRTT
VVVAHRLSTIKNADVIAVVKNGVVVEKGKHDTLINIKDGFYASLVALHMSASTA

65 cotton_GLEAN_10022598 gi|292653531 aquaporin TIP1;7

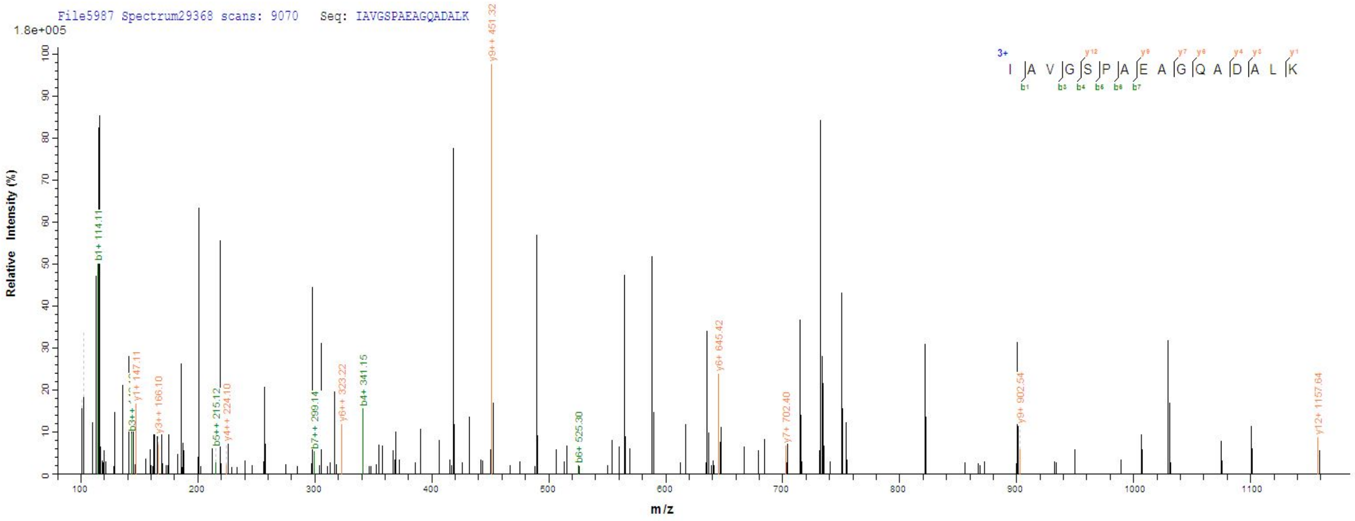

MPISRIAVGSPAEAGQADALKAALAEFISVLIFVFAGEGSGMAFNKLTDDGSSTPAGLVAAALAHALALFVAVSVGANIS
GGHVNPAVTFGAFVGGHITLVRSILYWIAQLLGSVVACLLLKFSTGGMTTSAFSLSSGVGAWNAVVFEIVMTFGLVYTV
YATAVDPKKGNIGIIAPIAIGFIVGANILAGGAFDGASMNPAVSFGPAVVSWTWDNHWVYWLGPFIGSAIAAIVYEVFFI
APSTYEELPSADF

66 cotton_GLEAN_10019420 gi|292653547 aquaporin TIP2;5, partial

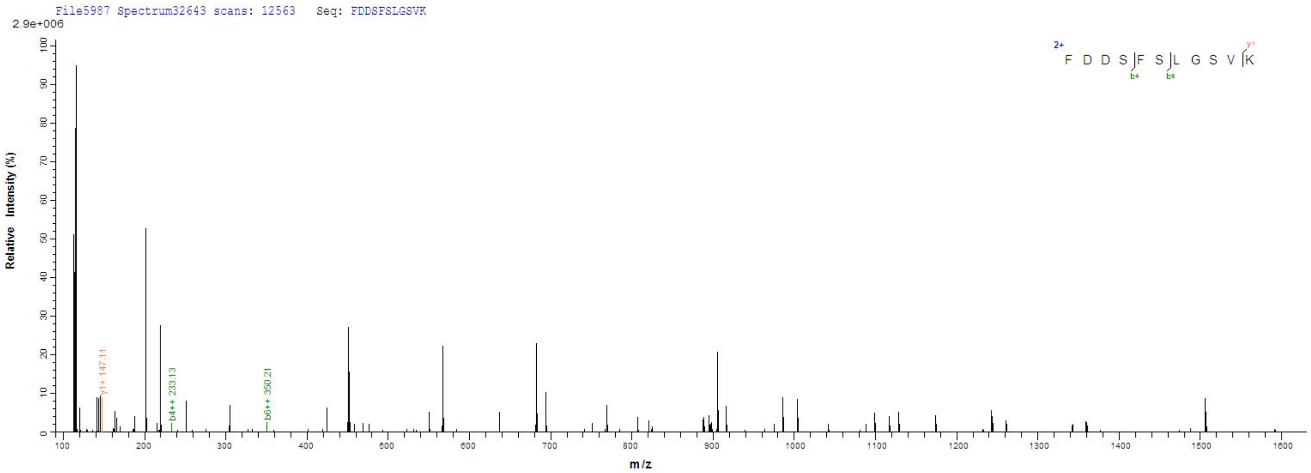

MAG
IAFGRFDDSFSLGSVKAYLAEFISTLVFVFAGVGSAIAYNKLTTDAALDPDGLVAIAVCHGFALFVAVAIGANISGGHVNP
AVTFGLALGGQITILTGIFYWIAQLLGSIVACFLLKAVTGGLTVPIHGLGAGVGAIQGVVMEIIITFALVYTVYATAADPK

KGSLGTIAPIAIGFIVGANILAAGPFSGGSMNPARSFGPAVASGDFNGIWIYWVGPLIGGGLAGLIYGNVFMNSDHAPLSNDF

67 Garb_19337 gi|461929 Probable aquaporin TIP-type

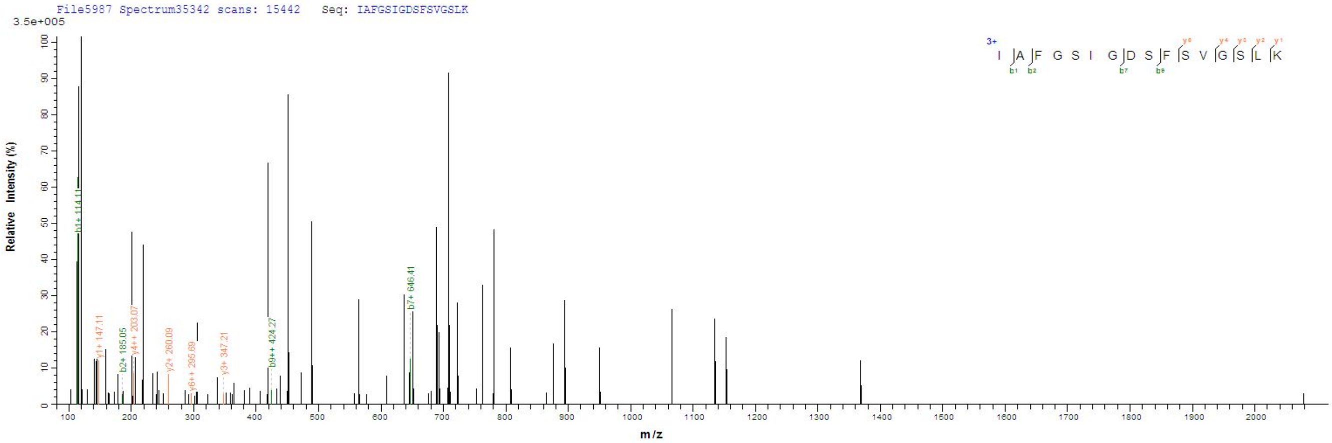

MVKIAFGSIGDSFSVGSLKAYLAEFIATLLFVFAGVGSAIAYGKLTSDAALDPPGLVAIAVAHALALFVGVAIAANISGGHLNPAVTFGLAIGGNITILTGLFYWVAQCLGSIVACLLLQFVTNGLSVPTHSVASGMNAVGGVVMEIVITFALVYTVYATAADPKKGSLGIIAPIAIGFIVAVVSGNFADNWIYWVGPLVGGGLAGLIYGDVFIGSYSAAPASEDYA

68 cotton_GLEAN_10018594 gi|300793602 TPA: TPA_inf: aquaporin TIP1;4

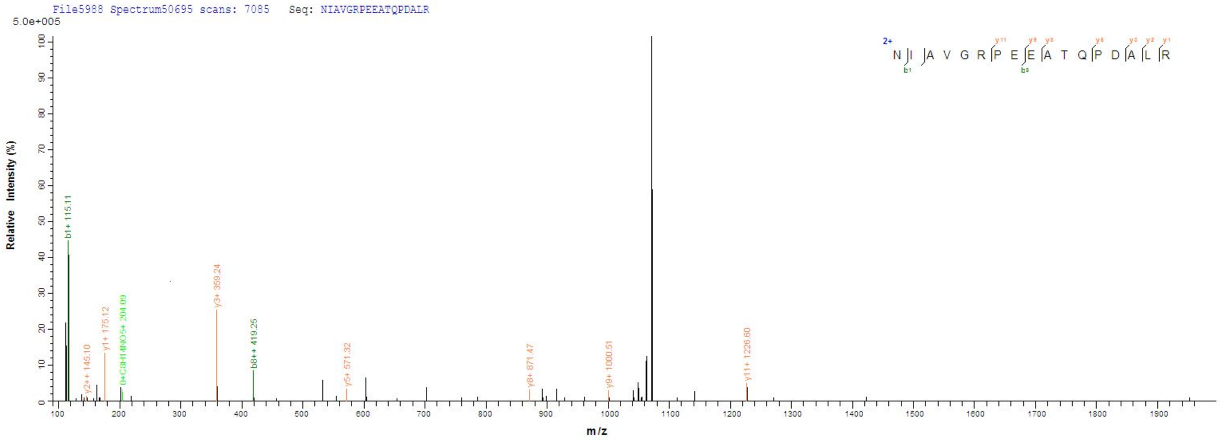

MPIRNIAVGRPEEATQPDALRAALAEFISTLIFVFAGSGSGMAFNKLTDNGATTPAGLVAASIAHGLGLFVAVSVGANISGGHVNPAVTFGAFVGGNISLLRGILYWIAQLLGSTVACLLLKFATSDMTVPAFGLSSGVGVWNAVVFEIVMTFGLVYTVYATAVDPKKGDLGVIAPLAIGFIVGANILAGGAFDGASMNPAVSFGPALVSWSWDWIYWLGPLIGGGLAGLIYEFAFINQSHEQLPPTDY

69 Garb_09969 gi|292653535 TPA: TPA_inf: aquaporin TIP1;4

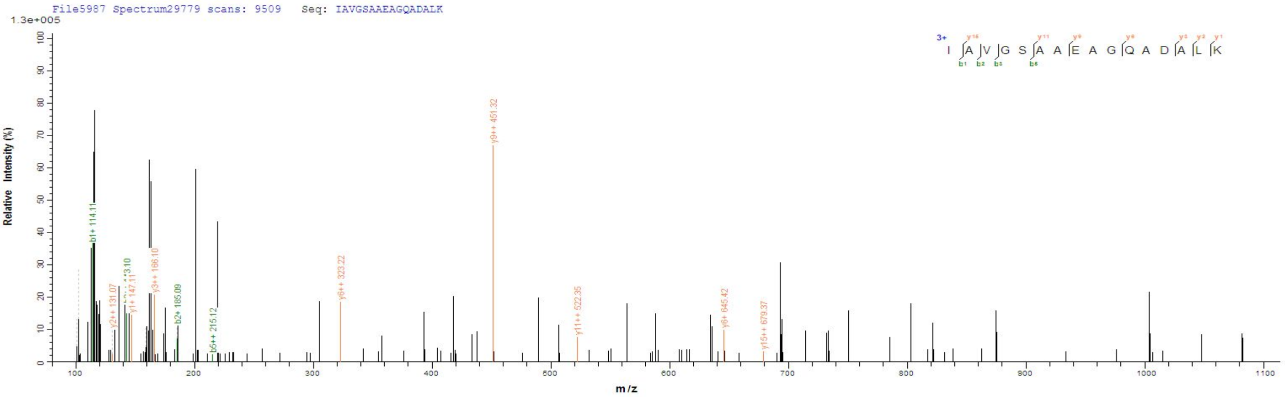

NNKKMPISRIAVGSAAEAGQADALKAALAEFISMLIFVFAGEGSGMAFNKLTDNGSTTPAGLVAASVAHAFALFVAVSV GANISGGHVNPAVTFGAFVGGHITLVRSILYWIAQLLGSVVACLLLKFSTGGLTTSAFALSSGVGAWNAVVFEIVMTFGL VYTVYATAVDPKKGNIGIIAPIAIGFIVGANILAGGAFDGASMNPAVSFGPAVVSWTWDNHWVYWLGPFVGSAIAAIVY EVFFIAPDTHEQLPTSEF

70 cotton_GLEAN_10022435 gi|300793598 aquaporin PIP2;10

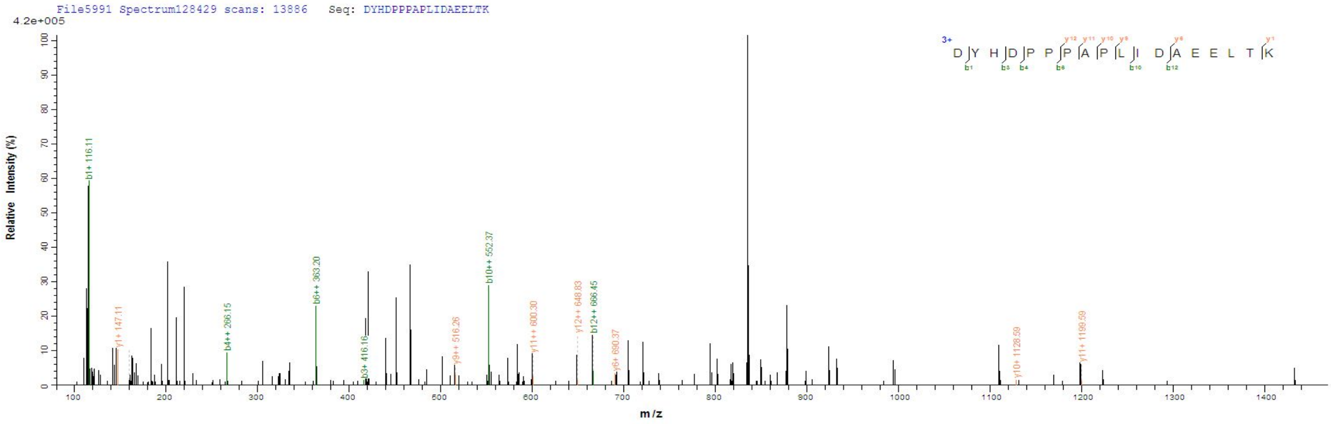

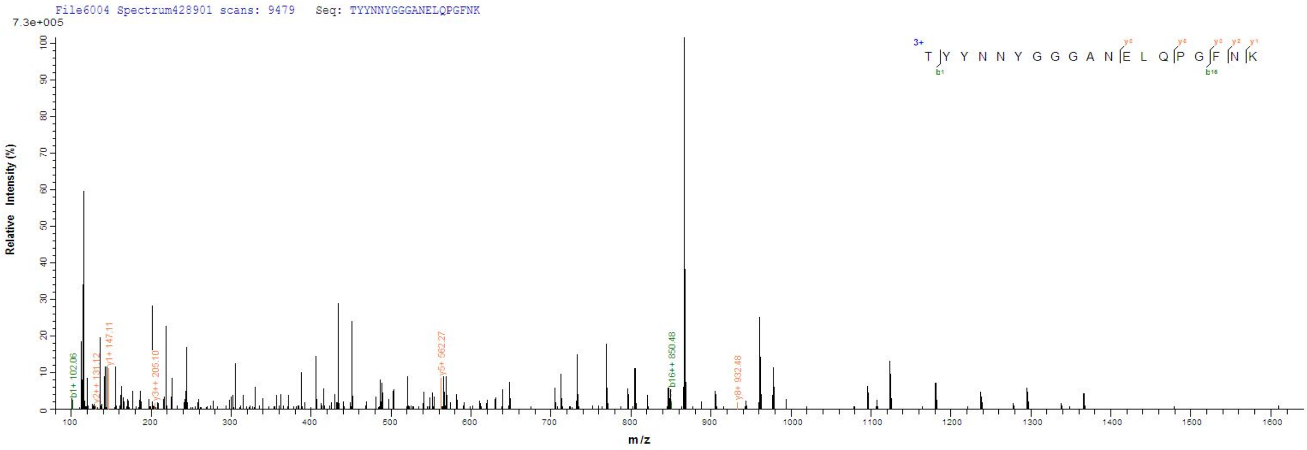

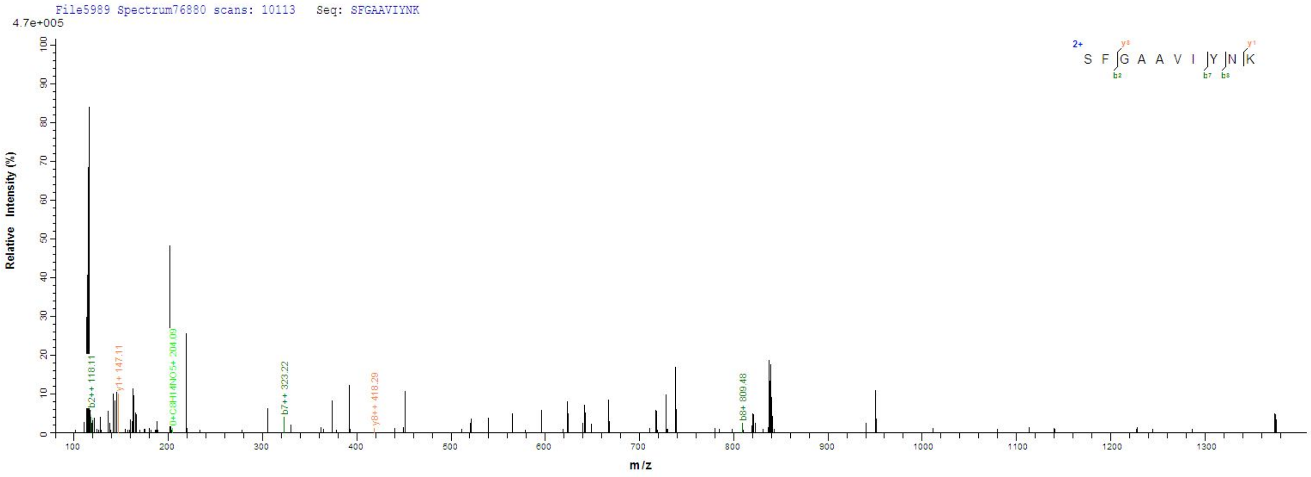

MTKDIETAEQHGGEFSAKDYHDPPPAPLIDAEELTKWSFYRAVIAEFIATLLFLYVTVLTVIGYKVQTDPAKNTVDPDCG GVGILGIAWAFGGMIFILVYCTAGISGGHINPAVTFGLFVGRKVSLIRALMYMVAQCLGAICGCGLVKAFQKTYYNNYG GGANELQPGFNKGTGLGAEIIGTFVLVYTVFAATDPKRNARDSHVPVLAPLPIGFAVFMVHLATIPVTGTGINPARSFGA AVIYNKEKAWDDQWIFWVGPFIGAAAAAFYHQYILRAAAIKALGSFRSNA

71 Garb_04584 gi|256568429 PIP protein

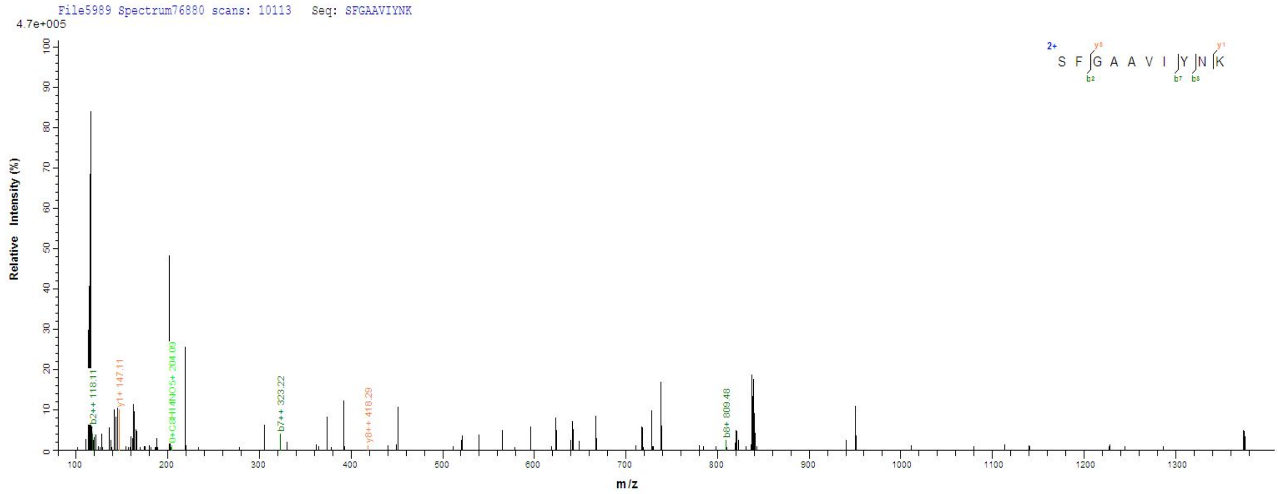

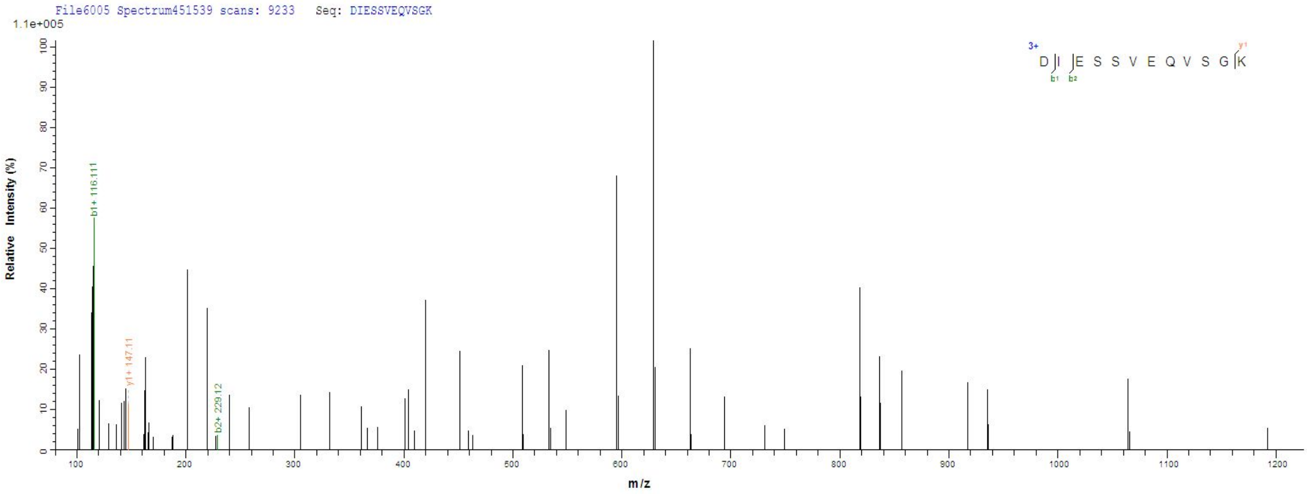

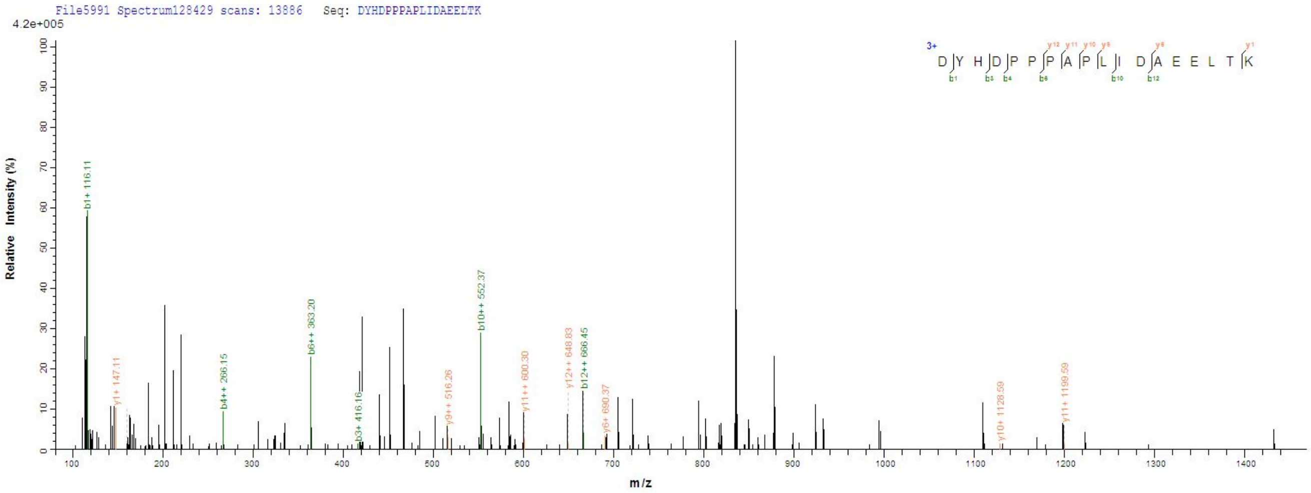

MGKDIESSVEQVSGKDYHDPPPAPLIDAEELTKWSFYRAVIAEFIATLLFLYVTVLTVIGYKVQTDPLKNTVDPDCGGV
GILGIAWAFGGMIFILVYCTAGISGGHINPAVTFGLFLGRKVSLIRAIMYMVAQCLGAICGCGLVKAFQKTYYNNYGGG
ANELQSGFNKGTGLGAEIIGTFVLVYTVFSATDPKRNARDSHVPVLAPLPIGFAVFMVHLATIPVTGTGINPARSFGAAVI
YNKEKAWDDQWIFWVGPFIGAAIAAFYHQYILRAAAIKAFGSSRSN

72 Garb_13025 gi|164668308 PIP2 protein

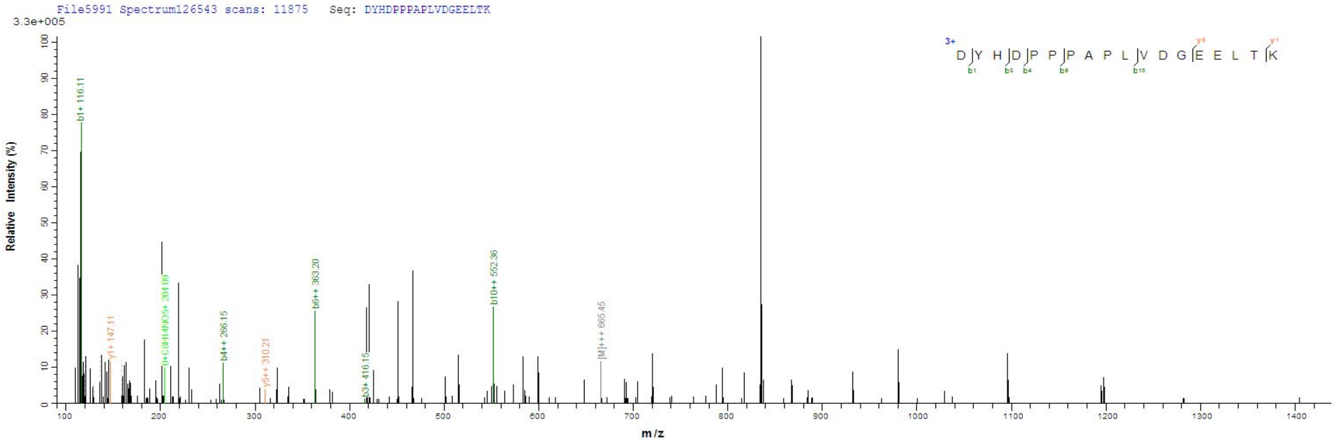

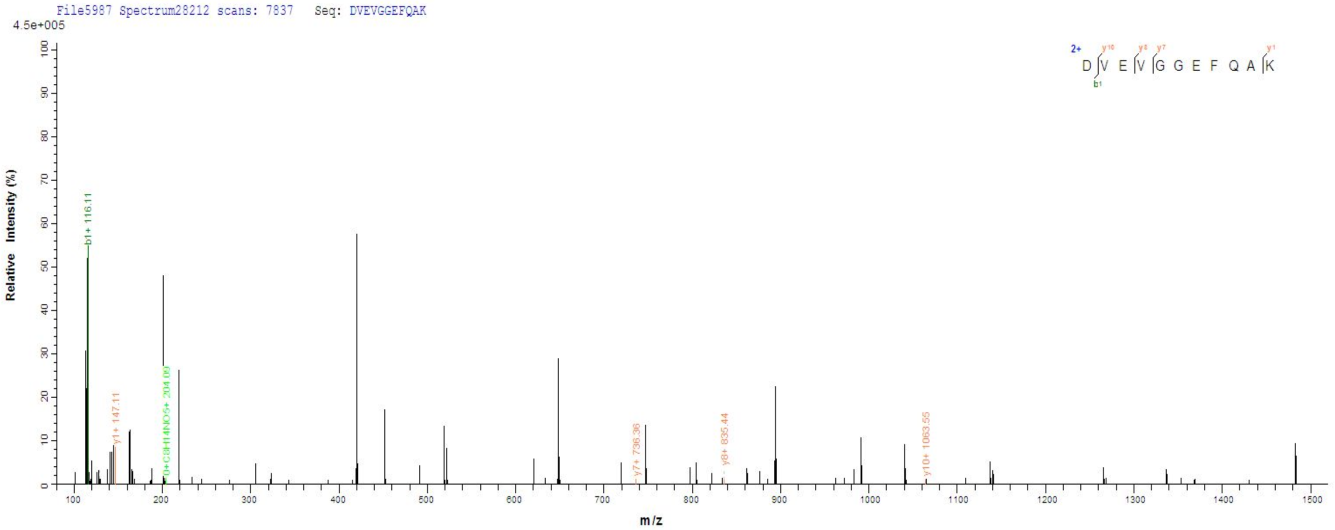

MAKDVEVGGEFQAKDYHDPPPAPLVDGEELTKWSFYRAVIAEFIATLLFLYITVLTVIGYKSQVDPDKGSDECGGVGIL
GIAWAFGGMIFILVYCTAGISGGHINPAVTFGLFLARKVSLVRAIFYMAAQCLGAICGCGLVKAFQKSYYNKYGGGANS
LADGYSTGTGLAAEIIGTFVLVYTVFSATDPKRNARDSHIPVLAPLPIGFAVFMVHLATIPITGTGINPARSFGAAVIYNQD
KPWDDHWIFWVGPFIGAAIAAIYHQFILRAAAVKALGSFRSSSAM

73 Garb_13932 gi|118132686 PIP1 protein

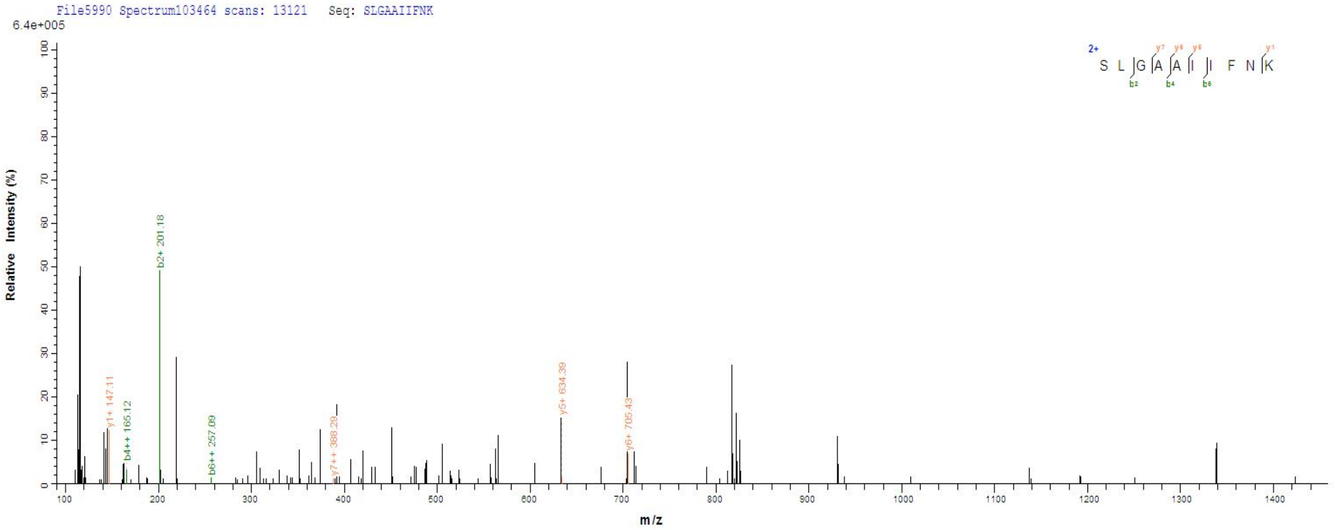

MEGKEEDVRLGANKFTERQPIGTAAQSQDDGKDYTEPPPAPFFEPGELTSWSFYRAGIAEFVATFLFLYITILTVMGVVK
EKTKCPTVGIQGIAWAFGGMIFALVYCTAGISGGHINPAVTFGLFLARKLSLTRAIFYMVMQCLGAICGAGVVKGFMGK
TQYGMLGGGANSVAHGYTKGDGLGAEIVGTFVLVYTVFSATDAKRSARDSHVPILAPLPIGFAVFLVHLATIPITGTGIN
PARSLGAAIIFNKDKGWDDHWIFWVGPFIGAALAALYHVVVIRAIPFKSK

74 cotton_GLEAN_10040184 gi|363807628 probable leucine-rich repeat receptor-like protein kinase At5g49770-like precursor

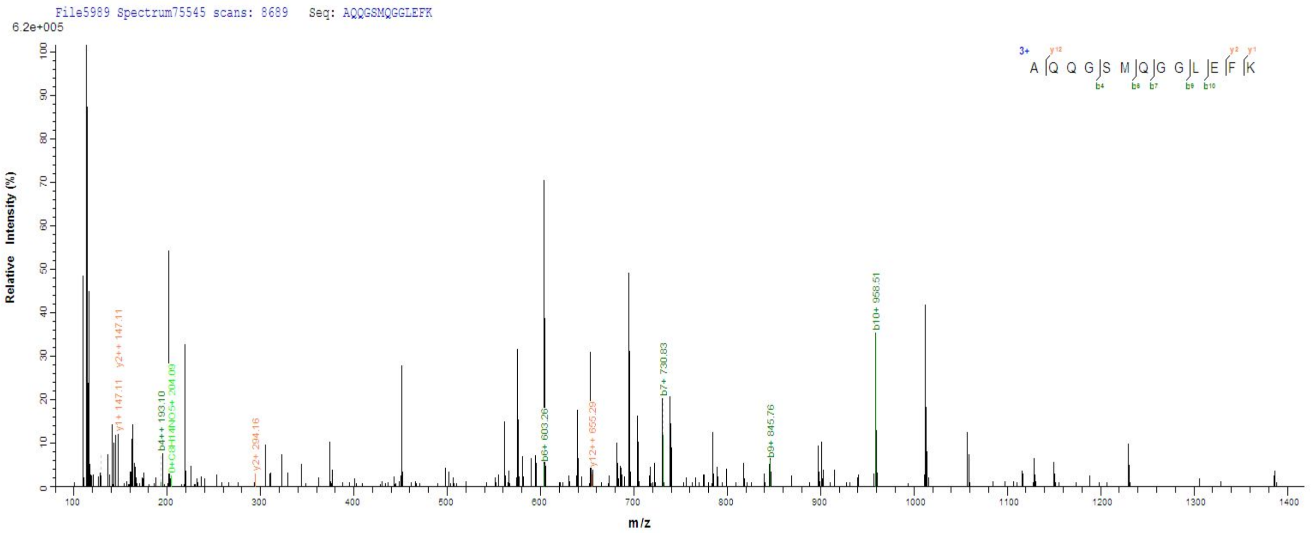

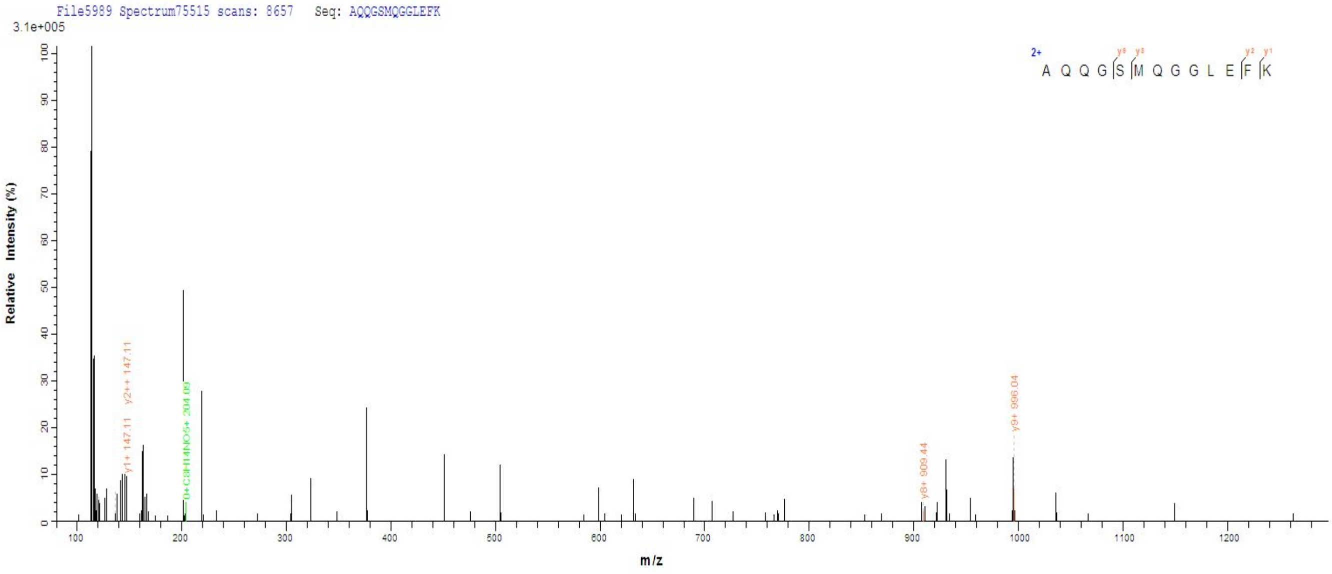

VQDLYLSNNKLTGPLPNLTGMSSLNTLYLSNNSFDSSDVPSWFPTLLSLTTLMMESTQLKGQIPASFFNLPQLQTVVLKQ
NELDGSFDIGPSFSNQLQIINLQGNSITSFNNTGGPISFDIVLVDNPVCQETGAGANDYCSLPQPDSSSVYTTAPMNCVPN
SCGSGQISSPRCICAYPYTGTLQFRGLYFSNLRNGTPYESLEQNLTQFFRFPELLVDTVSLSNPRMDQHLYLLLDLYLFPY
GQDRFNTSGISKIASAFSSQDYKPPEQYFGPYVFTGAEYEYFSDGPAHSNKSSAGIAIGAAVGASVLFILLVVAGIYAYRQ
RKRADRATKESNPFAHWDPKKSSGSIPQLKGARCFSFEELKKYTKKFSEANDIGSGGYGKVYRGTLPTGELVAIKRAQ
QGSMQGGLEFKTEIELLSRVHHKNVVSLLGFCFERGEQMLVYEYIPNGSLSDSLSGKSGIRLDWPRRLKIALGAAKGVA
YLHELANPPIIHRDIKSTNILLDERLNAKVADFGLSKPMGDSEKGHVSTQVKGTMGYLDPEYYMTQQLTEKSDVYSFG
VLMLEIITARRPIERGKYIVREMRMSMDKTKSLYNLQQILDPAIGFGTSSKGLERFVELAMRCVEESGADRPTMGEVVK
EIENIMQMDGMNPNAESASSSATYEDATKGADLHPYDNESFAYSGAFPHSAAKIEPH

75 cotton_GLEAN_10031546 gi|1702983 Auxin-repressed 12.5 kDa protein

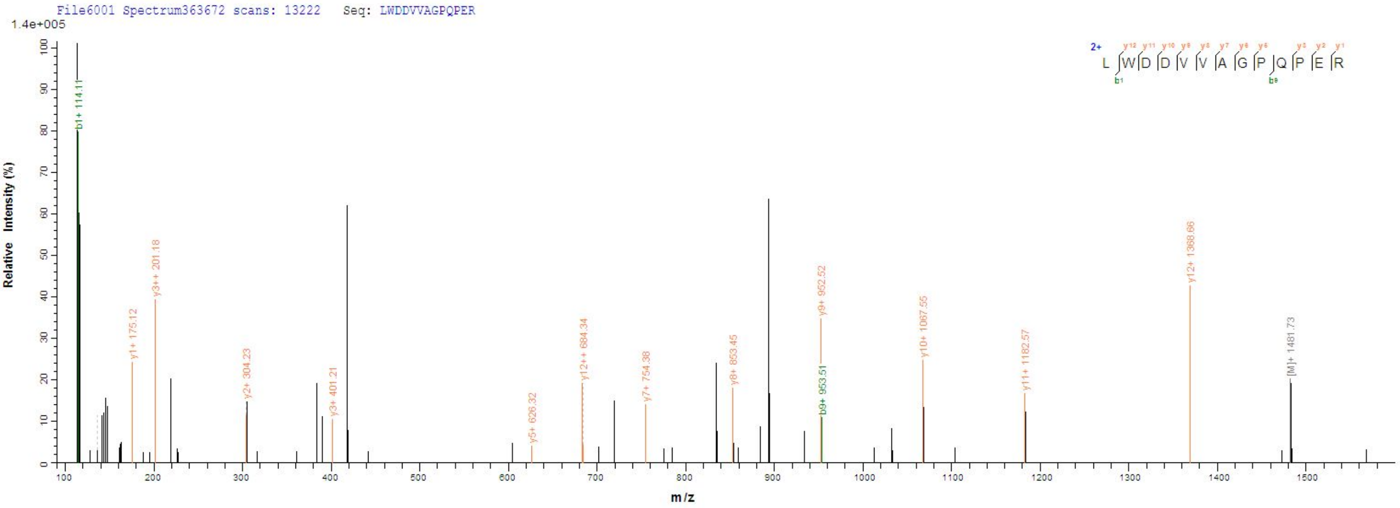

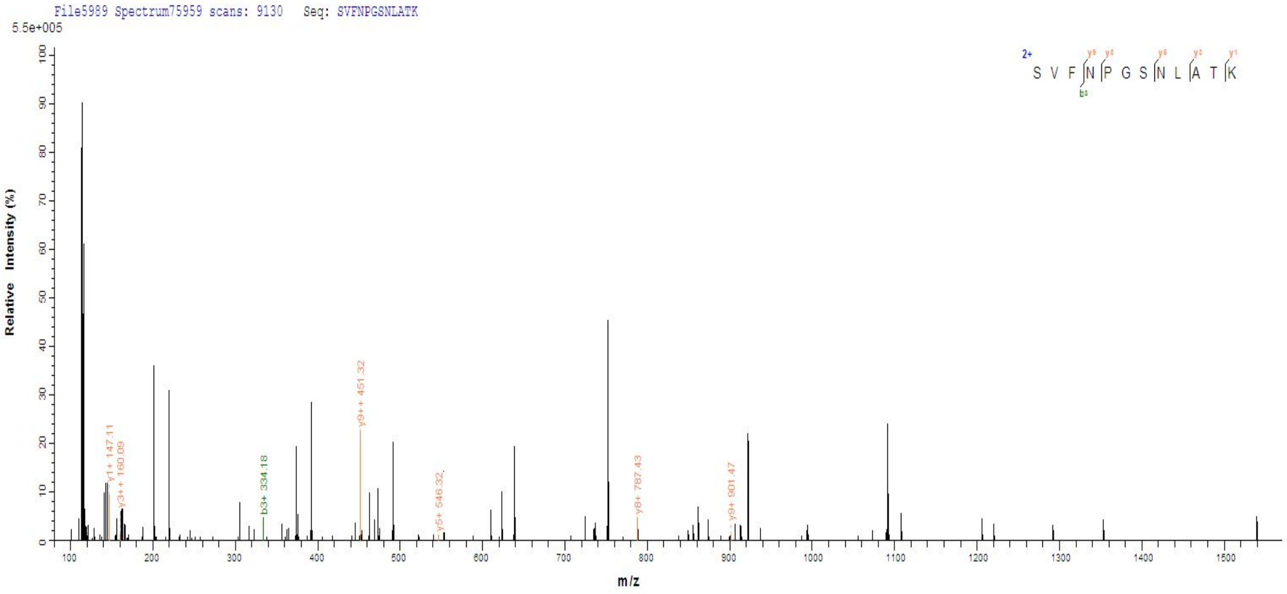

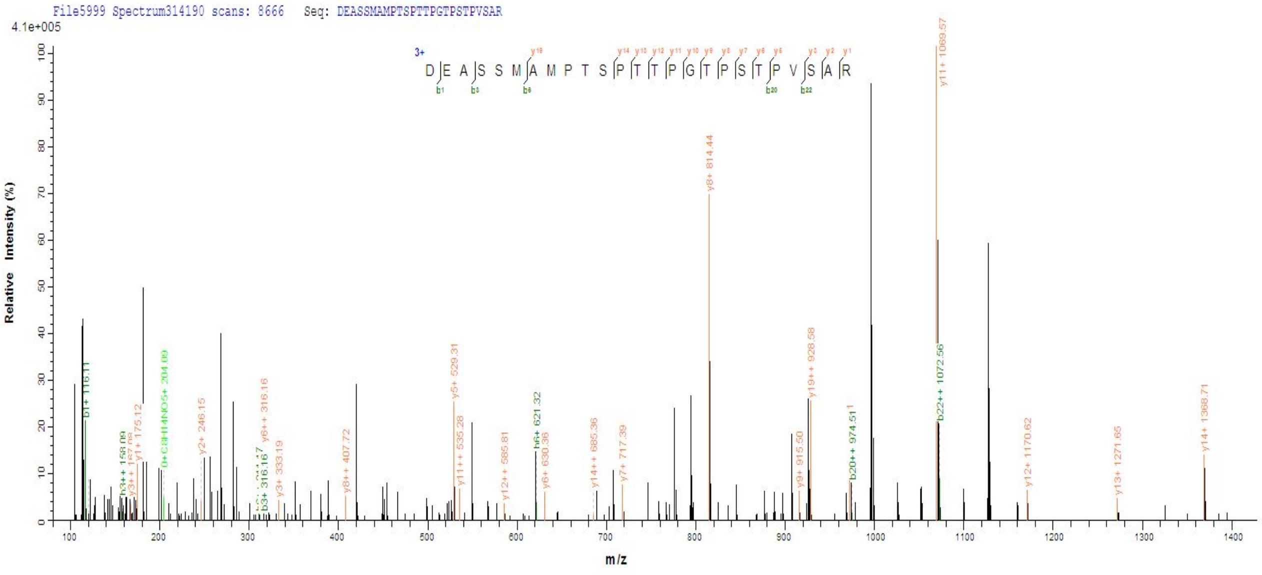

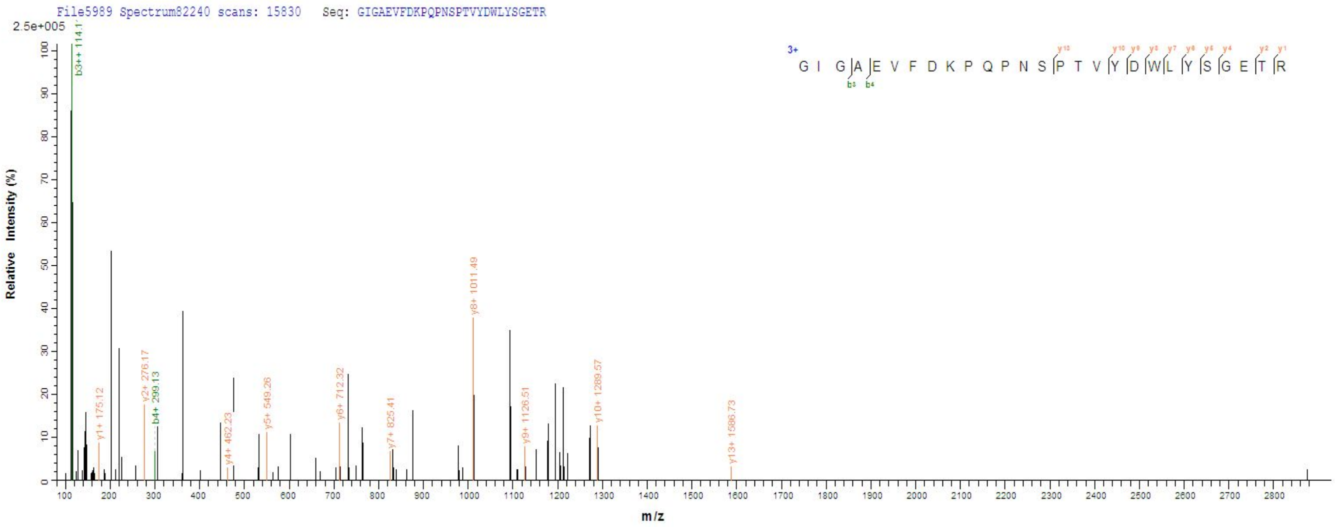

MVLLEKLWDDVVAGPQPERGLGRLRKITTTPPLSTKDEASSMAMPTSPTTPGTPSTPVSARRDNVWRSVFNPGSNLATKGIGAEVFDKPQPNSPTVYDWLYSGETRSKHHR

76 Garb_23718 gi|349504495 leucine rich repeat-containing protein

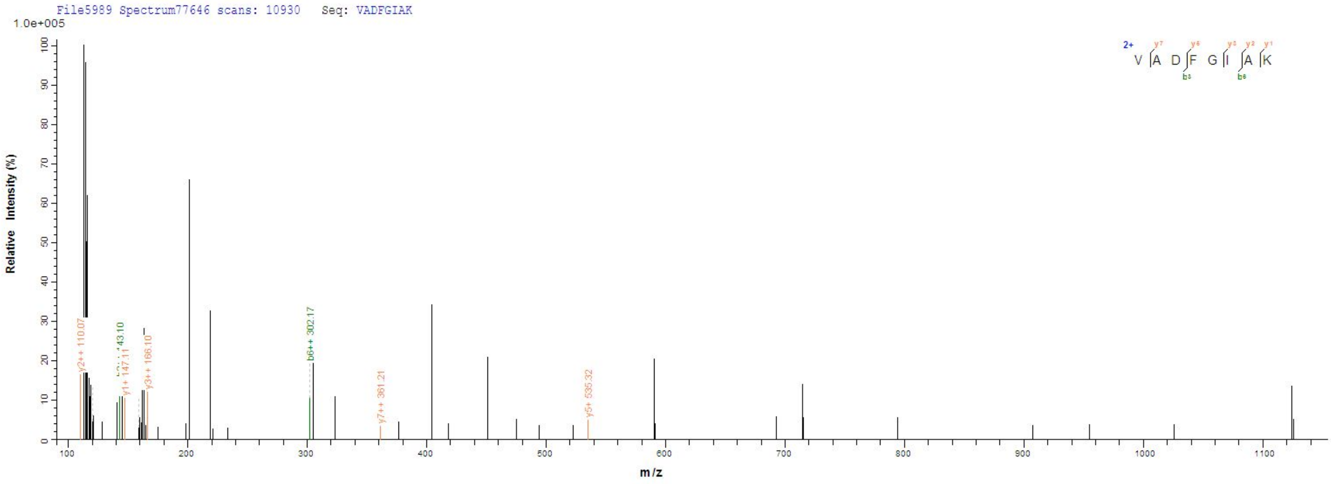

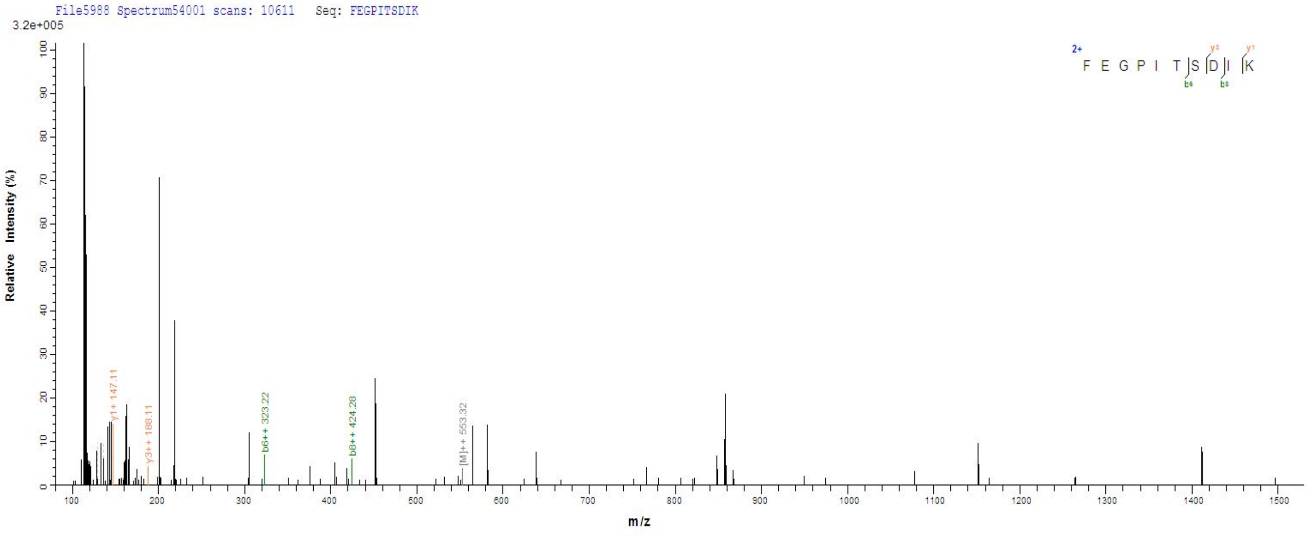

CSIEGKIPPSIGDLTELKDLELQLNYLSGAIPSEIGKLRKLWQLELYGNELTGKLPVGLRNLTSLEYFDASINYLEGDISEVKYLTNLVSLQLFMNRFNGGVPPELGEFKKLVNLSLYTNMLTGPLPQKLGSWADFDYIDVSENLLTGLIPPDMCKKGTM

RGLLMLQNRFTGEIPTTYASCATMKRFRVSNNSLSGIVPAGIWGLPQVEIIDIAYNRFEGPITSDIKNAKEIGILSAEFNRL
SGEVPKEISGATSLVKIELNDNQMSGEIPDGIGELKALSSLKLQNNMFSGPIPDSLGSCASISNINMANNSLSGKIPSSLGS
LPTLNSLDLSRNELSGQIPKELGLLKNLQQLELYYNQHLSGSIPEELGNLTELVDLDMSVNRLSGSIPESLCRLPKLQVL
QLYNNSLTGEIPGVIAESTTLTMLSLYENFLSGQIPQNLGKSSAMVLLDLSENKLSGPLPAELCRGGRLLYLLVLDNKFS
GKLPDSYANCKSLIRFRVSKNYLEGAIPEDLTGLPHAAIIDLADNSFTGPFPTSIGNAKNLSELFMQNNKLSGFLPPEISG
AINLMKIDLSNNLLSGPIPTEIGDLKKLNLLMLQGNKLSSSIPSSLSLLKSIGVVDLSNNHLTGNIPESLGELSSNTINFSN
NELYGPIPLSLIKDGLVESFSGNPGLCTPVEVQSFPKCSHKHNQKKRNSVWAIMISVTVFTIGAILFLRRRYSKQRGVVE
HEHDETLSSSFFPYNVKNFHRICFDQHEILEAMIEKNIVGHGGSGTVYRIGLRGGEVVAVKRLWSRTAKDTTPEDQLIFD
KGLKTEVETLGSIRHKNIVKLYSYITNFDRKMLVYEYMPNGNLWDALHKGWIHLDWPIRHQIALGVAQGLAYLHHDL
LTPIIHRDIKSTNILLDANYEPKVADFGIAKVLQARGGKDSIITVIAGTYGYLAPEYAYSSKATTKSDVYSFGVVLMELIT
GKKPVEADFGENKNIIYWVSTKLDTKEGVMEVLDKRVSGSFKDEMIQVLRIAMRCTYRNPTQRPPMNEVVQLLI

77 Garb_05514 gi|1346675 Nucleoside diphosphate kinase B

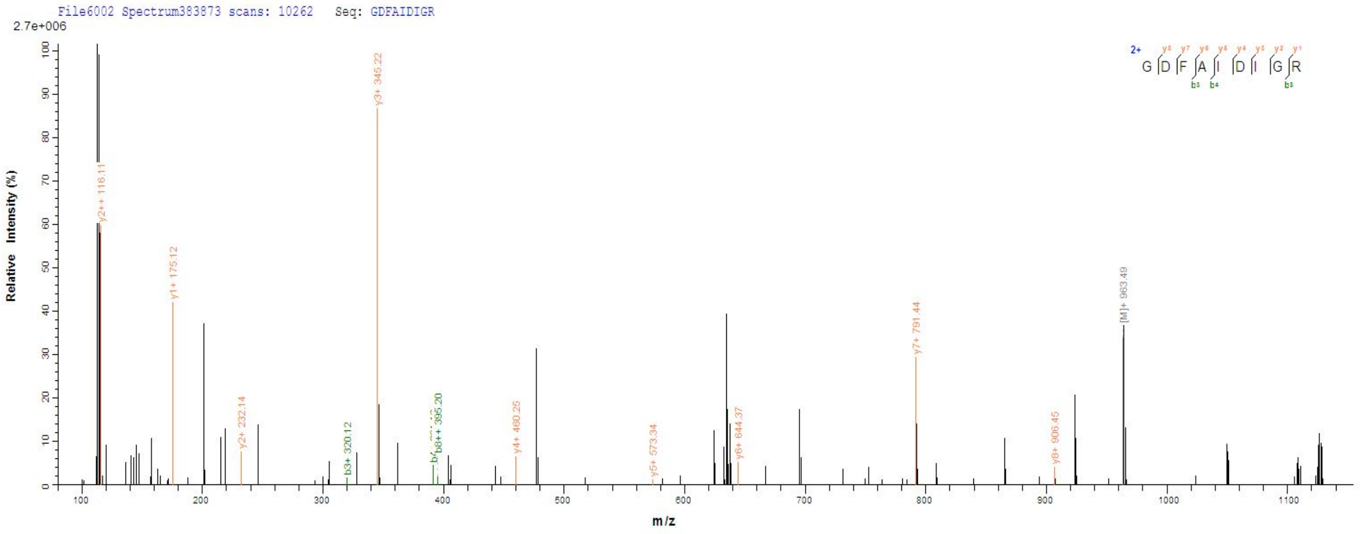

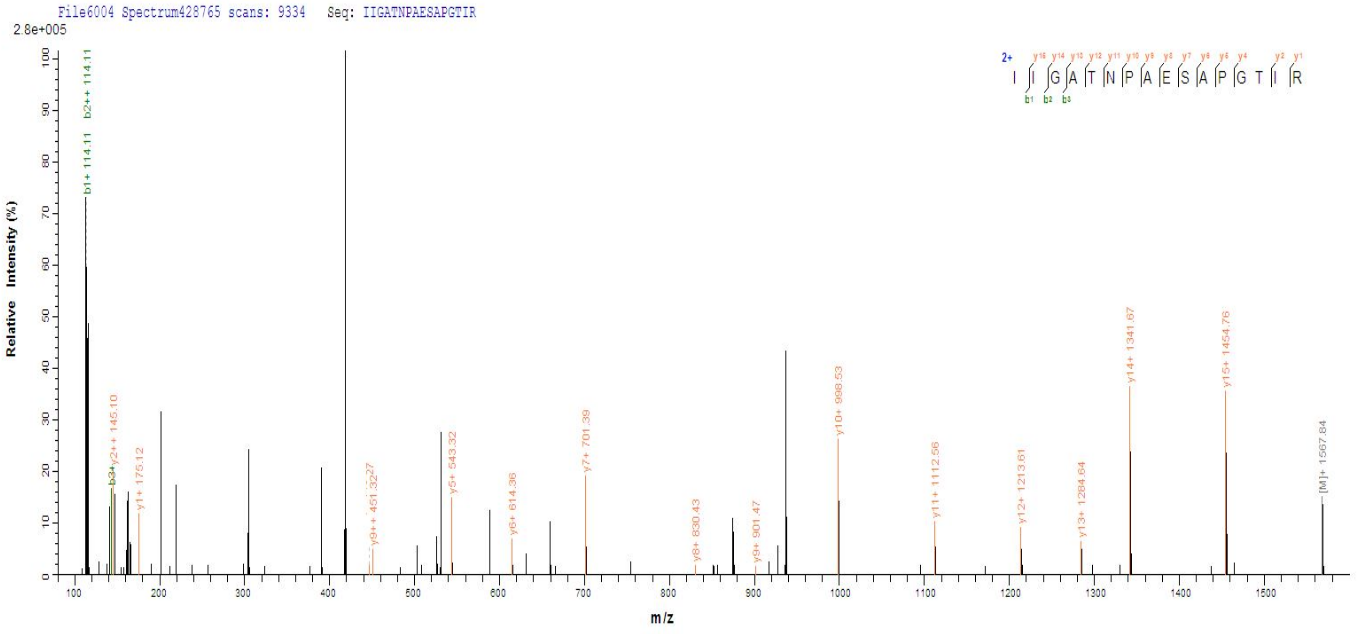

MEQTFIMIKPDGVQRGLVGEIIGRFEKKGFYLKGLKLITVDQSFAEKHYADLSAKPFFNGLVEYIISGPVVAMIWEGKNV
VTTGRKIIGATNPAESAPGTIRGDFAIDIGRNVIHGSDSVESAKKEIALWFPESPVNWQSSVHPWIYE

78 Garb_12824 gi|74229677 cytoplasmic Cu/ZnSOD

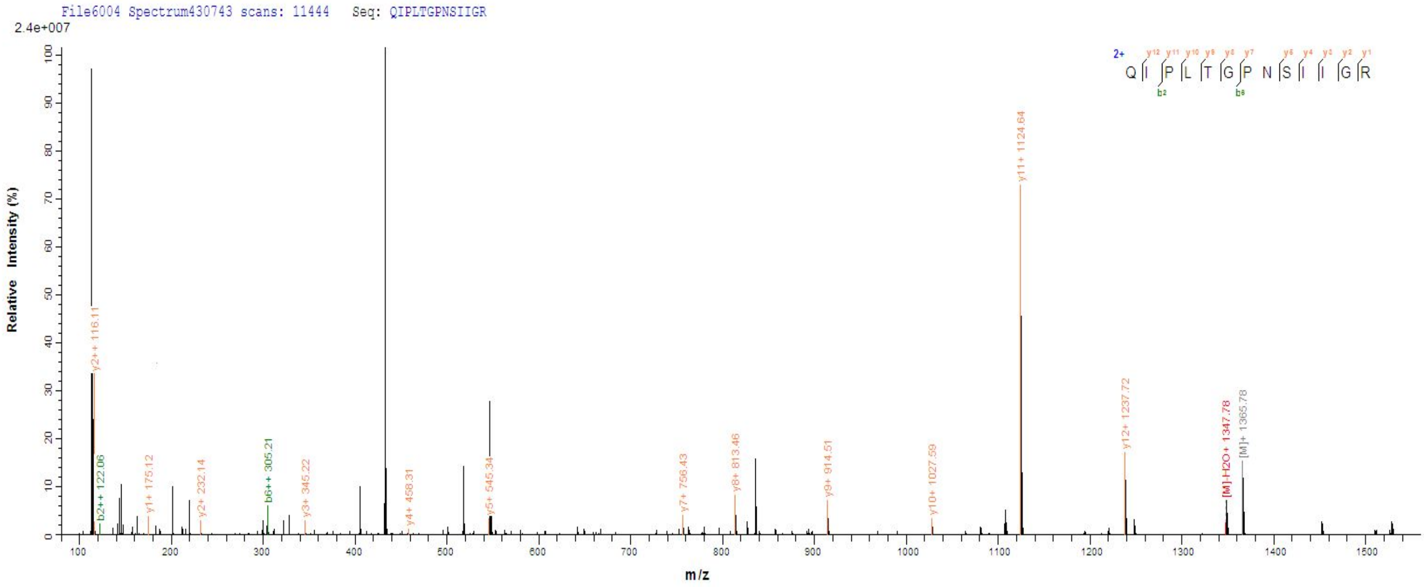

MVKAVAVLGSNEGVSGTVFFSQEGDGPTTVTGNLSGLKPGLHGFHVHALGDTTNGCMSTGPHFNPAGKEHGAPEDEN
RHAGDLGNVTVGDDGCASFSITDKQIPLTGPNSIIGRAVVVHADPDDLGKGGHELSKSTGNAGGRVACGKLSISITCSIIL

79 Garb_40884 gi|357470271 Peroxidase

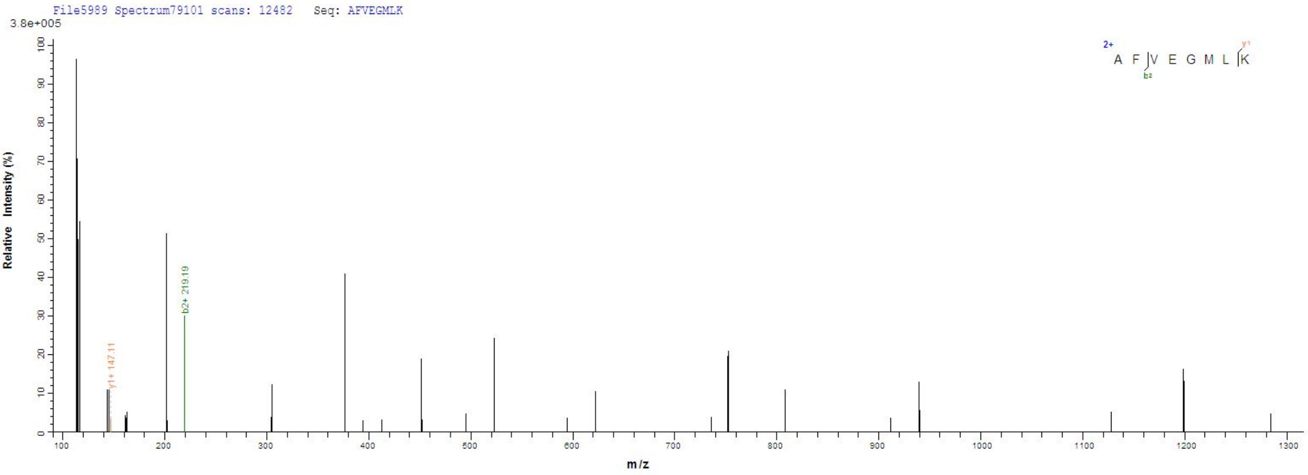

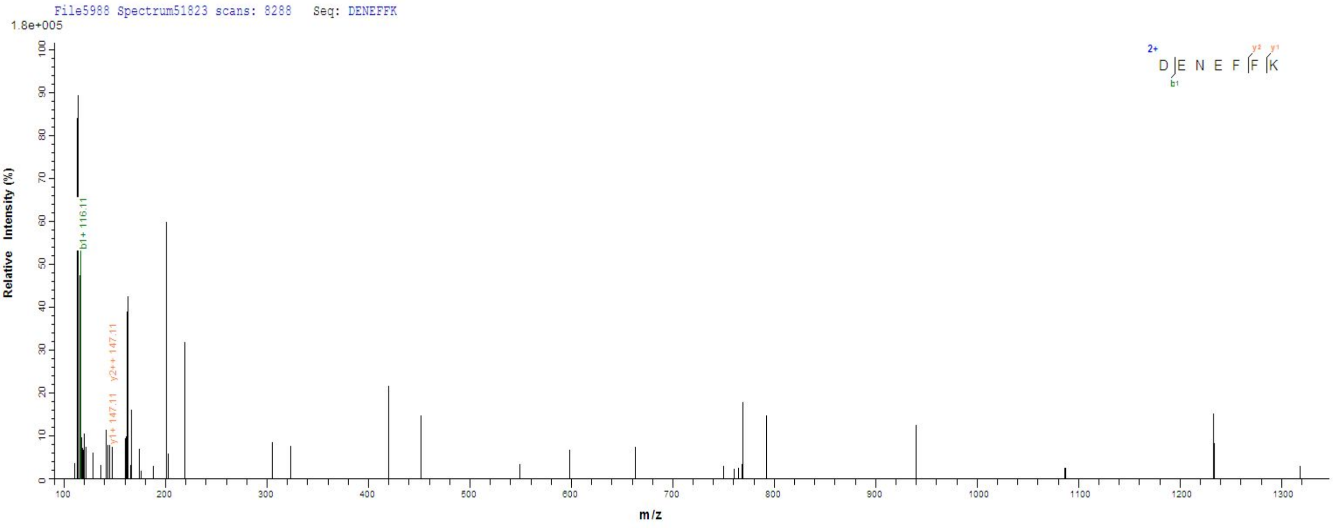

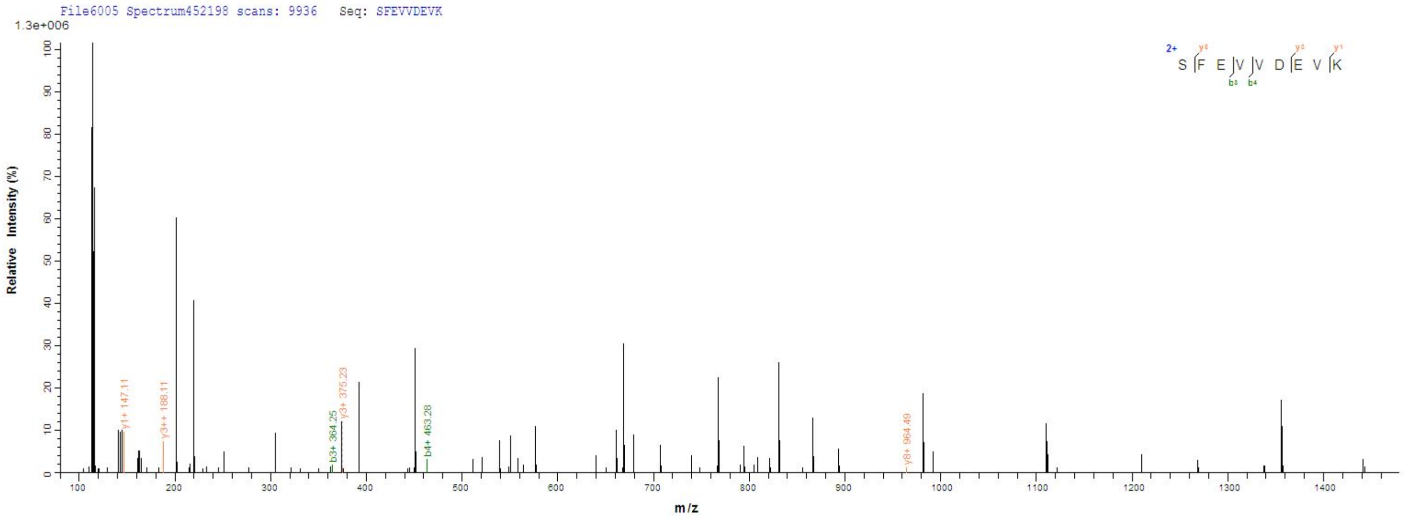

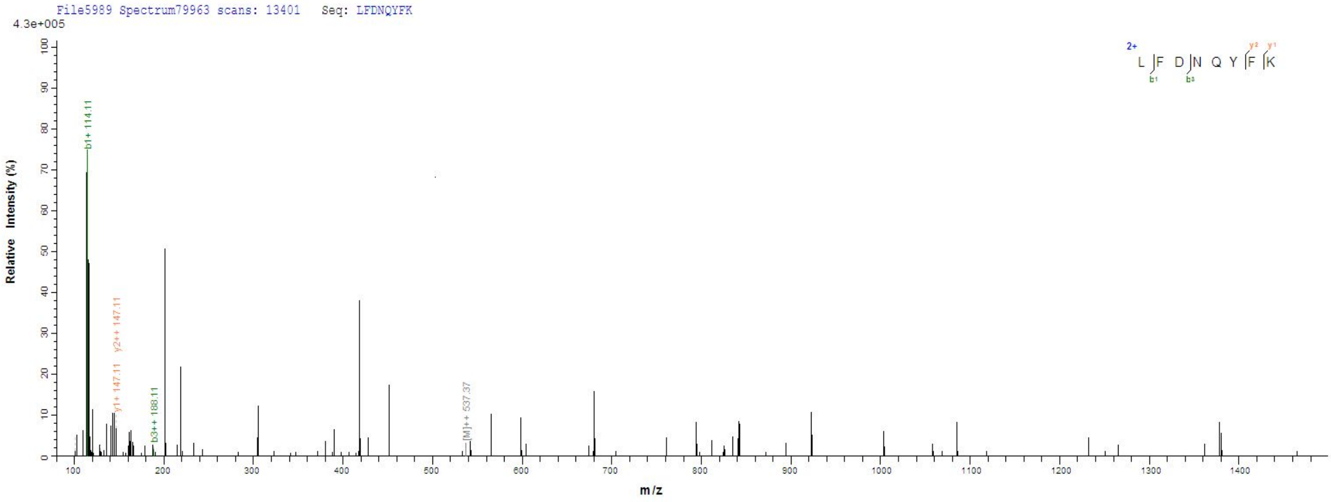

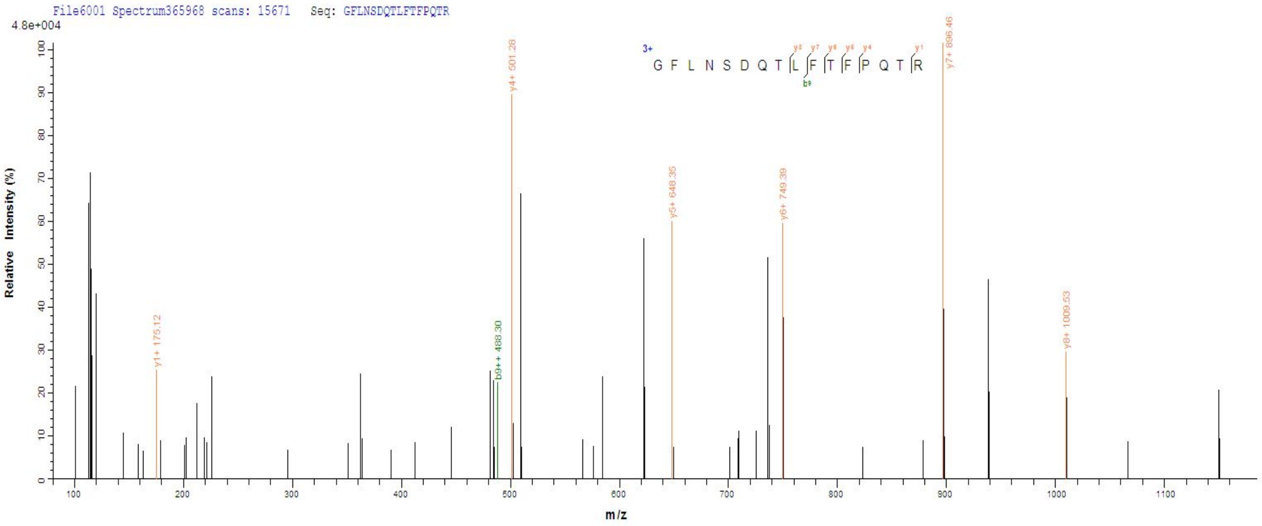

MSLYPSVLFVLLVTMAAAETLRPGFYAQTCPEAEAIVRYEMMKAMIREPRSVASVMRFQFHDCFVNGCDASLLLDDTP
NMLGEKLALSNINSLRSFEVVDEVKEALEKACPGIVSCADIIIMAARDAVALAGGPDWVVRLGRKDSLTASQEDSDNI
MPSPRANATSLIDLFTKFNLSVKDLVALSGSHSIGEARCFSIMFRLYNQSGSGKPDPAIEPGFREKLDKLCPLGGDENVT
GDLDATPKLFDNQYFKDLVAGRGFLNSDQTLFTFPQTREYVKLFSKDENEFFKAFVEGMLKMGELQSGKGGEIRTNCR
VVNSQALDV

80 Garb_38204 gi|115345276 peroxidase

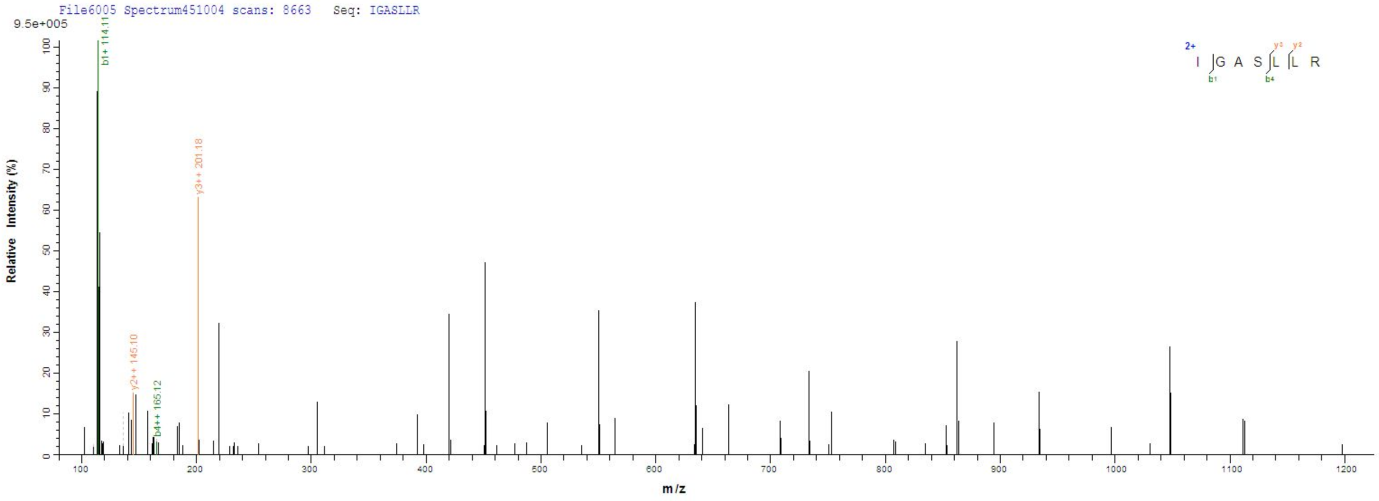

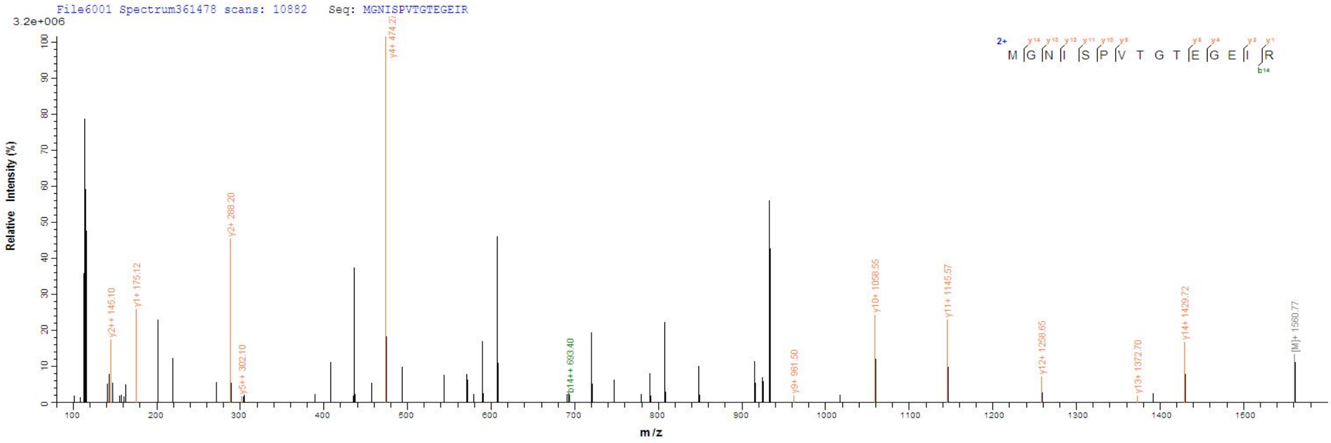

MPSLRCIIATLFFALLLPPGSFPATTLSPSFYDETCPAVFSIIRGIIEQALLSDPRIGASLLRLHFHDCFVNGCDASILLDNSAT
IESEKEAAPNNNSARGFEVVDAMKIALEFECPGIVSCADILAIAAQEAVNLAGGPSWLVLLGRRDSTAANRTLANLAIP
APFDTLNVLKSKFAAVGLNTSTDLVALSGAHTFGRAQCTILIERLYNFNGTGKADPTLNTTYLETLRKVCPEGGNGSVL
VNLDPTTPNTFDSNYYTNLQAQEGLLQSDQELFSTSGADTIEIVERFSSNQIAFFESFVVSMLKMGNISPVTGTEGEIRLS
CRRVNMDYTSSNKWSSS

81 Garb_21995 gi|73913500 peroxidase

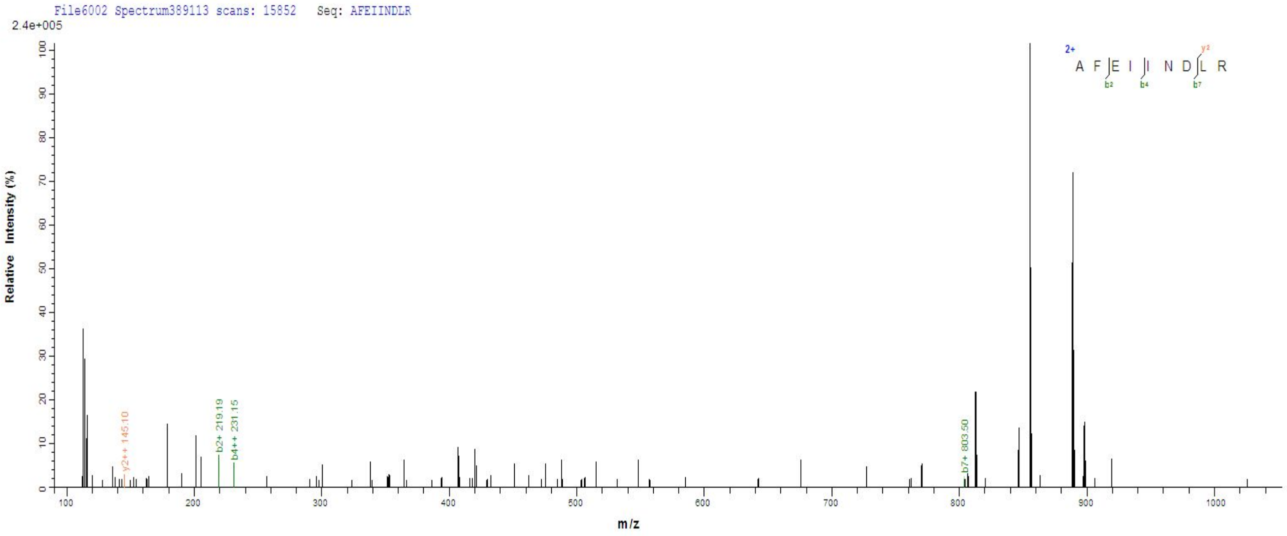

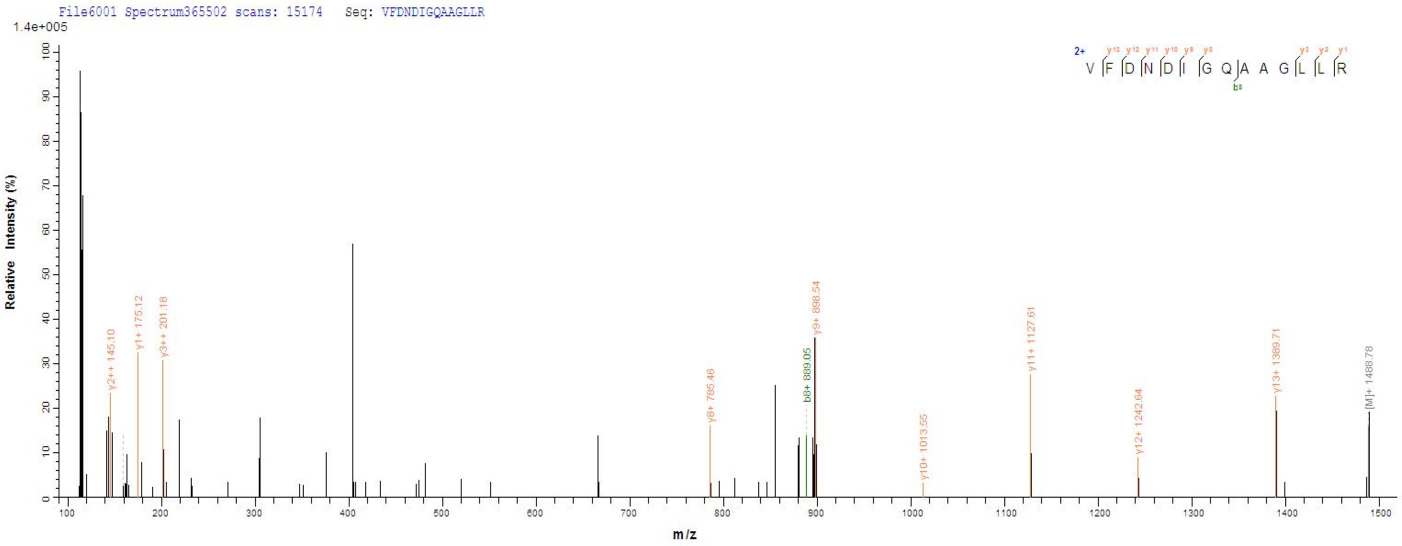

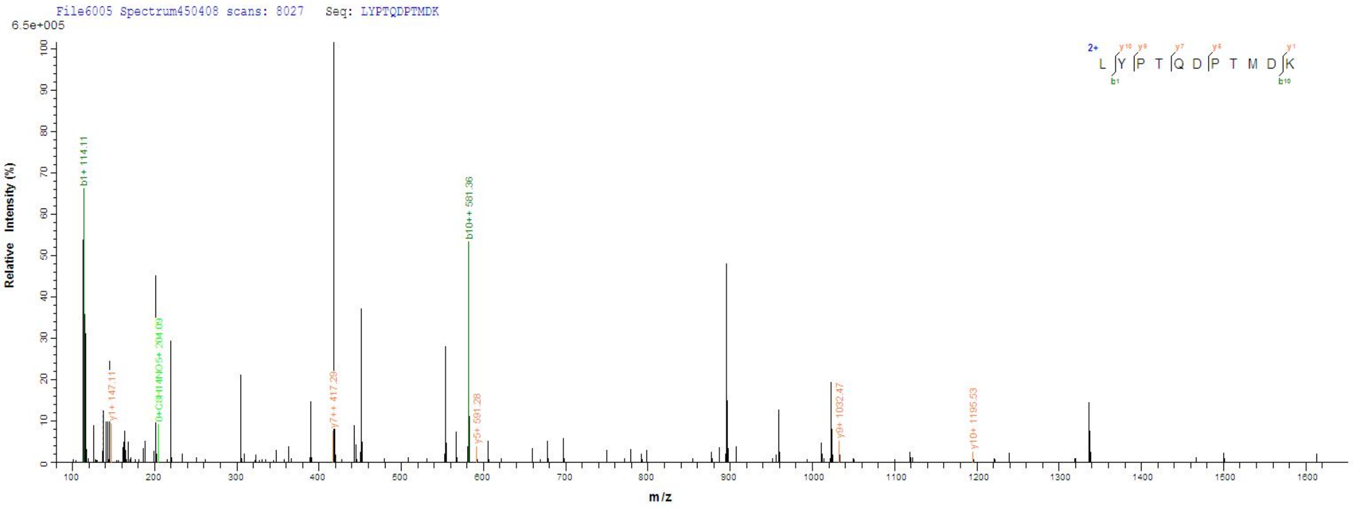

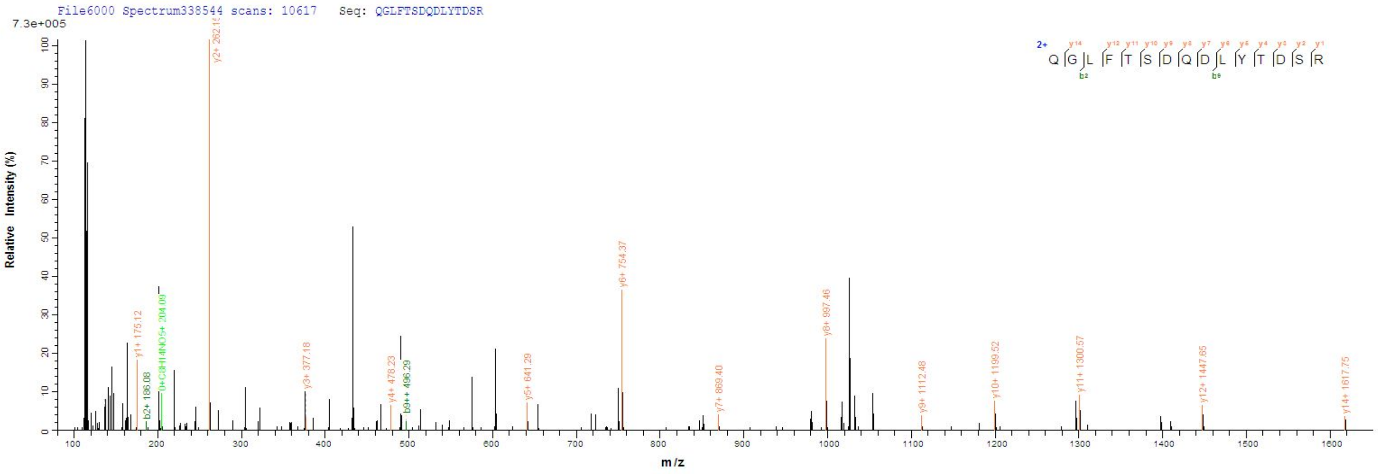

MAKVTNSFFALLLISSLLIAPYLSISEAADNSVPIVNGLSLTFYKTSCPKLESIIRTQLKKVFDNDIGQAAGLLRLHFHDCF
VQGCDGSVLLDGSASGPGEQQAPPNLSLRAKAFEIINDLRNRVEKACGRVVSCSDIVALAARDSVYLSGGPDYGIPLGR
RDGLTFATVNATLQNLPPPFANATTILSMLATKNFDPTDVVALSGGHTIGISRCTSFTTRLYPTQDPTMDKTFANNLKVIC
PTLNSTNTTVMDIRSPNKFDNKYYVDLMNRQGLFTSDQDLYTDSRGQLSVLTGNNGEIRANCSVRNADNKSYLASVA
EELPVEEAWSDL

82 cotton_GLEAN_10015273 gi|255551599 Peroxidase 26 precursor, putative

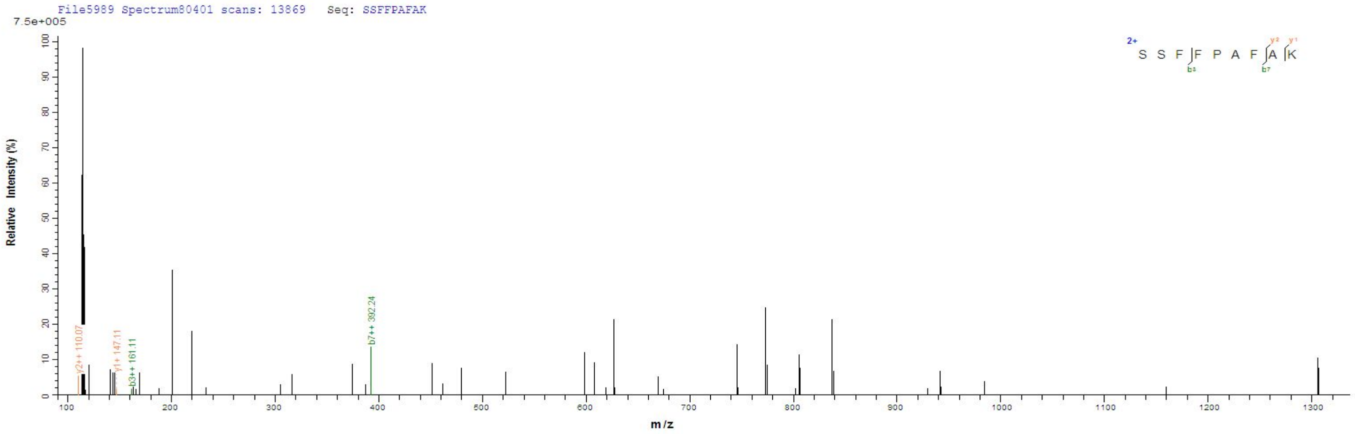

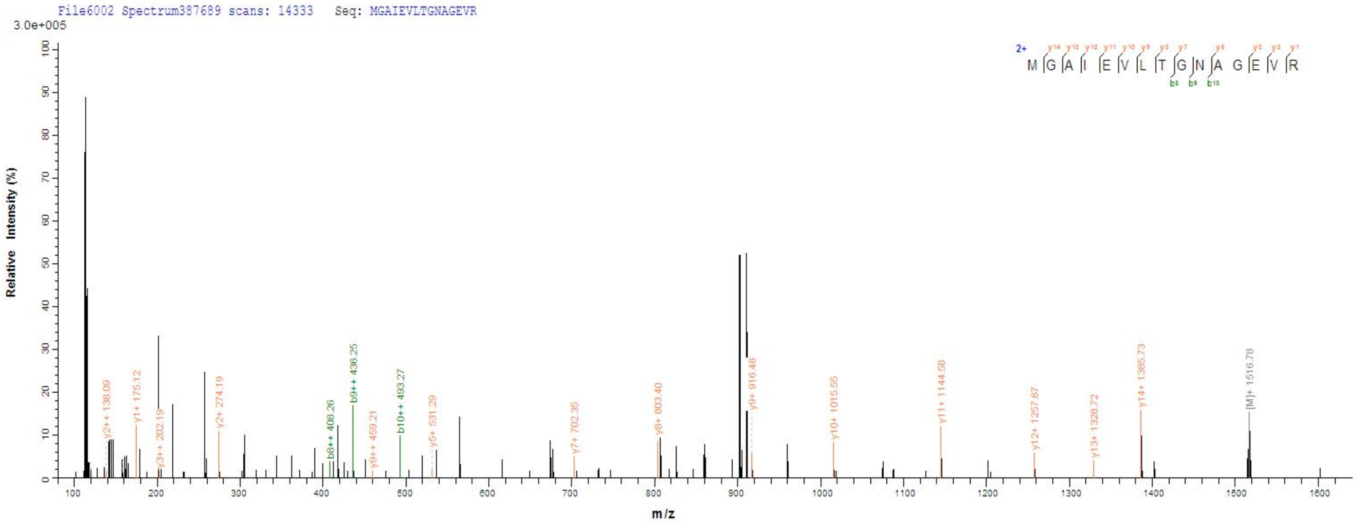

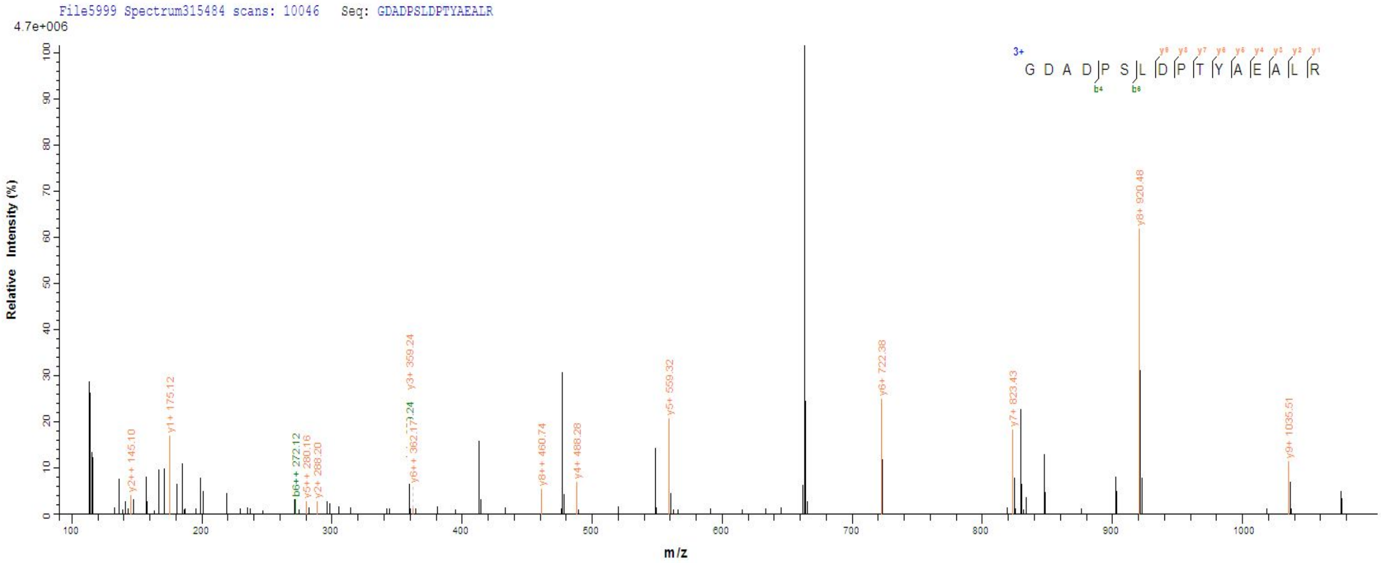

MRAGYCIFILLVSLTLFGIVDVCNAGKLSYKFYKRTCPRAEQIVKEIIQNRTQSSPSLGARLIRMQFHDCFVRGCDASVL
LDTVNNSTAEKEAIPNSSLSGFDVIDDVKTAIERVCPKVVSCADILALAARDAVSAPFSKPLWDVQLGRRDGMVSLATE
TNGNLPSPFANFTSLIQLFNRKGLDVNDLVVLSGAHTIGVAHCATFSSRLYNFTGKGDADPSLDPTYAEALRKQCPNPAS
PTITVEMDPTSSLSFDNHYYDILLQKKGLFGSDAALLTNRNSRKIVTRLQRSRSSFFPAFAKSVKKMGAIEVLTGNAGEV
RQNCRVVNP

83 Garb_10901 gi|32351452 class III peroxidase

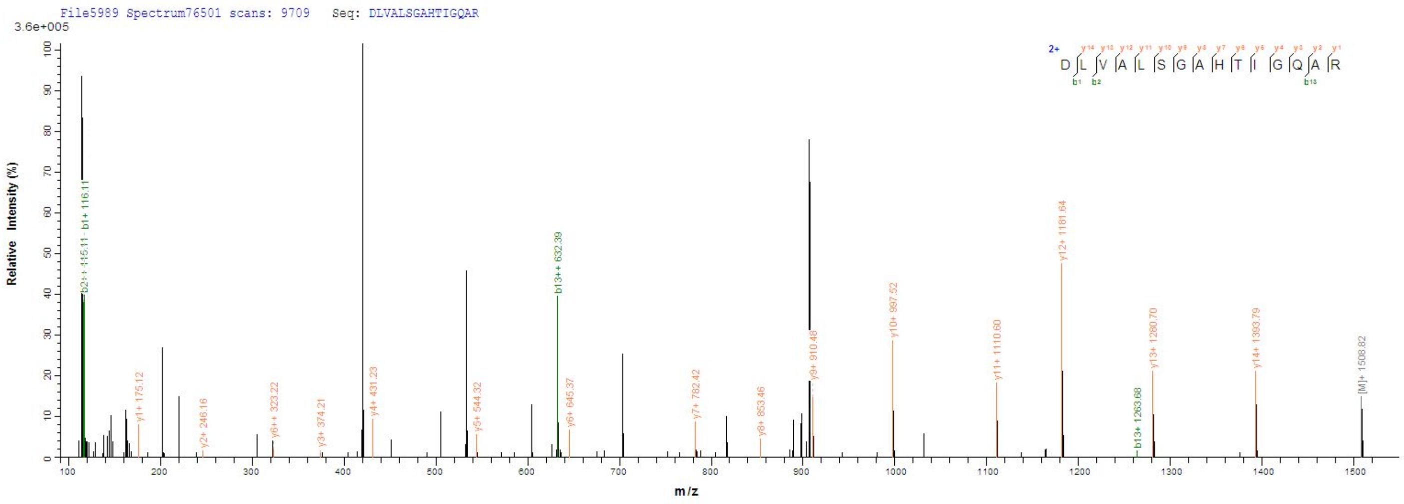

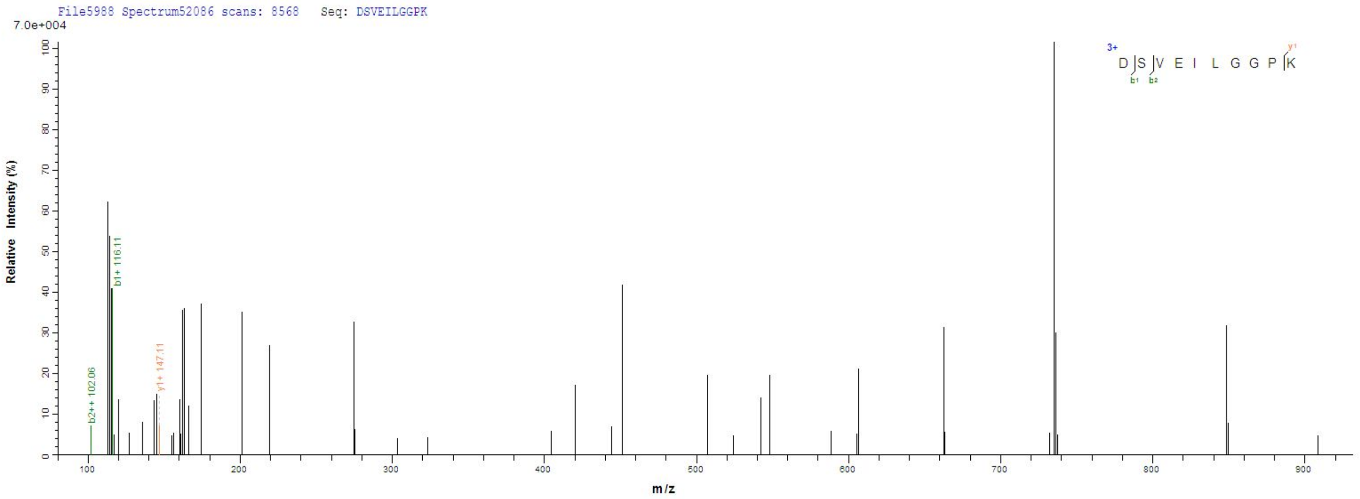

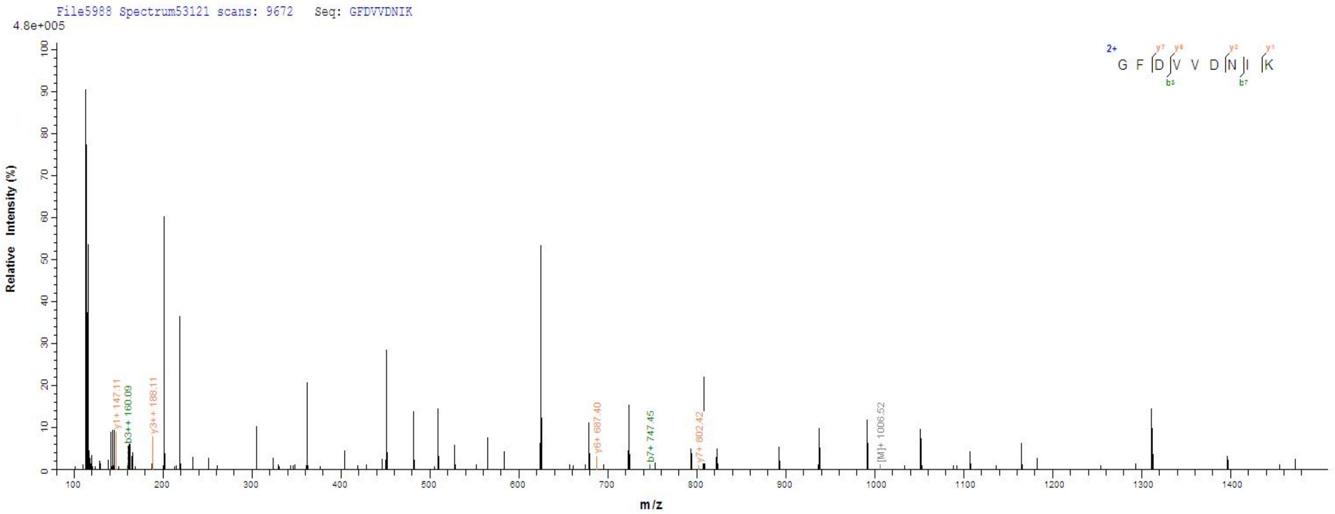

MGSASSFSKFCLTLLLLVVVLGSTNAQLSTNFYSKSCPKLLSTVKSTVTSAINKEARMGASLLRLFFHDCFVNGCDGSV
LLDDTSSFTGEKNAIPNRNSARGFDVVDNIKSAVENVCPGVVSCADILAITARDSVEILGGPKWAVKLGRRDARSASQS
AANNGIPAPTSNLNQLTSRFNALGLSTRDLVALSGAHTIGQARCTSFRARIYNESNIDASFAQTRQRNCPRTTGSGDNNL
APLDIQTPTSFDNNYFKNLVSRRGLLHSDQQLFNGGSTDSIVRGYGNSPSSFNSDFVSAMIKMGDISPLTGSRGEIRKNC

84 Garb_06127 gi|255581003 Peroxidase 2 precursor, putative

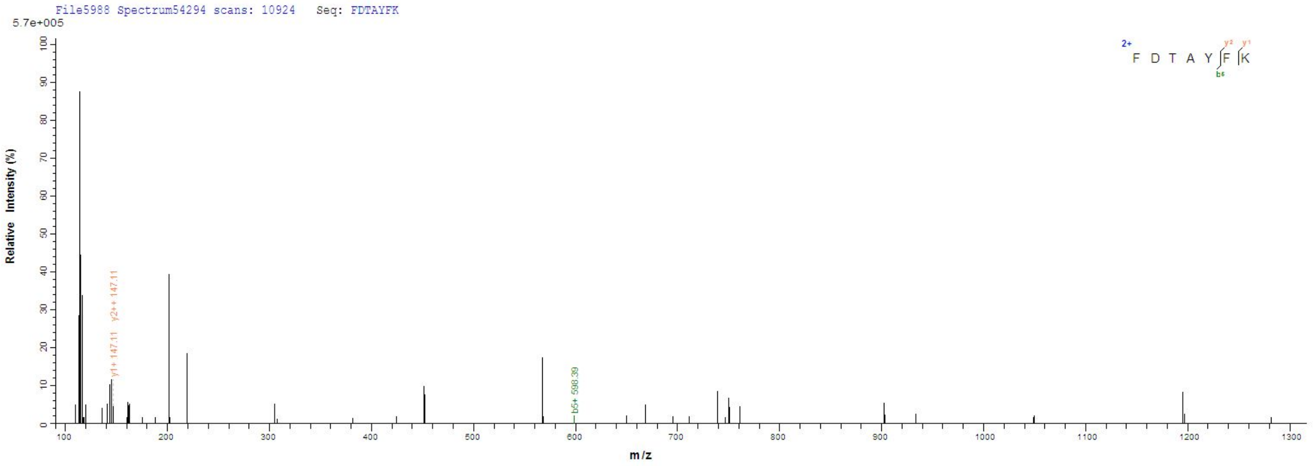

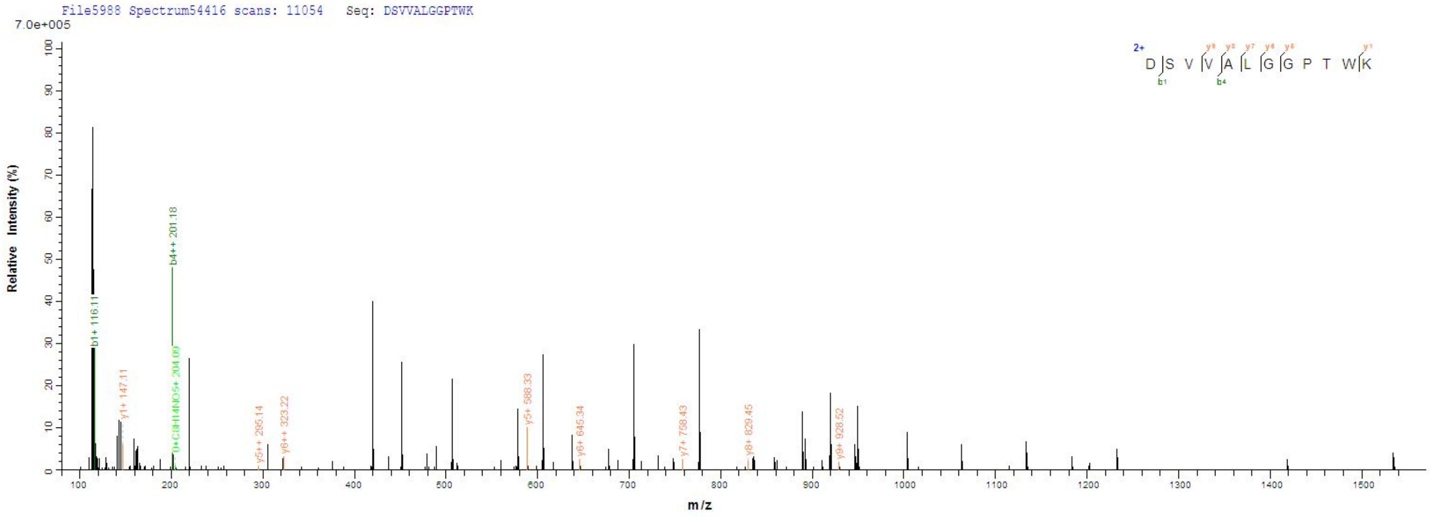

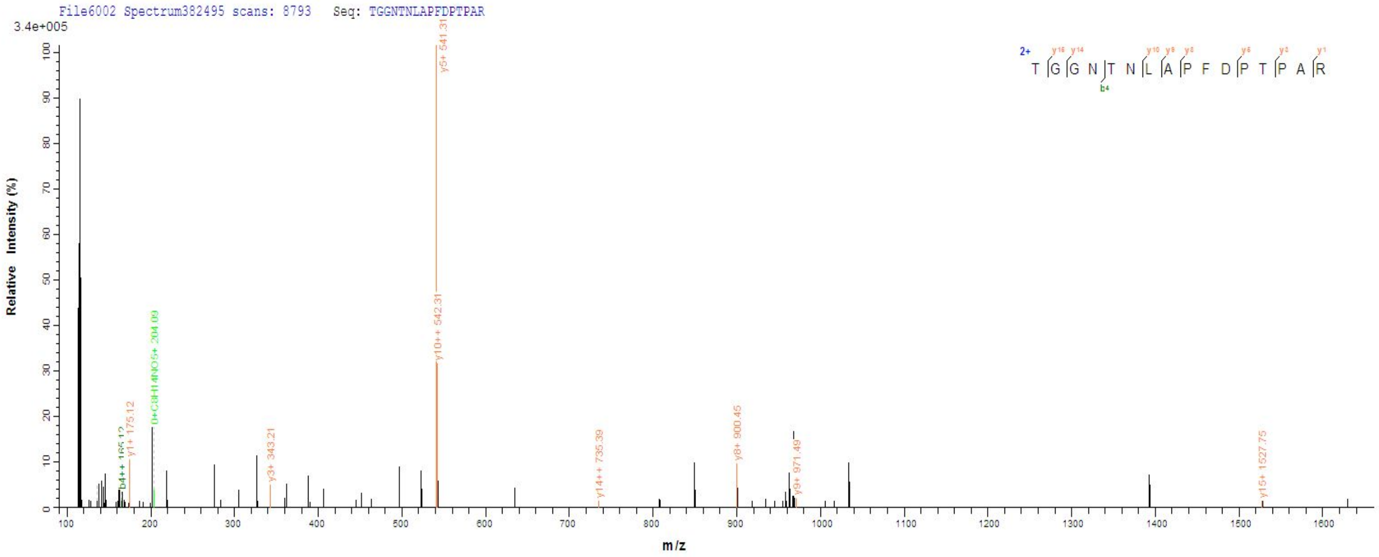

MGASLLRLHFHDCFVNGCDGSLLLDSTSSFETEKNARGNLNSVRGFEVVDQIKAEVDRVCGRPVVSCADILAVAARDSVVALGGPTWKVRLGRRDSTTASRTLADSVLPSASMDLPALINNFKNQGLSKRDLVALSGGHTIGLSQCVIFRNRIYNATNIDPAFAKERRATCPRTGGNTNLAPFDPTPARFDTAYFKNLVKERGLLTSDQALFSGGSTDKLVETYSKNPNVFWVDFGKSMIKMGNIKPLTGKQGQIRANCRKVN

85 Garb_30095 gi|25453205 Peroxidase 12

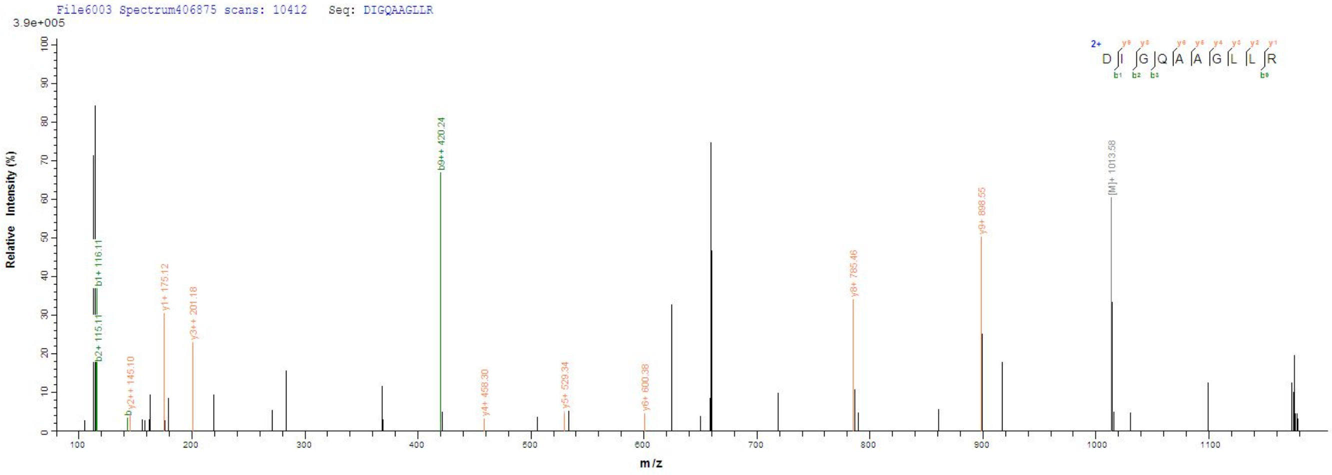

MSSLFLSSYFSVSKADNPAPIVSGLSWTFYKTSCPKVESIIRKQLQKVFKKDIGQAAGLLRLHFHDCFVQGCDASVLLDGSASGPSEQDAPPNLTLRGFEIINDLRARVHKECGRVVSCADIVALAARDSVYLSGGPDYDVPLGRRDGLSFATRNATLQNLPAPFANAAAILSSLATKNFDPTDVVALSGGHTIGISHCSSFTGRLYPTQDPTMDQTFAKNLKHVCPTANSSNTTVLDIRTPNKFDNKYYVDLMNRQGLFTSDQDLYTNSRTRGIVTSFAVNQTLFFEKFVAAMVKMSQLSVLTGKAGEIRANCSVRNANNNSLLASVVEEEARSEF

86 cotton_GLEAN_10023951 gi|225447324 PREDICTED: peroxidase 27

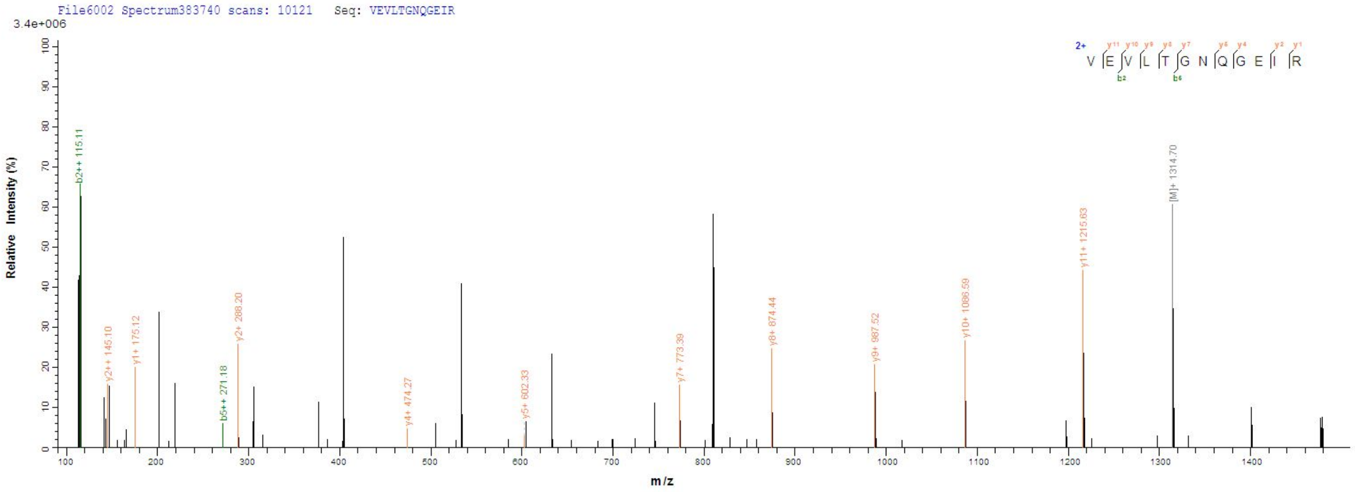

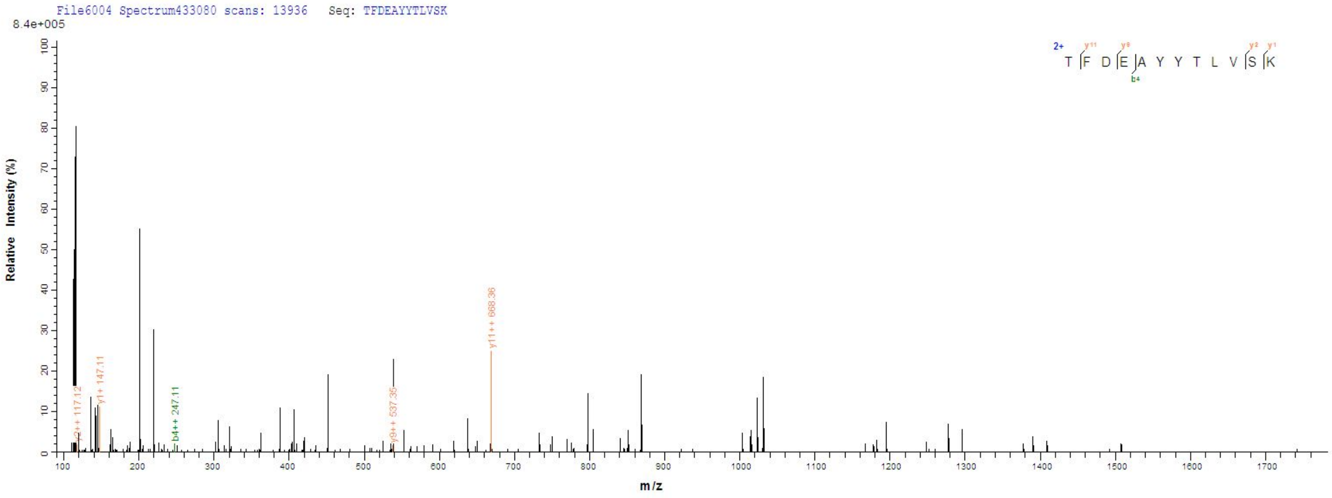

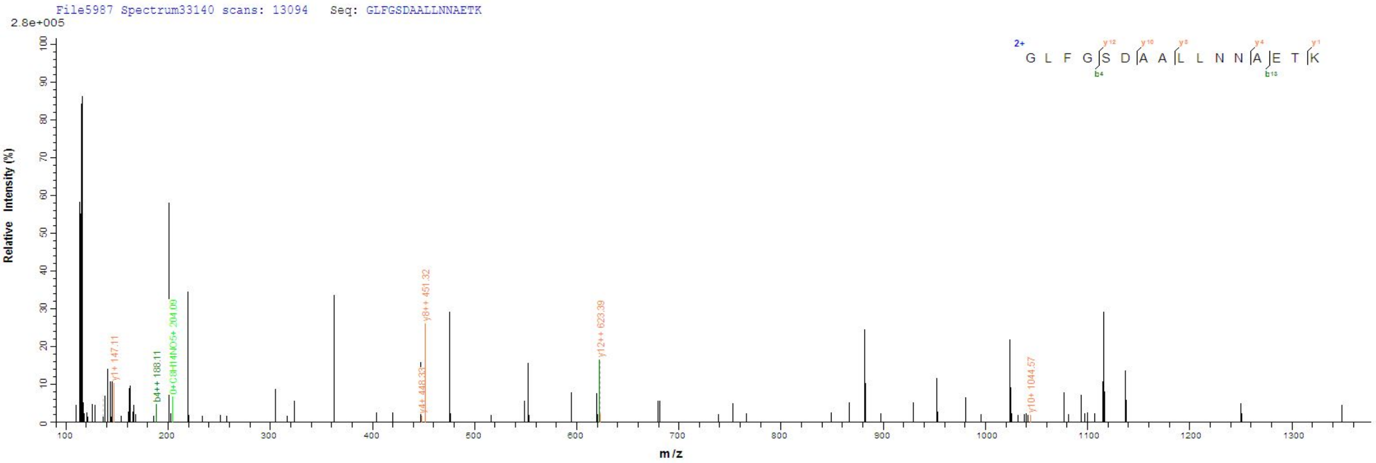

MEIGQRVLLCLHIQLIIVVMVLNHSNAQGLKLGFYSETCPNAESIIRKTTYGFISRAPTLAAPLLRLHFHDCFVRGCDGS
VLLNSTKNQAEKDAIPNLSLRGYHVIDAVKSAVEQACPGVVSCADILALAARDSVSMINGPSWKVPLGRRDGRISKLS
EALANLPSPFFNVTQLKQNFASKGLNMKDLAVLSGGHTIGTSHCVAFGLRLYNFSGKGDADPSMDPTYVTQLKQKCK
PGDITSLVEMDPGSFKTFDEAYYTLVSKRRGLFGSDAALLNNAETKAYVLQASRHGSTFAKDFAVSMEKMGKVEVLTG
NQGEIRKHCAMVN

87 Garb_23542 gi|220967704 monodehydroascorbate reductase

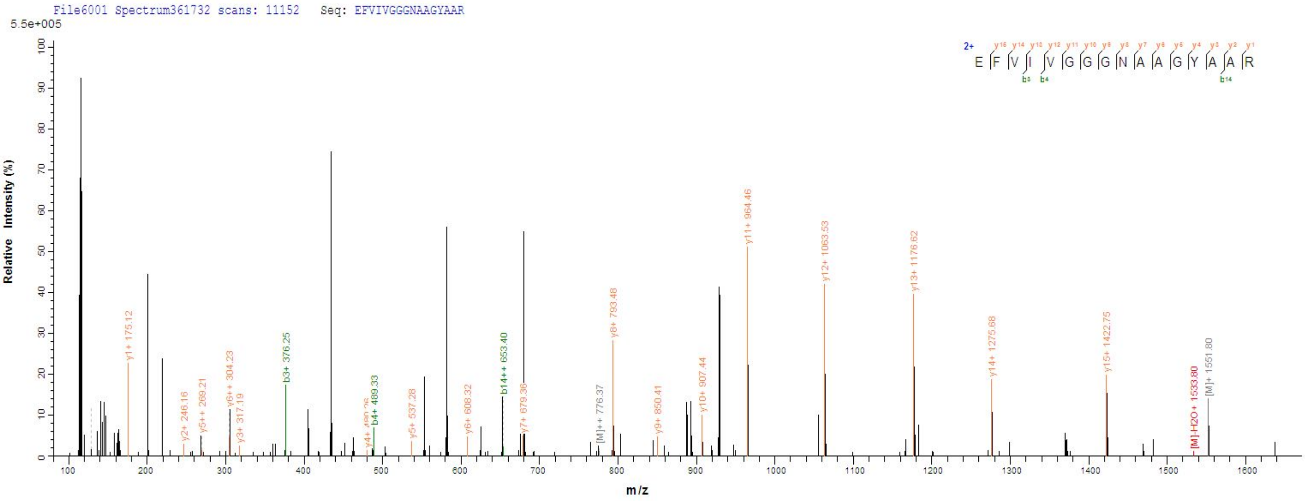

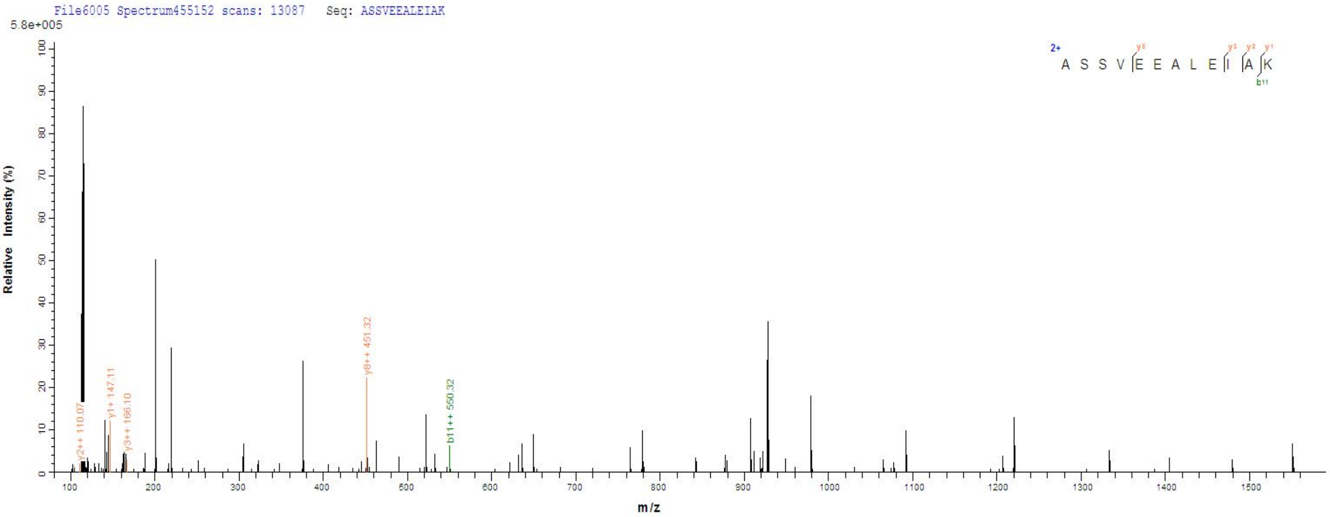

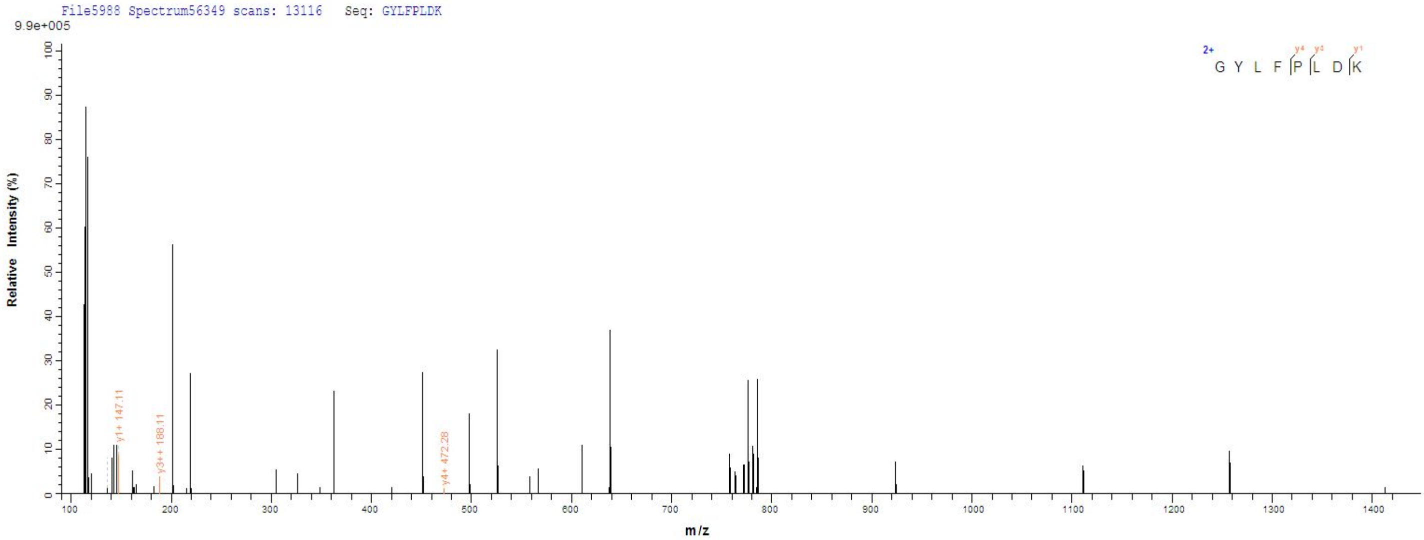

MTGTDQSSFLGRISIRRNQVNAMEGYHEQEIEDLELFQKQLSDRFAELLSAPDDAPTEAFLSISWIRKLVDVFLCCETEC
KAILLMERDAAQISKPPLDRLIPELLERVVKAMDICNAVTNGLELVRHCQKLAEIAVSALEQKPFGEGQARRAKKALVS
LMSAMHVDDKESSVQKTAERSWSFGRRSGNKDRHHGHYRSLSWQVAKHWSASKQIHAMTMNLAAPRGPEASCLPA
PVYILSLIIVFVMWVLIAAIPCQERSGLTTHLPINKHVNWTHSLAGIHDKIGEEWKKKEKKGMAGLLYEMQKMEKLGQ
SLIEFTDSYQFPGEKEKLDEVAAQVAELAEVCRRMDEGLVPLQMLIREAFHRPETMDRICVCFSTLYSAARKAMASLSN
SLQLKHGLTSWCPGSSALTRRLPSSSIRFRSFVVAASSFSNDNREFVIVGGGNAAGYAARTFVEHGMADGKLCIVSKEA
YAPYERPALTKGYLFPLDKKPARLPGFHTCVGSGGERQTPEWYKEKGIEMIYEDPVTGIDTEKQTLTTNSGKLLKYGSL
IIATGCTASRFPDKIGGNFPGVHYIRDVADADSLISSLEKAQKVVIVGGGYIGMEVAAAAVSWKLDTSIIFPENQLLQRLF
TPSLAQRYEELYKEYGVKFLKGASIKNLEAGPDGRVAAVKLGDGSTVEADMVVIGIGAKPAVSPFEVVGLNNTVGGIQ
VDGLFRTSVPGIFAVGDVAAFPLKMYDRVARVEHVDHARRSAQHCVKSLLSAQTHTYDYLPYFYSRVFEYEGSPRKV
WWQFFGDNVGETVEIGNFDPKIATFWIDSGKLKGVLLESGNAEEFKLLPELARNQPSIDKAKLEKASSVEEALEIAKAS
LQIV

88 cotton_GLEAN_10012655 gi|195973264 glutathione S-transferase

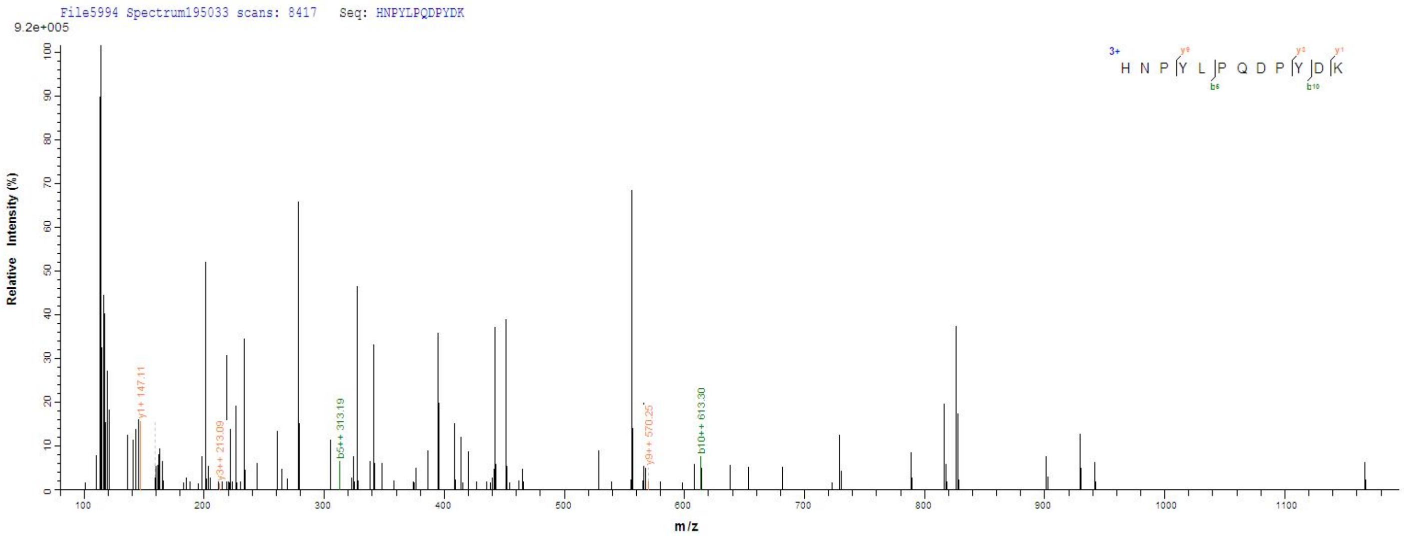

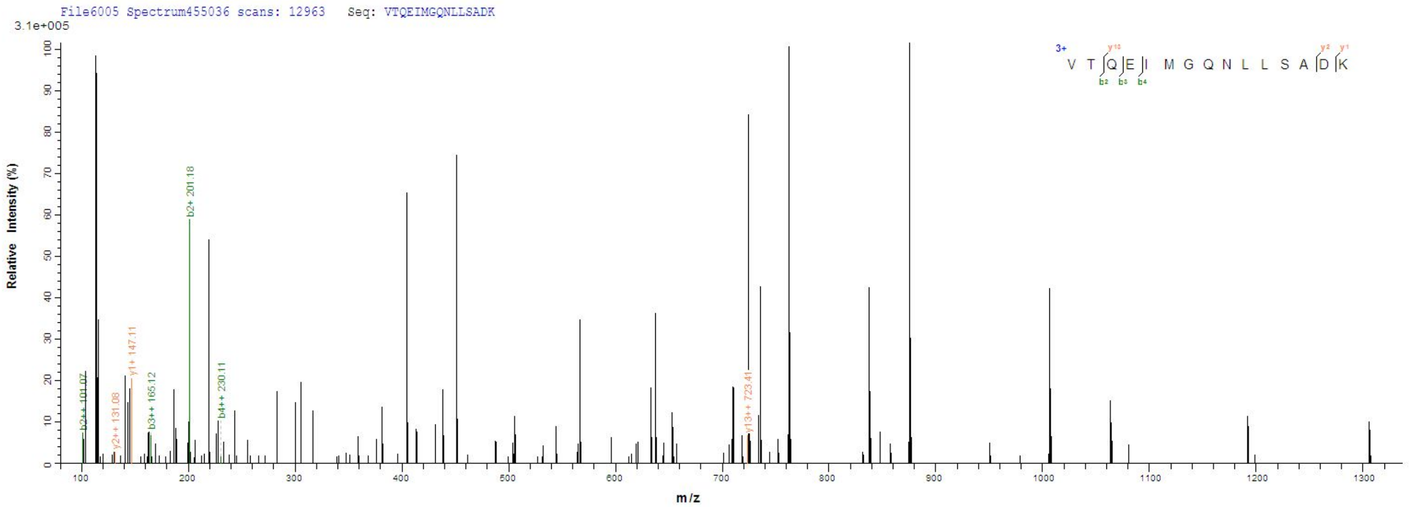

MGEEVKVFGYWASPYSYRAELALKLKGVSYEYINEDIFGNKSDLLLKYNPVHKKVPVLLHNGKPIVESLVILEYIEET
WKHNPYLPQDPYDKATARFWIKFIDEKCFPTLWLAAWSAENEREKVTNEACEYLKTLESALNGKKFFGGETIGMVDIV
ASSVGYFIRVTQEIMGQNLLSADKFPQLFQWSEDFANCSIVKESLPPRDKLLPFVKGLIAKYQQDNAKA

89 Garb_33533 gi|354620267 pCPR10-16

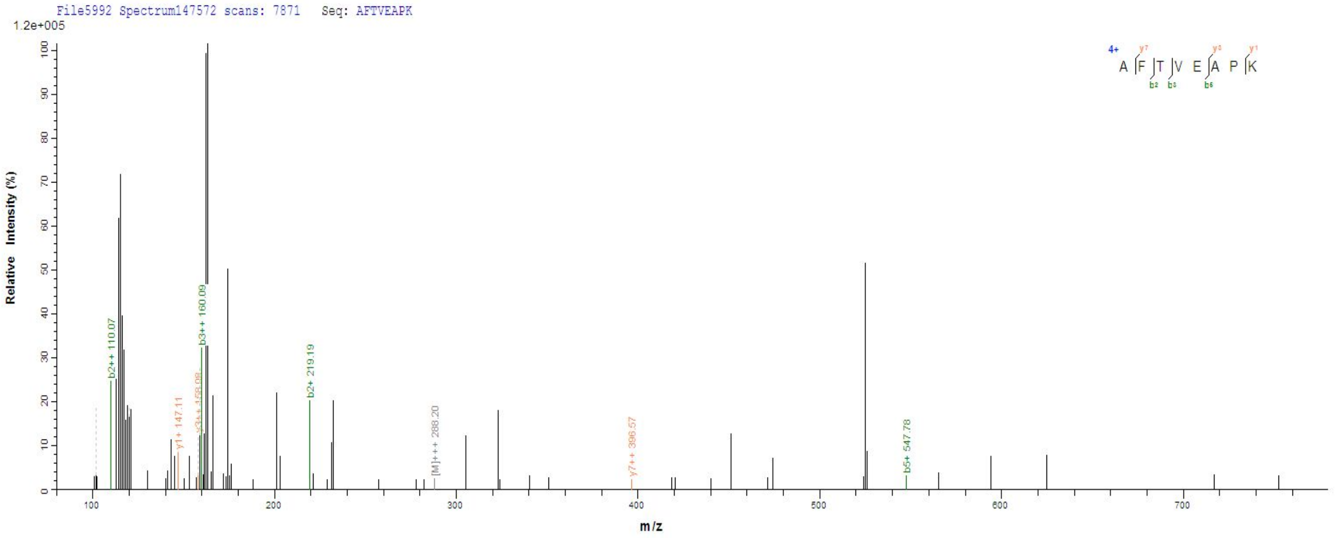

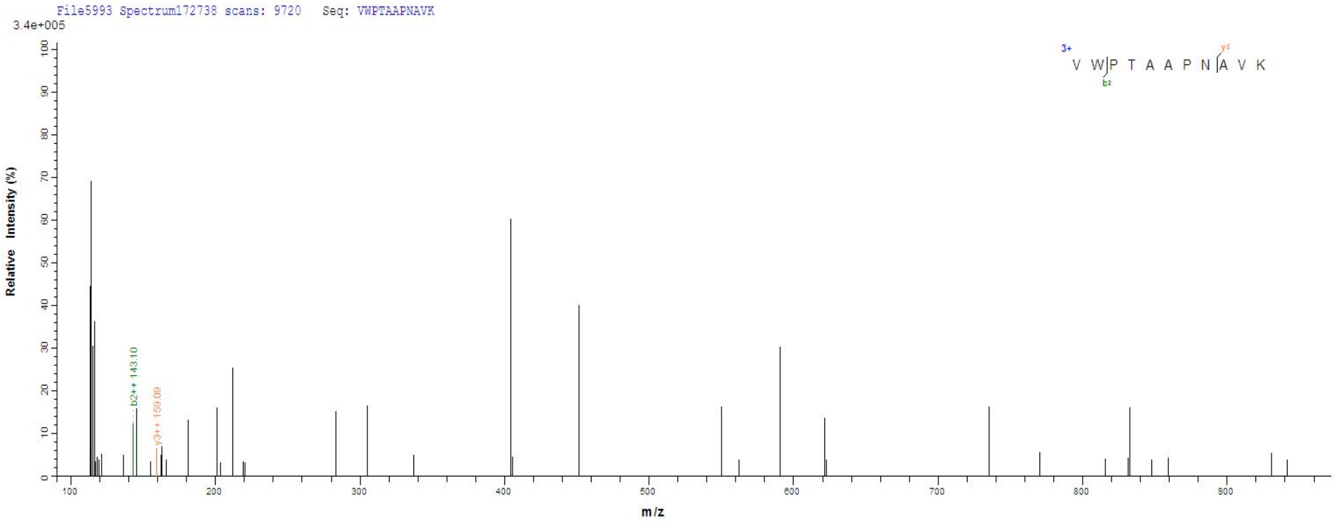

MGVFSYDYETTSPVAPARLFKAFTVEAPKVWPTAAPNAVKSIEVEANPSSGSIVKINFVEGFPFQYMKHQIGGHDENNF
SYSYSLIEGGPLGDKLEKISYENKFEATAGGGSICKSSMKFYTVGDNVITEDEIKARIKGSETVYKPIEAYLLANPEACN

90 Garb_26640 gi|15811629 ribonuclease-like PR-10

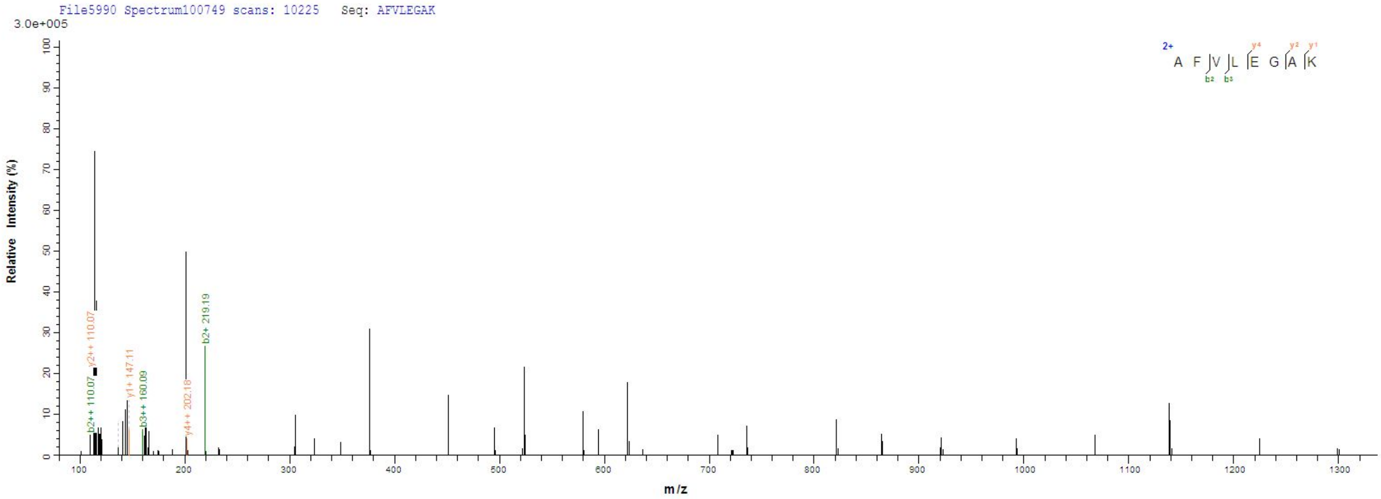

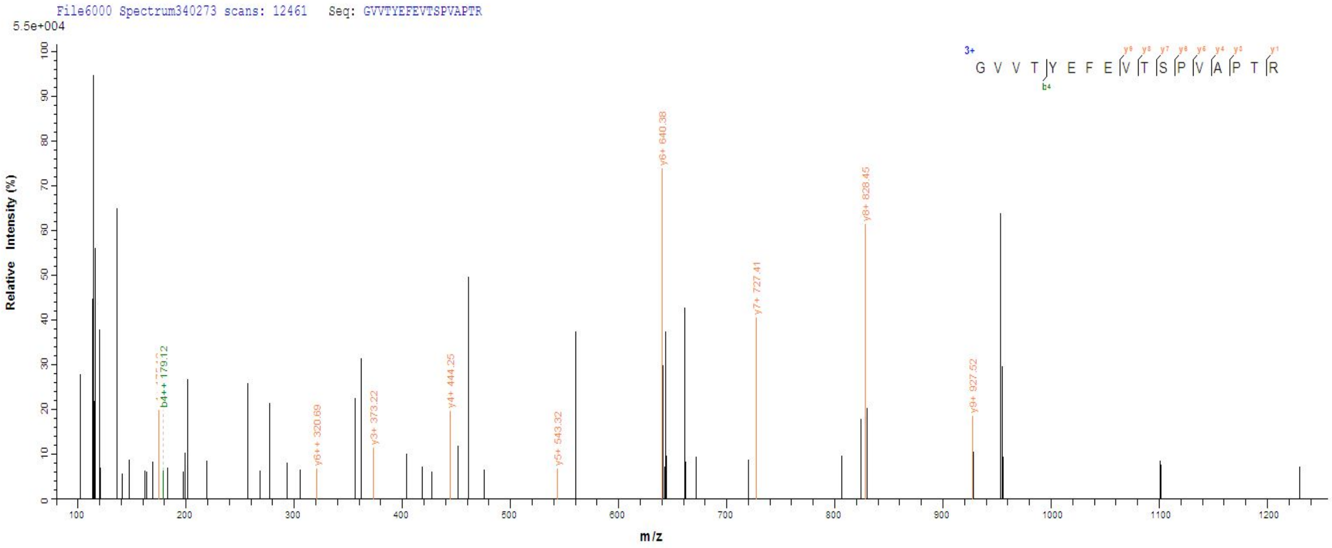

MGVVTYEFEVTSPVAPTRLFKAFVLEGAKVYPKAAPHAIHMIGGHDENNLSYSYSLIEGGPLGDKLEKISYENQFVAA
ASGGSVCKSSIKFYTVGDYVITEDEIKALIQRSEVVYKAIEAFLLANPDACN

91 Garb_14866 gi|33338347 osmotin-like pathogenesis-related protein

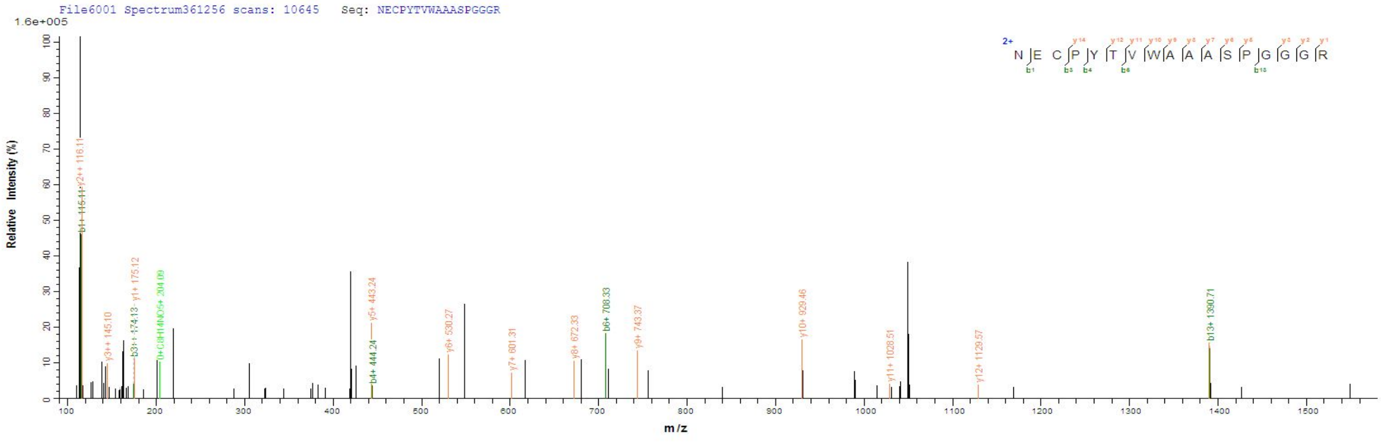

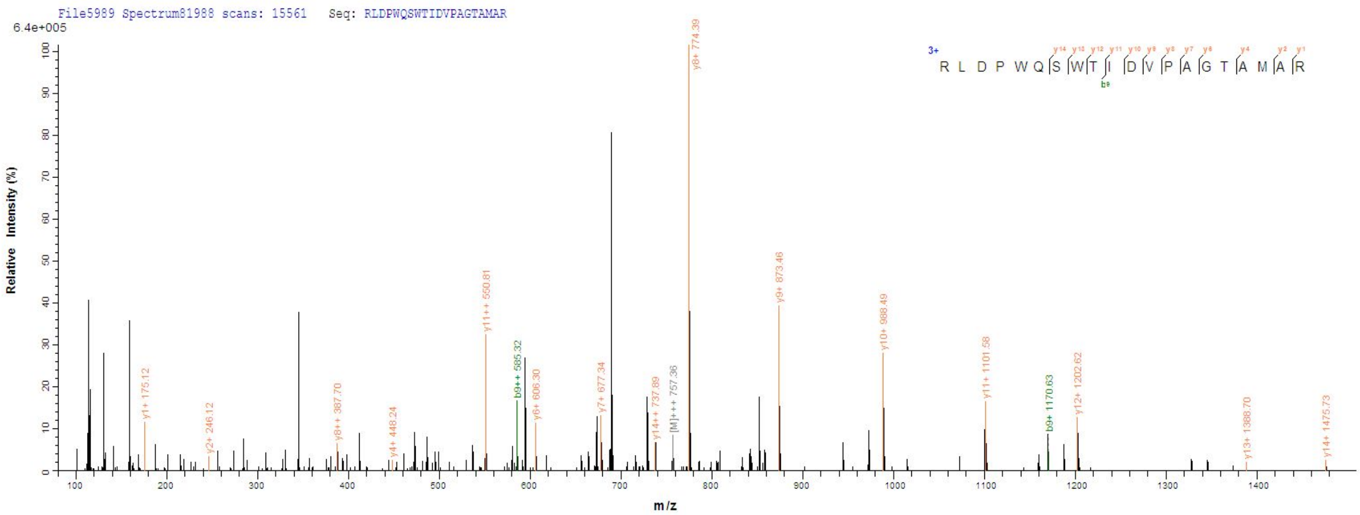

MSYLTISQISSILFFSVLFISAHAARFEIRNECPYTVWAAASPGGGRRLDPWQSWTIDVPAGTAMARIWGRTNCNFDASG
RGHCQTGDCGGLLQCQGWGVPPNTLAEYALNQFGNMDFYDISLVDGFNIPMVFGPTNGGCHNIRCTADINGQCPNEL
RAPGGCNNPCTVFKTNEYCCTQGYGTCGPTSYSRFFKDRCGDSYSYPQDDPSSTFTCPAGSNYRVVFCPRGSPRIEMV
GSKNQEK

92 Garb_05304 gi|255537367 Osmotin precursor, putative

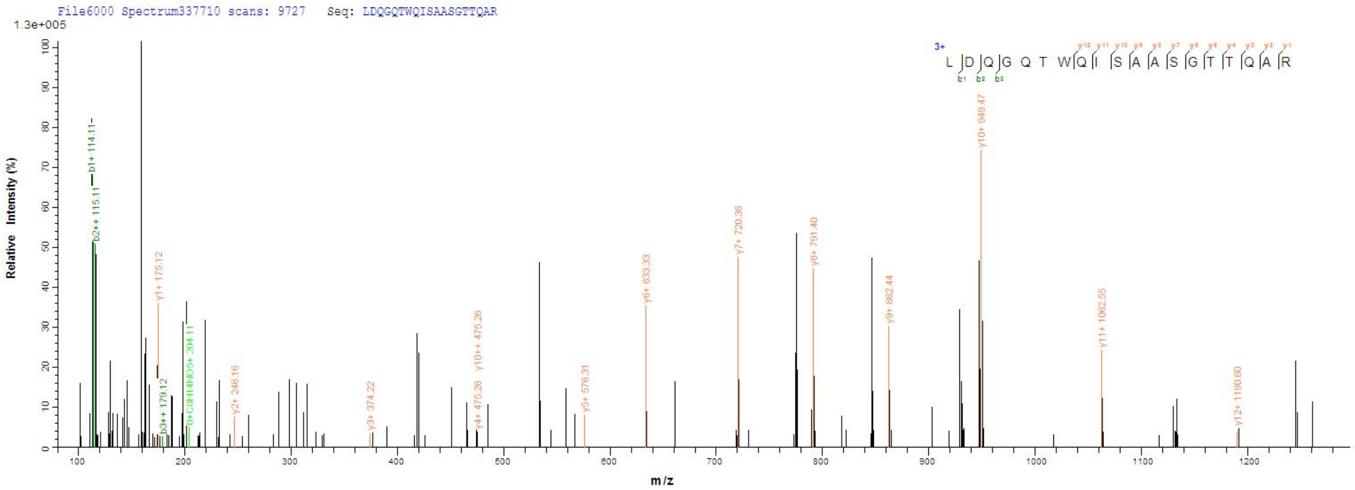

MNPFKTLPTLSFLFLTLLGLAHAATFDIRNNCPYTVWAAASPGGGKRLDQGQTWQISAASGTTQARIWARTKCNFDAS
GKGSCETGDCGGVLECKGYGKAPNTLAEYSIDQYEHQDFIDISNIDGFNVPMEFSSNSPGCTRVIKCTADIVGQCPNEL
KVPGGCNGPCPVFNTEEHCCNSGNCGPTNFSRFFKERCPDAYSYPKDDPTSLFTCATGTNYKVIFCP

93 Garb_26131 gi|383932370 nodulin-like protein

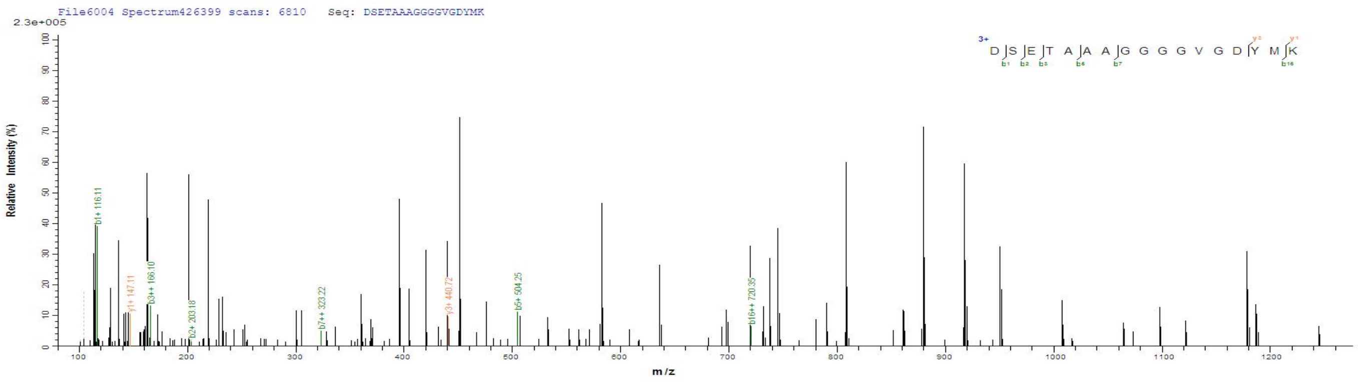

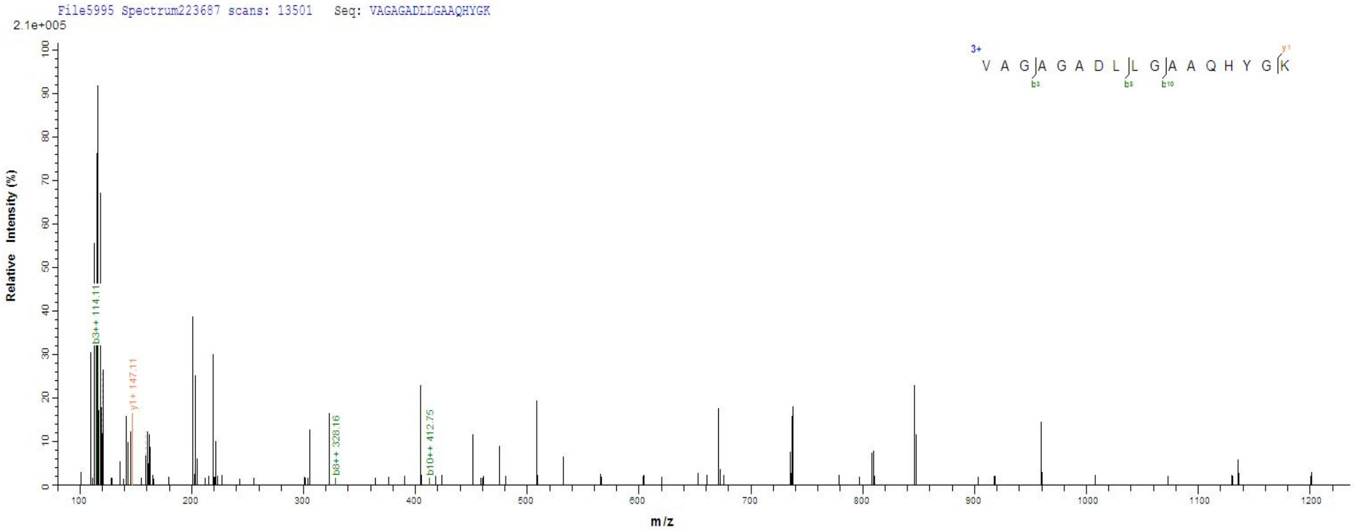

MASERDTKKGTSELLSSAKLVADAAKSTFNKESDKVDKGKVAGAGADLLGAAQHYGKLDKDKGVGQYVEKAETYLHQYQTSHSAPTTNPDSHGAGAAAKDSETAAAGGGGVGDYMKMAQGFLGK

94 Garb_06270 gi|38258655 Monocopper oxidase-like protein SKU5

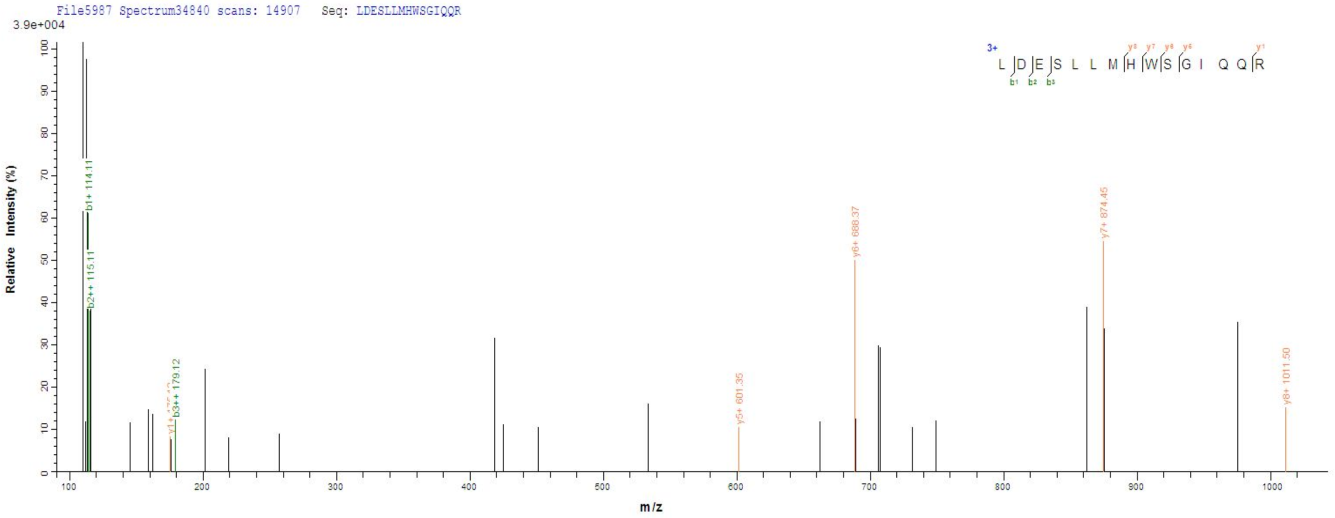

MTLSRFWGWFLIHIALFLSLCYAEDPFVFYDFEVTYITASPLGVPQQVIAINNKFPGPTINSTTNNNVVVNVRNKLDESLLMHWSGIQQRRSSWQDGVLGTNCPIPPKWNWTYQFQVKDQIGSFFYFPSLHFQRAAGGFGSFIINNRPIIPIPFDTPDGDIVILIGDWYTRNHTALRKALDAGKGLGMPDGVLINGKGPYRYNDTLVADGIEYETINVHPGKTYRLRVHNVGISTSLNFRIQSHNLLLAESEGSYTVQQNYTSLDIHVGQSYSFLLTTDQNASSDYYIVASARFVNESQWKRVTGVAILRYSNSKGKASGPLPDPPQDEFDKTFSMNQARSIRWNVSASGARPNPQGSFRYGSINVTEVYVLRNKPPETIDGKRRATLSGISFVNPATPIRLADQYKIKGVYKLDFPNKPLTGPPKMETSVINGTYRGFMEVILQNNDTKMHTYHMSGYAFFVVGMDYGEWSENSRGTYNKWDGIARSTTQVYPGAWTAILISLDNVGVWNLRTENLDSWYLGQETYVRVVNPEATNKTELPMPDNALFCGALSKLQKPQDVSSSLATSIIEGRSKLFFTVLMIASTLFLVSR

95 Garb_41622 gi|354620271 MLP

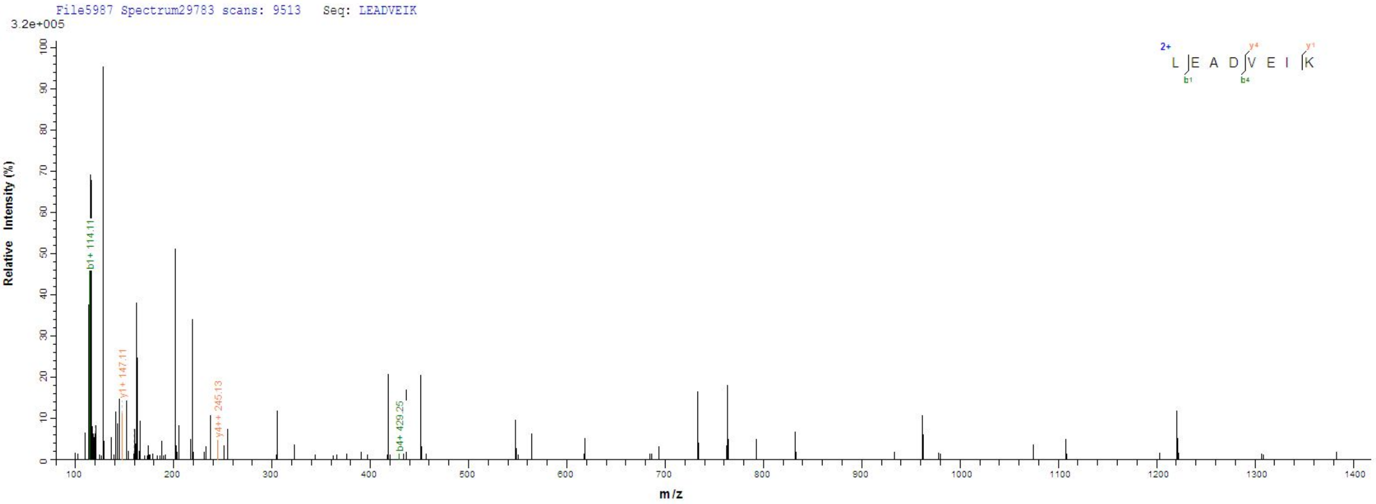

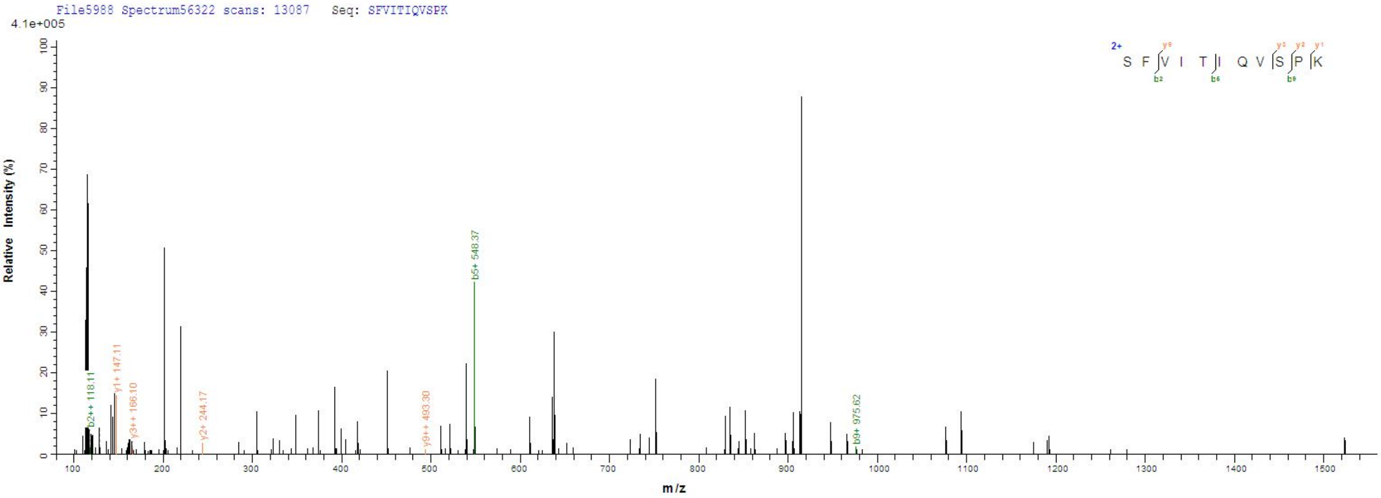

MAFSALTGKLEADVEIKASPEQFHEMFAHKPHHVHHTCYDKIQGCDLHEGEWGKVGTIVHWSYVHDGKAKKAKEVVEAVDPDKNLVTFRVIEGDLMEEYKSFVITIQVSPKSEGSGSVVHWTLEYEKLHDGIAHPETLLQFVQDVSKDIDAHLTQAS

96 Garb_14339 gi|194321204 laccase

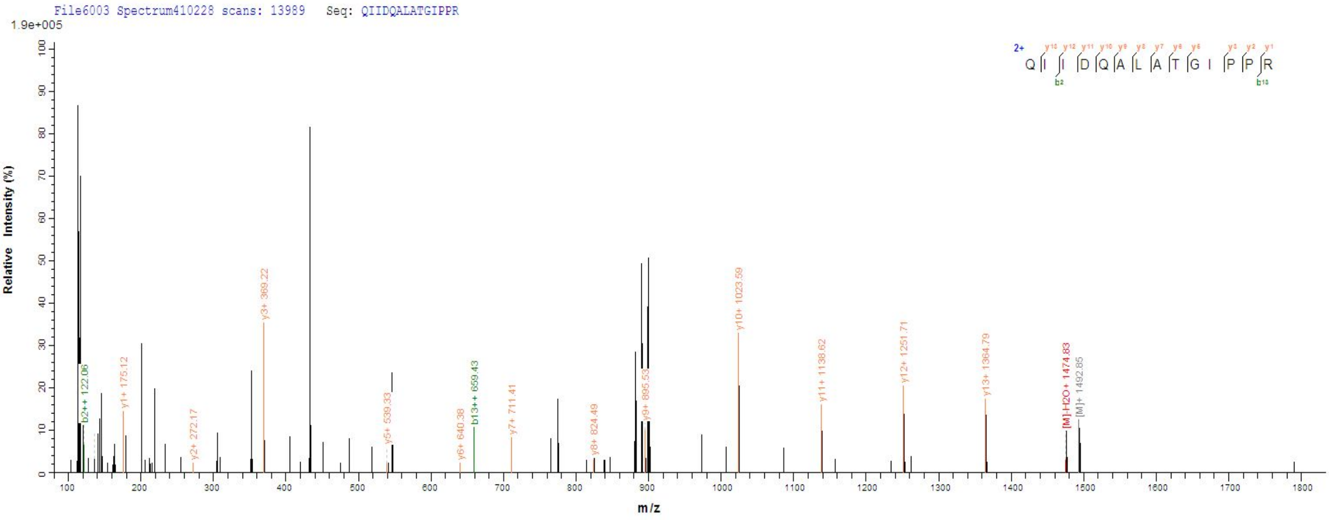

FLFQVRESNFTKLCNTTTLLVVNDSYPGPEIRVRRGDTVFVNVHNQGNYGFTIHWHGVKQPRNPWFDGPEFITQCPIQP GTNFTYEVILSDEIGTLWWHAHSDWTRGSVQGAFIILPAENETYPFPTPDADQTIILQSWYNGDYKQIIDQALATGIPPR QPDAYAINGNLGDTYGCTNDTIFRMQVDYEKMYLLRIINAAMNEQQFFSITNHTLTVVAQDASYVRRFTSDYILISPGQ TMDVLVSANQNVGQYYMATRPFSDASAMPPDNITTGIFQYTNSEGGLNASLITLPARDDTNATNSFISRIRNTNVTQNP PLNVPTGIDRRVFITIATNTVPCNTSQCLLPNRFVASLNNVSFVFPRIDILQAYYNSSTGGVFTEDFPLNPPVFYDFTGNLT GFNTRAELGTRAVVLNYGEAVEIVLQATQLGGGGSHPIHLHGFSFYRVGSGSGNFNNETDPRTYNLVDPPLINTIHVPG KGWAALRFFANNPVSNIYIYIHVSLQVKEATYTRLCSTKKILTVNGQFPGPTIEAQHGDTIYVNVYNQGKQNITIHWHG VLQPRYPWADGPEYITQCPIKPGGRFRQKIIFSTEEGTLWWHAHSDWSRATVYGAIIIHPKVGTRYPFPKPDAEV

97 Garb_20222 gi|65998365 dirigent-like protein

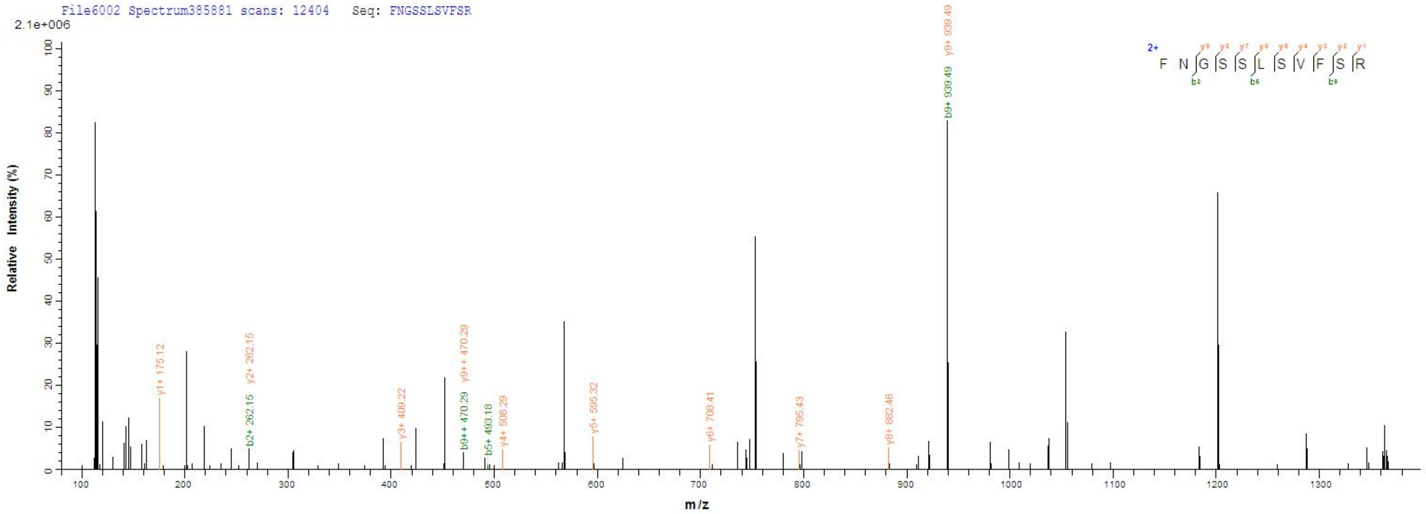

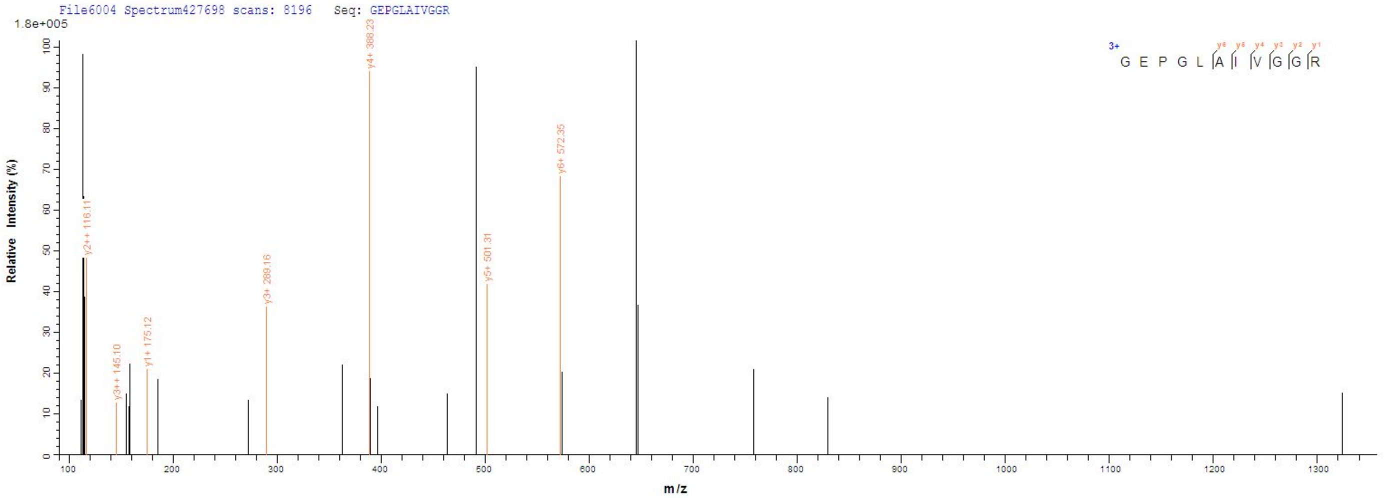

MRGSGTLMLSWVLIICLSLVGVQSQYYSETLPYRPRPVKVTNLHFFMHEFTGITAVQVAQVNITSNDNNSSVPFASLVAV NDPLRTGPEPDSELIGNVQGIALLAGMNASSTQYIDFGFNTGKFNGSSLSVFSRGEPGLAIVGGRGRFMMATGVAQFNP ILINATNVIIEFNVTVIHY

98 Garb_20230 gi|66276977 dirigent-like protein

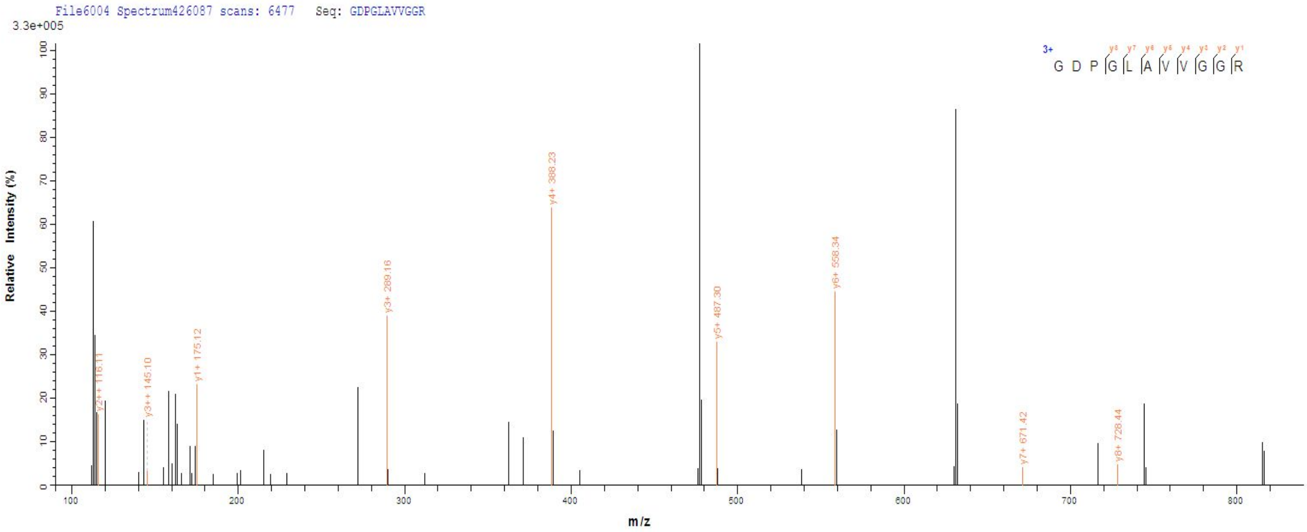

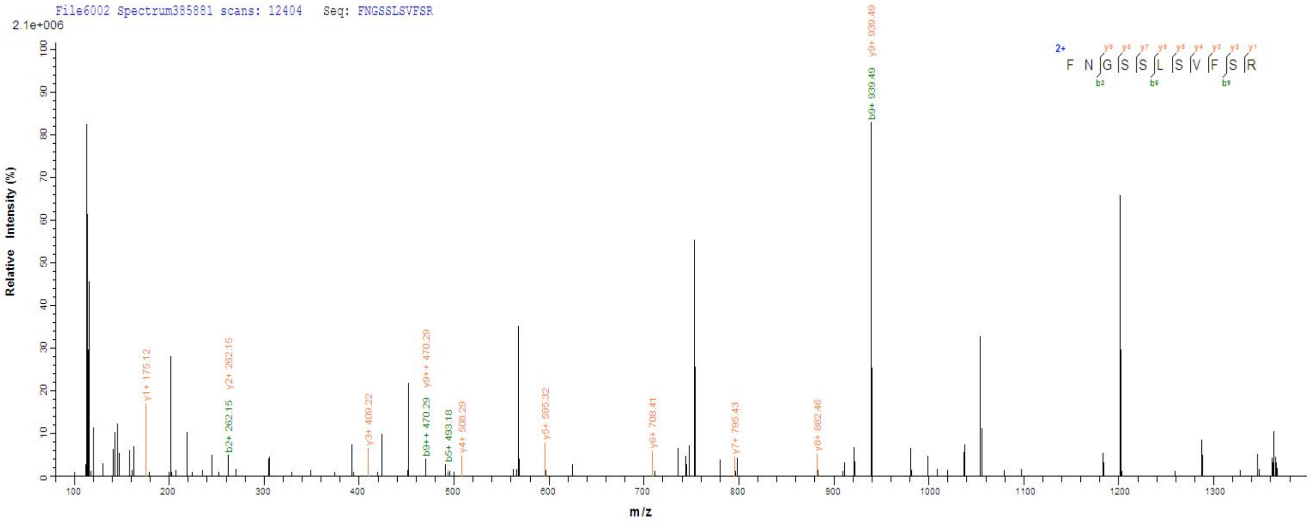

MRGTSVLSWILIICVCQVAVRSQYYSDTLPYQPRPVLVTNLHFYMHEFTGTTAVVLTQANITSNNSSVPFATLVAVNDPLRTGPEPDSELIGNVQGISLLAGSNASSTQYIEFGFNTGKFNGSSLSVFSRGDPGLAVVGGRGQFAMATGTALFNPILINATNVIIEFNFTVIHY

99 Garb_17269 gi|118926 Desiccation-related protein PCC13-62

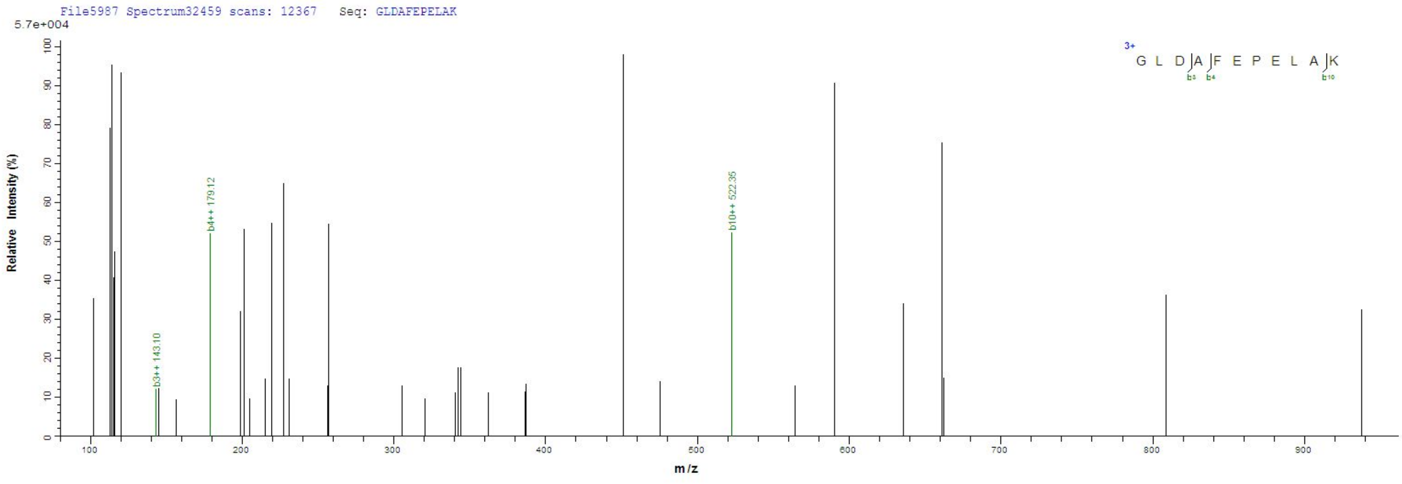

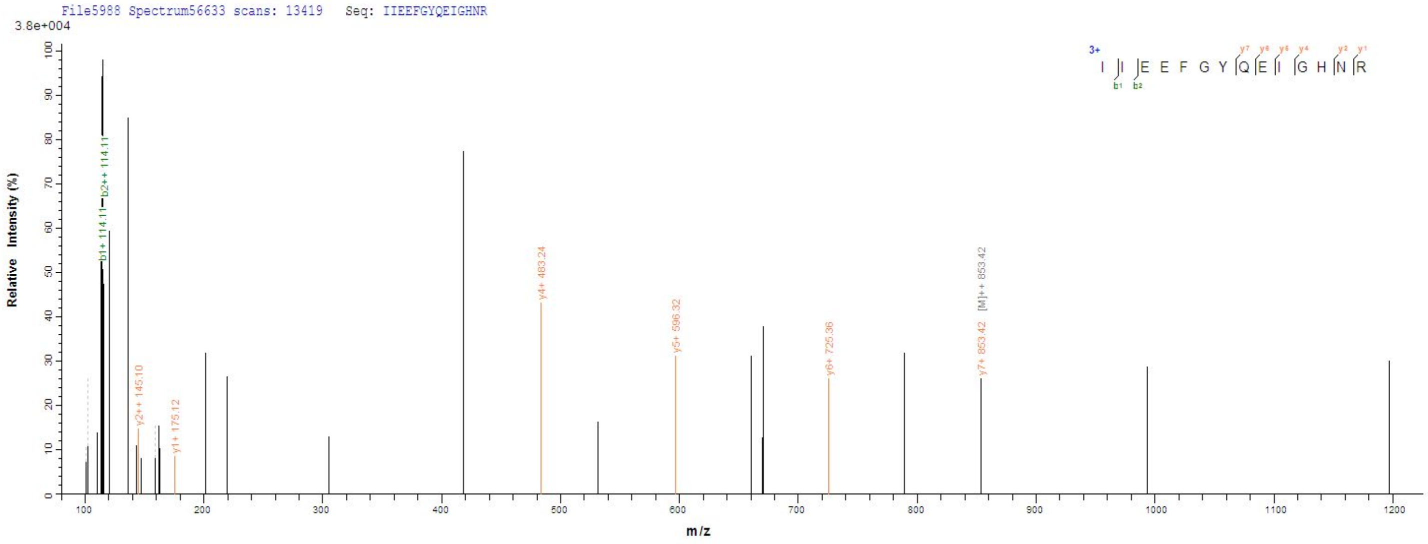

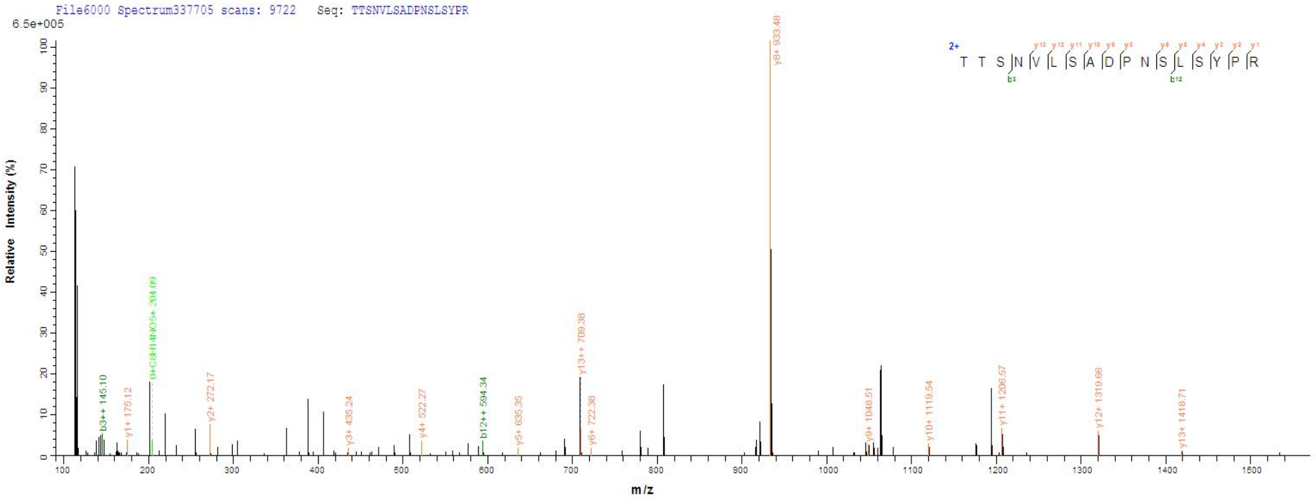

MATTTPSCPGDCEPIDADDNDRFHFAQNLEFLEAEFFLHGALGKGLDAFEPELAKGGPPPIGGRRANLDYLTRRIIEEFGYQEIGHNREIVRRTGGIPRPLIDISSQNFAKLFDKAAGYNLDPPFDPYEDPIKYMLAVYAIPYVGLNGYVGTTPCLKRFSSKQLVAGLLGVEAGQDAVTREWLYKKGDEKVEPYDITVVEFTNMISGLRNELGKCGIKDEGLIVPKELGAENRTTSNVLSADPNSLSYPRTPQEILRIVYSTGNEHRPGGFFPKGANGRIAREYLYNDQLRGL

100 Garb_40095 gi|359480830 PREDICTED: LOW QUALITY PROTEIN: L-ascorbate oxidase-like

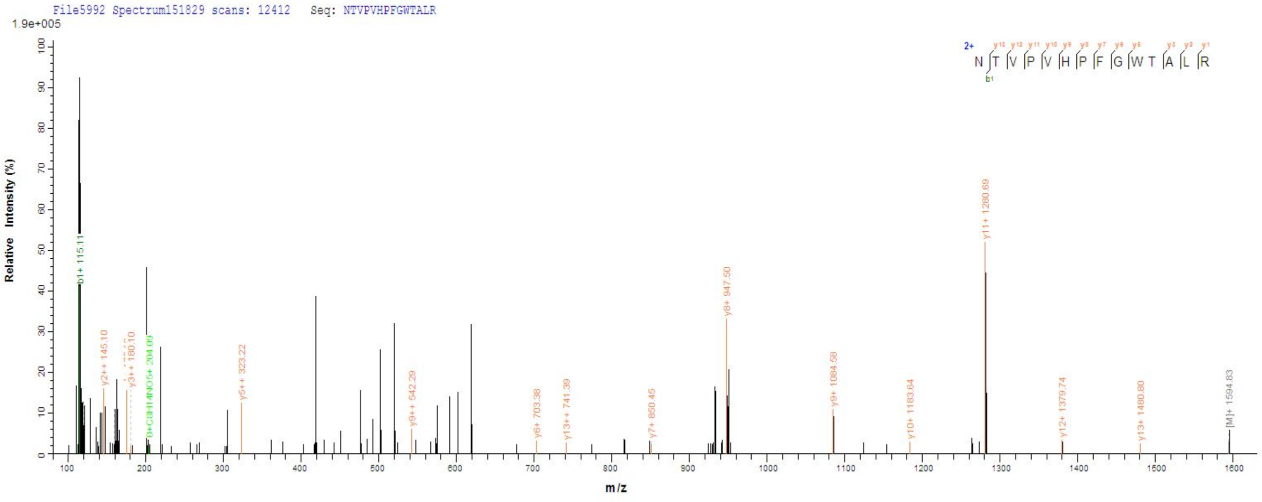

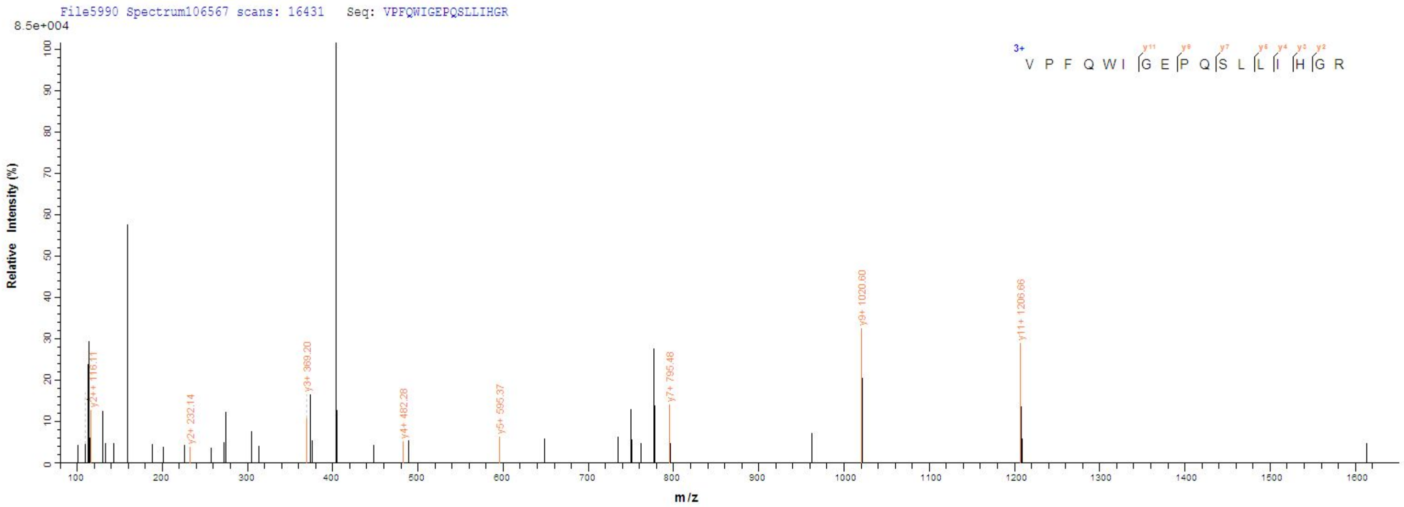

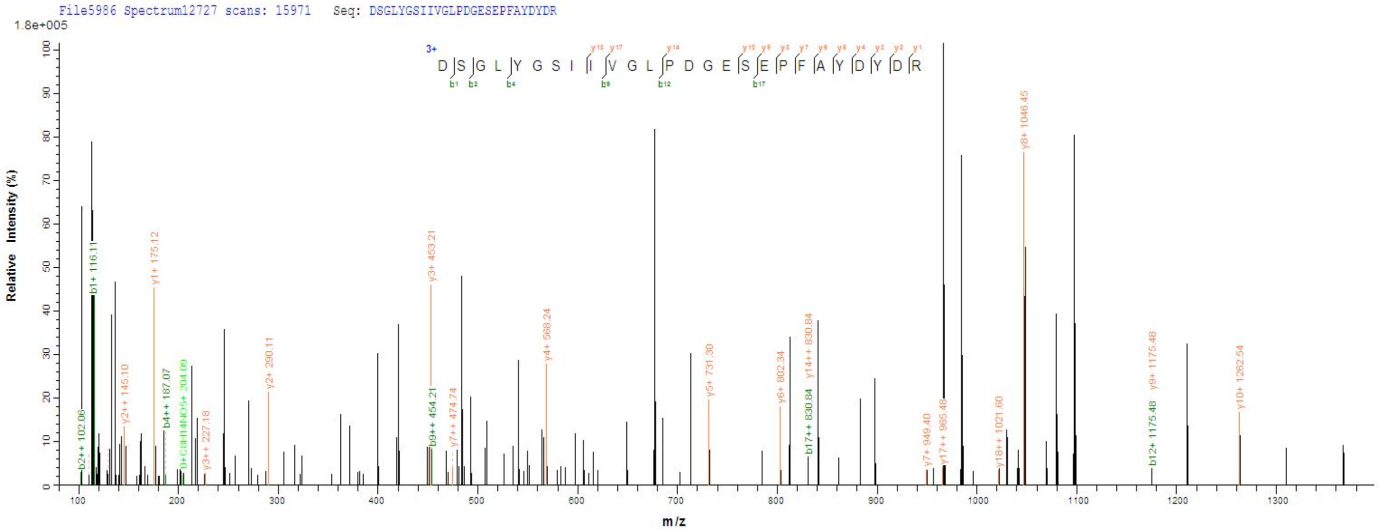

MVPVMFLLLIQLIALLNGYGVEARIRRYKWEVKNEFKSPDCYRKMVITINGRTPGPTIRAQQNDTIIVEVTNSLFTENIA IHWHGIRQIGTPWFDGSEGITQCPILPGTTFKYQFVVDRPGTYVYHAHYGMQRDSGLYGSIIVGLPDGESEPFAYDYDR NIILNDWYHNSTTEQAVGLSKVPFQWIGEPQSLLIHGRGRFNCSSLSVPSSDPGLCNTTNPDCSPFRLIVVPGKTYRLRV SSLTSLSALSFQIEGHNLTVVEADGHYVEPFVVQNLFIYSGETYSVLVKADQDPTTNYWITSNIVSRPAPNTPPGQGVLV YYPNHPRRSPPTIPPAAPVWNDSRPRMAQSQAIKARRGYIHIPPAVSDRVIVFLNTQNEINGRRRWSVNNVSFTHPHTPY LIALKHNLTQAFDQNPPPDGYDFLNYDIYERHPNANTTSSNGIYRLNFNSTMDIILQNANTMNPKNSETHPWHLHGHD FWVLGYGEGKFDMFNDPNKYNLVNPIMKNTVPVHPFGWTALRFKADNPGAWSFHCHIESHFFMGMGVVFAEGIEKV GKLPSSIMGCGQTQGL

101 Garb_17208 gi|166203457 universal stress protein 1

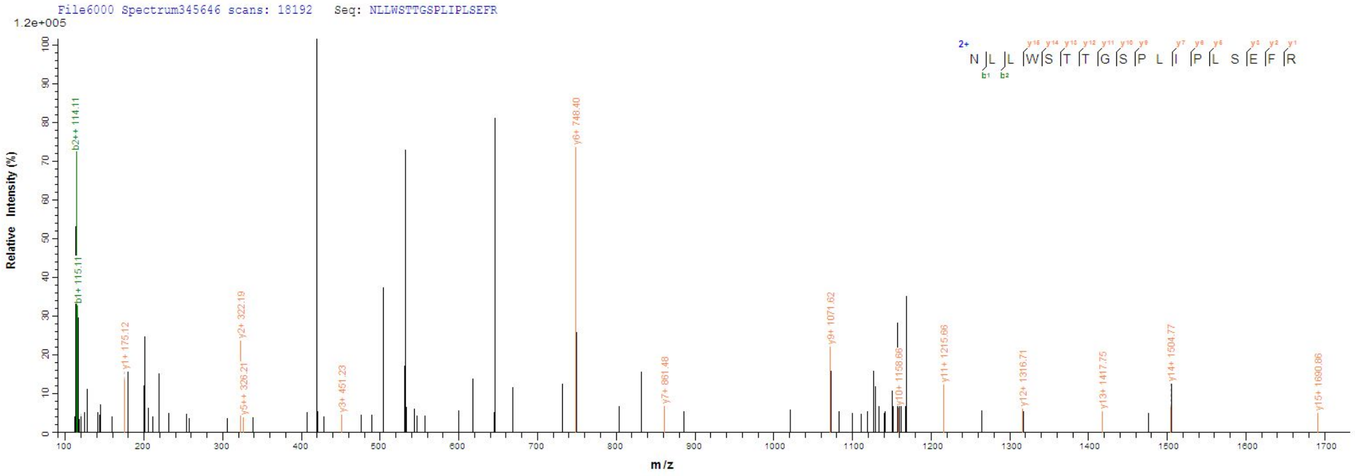

MTKDKKIGVAVDYSKGSKAALKWSIDNLLNQGDTLYLIHVKPHQSDESRNLLWSTTGSPLIPLSEFREKEVMKHYEVEPDPEILDLVDIASRQKQGTLVAKIYWGDARDKICESVEDLKLDCLVMGSRGLGTIQRVLIGSVSNYVMVNATCPVTIVKDPSAHGF

102 Garb_29702 gi|94717590 GDP-mannose 3,5-epimerase 2

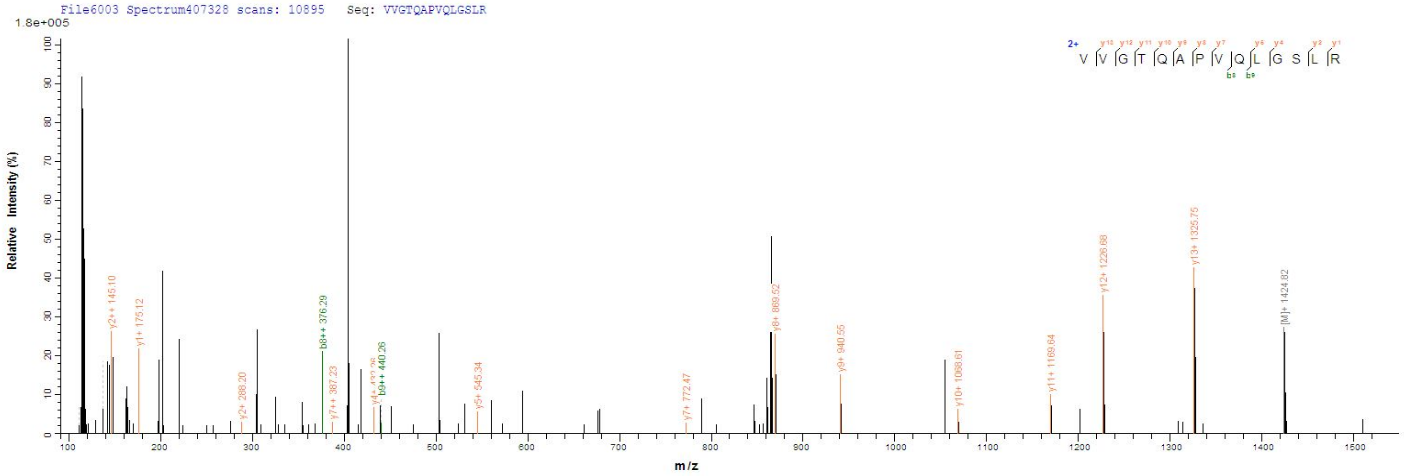

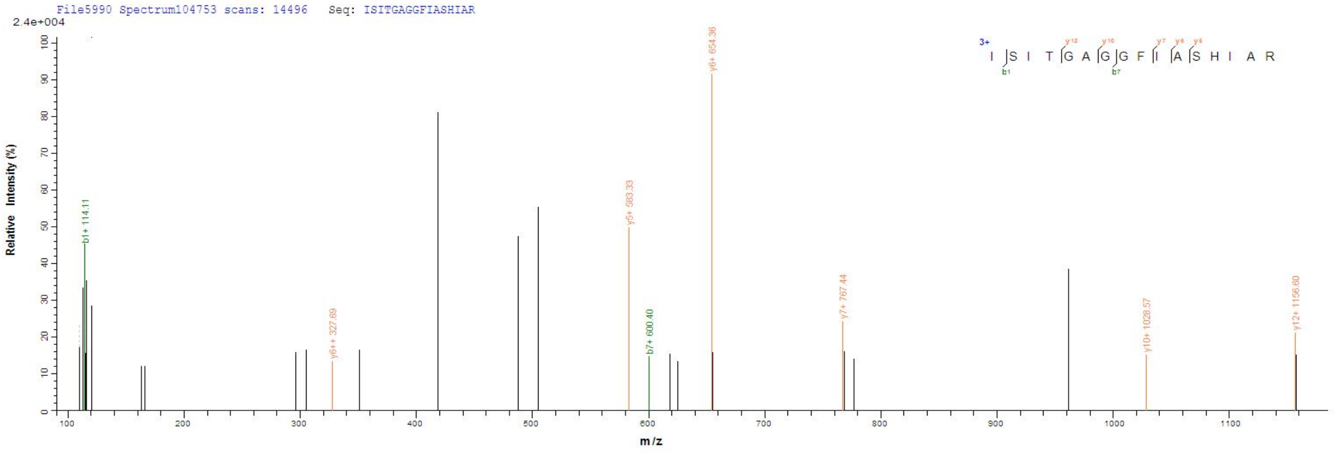

MGSTDGTSYGAYTYDALEREPYWPSEKLRISITGAGGFIASHIARRLKSEGHYIIASDWKKNEHMTEDMFCHEFHLVDLRVMENCLKVTNGVDHVFNLAADMGGMGFIQSNHSVIMYNNTMISFNMLEAARISGVKRFFYASSACIYPEFKQLETNVSLKESDAWPAEPQDAYGLEKLATEELCKHYTKDFGIECRIGRFHNIYGPFGTWKGGREKAPAAFCRKAITSIDKFEM

WGDGLQTRSFTFIDECVEGVLRLTKSDFREPVNIGSDEMVSMNEMAEIVLSFEDKKLPIHHIPGPEGVRGRNSDNTLIK EKLGWAPTMRLKDGLRITYFWIKEQIEKEKAQGIDLSVYGSSKVVGTQAPVQLGSLRAADGKE

103 cotton_GLEAN_10016101 gi|225455388 PREDICTED: germin-like protein 11-1

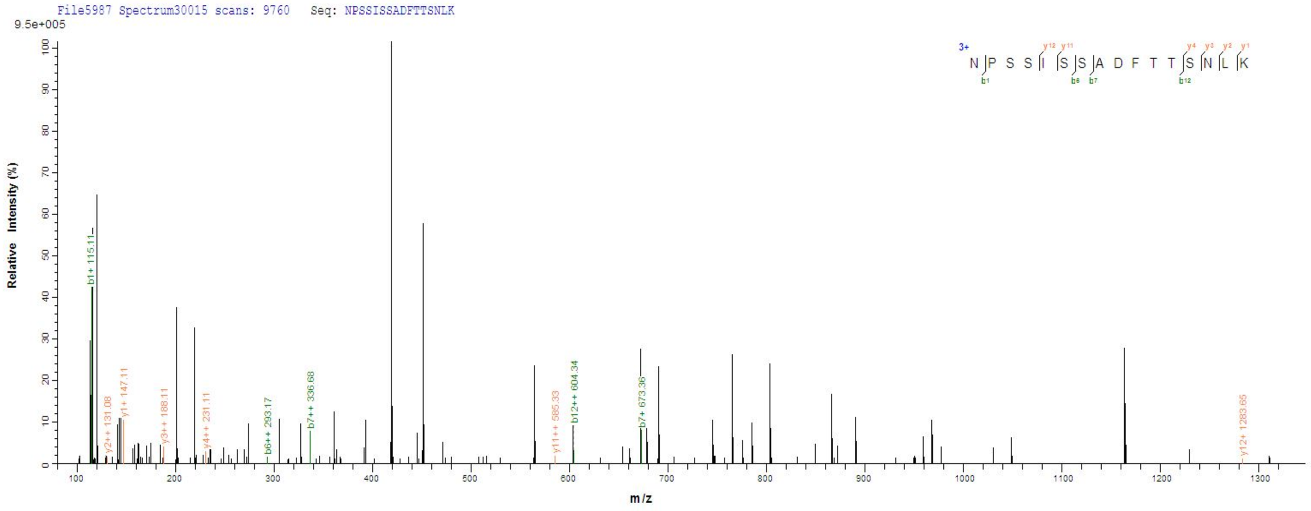

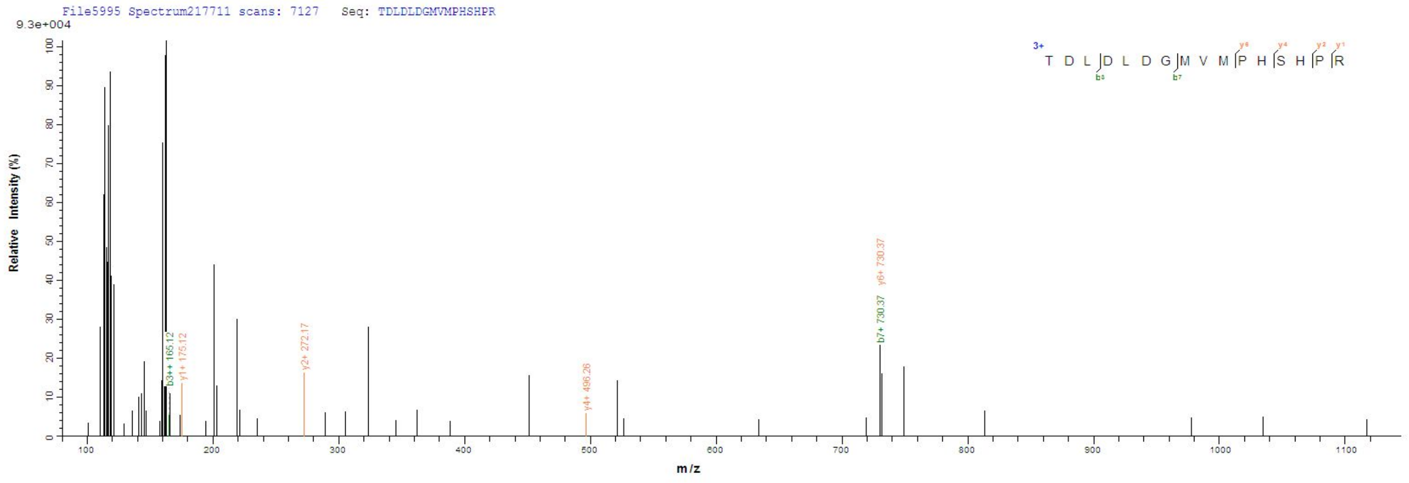

MNPTLVLFIIILCSTSISICLADNDNLQDACPTNTTVTRMVFINGFPCKNPSSISSADFTTSNLKDAGDTDNFLHSSVNIVT AADFPGLNTLGLSIARTDLDLDGMVMPHSHPRASELFFVRKGIVLAGFIDTNNNLFESLINEGDVFLFPRGLLHFCMNA GYEPAIAFSVMNSQNPGVVSIGGAVFETDKLLIDKIVRRLISVRGTNMANFSKIHIQ

104 Garb_28365 gi|470122858 PREDICTED: protein PLANT CADMIUM RESISTANCE 2-like isoform 1

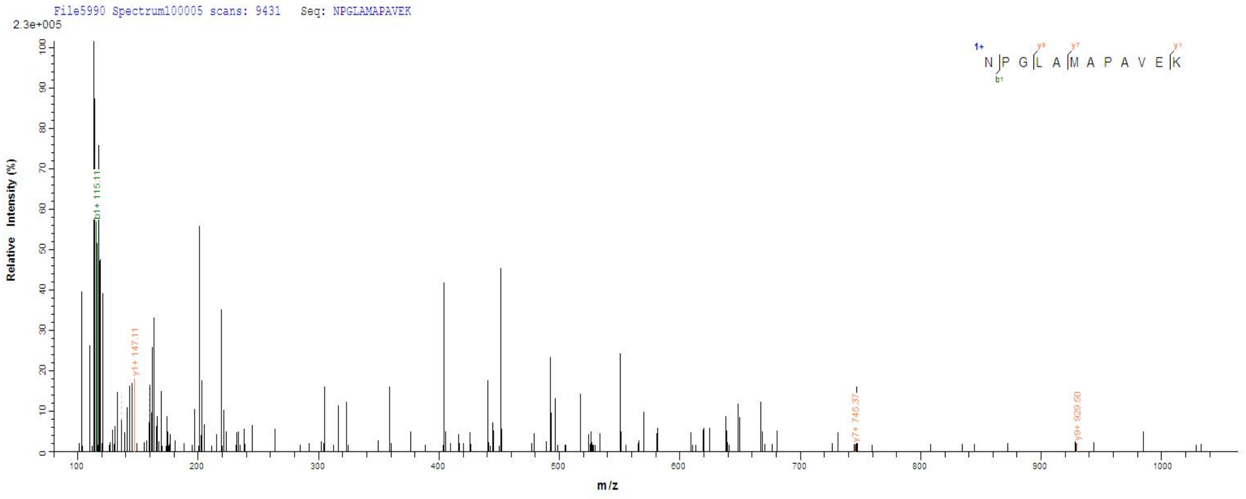

LSFLNSSTMNHPSGQPQNELPWSAGFCDCCSDLKTCCISYWCPCITFGQIAEIVDKGSTSCGASGALYTLIISCCYRSKLRKQYNLKGGNCGDCMRHFCCEPCALTQEYRELQNRGFDMSIGWHANVEKNPGLAMAPAVEKGMSK

105 Garb_16483 gi|75099392 Subtilisin-like protease

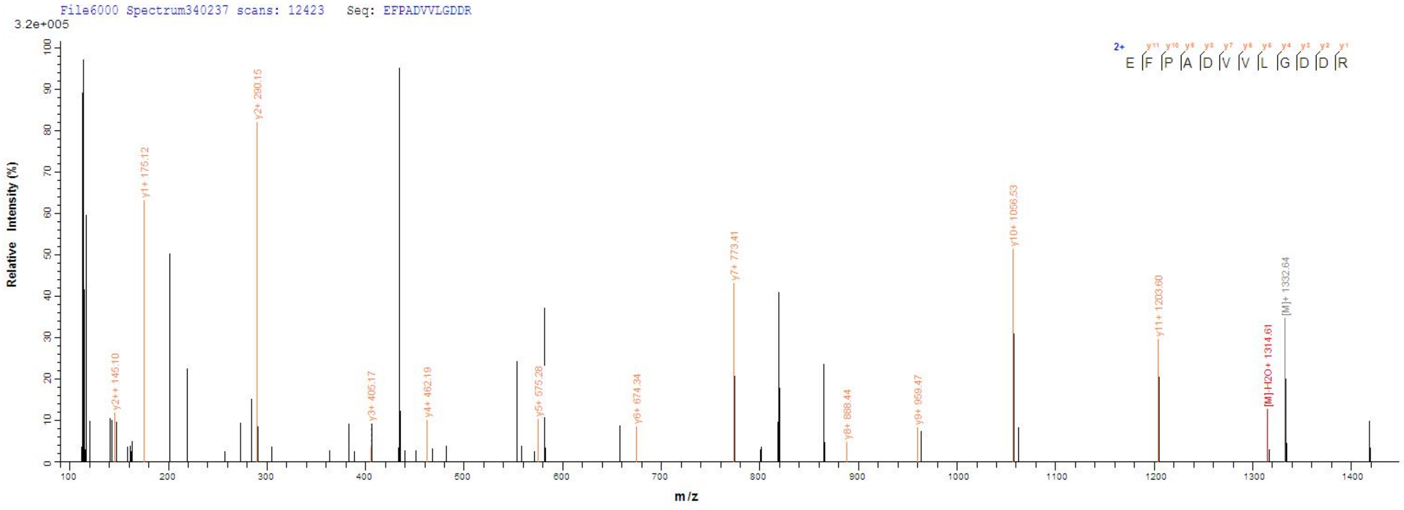

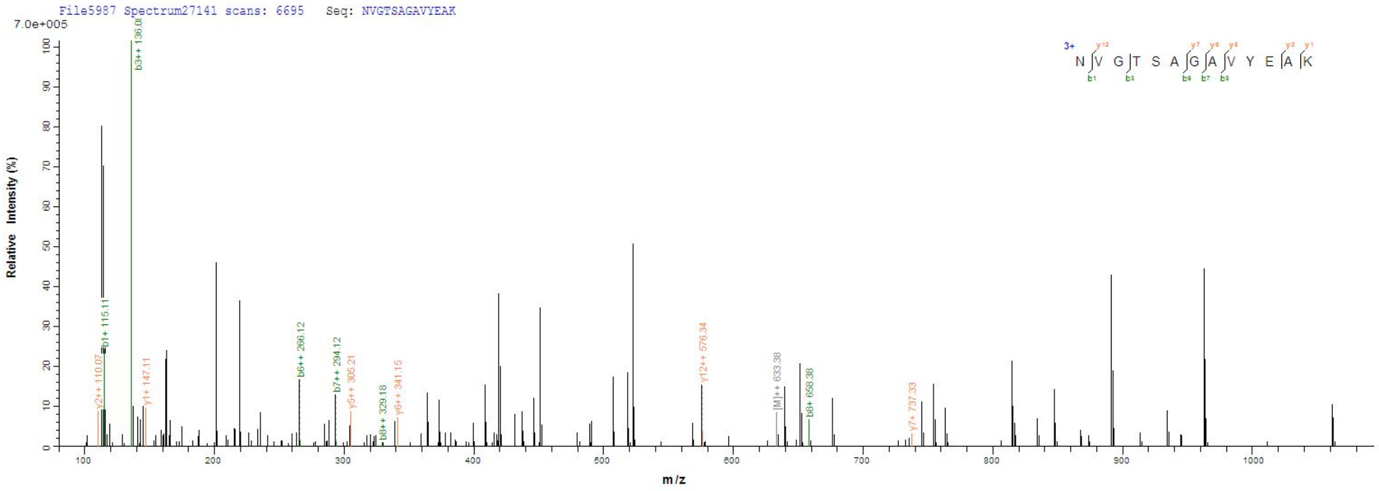

MVSNASLFEFAYGEARGMATNARIAAYKICWKMGCFDSDILAAMDQAIADGVDVISLSVGATGYAPQYDHDSIAIGAFGAANNGIVVSCSAGNSGPGPSTAVNIAPWILTVGASTIDREFPADVVLGDDRIFGGVSLYSGEPLGDSKLPLVYGGDCGDRYCHMGSLNSSKVEGKIVVCDRGGNARVEKGGAVKLAGGLGMILENTADNGEELISDAHLIPATMVGEAAGNKILEYIKTTQFPTATISFRGTVIGPSPPAPKVAAFSSRGPNHLTPEILKPDVIAPGVNILAGWTGAAAPTDLDIDPRRVDFNIISGTSMSCPHVSGLAALLKKAYPNWSPAAIKSALMTTAYNLDNSGHTINDLATGEEASPFIYGAGHVDPNRALNPGLVYDIDNSDYIAFLCSIGYDSKRIAVFVREPISSDVCATKLATPGDLNYPSFSVVFNSNDHVVKYRRKVKNVGTSAGAVYEAKVNAPPGVKISVSPSKLEFSAVNKTLSYTVSFASDGLGLSSVESQGFGSIEWSDGVHLVRSPIAVRWIQGVQEESF

106 cotton_GLEAN_10008709 gi|68064400 Thaumatin-like protein

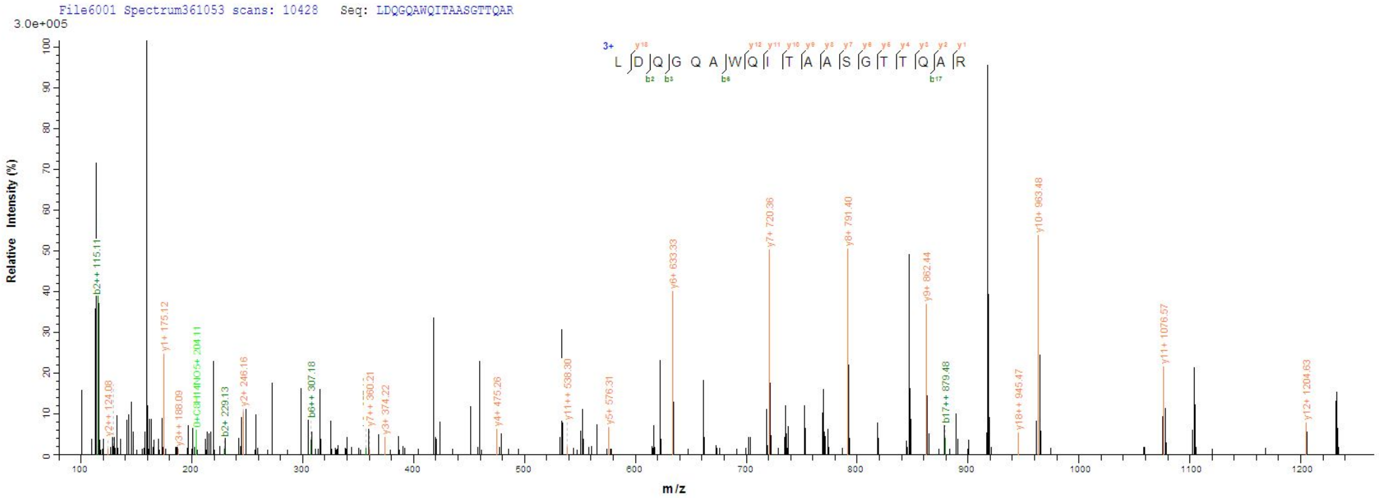

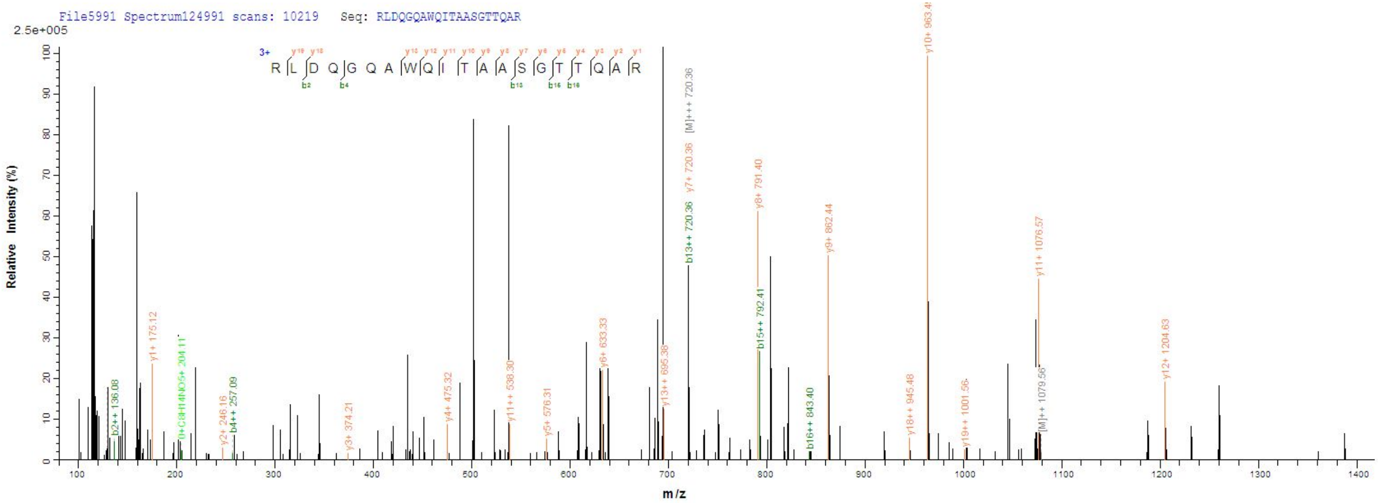

MNPFKTLPTLSFLFLTLFSLAHAATFDIRNNCPYTVWAAASPGGGKRLDQGQAWQITAASGTTQARIWARTKCNFDASGKGSCETGDCGGVLECKGYGKAPNTLAEYAIDQFEHQDFIDISNIDGFNVPMEFSSNSRGCTRVIKCTADIVGQCPNQLKVPGGCNGPCPVFKTEEHCCNSGNCQPTDFSRFFKERCPDAYSYPKDDPTSLFTCPTGTNYKVIFCP

107 Garb_26040 gi|319433441 copper binding protein 3

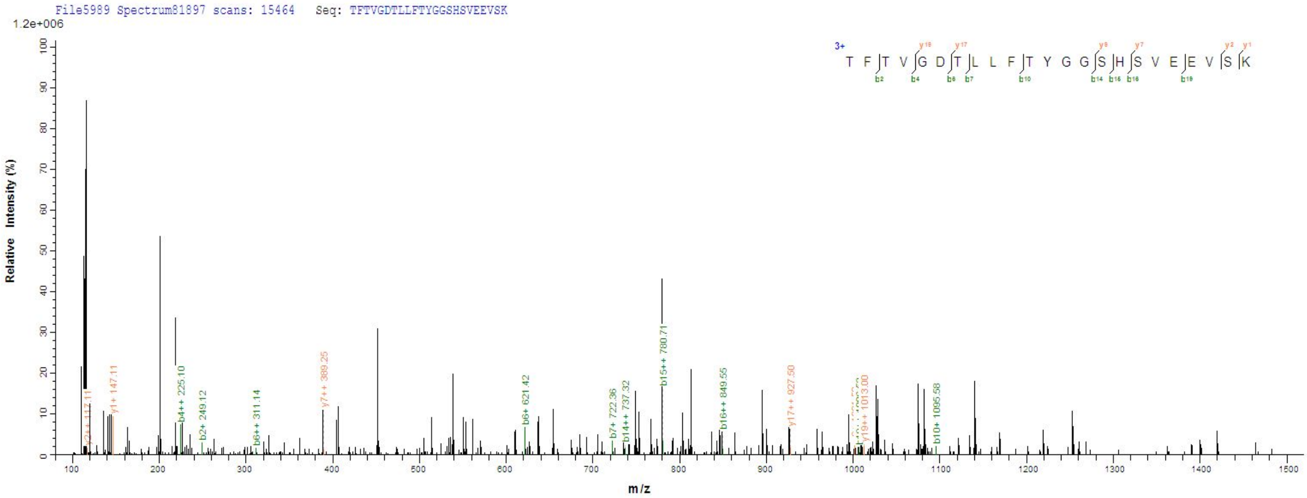

AAALLFLLLAAPAAFAEQYTVGDSSGWTTTGDYQSWVQGKTFTVGDTLLFTYGGSHSVEEVSKSDYDNCNTGNAIKS
YSDGNTVITLSNPGAMYFICPTIGHCAGGMKLAINVVAASGNSPSTPSTPSGSTTPSGTPPSGGTTTSPPSTPSGAPSTVN
YGFMVALLGAVVGIMS

108 cotton_GLEAN_10021644 gi|89258498 short chain alcohol dehydrogenase

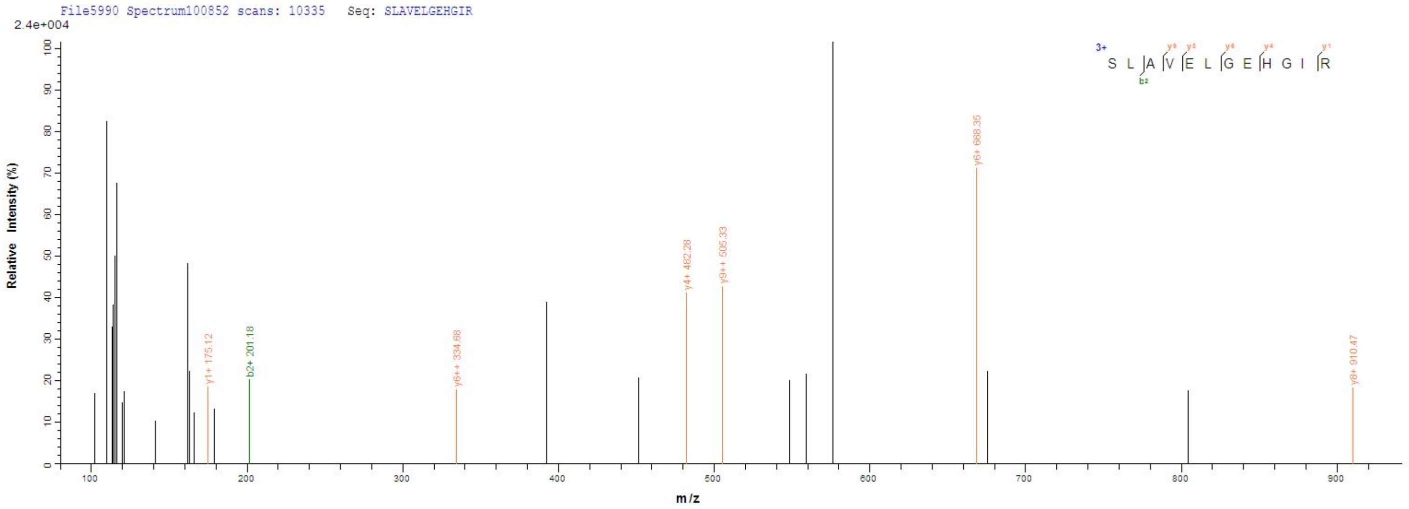

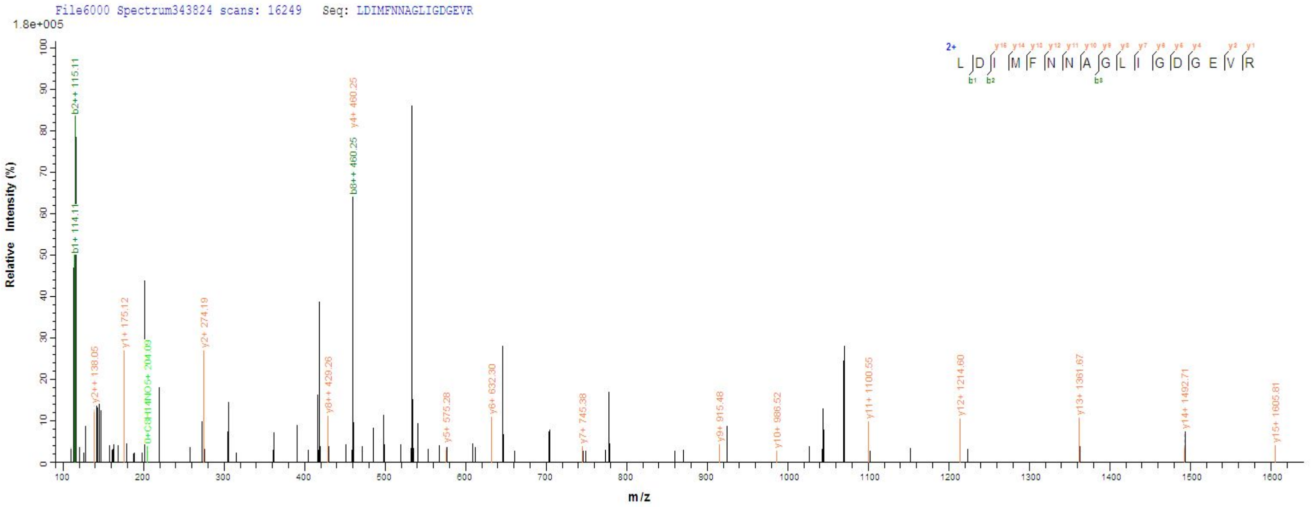

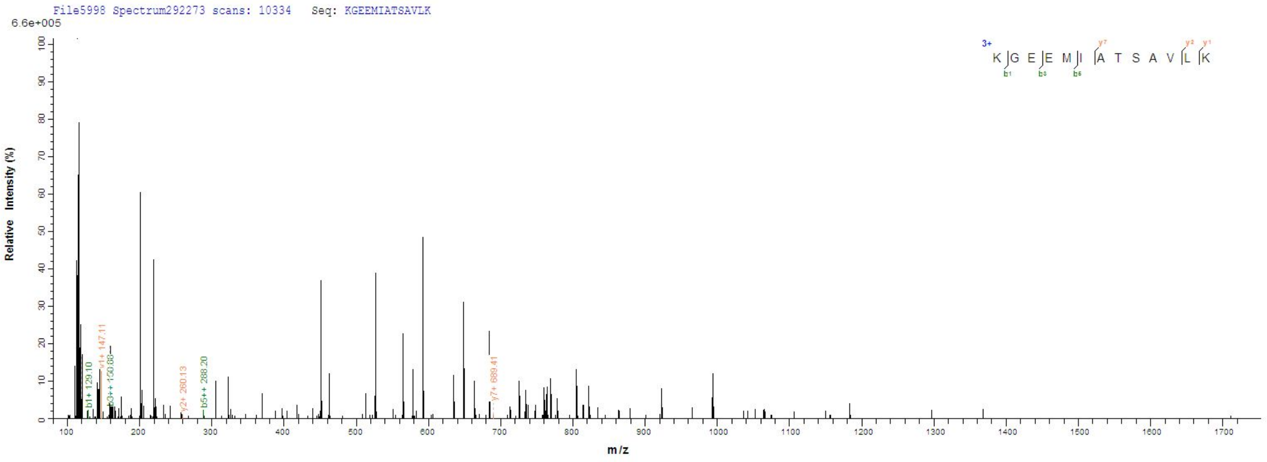

MSSESSMIKRLDGKVALITGGASGLGECSARLFVKQGAKVLIADIQDELGHSICQELGTENISYVHCDVTCESDVENAV
NLAVSKYGKLDIMFNNAGLIGDGEVRVTDASTDNFKRVFDINVLGGFLGAKYAAKVMVPAKKGCILFSSSISSKISIGLP

HAYKASKHGVVGLTKSLAVELGEHGIRVNCISPHATVTPLFLTTLGMFDKKKGEEMIATSAVLKGTVLEPEDFAHAALY LASDEAKFISGVNLPVDGGYSLSNQSWKMGFAALFG

109 Garb_09190 gi|7546402 Chain A, Structures Of Adenylosuccinate Synthetase From Triticum Aestivum And Arabidopsis Thaliana

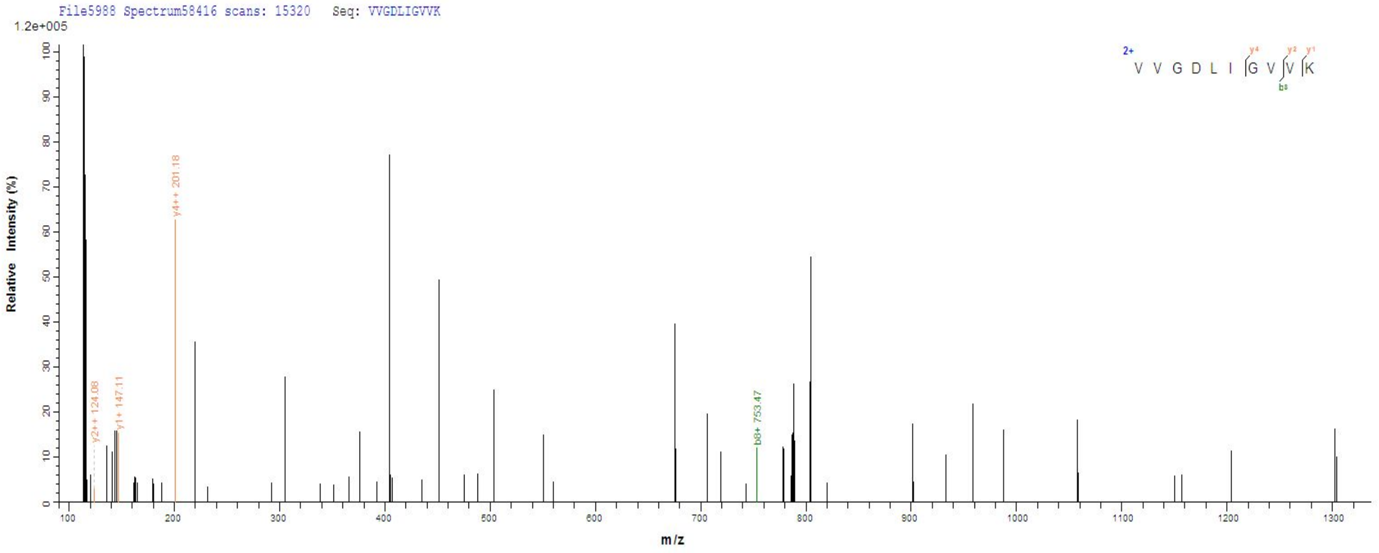

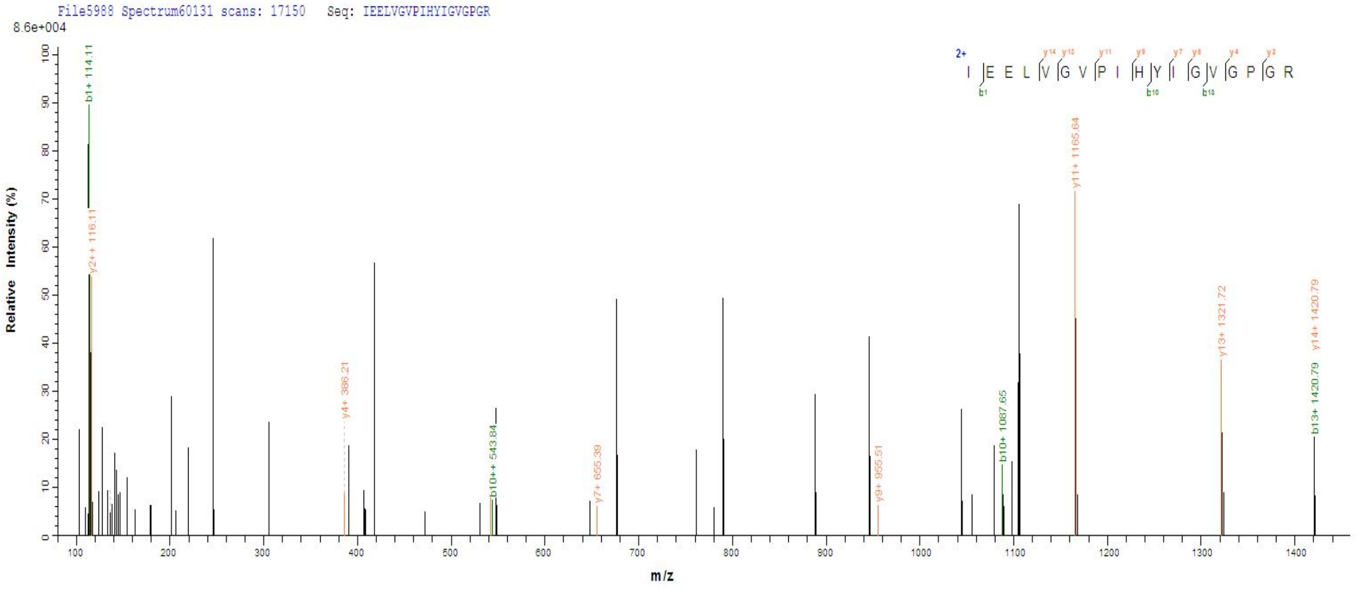

MNCSSLVLDPTPVATSRCSYRRPTLHFQHHRRNFVFCSLKPVASSSLTVAESASSESLNRIGSLSQVSGVLGSQWGDEGK GKLVDILAQHFDIVARCQGGANAGHTIYNSEGKKFALHLVPSGILNEETLCVIGNGVVVHLPGLFKEIDGLEANGVSCK GRILVSDRAHLLFDFHQVVDGLREAELNKSFIGTTRRGIGPAYSSKMNRNGIRVSDLRHMDTFPQKLHNLLSDVTSRFP DFNYTPEMLKEEVENYKRYAERLEPFIADTVHVMNESIAQKKRILVEGGQATMLDIDFGTYPFVTSSSPSAGGICTGLGI APRVVGDLIGVVKAYTTRVGSGPFPTEILGQGGDILRFAGQEFGTTTGRPRRCGWLDVVALKFCCEINGFSSLNLTKLD VLSDLPEIKLGVAYKHVDSTPVESFPADLQLLEQLKVEYEVLPGWQSDISSIRNYSDLPKAARQYVERIEELVGVPIHYI GVGPGRDALIY

110 Garb_03326 gi|74273629 gibberellin 20-oxidase 1

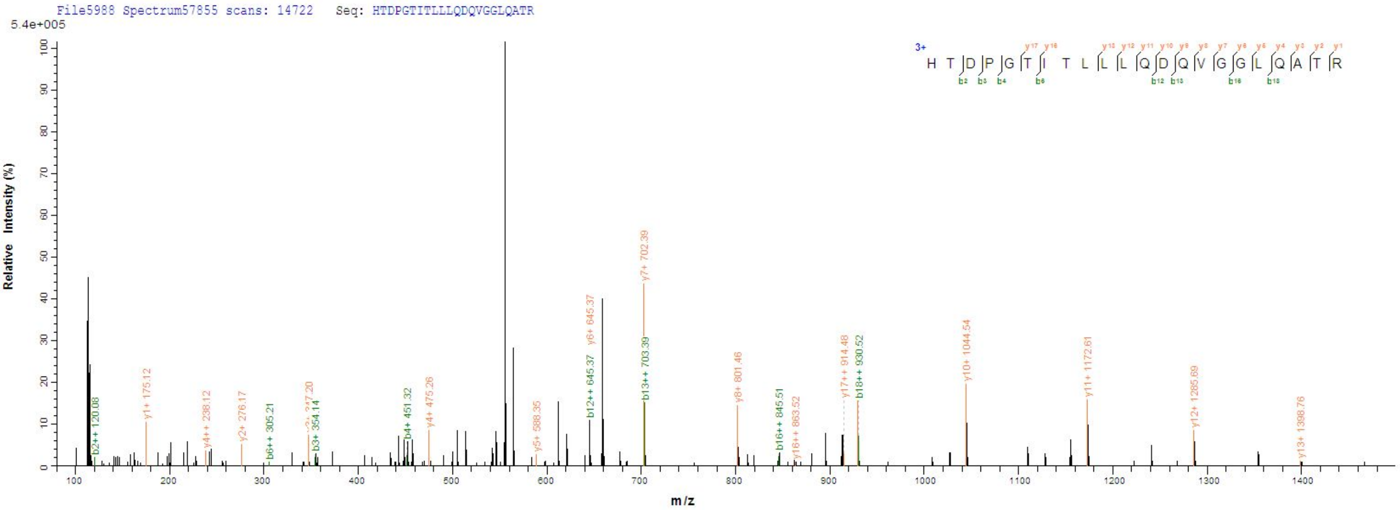

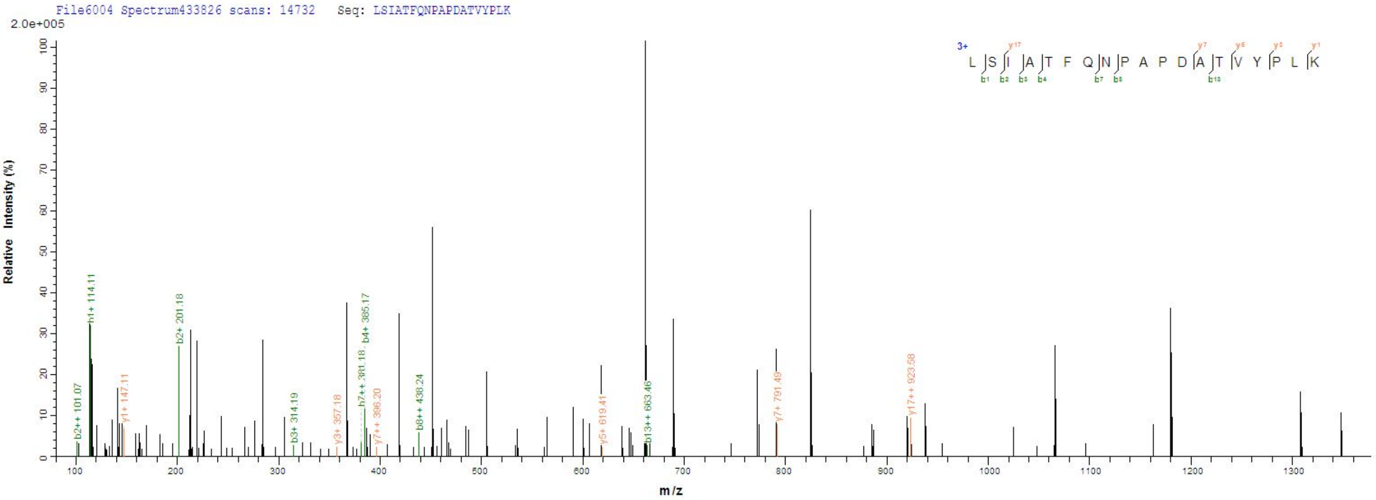

MAPSTLTALAEEKTLQASFVRDEDERPKVAYNQFSNDIPVISLAGIDDVDGKRGEICKKIVEACEDWGVFQVVDHGVDTKLVSEMTRLAREFFALPAEEKLRFDMSGGKKGGFIVSSHLQGEAVQDWREIVTYFSYPLRSRDYSRWPDKPQGWVEVTKEYSEKLMGLACKLLEVLSEAMGLEKEALTKACVDMDQKVVVNFYPKCPQPDLTLGLKRHTDPGTITLLLQDQVGGLQATRDNGKTWITVQPVEGAFVVNLGDHGHYLSNGRFKNADHQAVVNSDCSRLSIATFQNPAPDATVYPLKIREGEKPILEEPITFAEMYRRKMSKDLELARLKKLAKEQQQLKEKEAENEKPKLEAKPLEEILA

111 cotton_GLEAN_10034568 gi|255554698 homogentisate 1,2-dioxygenase, putative

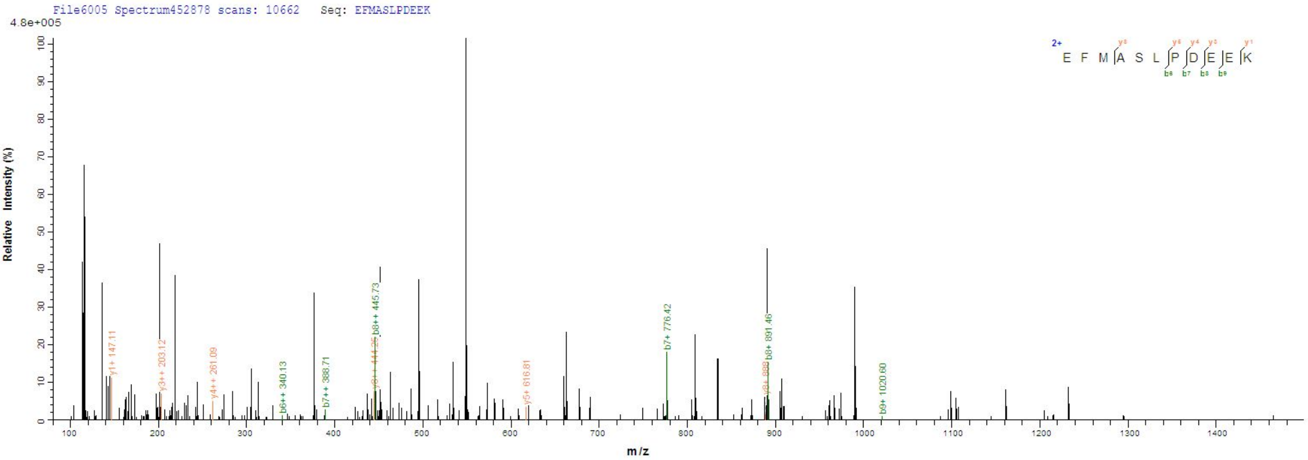

MLLVSLLICHLLSKVPATSLEPPKHFVLVHGSCHGAWSWYKVVPLLKSGGHNVTALDLGGSGVDPQQVNTLRSISDYI
KPLREFMASLPDEEKVVLVGHSLGGLAISQAMEMFPEKVAVAVFVTASMPGPTLNVSILIQKALRDQDSQMDNHYTYD
DGPSSPPTTFTFGPMFLSSKVYQLSPPEDSALASMLMRPIRLYSAEDMSREVVLSQKKYGSVNRVFIISEKDLVSKEDFV
GWMIRENPPRQVEVIKGSDHMVMMSKPIQLSKLLLCLATNYSQR

112 Garb_20016 gi|255554698 homogentisate 1,2-dioxygenase, putative

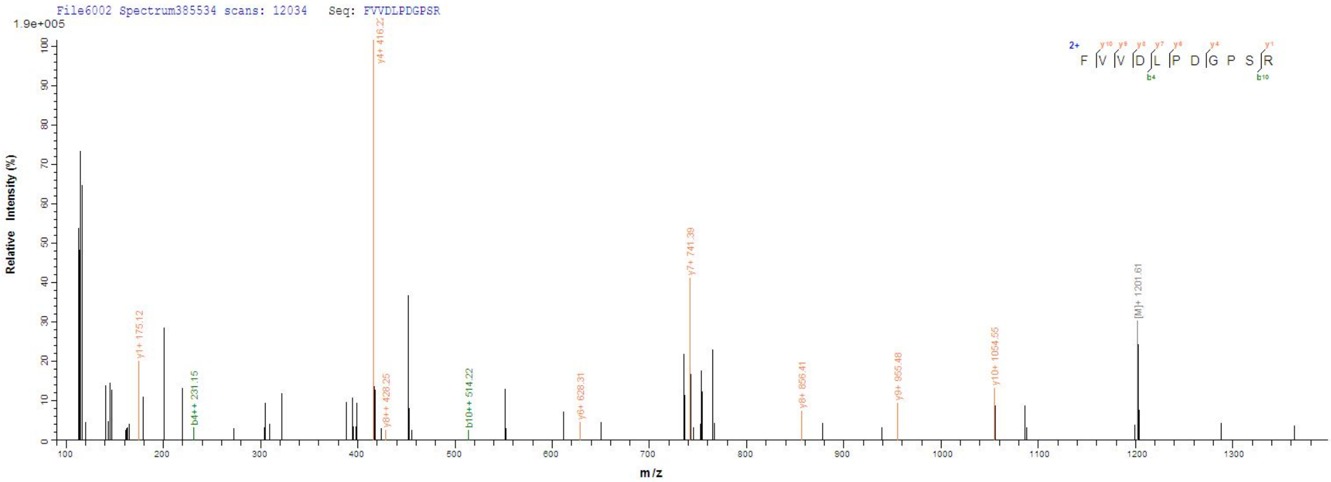

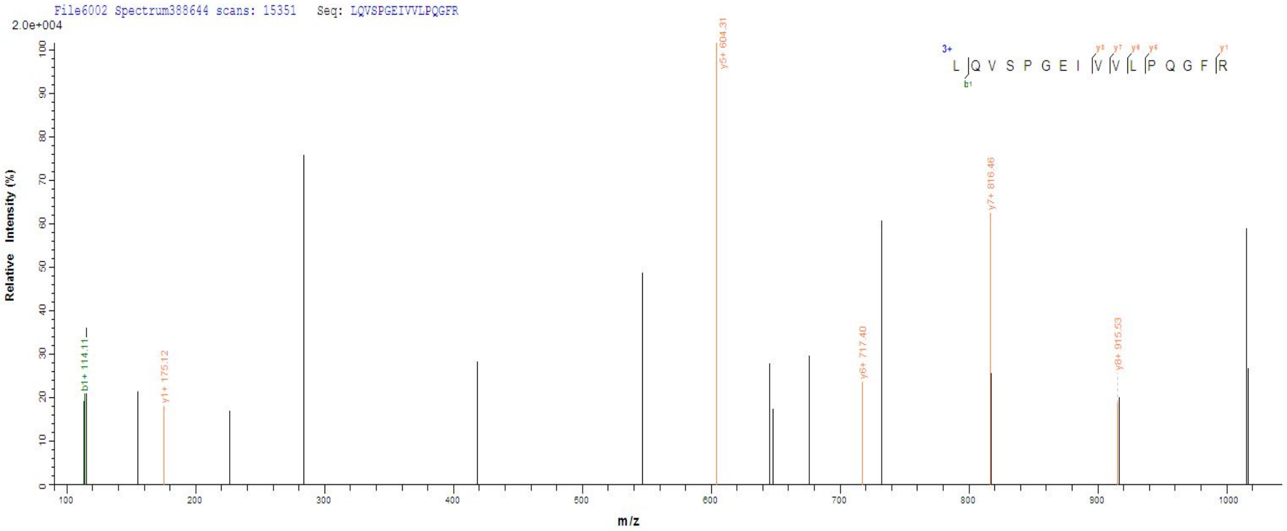

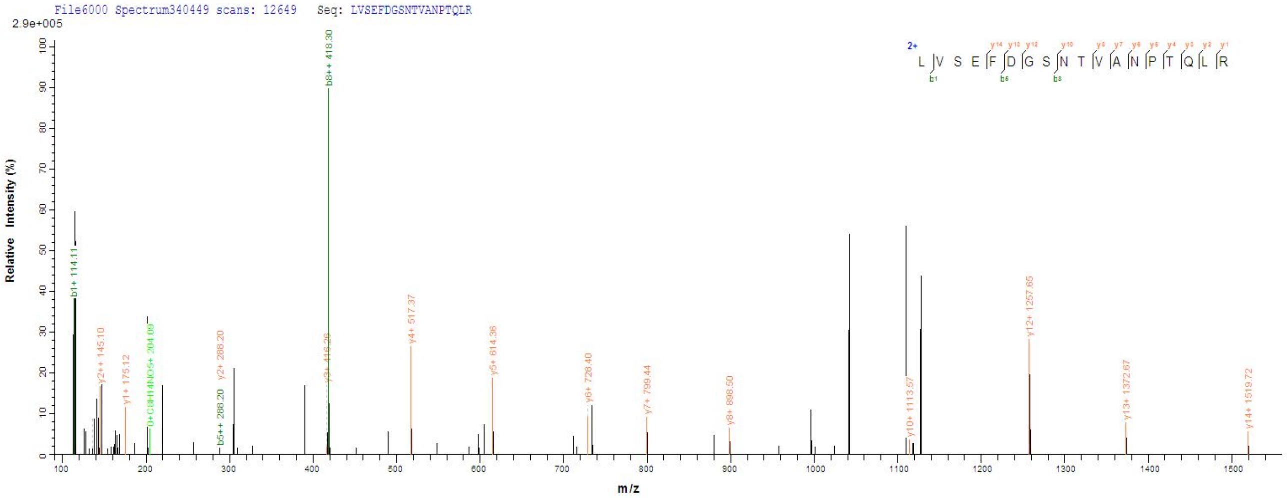

MEKPAAEVIKGEGWGRFPDDLEYHSGFGNHFESEAIAGALPRGQNSPLICPFGLYAEQISGTSFTSPRKLNQRSWFYRIK
PSVTHEPFWPREPSHKKLVSEFDGSNTVANPTQLRWKPVDIPDSPTDFIDGLYTLCGAGSSFLRHGFAIHMYTANKSMD
NCAFCNADGDFLVVPQQGRLWITTECGRLQVSPGEIVVLPQGFRFVVDLPDGPSRGYVAEVFGTHFQLPDLGPIGANG
LAASRDFLAPTAWFEDCPRPGFTIIQKFGGELFNARQDFSPFNVVAWHGNYVPYKYDLSKFCPYNTVLIDHGDPSINTV
LTAPTDKLGVALLDFVIFPPRWLVAEHTFRPPYYHRNCMSEFMGLIYGGYEAKADGFLPGGASLHSCMTPHGPDTKTY
EATVARGNEAGPHKITDTMAFMFESCLMPRTCPWALDSHFRDLDYYRCWIGLKSHFSHDKTDVKSNDLQNGNKSGCF
EH

113 Garb_30190 gi|3183454 Uncharacterized oxidoreductase ykwC

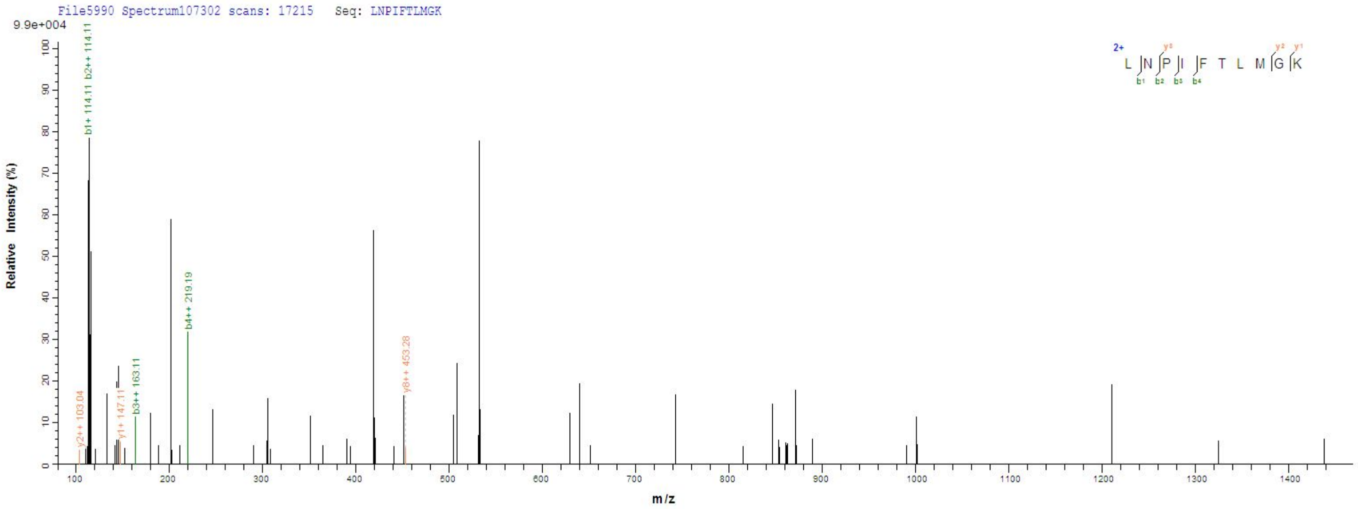

MRSLSYLLGSLSRYFPHSQSSSTPHLTFLLRSMATSAAAGPINPSNTRLGWIGTGVMGRSMCAHLINAGYSLTVFNRTLS
KAQPLVDMGARLAQTPHDLAAQSDVVFSIVGYPSDVLQVLLDPFNGALSGLRPGGILVDMTTSEPSLAVEISTAASSKN
CSSIDAPVSGGDRGAKNGTLAIFAGGDEAIVNRLNPIFTLMGKVNYMGPSGKGQFAKLANQITIASTMVGLVEGIIYAH
KAGLNVASFLDAISTGAAGSKSLDLYGSRILKRDFDPGFFVNHFVKDLGICLKECQNMGLALPGLALAQQLYLSLKAH
GEGNLGTQALILALERLNNATLGS

114 cotton_GLEAN_10009019 gi|224137260 predicted protein

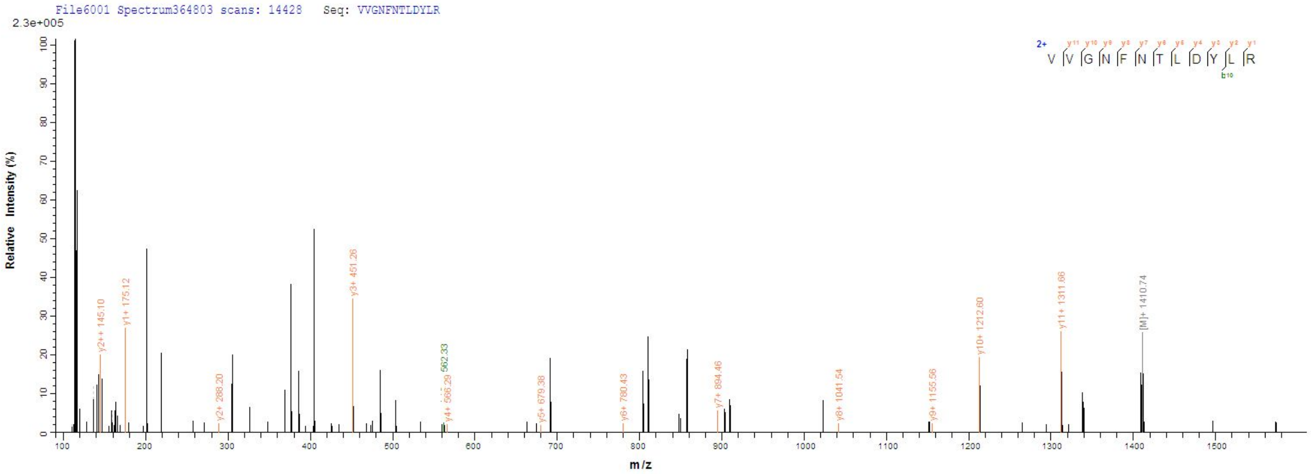

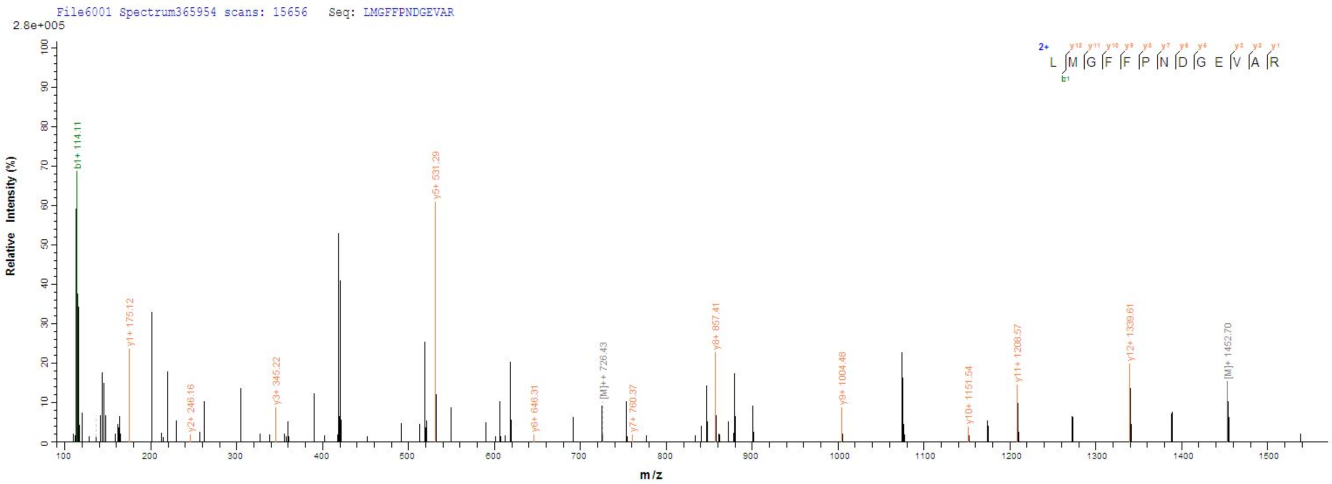

MNTDITASAKPEYPVIDRNPPFTKVVGNFNTLDYLRFTTITGVSVTVGYLSGIKPGLKGPSMVTGGLIGLMGGFMYAYQNSAGRLMGFFPNDGEVARYRKRGLKN

115 Garb_05122 gi|297736988 unnamed protein product

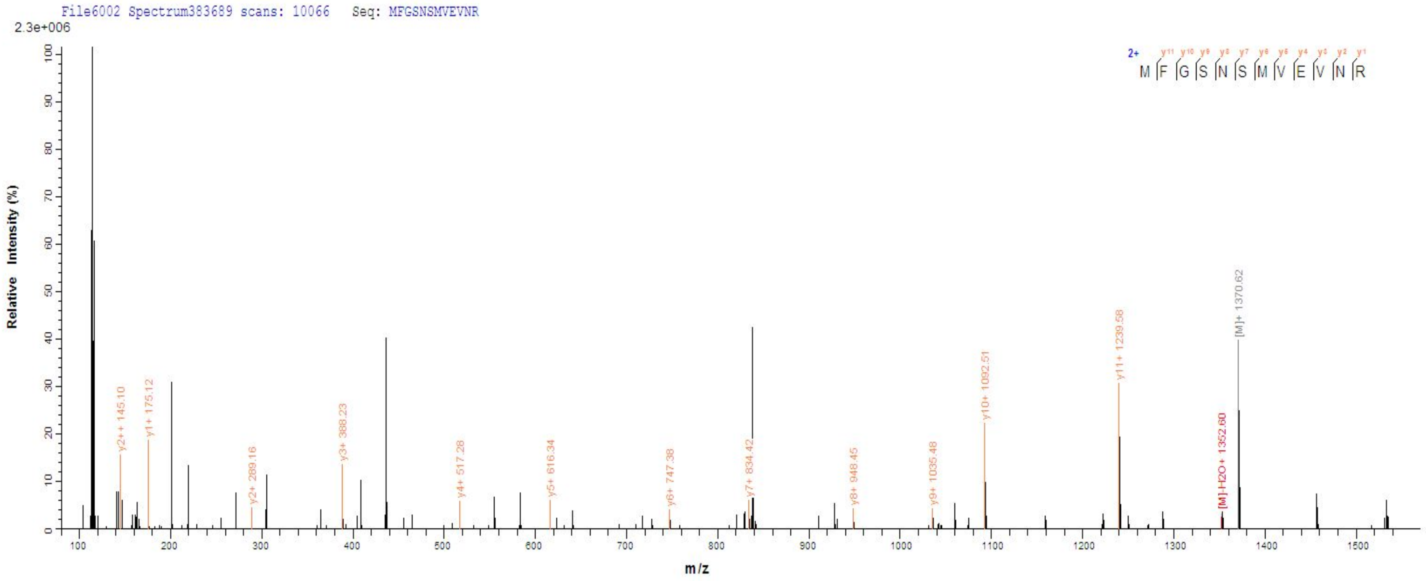

MASLHCFLFTCLLFFFSSPSIAQQSFRPKALVIPVAKDASTLQYLTTISQRTPLMPIDLVVDLGGRHMWVDCDRDYVSSTYRPARCRSAQCSLAGADGCGDCFSAPRPGCNNNTCGVTPDNSVTHVATSGEVAQDIVSIQSTDGKNPGRVVSVPRFLFACAPSFLLQGLATGVVGMAGLGRTRIALPSQFAAAFSFHRKFAVCLSSSTSANGVIFFGDGPYVFLPGVDASQSLTYTPLFINPVSTASAFPMGEASAEYFIGVKSIKVNENSISLNTSLLSINNEGVGGTKISTVDPYTVLESSIFKAVTEAFINEATAMNISRVAGVAPFEVCFSSSNIASTRLGPAVPSIDLVLQNQNVFWRMFGSNSMVEVNRDVLCLGFVNGGSNPTTSIVIGGHQLENTLLQFDLATSRLGFSATLLGRQTTCANFNFTSNA

116 Garb_15201 gi|224106732 predicted protein

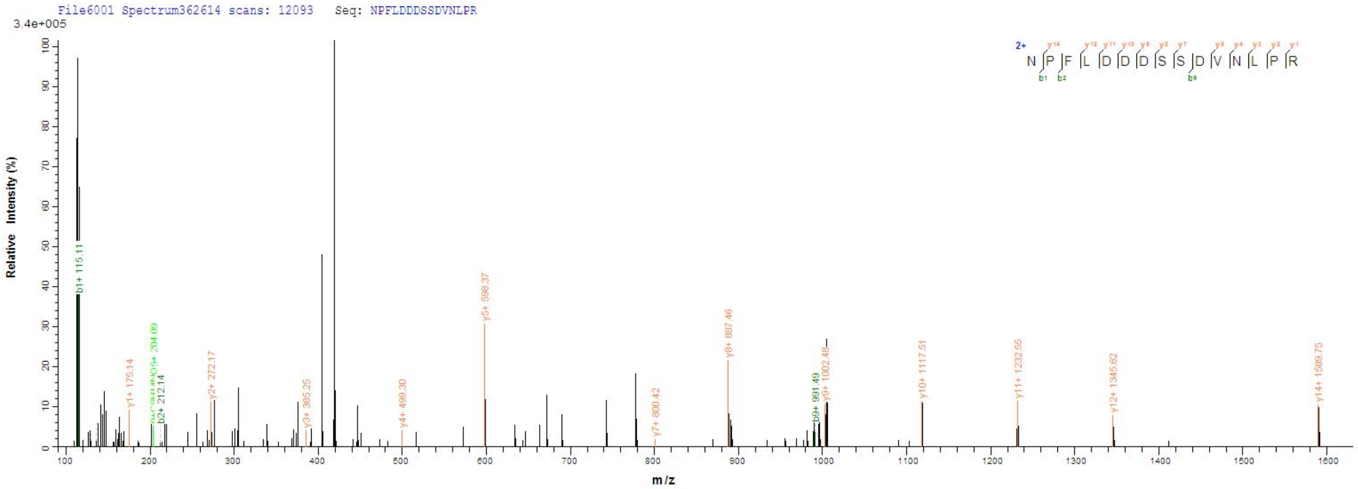

MFWKLTSLSASSPVESILDKENFTLEELLDEEEIIQECKALNSRLINFLRDRAQVEQLLRYVVEEPPEDADSKRVFKFPFI
ACEIFTCEIDVILKTLVEEEQLMNLLFSFLEPNRPHSALLAGYFSKVVVCLMLRKTVPIMNYVQVHQDVFRQLVDLIGIT
SIMEVLVRLVGGDDHVYPNFLDVMQWLADSNLLEMIVDKLSPSCPPEVHANAAETLCTITRNSPSALATKLSSPSFVAR
IFGHALENSHSKSGLIHSLSVCISLLDPKRSAIVSPLMHSFRHQHMYEPSIPVNSETVNAMLPKLGDLLMLLNVSSDEKI
LPTTYGDLRPPLGKHRLKIVEFIAVLLRIGNEAAEKELVNTGTIQRVIDLFFEYPYNNALHHHVGSIILSCLESKNDAIVD
HLLQECDLIGKFLQTDKNPVLSGDSNQPTLPAAGKCAPRVGNIGHITRISNKILQLGSSNSRIQACIQENSEWNEWQAN
VLQERNAVENVYRWACGRPTAFQDRTRDSDEDDLHDRDYDVAALANNLSQAFSYKIYGNDDNEEDHGGLGRDDEDV
YFDDESAEVVISSLRLSDDQGSSLFTNSNWFAFQDDKIGNEPMATSPTEVFDDVNLNGTANGGNSSSDDEVIVGEEDDK
NPFLDDDSSDVNLPRNIETVMTDVAQPSNGEPILANGSSDSMDMSDGSVSSNSSKKSPPSVRSLFEEDVEFVGVELEGT
EKAMEQALKEGIVGEAGPLKRNIIPHVPEKENSDEVGAGIKEFNDANYWRVDKEVAVSE

117 cotton_GLEAN_10014707 gi|297736988 unnamed protein product

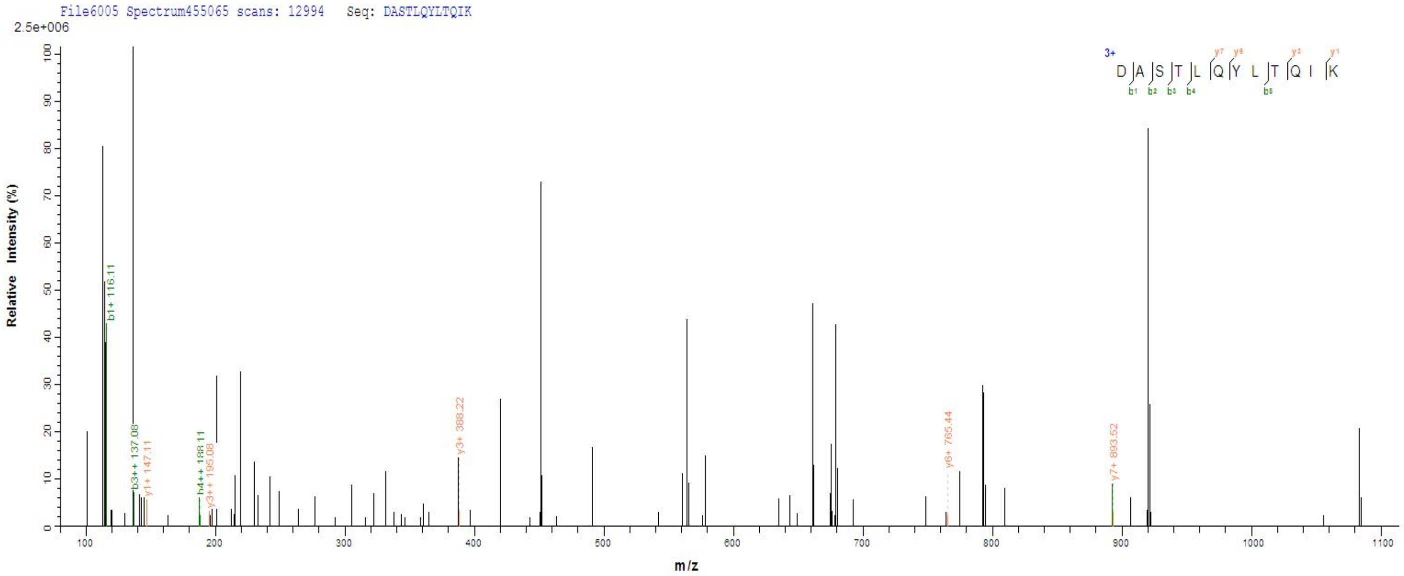

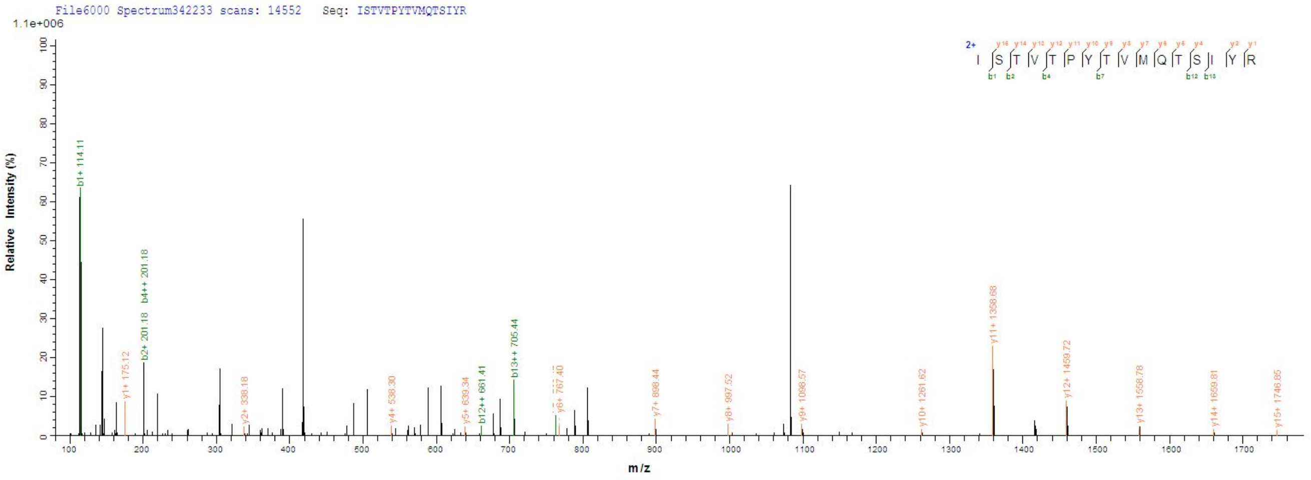

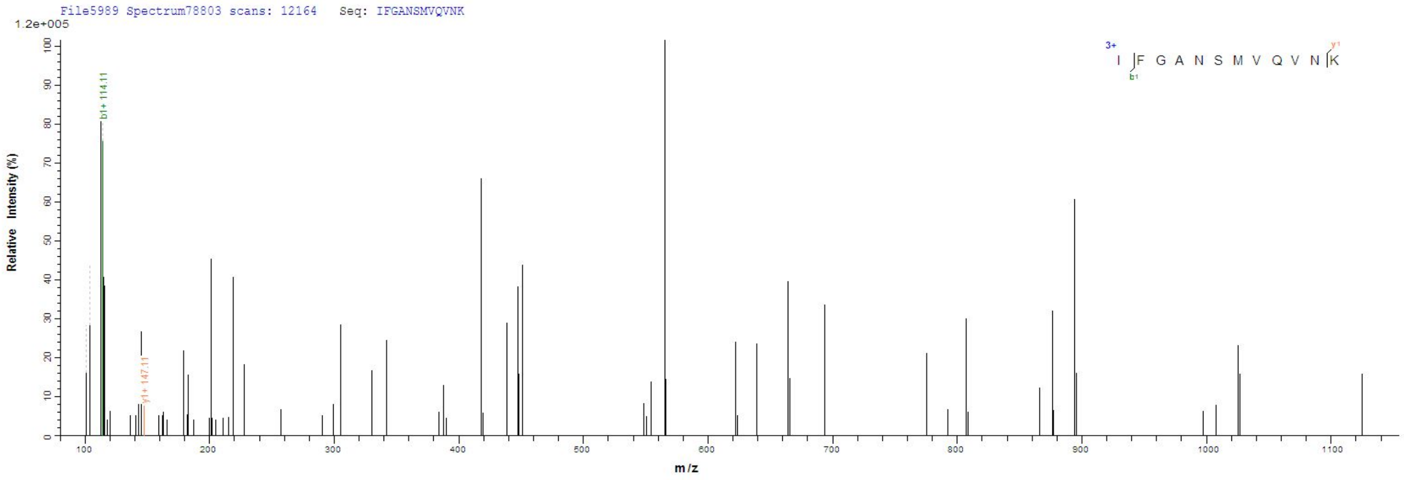

MVASSNFLLFSASLFFFIVSPSIQASFRPKALLLPVSKDASTLQYLTQIKQRTPLVPIKLTLDLGGEYLWVDCDQGYVSSS
YKPARCNSAQCNLARSKACGSCFDGPKPGCNNNTCSLLPSNSVKNSGTIGEVAQDVVSIQSTNGKNPGKEVTVSKFLF
TCGSSFLLDGLAGGVKGMAGLGRTKISMPSQFAAAFSFPRKFAVCLSSSSGSNGVVIFGDGPYNLLPDIDVSKSLMYTP
LILNRVSTSSASFQGDPSADYFIGVNGITINTKPVSFNKTLLSINKEGQGGTKISTVTPYTVMQTSIYRAVVNAFIKQTSK
VARVPAVAPFSACYNAKSLGSTRVGPAVPQIDLLLPNKVVWRIFGANSMVQVNKDVLCLGFVDGGLEPTTSIVIGGHQI
EDNLLQIDLAASKLGFSSSLLFRRTTCSNFNFTSTA

118 cotton_GLEAN_10010353 gi|225458697 uncharacterized protein

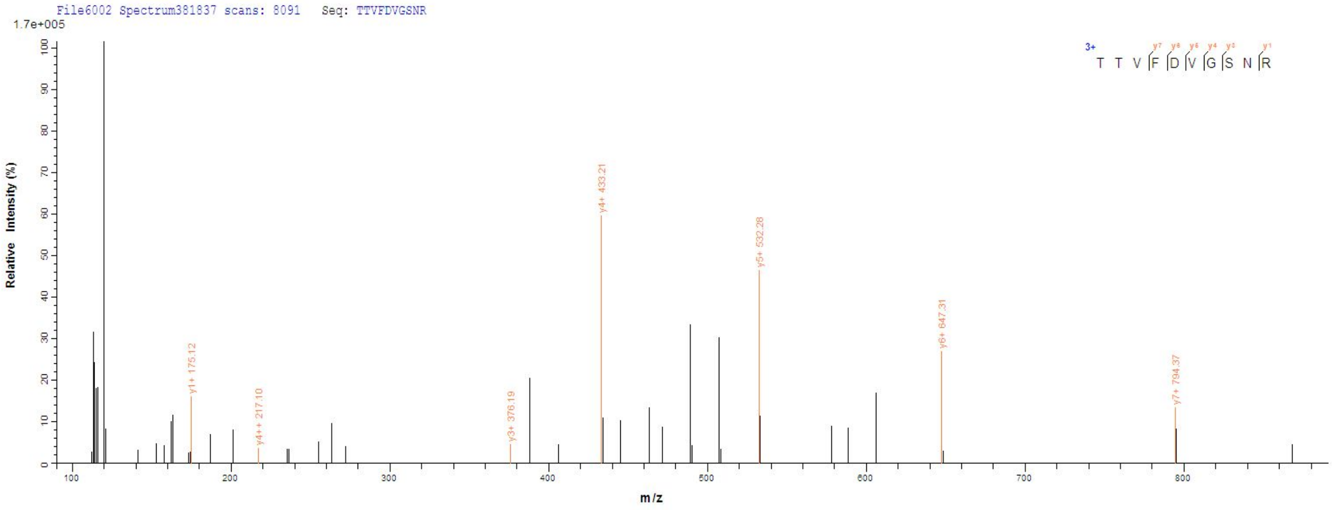

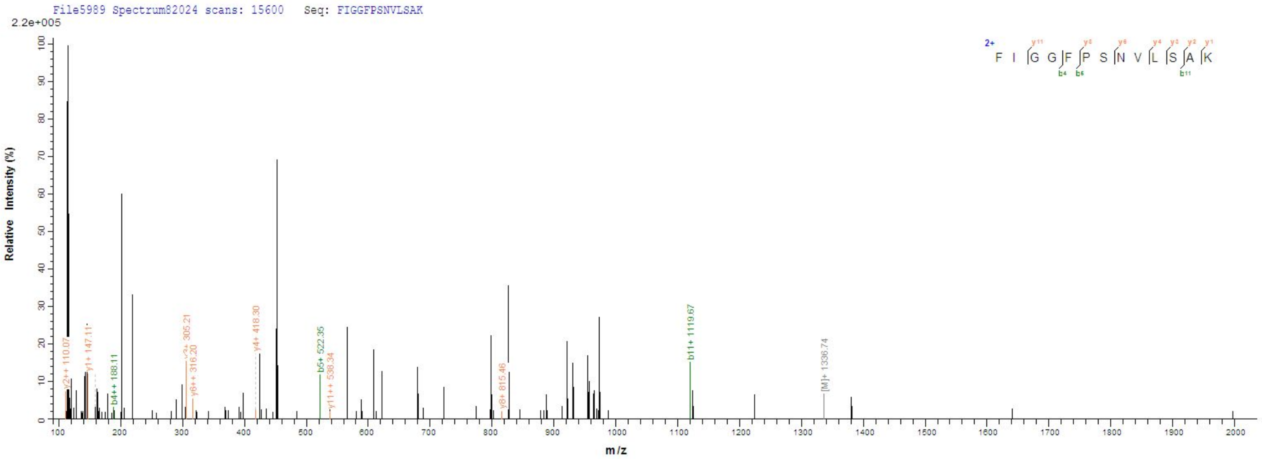

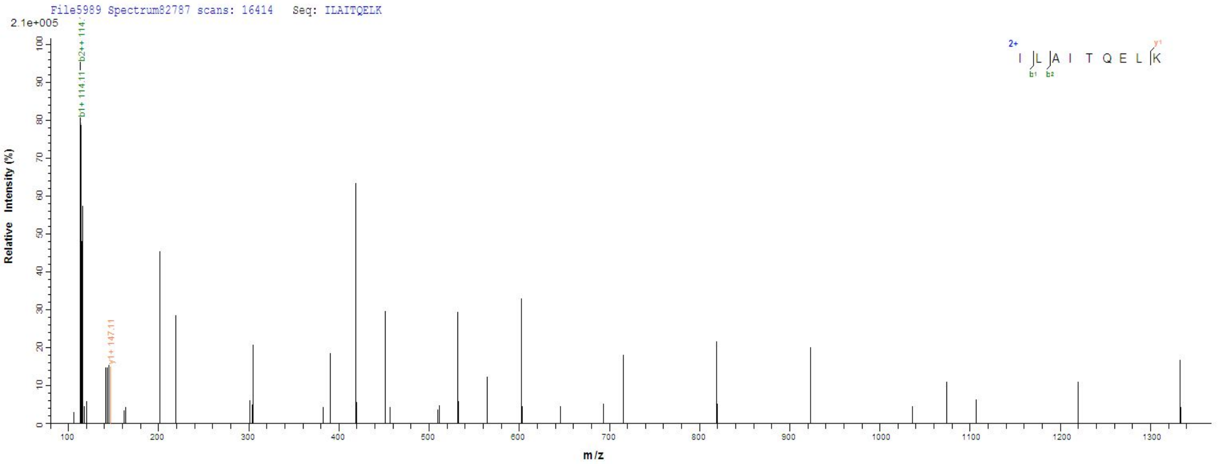

MVPKLLFSTFTLLITCFFGFGIAQPCPRPRPCPSISPPPRPTRTVSYVRPRPPLYDPSLRLLPSQPNNNPGPLANRARILAITQELKRNITFDPQNYTPTWVGNNYCLFKGFICDTVPDLNITGLAGIDFNGARFGGNLNFYRFIQNLPDIAIFHANSNNFSGVINQGLGGLRYFYEIDLSNNKFIGGFPSNVLSAKNLTFVDLRFNNYLGTIPRSLFNFDTDVLFINNNVFGQGIPRNLGNTPALYLTLANNNFNGTIPRSIGAAWETMTEVLLLGNKLSGCLPFEIGYLNRTTVFDVGSNRLTGPIPESFGCMAKLQLLNMAHNRFYGAVPEVLCRLPNAFNFTLSNNYFTQVGPQCRRLIKLRRLNVNRNCIMGLPFQRSATDCANFFSKPRSCARESTFSIIPCRLPAASLRTRTVFQEDEVAPSPKTYNALEKPPH

119 cotton_GLEAN_10029849 gi|147767808 gi|147767808

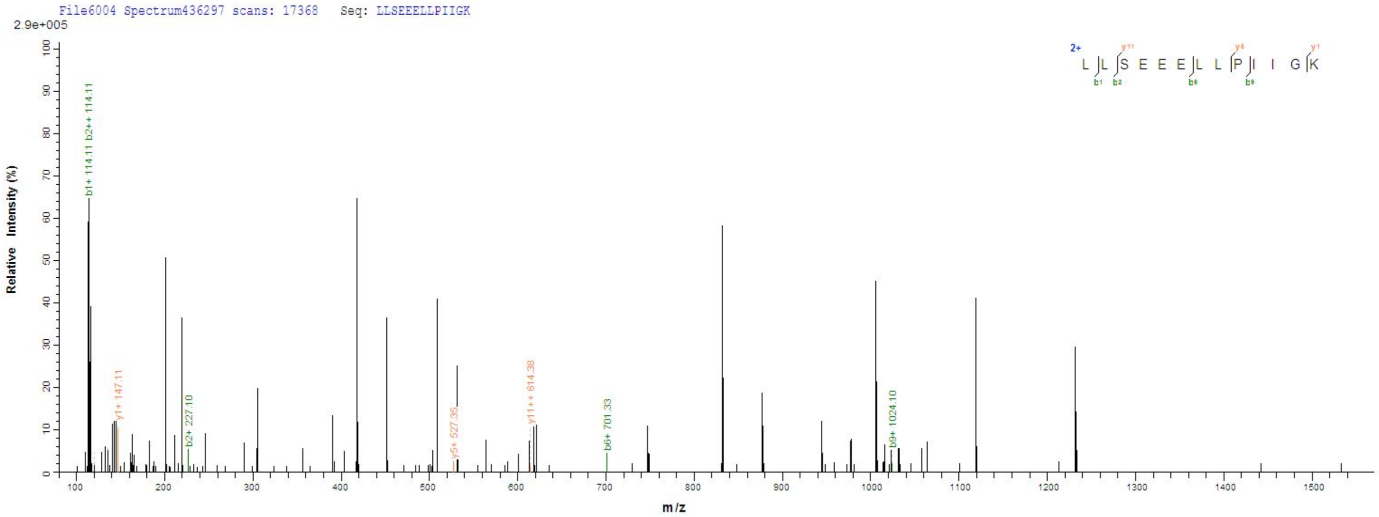

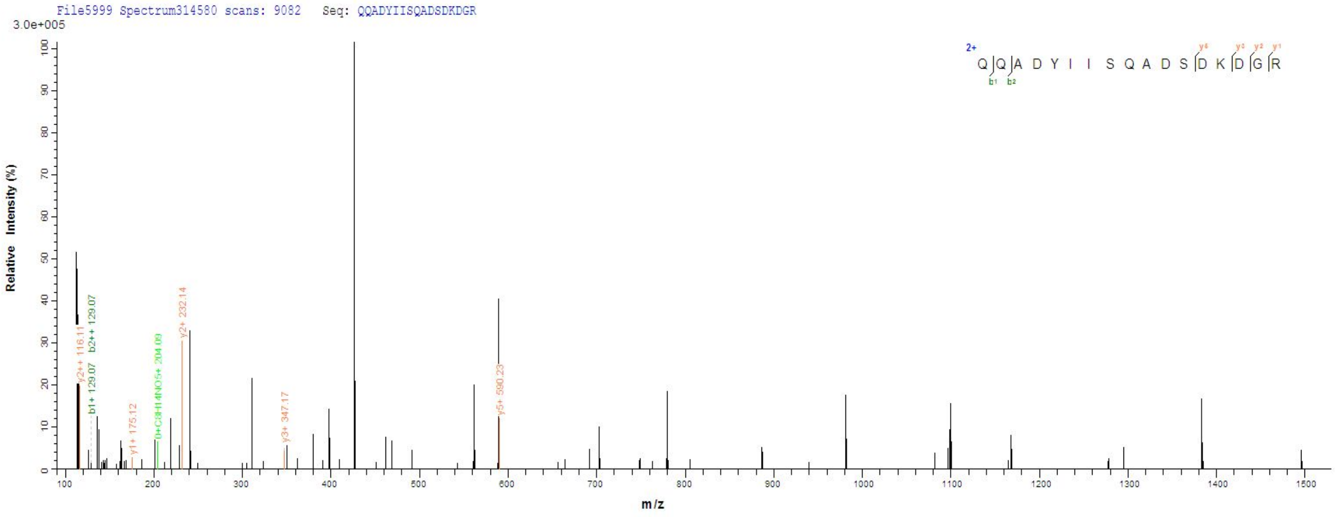

MGKVSAIIYIAVALLILFLISHSPKKHPNHSRHRRLKLRSSFSFSNPTHHEPVAFDPLVADIERRREDRQWEKQYLEHTHPELVNDHAPGHESQPEWEDFMNAEDYLNDEDKFNVTNRLVMLFPKIDADPADGFVTENELTEWHLQQAAKEVLHRSMREMEVHDKNHDGFVSFAEYEPPSWVKNDNNSFGYDMGWWKEEHFNASDANGDGLLNITEFNDFLHPSDSKSPKLLHWLCKEEVRERDTDRDGKVNFDEFFHGLFDLVRNYDEEDHNSSHPSHDSLESPARHLFSQLDKDGDRLLSEEELLPIIGKIHPSERYYAKQQADYIISQADSDKDGRLSLLEMIENPYVFYSAIFSEDEDDDDYEYHDEFR

120 cotton_GLEAN_10029653 gi|224092318 predicted protein

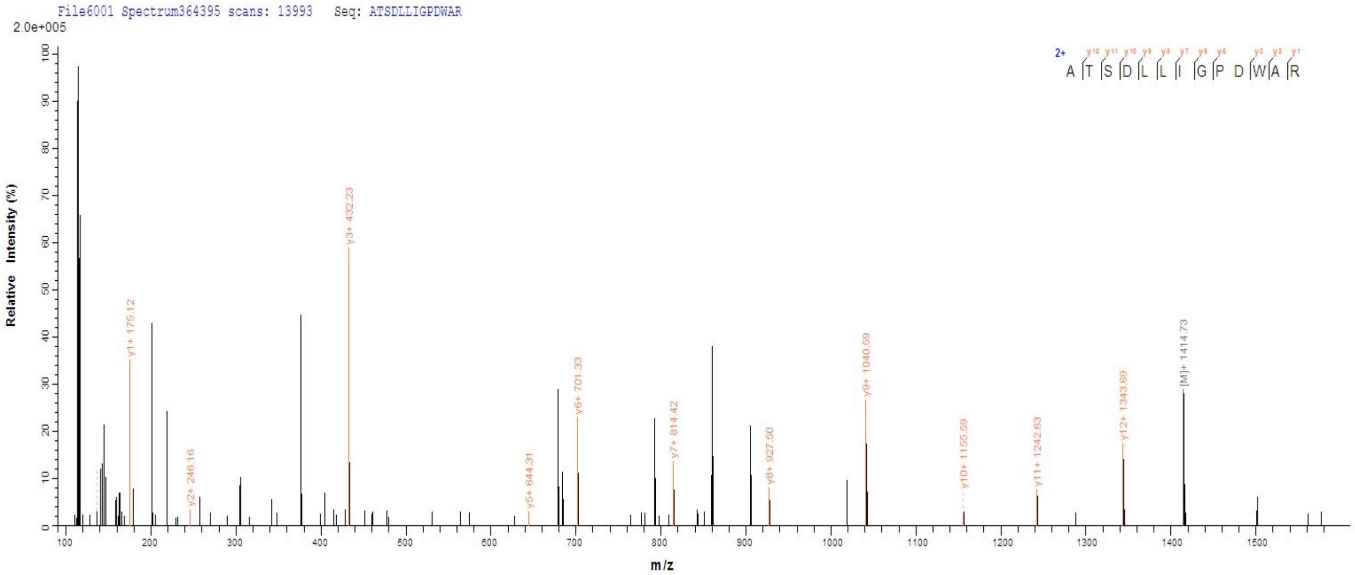

MVNSMVERATSDLLIGPDWARNIEICDMLNHDPGQAKDVVKGIKKKLGSKNPKVQLLALTLLETIIKNCGDIVHMHVAERDVLHEMVRIVKKKPDFNVKEKILTLIDTWQEAFGGARARYPQYYVAYQELLRLGAVFPPRSERSAPVLTPPQTQPLSSYPPNIRNSDRQDTAESSAESEFPTLSLTEIQNARGIMDVLSEMLNAIDPGNKEGLRQEVIVDLVEQCRTYKQRVVHLVNSTSDESLLCQGLALNDDLQRVLTRHEAIASGTPSQANKPKPEPAKELVNVDSPLIDTGDSSKQSEGRSTSSTVTSSPFNQLLLPAPPATNGSTPPPAANPKMDLLSGDDFNSPKADNSLALVSLGEPQQAPTASQQNALVLFDMFSDGSNTSNSVNTQSSGAESSGSLPPPPWEAQAADSSPVAGAQHPQYVGSDQMAGVYIQPTTTGHLPTTNNHVALGNQFAGYHPQPIQGAPQYTGMLPQQMPVGQMSSMYPQQMPTGQMGSMYPQQVPGAQMGSMYPQQMPAGQMGSMYPQQMYGNQMGAYGYGQQQYLNQQMYGLSIRDDNALRNSSYQVSTSSYVPPSKPSKPEDKLFGDLVDMAKIKSTKTTPGRAGSM

121 cotton_GLEAN_10037769 gi|147820236 hypothetical protein VITISV_010210

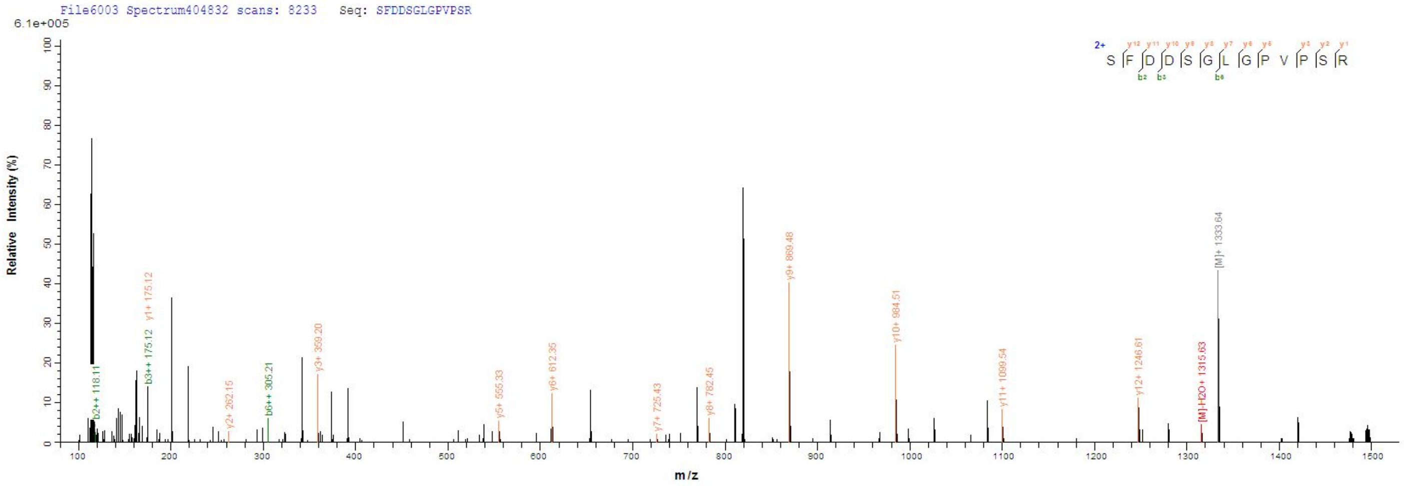

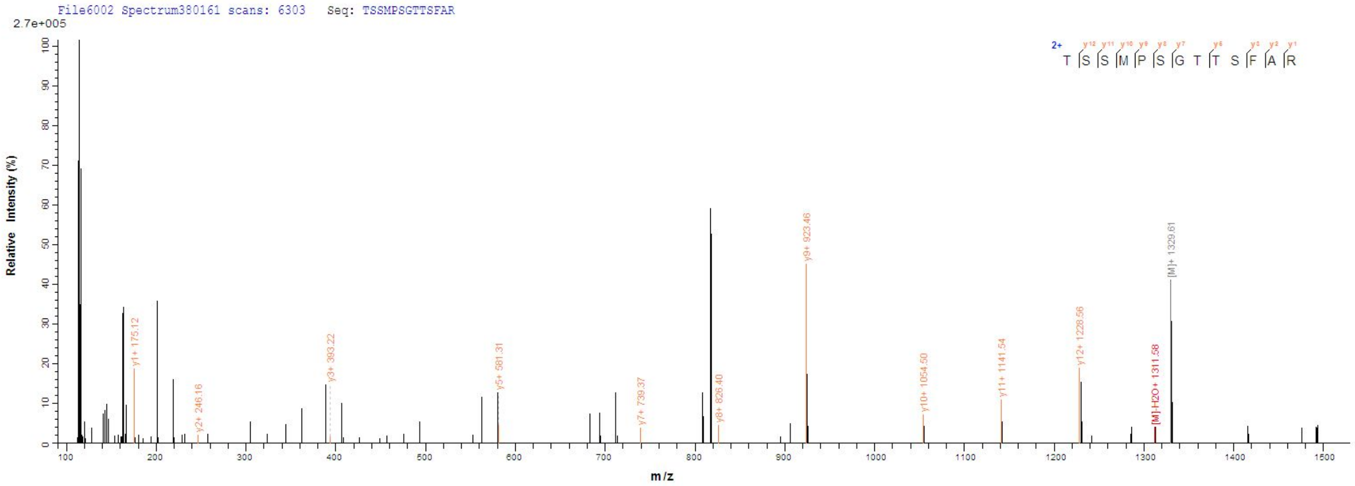

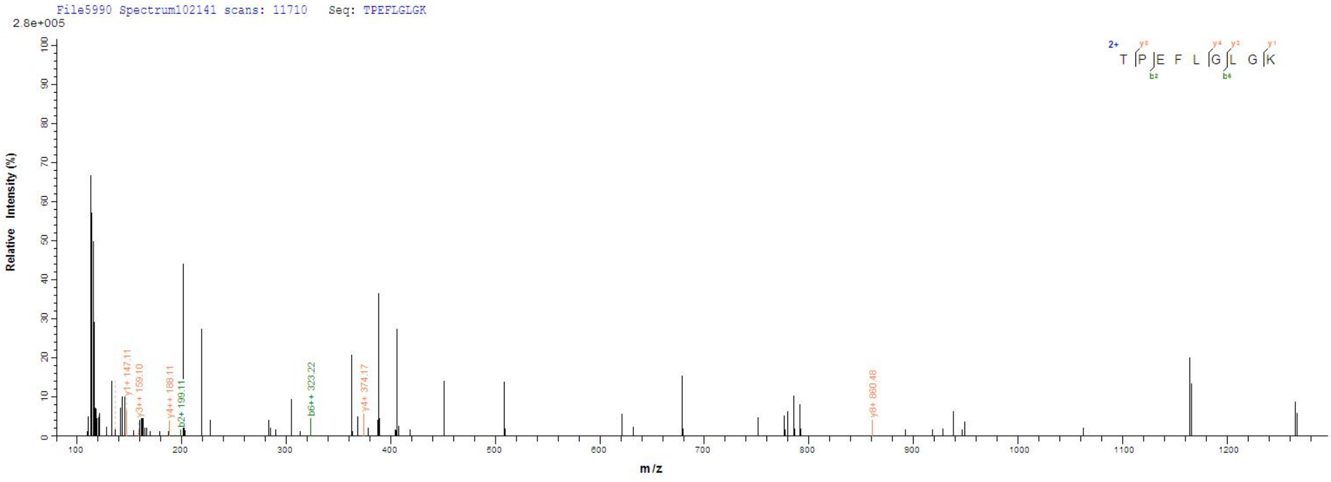

MKSLKFKLVEIFLFLSLYYVCIAAEEKIQKAKRTYIVHVDKSNMPETFTDHALWYDTSLKSVSDSASVLYRYENVVHG
YSTMLTTEEAEALGEQPGILSVLPEVIYELHTTRTPEFLGLGKSTTLFPTSDSMGEVIVGVLDTGVWPELKSFDDSGLGP
VPSRWKGECQVGKNFSASSCNRKLIGAKFFSKGYEVAFGPIDETMESRSPRDDDGHGTHTATTAAGSVVPSANLLGYA
SGTARGMASHARVAVYKVCWLGGCFGSDIVAAMDAAVADGVDVLSMSIGGGLSEYYSDTVAIGAFTAAANGIFVSCS
AGNGGPMPSSLSNVAPWITTVGAGTLDRDFPASITLGNSELHSGVTLYNGKQLSDSMVPLVYGGNVSNSSGGALCMA
GSLIPEKVARKIVVCDRGGSARVQKGVVVKDAGGIGMILTNTDTFGEELVADAHLLPSAAVGQKTGDAIKKYISSTRNP
TAKIGPGTTKLGVQPSPVVAAFSSRGPNPVTPAILKPDIIAPGVNILAGWTAAVGPTGLQSDPRHVNFNIISGTSMSCPHV

SGLAAIVKAAHPEWSPAAIKSALMTTAYTAYKTGQKIEDVATGGPATPFDYGAGHADPVAALDPGLVYDATIDDYLGFL
CALNYTPNQIKSTTHRDFTCQTSKKYTLGDFNYPSFSVPLETASGRRGGADSSSIIKYTRTLTNVGAPATYKVSLHSQTQ
AVKMSVEPETLSFKAQYEKKSYTVTFKTSSMPSGTTSFARLEWSDGKHIVGSPIAFSWT

122 Garb_09350 gi|217073300 unknown

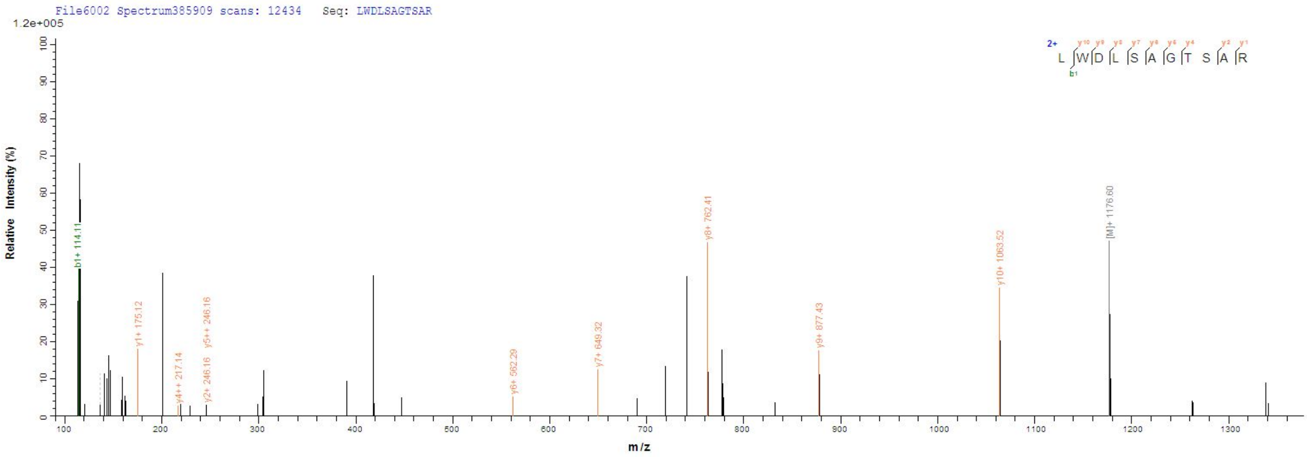

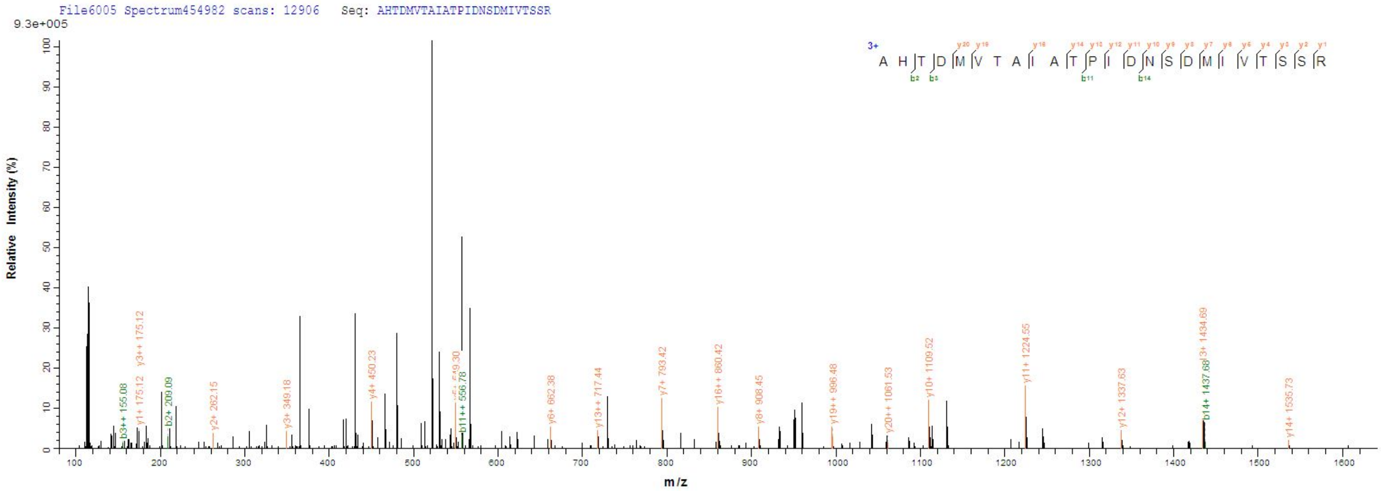

MAGSEGLILRGTMRAHTDMVTAIATPIDNSDMIVTSSRDKSIILWHLTKDEKTYGVPRRRLTGHSHFVQDVVLSSDSQF
ALSGSWDGELRLWDLSAGTSARRFVGHTKDVLSVAFSIDNRQIVSASRDRTIKLWNTLGECKYTIQEGDAHTDWVSCV
RFSPNTLQPTIVSASWDKTVKVWNLTNCKIRNTLAGHSGYVNTVAVSPDGSLCASGGKDGVILLWDLAEGKKLYSLD
AGSVIHSLCFSPNRYWLCAATEQGIKIWDLESKSIVEDLKVDLKAEAEKSDVTDIGNKKKVIYCTSLNWSADGSTLFSG
YTDGVIRVWGIGRY

123 cotton_GLEAN_10027063 gi|359496362 uncharacterized protein LOC100854560

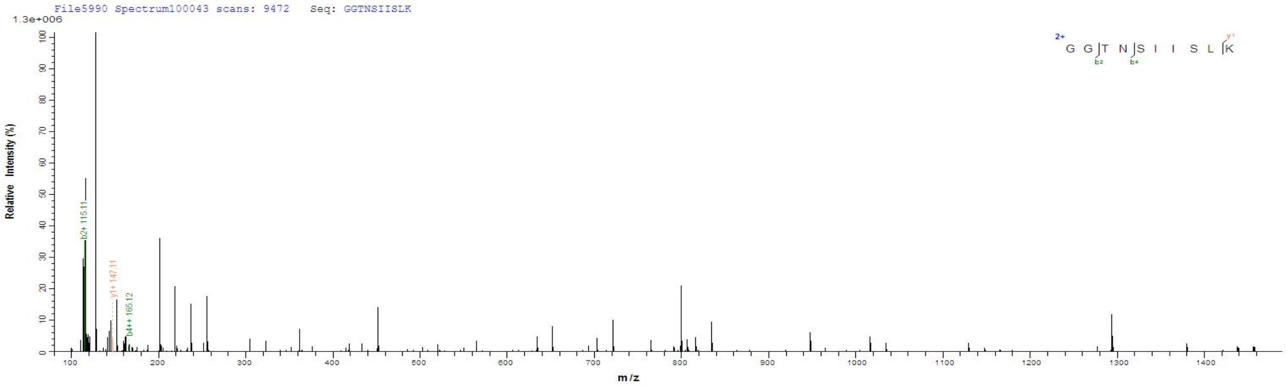

MATPKQLILPFFFLLSLSSIALSEGEDCVYTIYIRTGTIIKGGTNSIISLKLYDAKGEYVEIENLEAWGGLMGEGHDYYER
GNLDIFAGRGRCLASPVCAMNLTSDGTGPQHGWYCNYVEVTMTGIHTPCSQQMFAVEQWLAFDTLPFDLTAIRNYCS
AELRTNQQNSQEVPSRSST

124 Garb_04125 gi|388499178 unknown

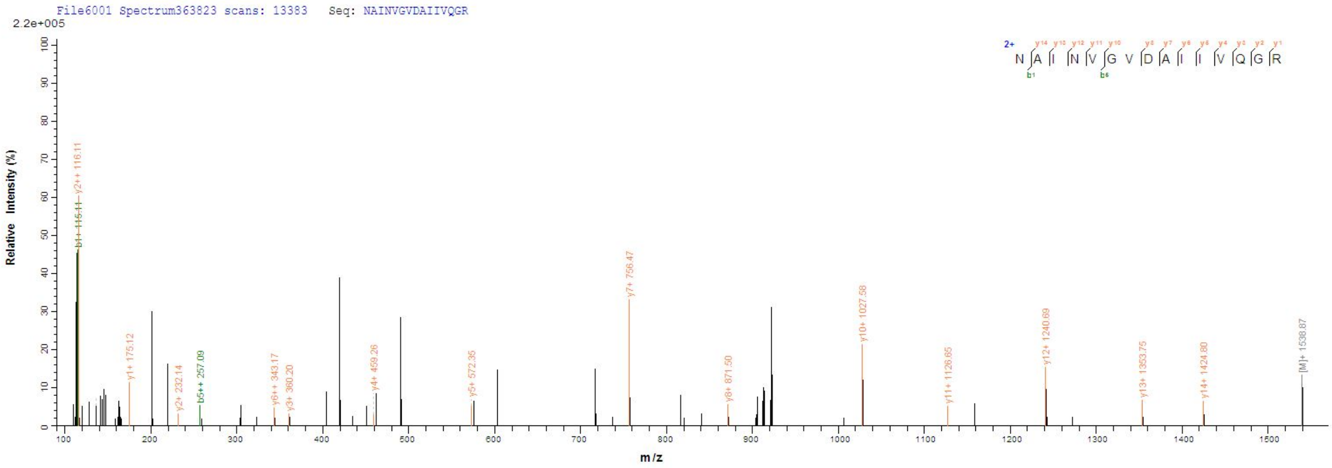

MGWKGILGFEYGIVQGPLGPDIAGPELVAAVANAGGLGLLRAPDWESPDYVKELIRKTRKLTDKPFGVGVVLAFPHKE
NVKAILEEKVAVLQLYWGECSKELVIEAHNAGVKVVPQVGSLEEAKNAINVGVDAIIVQGREAGGHVIGQEGLISLLPR
VVDLVGDHGIPIIAAGGIVDARGYVAALALGAKGICMGTRFLATHESYAHPTYKRKLIEYDKTEYTDVFGRARWPGAP
HRVLQTPFFCDWKYLSAQENETNQPIIGRTIIHGVEREIRRFAGTVPNPTTTGDIESMVMYAGQSVGLIKEILPAGQVVK

125 Garb_28296 gi|223943077 unknown

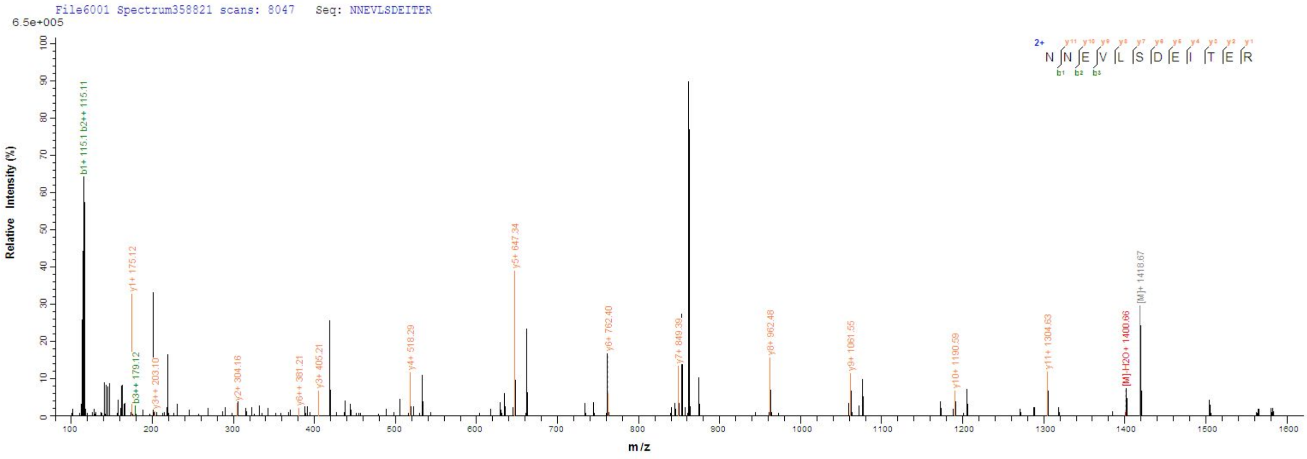

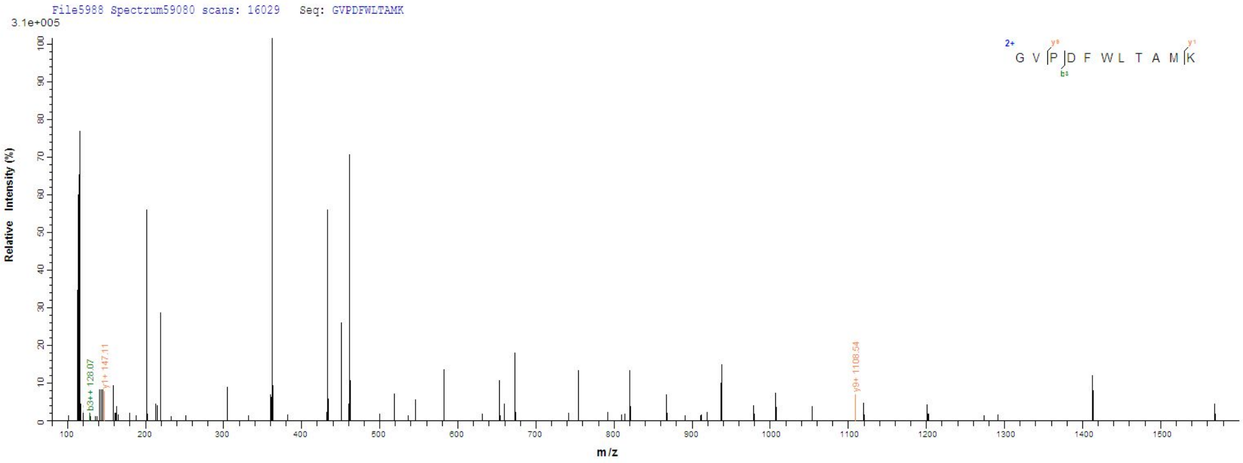

MSNEGVNFNMSGLGDALNAEARAGLVNALKNKLQSLAGDHSDVLESLSPIVRKRVEVLREIQACSFSLSFFDALLSFT
LISLMSQHDELEAKFFEERAALEAKYQKLYQPLYAKRYDIVNGLADAEGTANEAAKDQGEEKDAEEKGVPDFWLTAM
KNNEVLSDEITERDEGALKYLKDIKWYRVEEPKGFKLEFYFDTNPYFKNTVLTKTYLMIDEDEPILEKAIGTEIEWYPG
KCLTQKLLKKKPKKGSKNAKPITKTEECESFFNFFNPPQVPEDDEDIDEDTAEELQNQMEQDYDIGSTIRDKIIPHAVSW
FTGEAIQGDELDIDDDEEDDDDDDEEEEDDEEEDEDADDEDEDDDEGSKTKKKKSGKAVGDGQQGERPPECKQQ

126 cotton_GLEAN_10004093 gi|388500070 unknown known

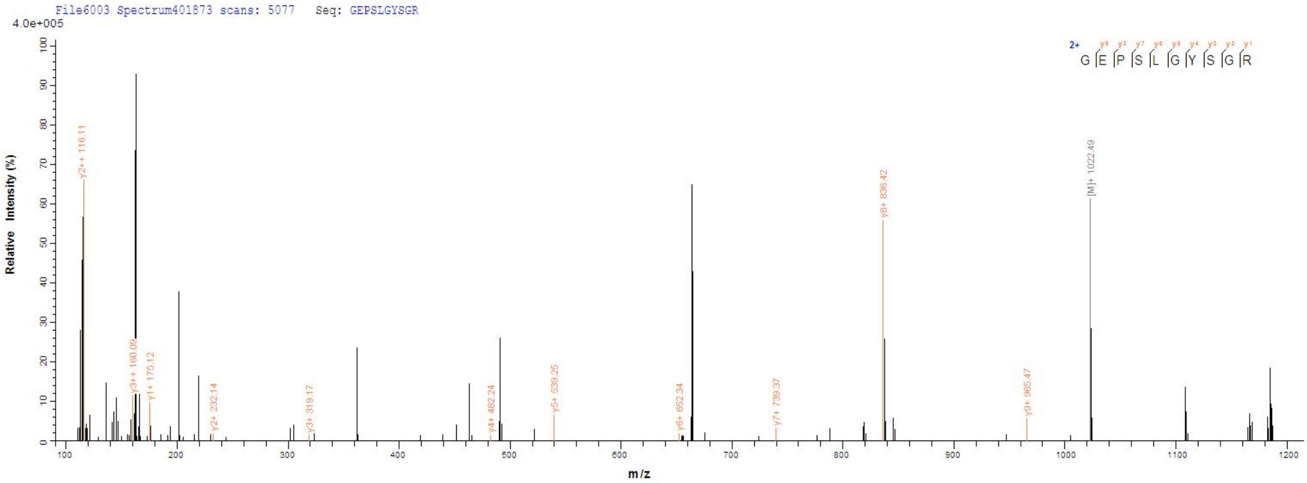

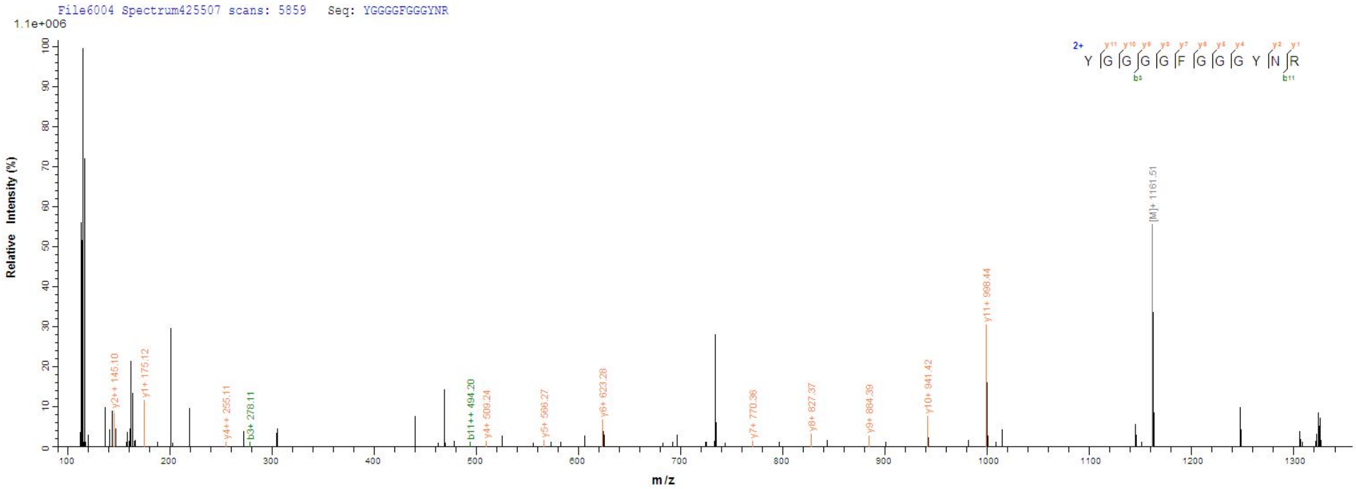

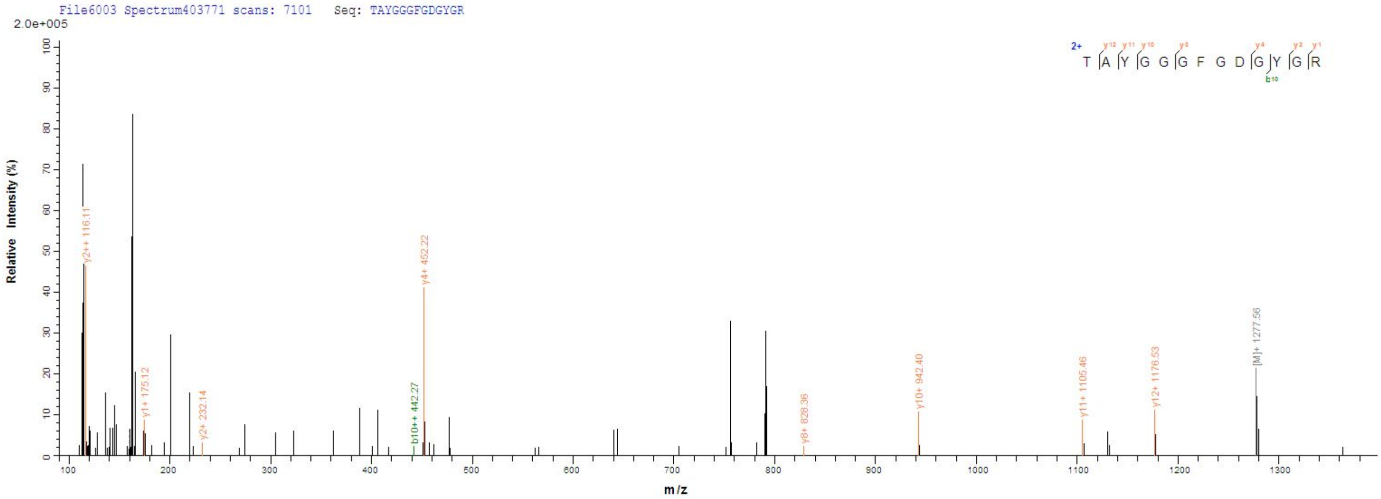

MDPRGGDAIPDGETNDFRSSDHPDDDKSHPHTGDGASPGKIFVGGLARETSSAQFLEHFGKYGEITDSVIMKDRKTGQ
PRGFGFVTYAEPSVVDKVIEDTHIINGKQVEIKRTIPKGAAGSKDFKTRKIFVGGIPSTVSEDEFKDFFTQYGVVREHQI
MRDHATNRSRGFGFITFETEQAVDDLLEKGNKIEFAGAQVEIKRAEPKKPNPPPPPSKRYNDSRTAYGGGFGDGYGRYG
GGGFGGGYNRSSGAYGGRAGGFGAYGGGEFGSYGGYGGGGSGGIGPYRGEPSLGYSGRYGGNFNRGYDMGSGYGG
PGEFYGGYGAGAAGGGYGSSYDAGLGGGYGGGAAGGSSFYGSRGGYSGAGSGRYHPYGR
127 Garb_20707 gi|84453208 putative cytosolic factor

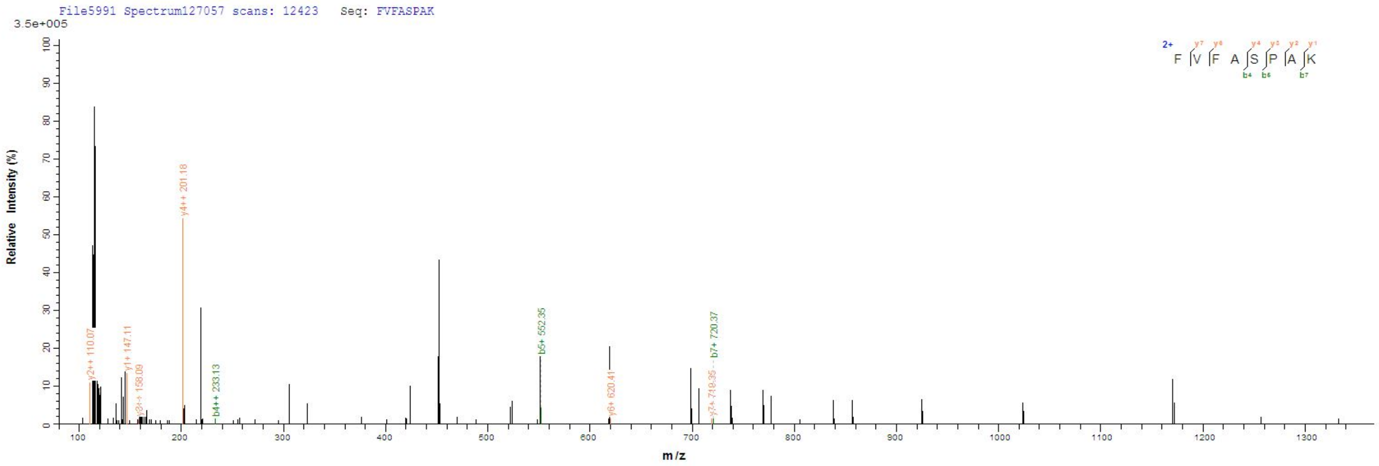

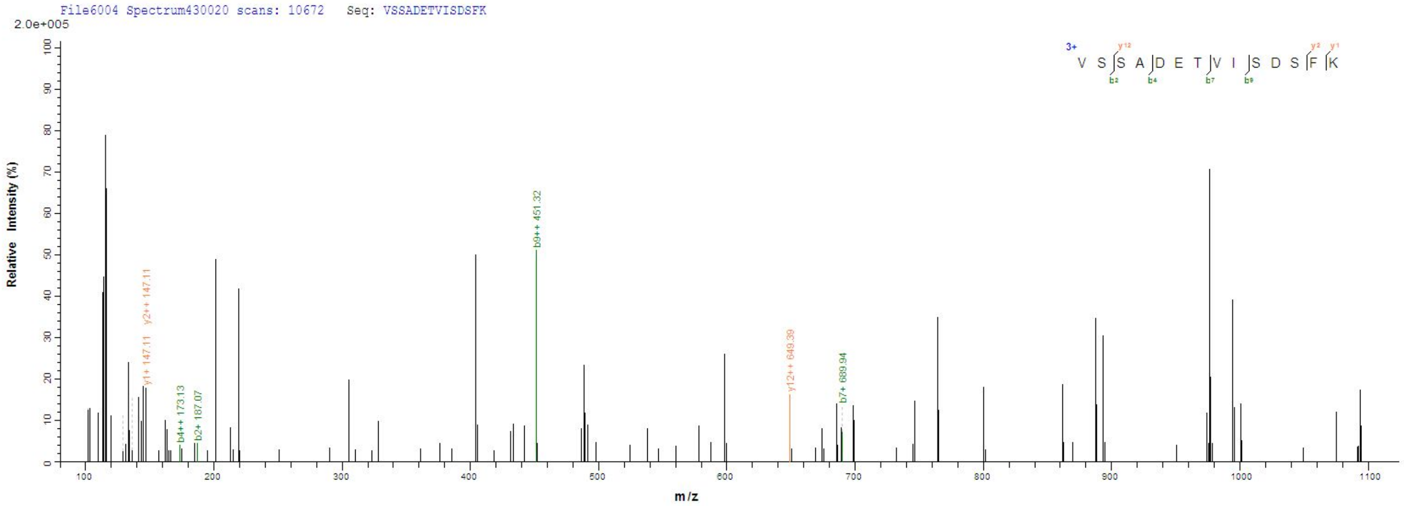

MAQETLDSESAPAAPAQEVSVVVEKPQVTEKEPQPSAPAPLPEPEVPEKPAVVAVEEEAVEVEKPKVEEKEETKITQSVS
FKEETNIAGELPEPQKKALDELKQLIQEALNNHEFTAKPKAEEKPVAEPEPEPKKEEEENKEEKKEEKEEETPAAEISEEP
KVSIWGIPLLADEKSDVILLKFLRARDFKVKDAFTMIKNTVSWRKEFGIETLLDEDLGNELEKVVFMQGFDKEGHPVC
YNVYGEFQNKELYQNTFADEEKRSKFLRWRIQFLEKSIRKLDFNPTGINTMVQVNDLKNSPGPGKKELRQATNQALNL
LQDNYPEFVAKQVFINVPWWYLAFNRMISPFLTQRTKSKFVFASPAKSAETLFKYIAPEQVPVQYGGLSREGEQEFSVA
DAVTEVTIKPAAKHSVEFPITENCNLVWELRVVGWEVNYGAEFVPTAEDGYTVIVSKTRKVSSADETVISDSFKTGEPG
KVVLTIDNQTSKKKKLLYRSKTKPYSD

128 Garb_28018 gi|147782603 hypothetical protein VITISV_010455

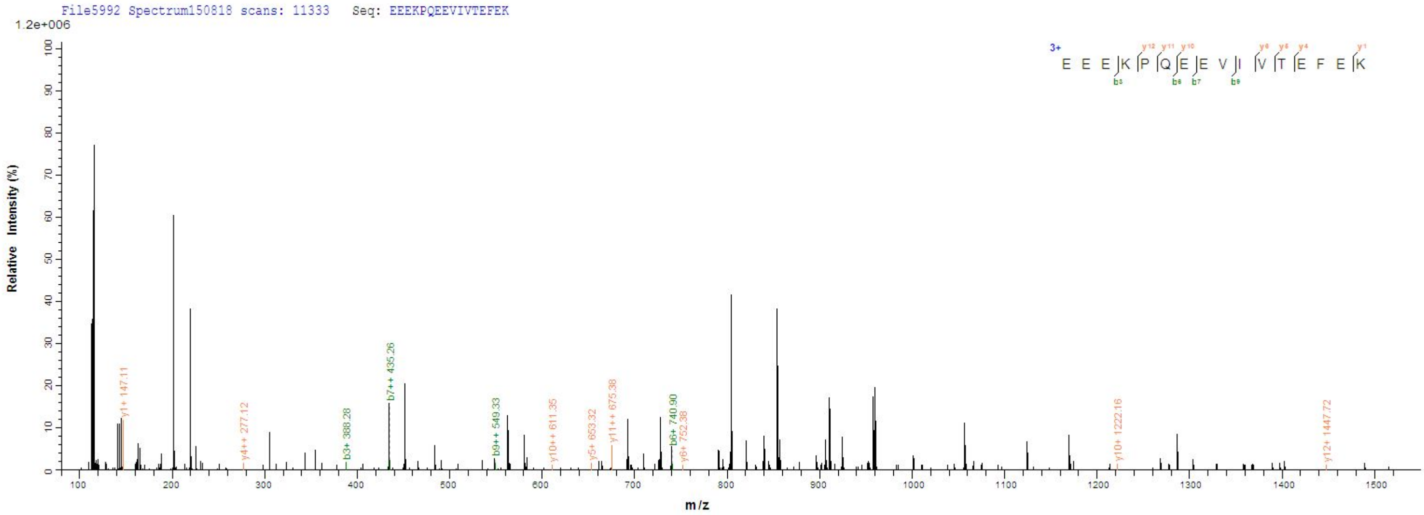

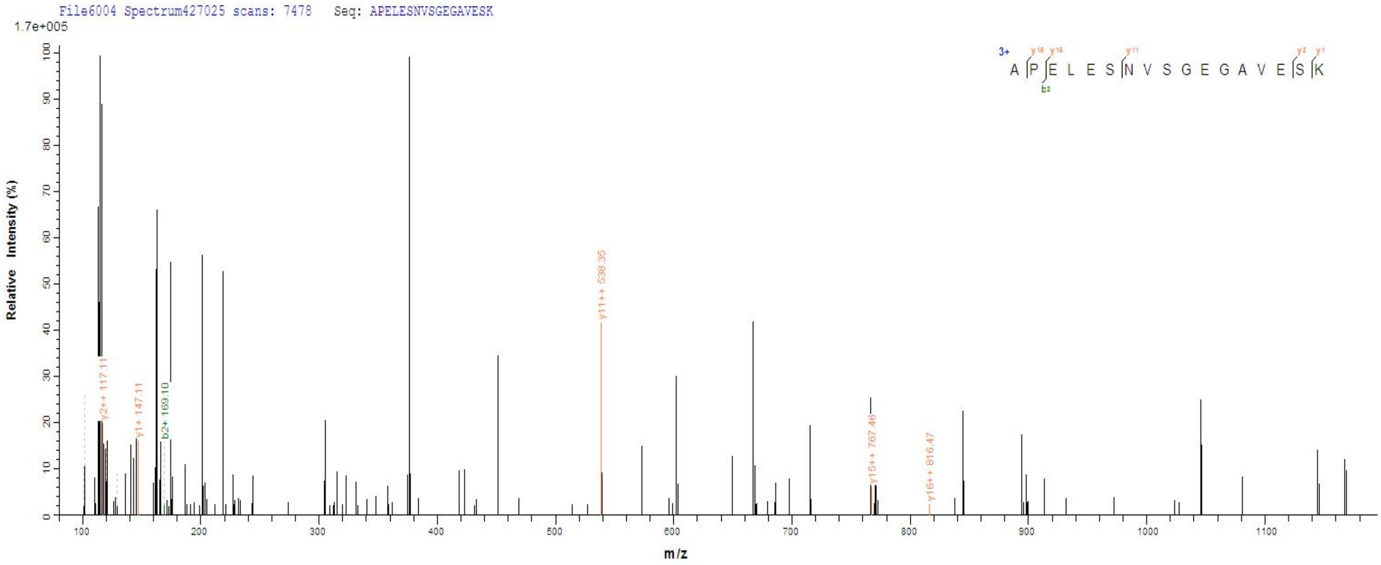

MAEEHTSKAPELESNVSGEGAVESKERGLFDFMGKKEEEKPQEEVIVTEFEKVNIEETKAEEEGEEKKKHGLLEKLHR SDSSSSSSSSDEEEGEGEEKKKKKKKEKKGLEEKIEEKLEGEKKEEDTSVPVEKCDEPVVQPETPEKKGFLEKIKEKLPG QHKKAEEASSPAPAPAAEPHHEEESKEKKGILEKIKEKLPGYHSKSDEEKEKA

Garb_ or cotton_: the accession of cotton AD genome annotation database
gi: NCBInr Accession
